# Photoinduced Nitroarenes as Versatile Anaerobic Oxidants for Accessing Carbonyl and Imine Derivatives

**DOI:** 10.1021/acs.orglett.3c02292

**Published:** 2023-08-24

**Authors:** Joshua K. Mitchell, Waseem A. Hussain, Ajay H. Bansode, Ryan M. O’Connor, Dan E. Wise, Michael H. Choe, Marvin Parasram

**Affiliations:** Department of Chemistry, New York University, New York, New York 10003, United States

## Abstract

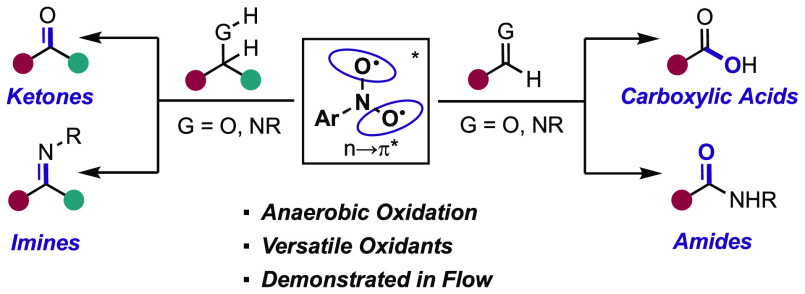

Herein, we report a protocol for the anaerobic oxidation of alcohols, amines, aldehydes, and imines promoted by photoexcited nitroarenes. Mechanistic studies support the idea that photoexcited nitroarenes undergo double hydrogen atom transfer (HAT) steps with alcohols and amines to provide the respective ketone and imine products. In the presence of aldehydes and imines, successive HAT and oxygen atom transfer (OAT) events occur to yield carboxylic acids and amides, respectively. This transformation is amenable to a continuous-photoflow setup, which led to reduced reaction times.

Oxidations of C(sp^3^)– and C(sp^2^)–heteroatom systems are essential transformations in organic chemistry (Scheme 1).^1^ Classical oxidation methods such as Jones,^2^ Swern,^3^ and Baeyer–Villiger^4^ are powerful; however, they are mostly conducted under super stoichiometric amounts of reagents. Furthermore, these reactions are often highly exothermic and can lead to undesired side products, like overoxidation, which limit the substrate scope (Scheme 1A). Hypervalent iodine-based reagents like IBX^5^ and DMP^6^ offer milder reaction conditions but are limited in large-scale applications due to the issues of solubility, cost, and explosive nature. Recently, oxidative approaches employing nitroxyl radicals can be achieved catalytically under milder aerobic or anaerobic conditions.^7−9^ The latter approach can lead to an expansion of the substrate scope that complements classical oxidation strategies. However, the employment of *N*-hydroxyl-based catalytic systems can suffer from the limitations of high catalyst loading and poor functional group tolerance.^7^ Hence, a complementary anaerobic oxidation protocol that is economical, practical, and sustainable is highly warranted.

**Scheme 1:**
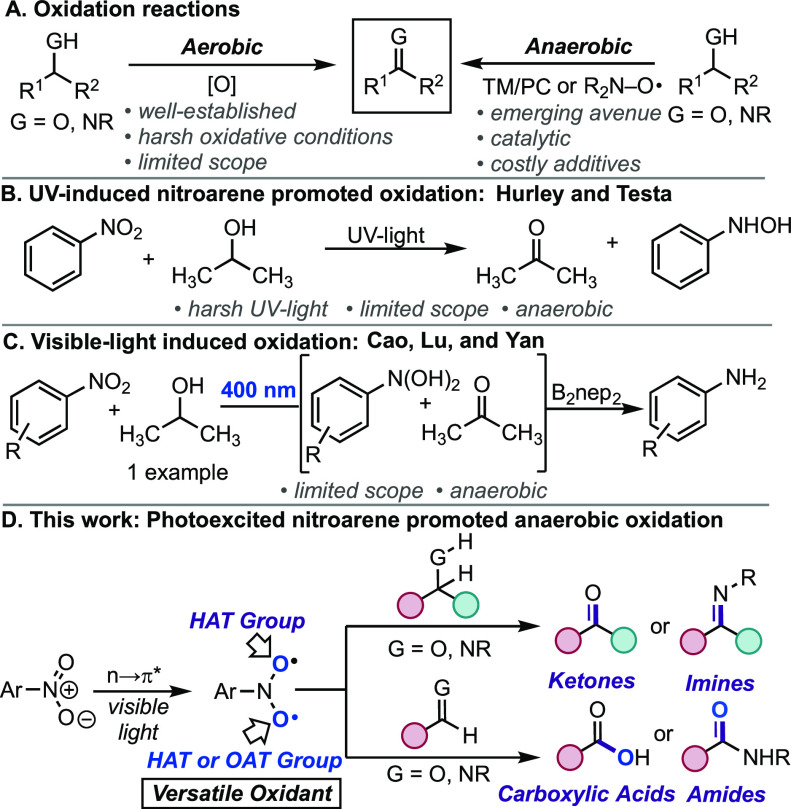
Prior Methods and Hypothesized Work

In 1966, Hurley and Testa (Scheme 1B)^10^ and others^11−13^ studied the intermolecular oxidation of alcohol solvents in the presence of nitroarenes under UV irradiation. The authors uncovered that two sequential hydrogen atom transfer (HAT) events occur during the redox event with an alcohol solvent. Very recently, the groups of Cao, Lu, and Yan redirected the aforementioned reactivity toward the visible-light region for the photoreduction of nitroarenes with concomitant oxidation (Scheme 1C).^14,15^ Though limited in scope, both approaches illustrate that photoinduced nitroarenes are capable of anaerobic alcohol oxidation.^13,16,17^ Based on our previous work on hydrocarbon oxidation using nitroarene photochemistry,^18−20^ we hypothesized the possibility of harnessing multiple HAT events with nitroarenes to promote the anaerobic oxidation of heteroatom systems under visible-light irradiation. Herein, we illustrate that the photoexcited state of the nitroarene can trigger a double HAT event with C(sp^3^)–heteroatom systems to generate valuable ketone and imines and a successive HAT and oxygen atom transfer (OAT) event at C(sp^2^)–heteroatom systems to furnish synthetically useful carboxylic acids and amides in a general, mild, and cost-effective manner compared to established oxidation protocols.

We began our investigation by testing the conversion of 1-phenylpentan-1-ol **1a** to ketone **3a** under our previously reported conditions featuring 2-chloro-4-nitropyridine under 390 nm.^19^ The oxidation was successful, resulting in a 61% yield of **3a**. After an extensive optimization campaign (Tables S1–4), the yield was increased to 91% with 3,5-bis(trifluoromethyl)nitrobenzene (**5**) under 390 nm irradiation at 0.1 mmol scale. After the optimized reaction conditions were discovered, the electronic effect of the oxidation reaction was investigated with 4-substituted-phenyl-1-ethanol derivatives (Table 1A, **1b**–**g**). It was found that the transformation was not sensitive to the electronic pattern, as substrates possessing both electron-rich and -deficient groups resulted in good to excellent yields of the oxidation products **3b**–**g**. This could be attributed to small differences in the bond dissociation energy for α-C(sp^3^)–H of electronically different alcohols. *Meta*- and *ortho-*substituted benzylic alcohols were also tested. **1h,i,k** gave **3h,i,k** in low to good yields; however, to our surprise, **1j** did not convert. We believe that hydrogen bonding between the O–H and *ortho* F-substituent in **1j** may strengthen the α-C(sp^3^)–H bond and disfavor HAT with the photoexcited nitroarene.^21^ Cyclic benzylic alcohol systems, such as indanol and tetrahydronaphthalenol, resulted in a moderate yield of oxidation products **3l**–**m**. Acyclic α-substituted benzylic alcohols containing sensitive and important functional handles, such as cyclopropyl **1n**, halogen **1p**–**r**, and carbonyl groups **1t**, all resulted in corresponding oxidation products in good yields under conditions B (**3o**–**r**) and A (**3t**). Notably, secondary benzylic alcohol **1s** and **1u** possessing a free aliphatic alcohol unit underwent site-selectivity oxidation at the benzylic position (**3u**), which typically cannot be accessed from the Stevens-Stahl protocol.^7−9^ Haloperidol (**3v**), a common antipsychotic,^22^ was synthesized from **1v** under this protocol in 61% yield. Finally, diaryl substituted ketones of medicinal relevance (**3w,x**)^23,24^ as well as halogenated heterocycle **3y** were afforded in low to good yields under the reaction conditions, highlighting the synthetic utility for late-stage oxidation.

**Table 1:**
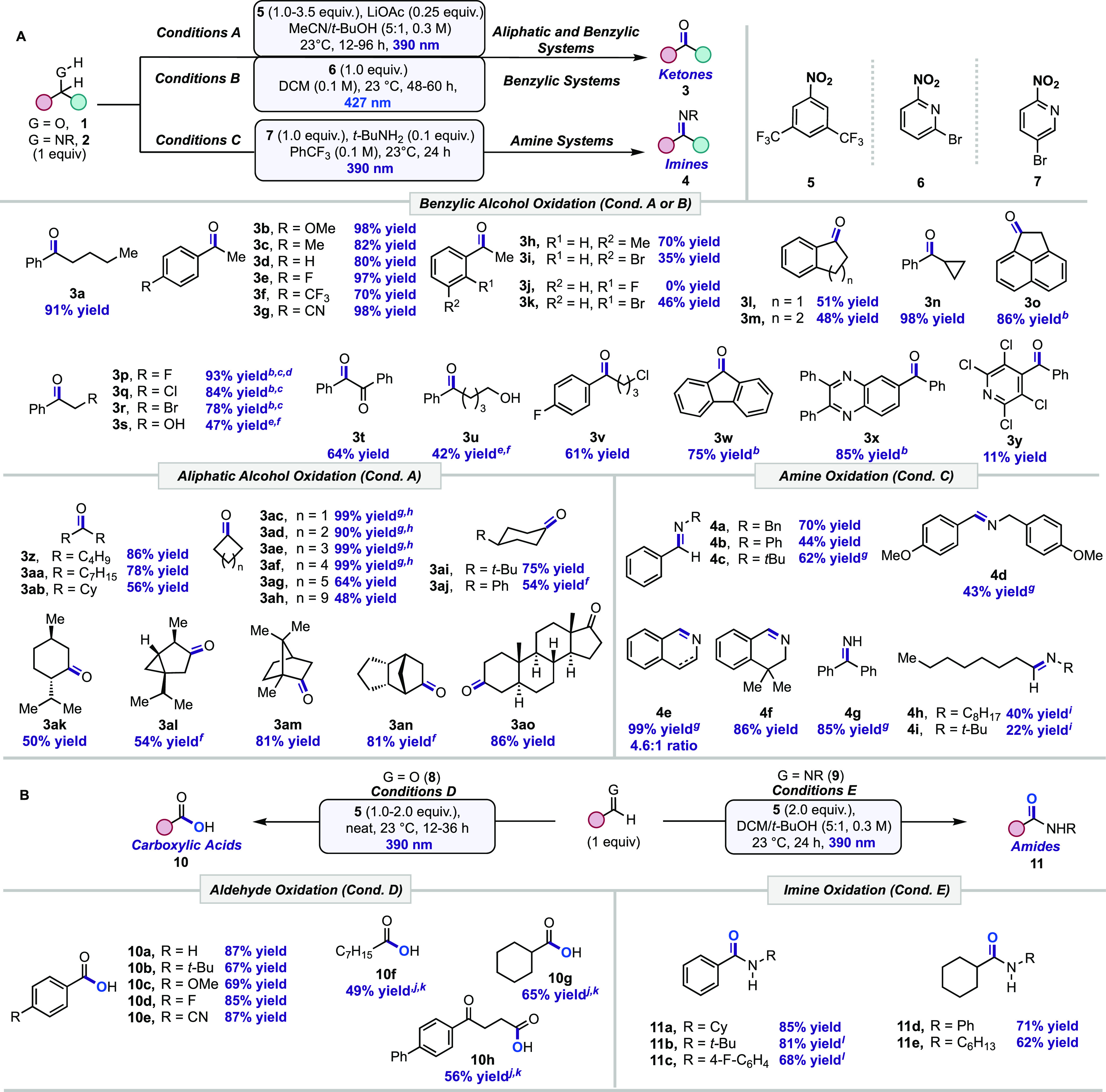
Scope of the Photoinduced Nitroarene Promoted Oxidation of (A) Alcohols and Amines as Well as (B) Aldehydes and Imines^a^

a Table 1A,B. Isolated yields.

b Conditions B.

c Using 2-bromo-4-nitropyridine.

d Under 390 nm.

e In MeCN/H_2_O (1:1, 0.3 M).

f No LiOAc.

g Denotes ^1^H NMR yield using CH_2_Br_2_ as an internal standard.

h Isolated as a hydrazone derivative (see SI).

i Denotes ^1^H NMR yield using CH_2_Cl_2_ as an internal standard.

j MeCN (1 M).

k H_2_O (1.0 equiv).

l Neat.

Next, unactivated aliphatic alcohols were studied under conditions A (Table S5). Secondary acyclic alcohols containing linear hydrocarbon chains yielded the oxidation products with good efficiency under the reaction conditions (**3z**, **3aa**). However, sterically encumbered dicyclohexylmethanol **1ab** resulted in a moderate yield of **3ab**. Cyclic alcohols featuring small to large ring sizes gave good to excellent yields under the reaction conditions (**3ac**–**3ag**); however, a decreased yield of 48% for **3ah** was observed due to the overoxidation of secondary C(sp^3^)–H sites. Next, 4-substituted cyclohexanol substrates were exposed to the reaction conditions, resulting in fair to moderate yields of desired ketone products **3ai**–**3aj**. The oxidations of naturally occurring terpenes **1ak**–**1an** and steroid **1ao** were tested. Oxidation of L-(−)-menthol and thujone precursor proceeded moderately well **3ak**–**3al**, while the oxidation of borneol was highly efficient under the reaction conditions **3am**. Corodane (**3an**) was obtained in 81% yield via the oxidation of **1an** under our conditions, which is comparable to the Jones oxidation^25^ (84%) and Stahl’s protocol^26^ (78%). Lastly, the reaction of *trans*-androsterone **1ao** generated **3ao** in 86% yield.

Then, we investigated if amines (**2**)^27−29^ could be oxidized in the presence of photoexcited nitroarenes (Table 1A, **2** → **4**). Exposure of conditions A and B to dibenzyl amine **2a** resulted in a low yield of the desired oxidation product **4a**. Further optimization revealed the use of nitroarene **7** in PhCF_3_ as a solvent under 390 nm irradiation led to higher yields (Conditions C, Table S6–7). Other benzylic amines **2b**–**c** were tested, giving the corresponding imines **4b**–**c** from moderate to good yields. Electron-rich amine **2d** was tolerated under the reaction conditions, but **4d** was prone to hydrolysis. Cyclic amines **2e** and **2f** gave the corresponding imines in good yields (**4e**–**f**). Amine **2e** led to the dihydroisoquinoline **4e** and overoxidized aromatic isoquinoline (**4e′**) in a 4.6:1 ratio with a 99% total NMR yield. Free amine **2g** gave the corresponding imine **4g** in 85% NMR yield, whereas reported oxidation of free primary amines can result in undesirable homocoupling.^30^ Furthermore, aliphatic amines reacted quickly and generated the desired oxidation product **4h**–**i** in low yield with concomitant overoxidation to the amide (*vide infra*).

Classical transformations for the oxidation of C(sp^2^)–heteroatom systems, such as aldehydes and imines, would often suffer from low reactivity as well as poor substrate scope and require the use hazardous oxidants and expensive additives or transition metals.^31−38^ Hence, we questioned whether the oxidation of aldehydes (**8**) and imines (**9**) could be achieved under our mild protocol (Table 1B). It was discovered that the employment of nitroarene **5** under 390 nm irradiation promoted the effective oxidation of aldehydes to acids (**8** → **10**, Conditions D, Tables S8–9). Oxidation of benzaldehyde **8a** resulted in an 87% yield of benzoic acid **10a** under the optimized conditions. Varying the electronic pattern of aromatic aldehydes did not affect the reaction yields (**10b**–**e**). Oxidation of octanal **8f** and cyclohexanecarboxaldehyde **8g** afforded the corresponding products **10f** and **10g** in 49 and 65% yields, respectively. To illustrate the synthetic utility of the transformation, the synthesis of therapeutic fenbufen^39,40^ (**10h**) was achieved in 56% yield via oxidation of **8h**. Finally, the oxidation of imines to amides was examined (**9** → **11**). Under conditions E (Table S10), *N*-cyclohexyl-1-phenylmethanimine (**9a**) afforded *N*-cyclohexylbenzamide (**11a**) in 85% yield. Benzyl imines such as *N*-alkyl (**9b**) and aryl imine (**9c**) generated the expected amide products (**11b**–**c**) in a good yield. Aliphatic imines containing *N*-phenyl and -hexyl substituents were subjected to the reaction conditions and resulted in 71 and 62% yields of amides **11d** and **11e**, respectively.

While the reported approach provides a complementary method to existing oxidations, the extended reaction times provide an opportunity for improvement. We postulated that reduced reaction times could be achieved under continuous-photoflow conditions (Scheme 2).^41^ A flow reactor consisted of a syringe pump to control residence time (*t*_R_), and a coil of fluorinated ethylene propylene (FEP) Teflon tubing irradiated by two LED lamps (general procedure F, see SI) was used to test the oxidation of one representative molecule from each substrate class assessed in batch (Scheme 2). Markedly, it was found that in all cases a 4- to 25-fold productivity improvement in mmol/h of the desired products was obtained leading to reduced reaction times.

**Scheme 2:**
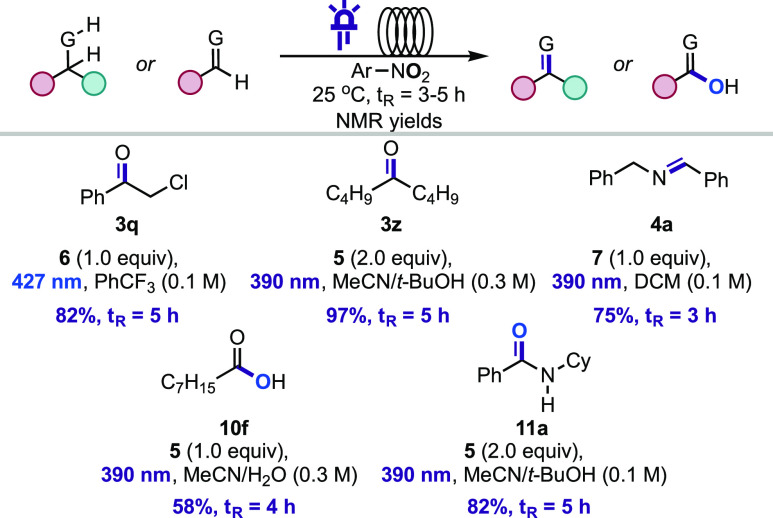
Continuous-Photoflow Oxidations

Based on the mechanistic studies from our lab,^18−20^ and others,^42,43^ the following mechanism is proposed (Scheme 3). Visible-light irradiation of the nitroarene **12** results in triplet diradical intermediate^19,44^**13** that engages in HAT of the α-C(sp^3^)–H bond of **1** or **2** to generate α-hydroxyl radical **15** and *N*-hydroxy-*N*-phenylhydroxylamine radical **14**. Kinetic isotope effect (KIE) studies^9^ and a radical clock probe test^45^ support that HAT participates in the rate-limiting step of the transformation and the formation of the α-hydroxyl radical intermediate, respectively (see SI). Subsequent HAT of intermediates **14** and **15** results in the desired oxidation products **3** or **4** and *N*-phenylhydroxylamine byproduct (see SI). An alternative pathway involving recombination of **14** and **15** and successive fragmentation leading to the oxidation products (**3** or **4**) is not supported based on ^18^O-labeling studies (see SI). For oxidation of C(sp^2^)–heteroatom systems, we propose that the HAT of **8** or **9** yields acyl radical **17** and *N*-hydroxy-*N*-phenylhydroxylamine radical **16**. Radical recombination of the latter intermediates generates the OAT products **10** or **11** and condensation byproducts stemming from the nitrosoarene.^46^

**Scheme 3:**
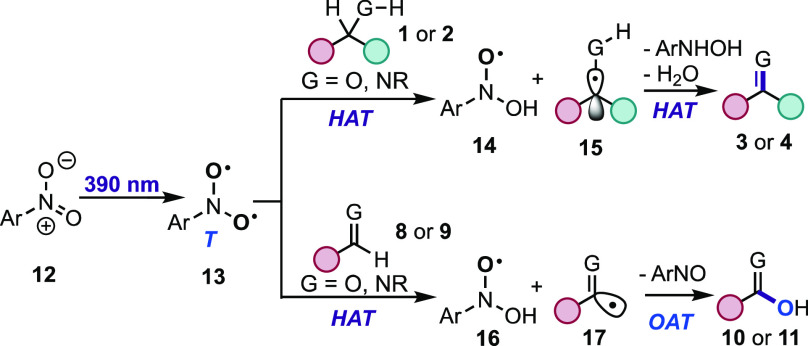
Proposed Mechanism

In conclusion, we have illustrated that nitroarenes are potent photo-oxidants capable of oxidizing C(sp^3^)– and C(sp^2^)– heteroatom systems to generate synthetically useful ketones, imines, carboxylic acids, and amides with good reaction efficiency. Notably, our transformation can target vicinal and extended diols, contrary to aerobic *N*-hydroxyl-based protocols. Furthermore, we are able to oxidize free amines to imines without homocoupling and produce amides from imines under milder conditions. Also, this approach precludes the use of precious transition metals and expensive additives, thereby providing an opportunity for late-stage oxidation of medicinally relevant compounds in a cost-effective manner. The synthetic utility of the transformation is highlighted by its amenability to continuous-photoflow setup. Due to the anaerobic nature of the transformation and the practicality of using nitroarene oxidants, this protocol provides a sustainable alternative complementary to established oxidation methods.

## Data Availability

The data underlying this study are available in the published article and its Supporting Information.

## Supporting Information Available

Experimental details, optimization studies, characterization data, and NMR spectra (PDF)
