# Supplementary material for: Photoinduced Nitroarenes as Versatile Anaerobic Oxidants for Accessing Carbonyl and Imine Derivatives
Source: Org Lett. 2023 Aug 24;25(35):6517–21. doi: 10.1021/acs.orglett.3c02292 (PMC10496125; doi:10.1021/acs.orglett.3c02292)
Supplement: Supplementary file 1 — ol3c02292_si_001.pdf [file ol3c02292_si_001.pdf]

Photoinduced Nitroarenes as Versatile Anaerobic Oxidants for Accessing Carbonyl and Imine Derivatives

Joshua K. Mitchell,[‡] Waseem A. Hussain,[‡] Ajay H. Bansode,[§] Ryan M. O'Connor,[§] Dan E. Wise, Michael H. Choe, and Marvin Parasram*

Department of Chemistry, New York University, 24 Waverly Place, 3rd floor, New York, NY 10003.

Supporting Information

Table of Contents

General Information S2
Optimization of the reaction parameters for the oxidation of benzylic alcohols. S3
Optimization of the reaction parameters for the oxidation of unactivated alcohols. S6
Optimization of the reaction parameters for the oxidation of amines. S8
Optimization of the reaction parameters for the oxidation of aldehydes. S9
Optimization of the reaction parameters for the oxidation of imines. S11
General Procedures S12
Characterization of oxidized products S16
Characterization data of ketones S16
Characterization data of imines S30
Characterization data of carboxylic acids S32
Characterization data of amides S34
Photo-Flow reaction data S36
Mechanism studies S38
Kinetic isotope effect studies S38
Radical clock study S40
Labeling study S42
Hammett plot study S44
Pinacol probe study S45
Light on/off study S47
Discussion of mechanistic studies S48
NMR spectra S49
NMR yield spectra S108
Continuous-Flow NMR yield spectra S113
References S116

General Information

All requisite chemicals were purchased from Fisher Scientific, Sigma Aldrich (Merck), Oakwood Chemical (Oakwood Products), Ambeed, TCI, and used without further purification unless otherwise stated. ^{1}H NMR spectra were recorded at 400 and 500 MHz, ^{13}C NMR spectra were obtained at 126 MHz, and ^{19}F NMR spectra at 377 MHz. All NMR were obtained on a Bruker 400 and 500 MHz Advance spectrometer and are referenced to the deuterated solvent resonance. Chemical shifts (δ) are reported in parts per million (ppm), multiplicity (s = singlet, br = broad, d= doublet, t = triplet, q = quartet, qt = quintet, sext = sextet, m = multiplet), and coupling constants (J) are in Hertz (Hz). All reactions were carried out under an inert nitrogen atmosphere in glassware that had been either flame-dried under vacuum or oven-dried unless otherwise noted. Thin-layer chromatography (TLC) was performed on 250-μm glass-backed silica gel plates and column chromatography were performed using 200–300 mesh silica gel. Solvents were dried and deoxygenated by passing through alumina in a solvent purification system. Deuterated chloroform ($CDCl_3$), dimethyl sulfoxide (DMSO-d_6), and acetonitrile (CD_3CN) were purchased from Cambridge Isotopes. GC chromatograms were taken on an Agilent 8890 GC with 5977B MSD, and helium as the carrier gas. High-resolution mass spectra (HRMS) were obtained on an Agilent 6224 TOF LC/MS which was acquired through the support of New York University. We utilized 34 W Kessil Lamps with varying wavelengths for our photochemical set ups.

Optimization of the reaction parameters for the oxidation of benzylic alcohols.

Table S1. Solvent screen for the anaerobic oxidation of benzylic alcohols to ketones.

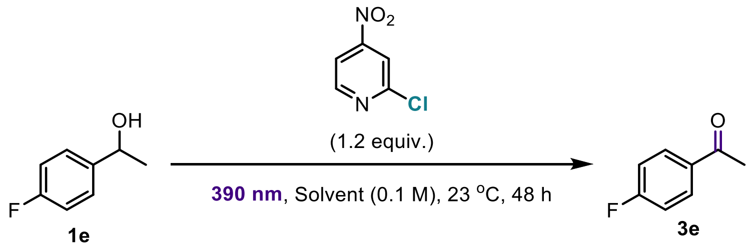

Entry	*Conditions*	*Conversion*[a]	***3e***[b]
1	**DCM**	**95%**	**88%**
2	$CHCl_3$	79%	65%
3	CCl_4	>99%	54%
4	**$PhCF_3$**	**90%**	**89%**
5	Acetone	75%	74%
6	$MeNO_2$	72%	58%
7	MeCN	75%	70%
8	Benzene	50%	50%
9	HFB	72%	50%
10	Cyrene™	0%	0%

Reactions were performed on a 0.1 mmol scale. [a]Conversion of **1e**. [b]Denotes ^{1}H NMR yields of **3e** using CH_2Br_2 as an internal standard.

*Solvents found to react with the photoexcited nitroarene: DMSO, DCE, 1,4, dioxane, MTBE, THF, DMA, Toluene, IPA, EtOAc, and EtOH.

Table S2. Control experiments for the anaerobic oxidation of alcohols to ketones.

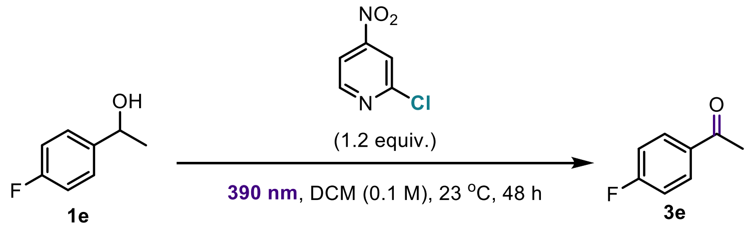

Entry	*Conditions*	*Conversion*[a]	***3e***[b]
1	-20°C	0%	0%
2	0°C	21%	16%
3	dark	0%	0%
4	degassed	>99%	>99%[c]
5	Air	76%	73%
6	N_2	95%	88%
7	N_2	>99%	>99%[c]

Reactions were performed on a 0.1 mmol scale. [a]Conversion of 1e. [b]Denotes ^{1}H NMR yields of **3e** using CH_2Br_2 as an internal standard. [c]Using 4-methoxyphenyl methyl carbinol.

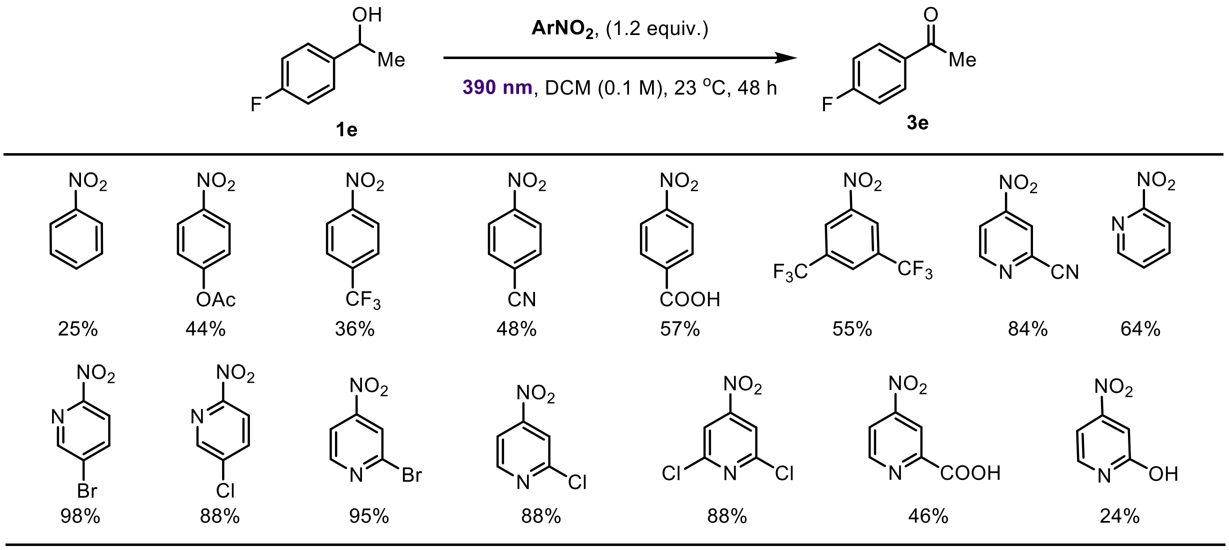

Figure S1. Nitroarene screen for oxidation of benzylic alcohols. Reactions were performed on a 0.1 mmol scale. Yields of **3e** were determined by ^{1}H NMR using CH_2Br_2 as an internal standard.

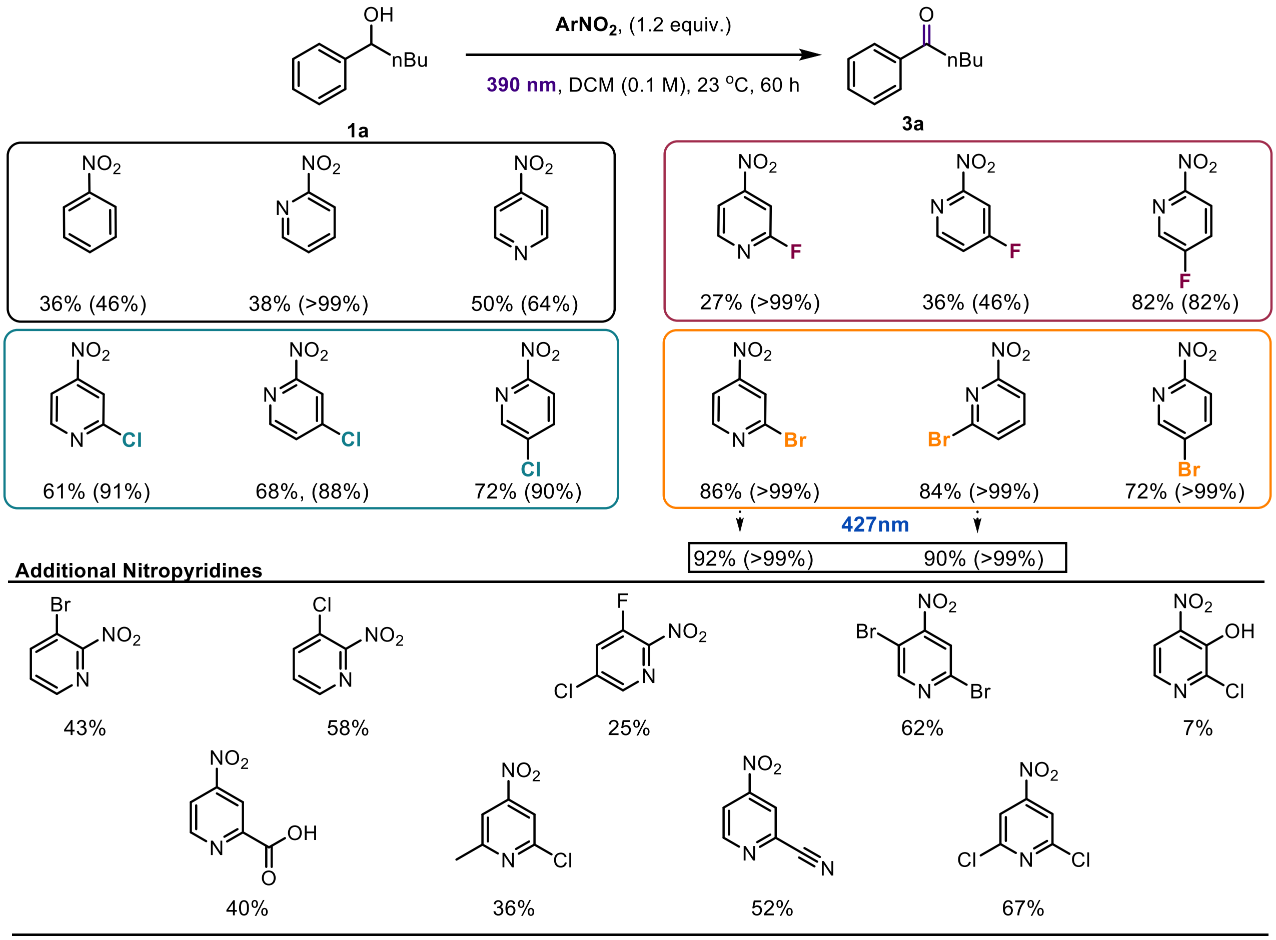

Figure S2. Nitropyridine screen for the oxidation of benzylic alcohols. Reactions performed on a 0.1 mmol scale. Yields of **3d** were determined by ^{1}H NMR using CH_2Br_2 as an internal standard.

A) Anaerobic Oxidations with hydroxylaniline

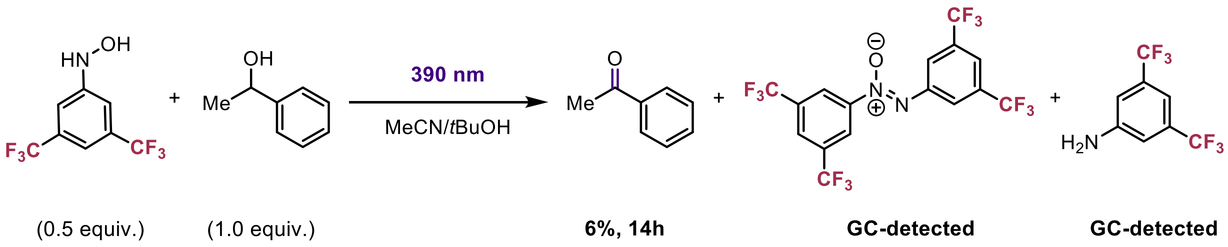

B) Anaerobic Oxidations with nitrosoarene

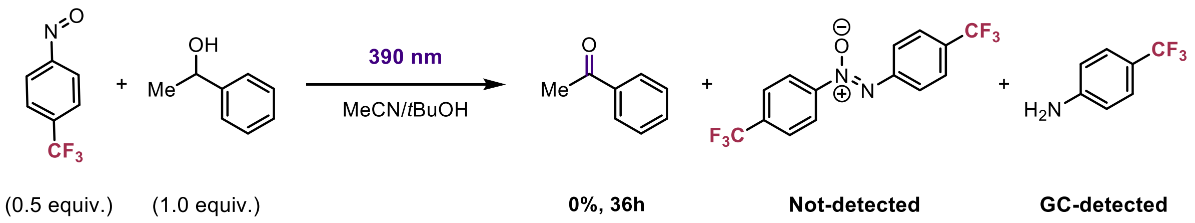

C) Anaerobic Oxidations with azoxyarene

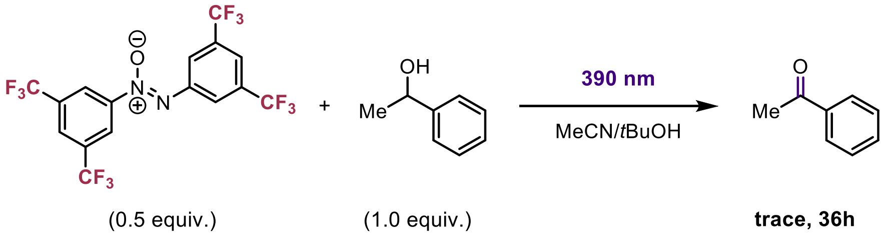

D) Anaerobic Oxidations with aniline

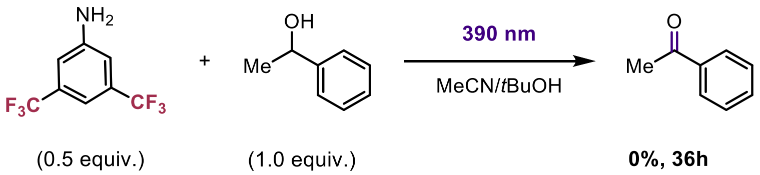

Figure S3. Byproduct Experiments: anaerobic oxidations using synthesized *N*-(3,5-bis(trifluoromethyl)phenyl)hydroxylamine[1] (A), synthesized 1-nitroso-4-(trifluoromethyl)benzene[2] (B), 1,2-bis(3,5-bis(trifluoromethyl)phenyl)diazene 1-oxide (C), and 3,5-bis(trifluoromethyl)aniline (D) as oxidants. Reactions were performed on a 0.5 mmol scale and the yield of acetephenone was determined by ^{1}H NMR using CH_2Br_2 as an internal standard.

Optimization of the reaction parameters for the oxidation of unactivated alcohols.

Table S3. Solvent screen for the oxidation of unactivated alcohols to ketones.

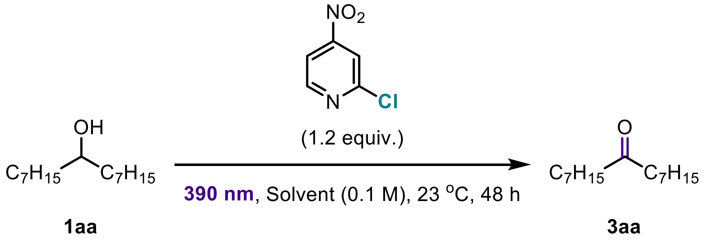

Entry	*Solvent*	***3aa***[a]
1	DCM	27%
2	$PhCF_3$	30%
3	Benzene	ND
4	Cyrene™	ND
5	Acetone	39%
6	EtOAc	ND
7	HFIP	ND
8	**MeCN**	**41%**

Reactions were performed on a 0.1 mmol scale. [a]Denotes ^1H NMR Yield using CH_2Br_2 as an internal standard.

*Solvents found to react with the photoexcited nitroarene: DMSO, DCE, 1,4, dioxane, MTBE, THF, DMA, Toluene, IPA, EtOAc, and EtOH.

Table S4. Additives screen for the oxidation of unactivated alcohols to ketones.

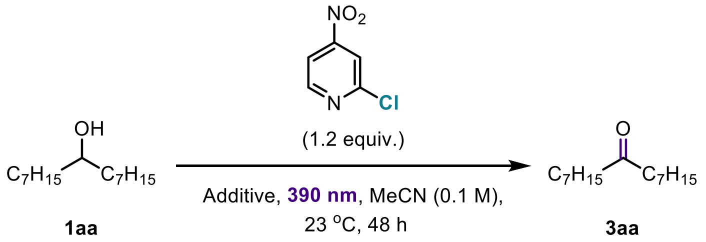

Entry	*Additive (1 equiv.)*	***3aa***[a]
1	-	41%
2	**LiOAc**	**58%**
3	KH_2PO_4	40%
4	Quinuclidine	3%
5	Aceclidine	27%
6	Quinuclidinol	3%
7	LiCl	42%
8	$LiBF_4$	33%
9	KBr	41%
10	KCl	34%
11	CsF	12%

Reactions were performed at 0.1 mmol scale. [a]Denotes ^1H NMR yield using CH_2Br_2 as an internal standard.

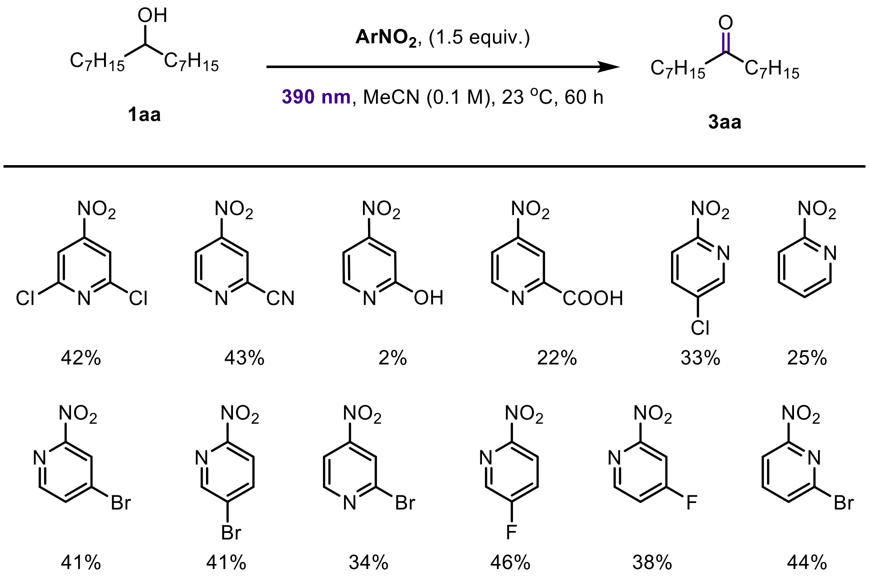

Figure S4. Nitroarene screen for oxidation of unactivated alcohols to ketones. Reactions performed on a 0.1 mmol scale. Yields were calculated using CH_2Br_2 as an internal standard.

Table S5. Solvent Screen for the oxidation of unactivated alcohols to ketones.

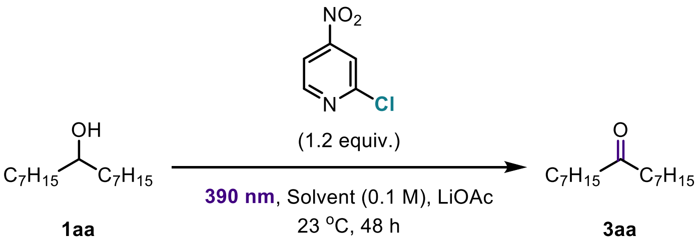

Entry	*Time*	*Solvent*	*LiOAc equiv.*	***3aa***[a]
1	48 h	MeCN	1	54%
2	48 h	MeCN	0.25	59%
3	60 h	MeCN	0.25	67%
4	60 h	MeCN/*t*BuOH (5:1)	-	65%
5	60 h	MeCN/*t*BuOH (5:1)	-	69%
6	60 h	MeCN/*t*BuOH (5:1)	0.25	77%
7	72 h	MeCN/*t*BuOH (5:1)	0.25	84%
8	**72 h**	**MeCN/*t*BuOH (5:1)**	**0.25**	**85%**[b]

Reactions were performed at 0.1 mmol scale. [a]Denotes ^1H NMR yield using CH_2Br_2 as an internal standard. [b]Using 3,5-bis(trifluoromethyl)nitrobenzene.

Optimization of the reaction parameters for the oxidation of amines.

Table S6. Nitropyridine equivalence screen for the oxidation of amines to imines.[a]

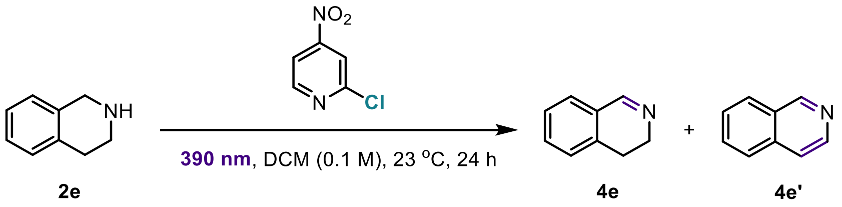

Equiv.	*4e*	*4e'*
0.25	47%	5%
0.5	49%	12%
1.0	41%	18%
1.5	32%	21%
2.0	28%	22%

Reactions were performed on a 0.1 mmol scale. ^{1}H NMR Yields calculated using CH_2Br_2 as an internal standard.

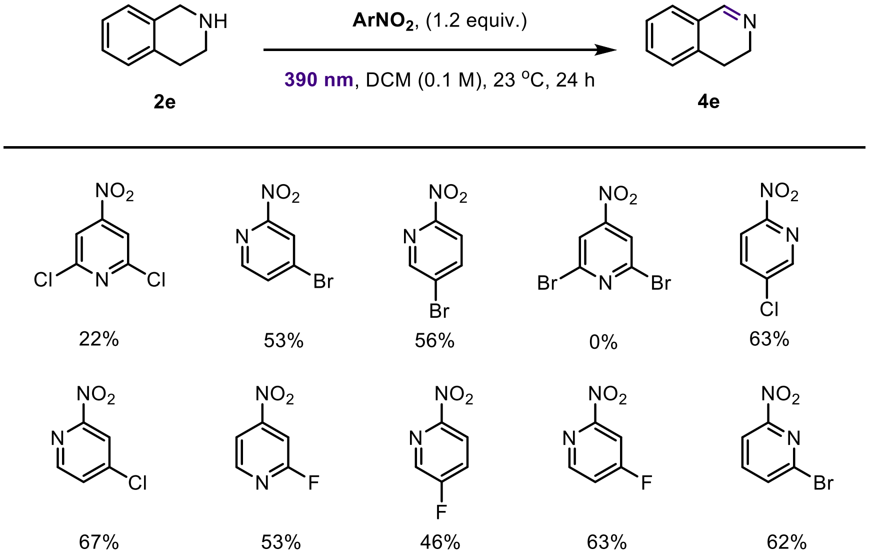

Figure S5. Nitropyridine screen for the oxidation of amines to imines. Reactions were performed on a 0.1 mmol scale. [a1]H NMR Yields calculated using CH_2Br_2 as an internal standard.

Table S7. Further wavelength, solvent, time, and additive screen for the oxidation of amines to imines.

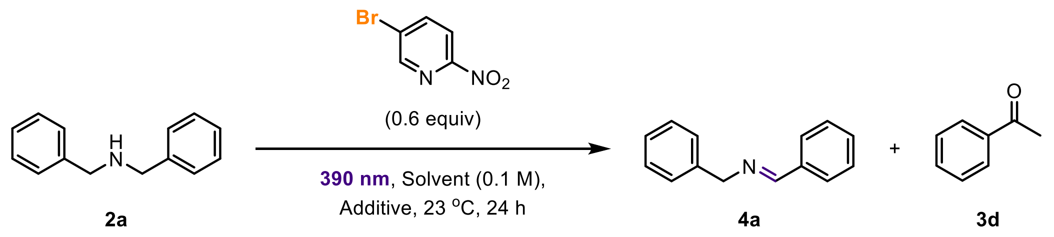

Entry	*Solvent*	*Wavelength*	*Time*	*Additive (0.1 equiv.)*	***4a***[a]	***3d***[a]
1	DCM	440 nm	72 h	-	84%[b]	16%
2	DCM	440 nm	72 h	-	57%	36%
3	DCM	440 nm	72 h	Pyr	80%[b]	16%
4	DCM	440 nm	72 h	Pyr	71%	22%
5	DCM/*t*BuOH (5:1)	440 nm	72 h	-	73%	24%
6	DCM/*t*BuOH (5:1)	440 nm	72 h	Pyr	58%	2%
7	DCM/*t*BuOH (5:1)	440 nm	72 h	tBuNH$_2$	88%	12%
8	$PhCF_3$	390 nm	24 h	tBuNH$_2$	90%	16%
9	**$PhCF_3$**	**390 nm**	**24 h**	**tBuNH$_2$**	**96%**[c]	**<1%**

Reactions were performed on a 0.1 mmol scale. [a]^{1}H NMR yields calculated using CH_2Br_2 as an internal standard. [b]Using 2-bromo-6-nitropyridine. [c]Using 1.0 equiv. of nitro.

Optimization of the reaction parameters for the oxidation of aldehydes.

Table S8. Optimization for the oxidation of benzylic aldehydes to carboxylic acids.

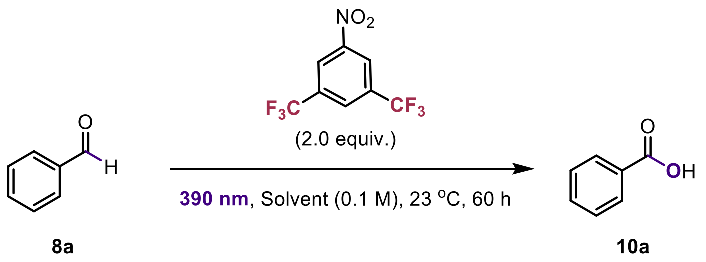

Entry	*Solvent*	*Conc.*	*Time*	*Conversion*[a]	*Yield*[b]
1	DCM	0.1 M	60 h	75%	70%
2	MeCN	0.1 M	60 h	70%	64%
3	MeCN	0.9 M	60 h	90%	80%
4	MeCN	1.0 M	60 h	90%	80%
5	Neat	-	12 h	70%	65%
6	**Neat**	**-**	**24 h**	**>99%**	**93%**

Reactions were performed on a 0.5 mmol scale. [a]Amount of **8a** converted. [b]^{1}H NMR Yields calculated using CH_2Br_2 as an internal standard.

Table S9. Optimization for the oxidation of aliphatic aldehydes to carboxylic acids.

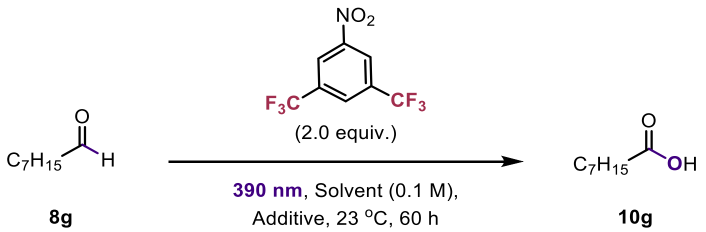

Entry	*Nitro (equiv.)*	*Solvent*	*Conc.*	*Additive*	*Time*	*Conversion*[a]	***10g***[b]
1	2	Neat	-	-	4 h	-	22%
2	2	Neat	-	-	12 h	-	27%
3	2	DCM	0.1 M	-	60 h	-	10%
4	2	MeCN	0.1 M	-	60 h	-	15%
5	2	MeCN	1.0 M	-	60 h	-	22%
6	2	MeCN	1.0 M	-	24 h	-	32%
7	1.5	MeCN	1.0 M	-	24 h	-	34%
8	1	MeCN	1.0 M	-	24 h	6%	40%
9	0.5	MeCN	1.0 M	-	24 h	30%	22%
10	1	MeCN	1.0 M	*t*BuOH (0.1 equiv.)	24 h	-	19%
11	1	MeCN	1.0 M	CH_3COOH (0.1 equiv.)	24 h	-	37%
12	1	MeCN	1.0 M	H_2O (0.1 equiv.)	24 h	-	40%
13	**1**	**MeCN**	**1.0 M**	**H_2O (1.0 equiv.)**	**24 h**	**-**	**55%**

Reactions were performed on a 0.5 mmol scale. [a]Amount of **8g** converted. [b]^{1}H NMR Yields calculated using CH_2Br_2 as an internal standard.

Optimization of the reaction parameters for the oxidation of imines.

Table S10. Optimization for the oxidation of imines to amides.

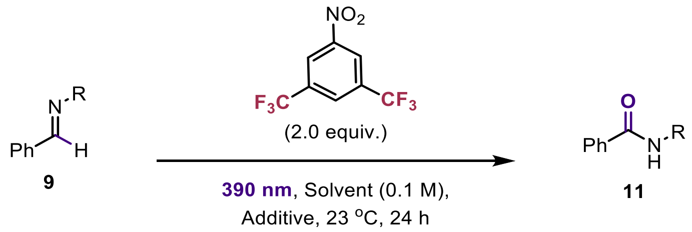

Entry	*R*	*Nitro (equiv.)*	*Solvent*	*Conc.*	*Time*	*Yield*[a]
1	*t*Bu	2	*t*BuOH	2 M	24 h	56%
2	*t*Bu	2	MeCN	2 M	24 h	64%
3	*t*Bu	2	MeCN	2 M	24 h	81%
4	*t*Bu	2	DCM/*t*BuOH (5:1)	0.4 M	24 h	56%
5	***t*Bu**	**2**	**Neat**	**-**	**24 h**	**91%**
6	*t*Bu	3	Neat	-	24 h	75%
7	**Cy**	**2**	DCM/*t*BuOH (5:1)	**0.4 M**	**24 h**	**96%**
8	Cy	1.5	DCM/*t*BuOH (5:1)	0.4 M	24 h	46%
9	Cy	1	DCM/*t*BuOH (5:1)	0.4 M	24 h	50%
10	Cy	0.5	DCM/*t*BuOH (5:1)	0.4 M	24 h	18%

Reactions were performed on a 0.5 mmol scale. [a] ^{1}H NMR Yields calculated using CH_2Br_2 as an internal standard.

General Procedures

General Procedure A. Standard Anaerobic Oxidations for Benzylic and Aliphatic Alcohols.

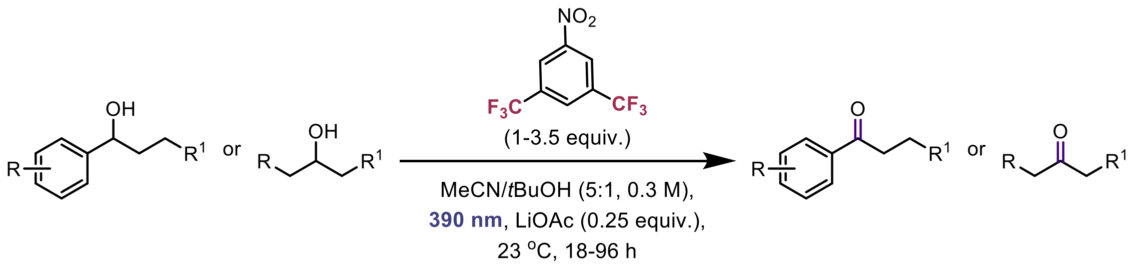

Oxidation for benzylic/activated alcohols to ketones:
Except when otherwise stated, in an oven-dried 0.5-dram vial equipped with a stir bar was added 3,5-bis(trifluoromethyl)nitrobenzene **(5)** (130 mg, 0.50 mmol, 1.0 equiv.), benzylic alcohol (0.50 mmol, 1.0 equiv.), lithium acetate (0.13 mmol, 0.25 equiv.) and 5:1 MeCN/*t*BuOH (2.3 mL, 0.30 M) under N_2 gas. The reaction vessel was left to stir at 1000 rpm and irradiated under 390 nm Kessil lamps with a cooling fan, for 24-96 h. Completion of the reaction was determined by GCMS analysis. After the reaction was complete, the solvent was removed under a stream of nitrogen. The crude product was purified by column chromatography (0 – 5% EtOAc/Hexane) to afford the ketone products.

Oxidation for unactivated alcohols to ketones:
Except when otherwise stated, in a glove box an oven dried 1.5-dram vial equipped with a stir bar was charged 3,5-bis(trifluoromethyl)nitrobenzene (**5**) (1 – 3 equiv.), alcohol (1.0 mmol, 1 equiv.), and where applicable, lithium acetate (0.40 mmol, 0.25 equiv.). Anhydrous, degassed 5:1 MeCN/*t*BuOH (1.0 mL, 0.36 M) was added. The reaction vessel was sealed and left to stir at 900 rpm, and the reaction was irradiated under 390 nm Kessil lamps with a cooling fan, for 24-96 h. Completion of the reaction was determined by GCMS analysis. The seal vessel was opened and K_2HPO_4 (2.5 mmol, 5.0 equiv.), urea (2.5 mmol, 5.0 equiv.) and formaldehyde (2.5 mmol, 5.0 equiv.) were added in the flask and left to stir for another 5-10 h or until the appearance of white solid. The contents of vial were diluted with EtOAc (20 mL) and washed with deionized H_2O (100 mL) and extracted using EtOAc (3 × 30 mL). Combined organic phases were dried with $NaHSO_4$ and concentrated in vacuo. The crude product was then purified by column chromatography (2.5 – 5% Acetone/ Hexane) to afford the ketone products.

Analysis of volatile ketone products:

Owing to the volatility of products **3ac**, **3ad**, **3ae**, and **3af**, these products were not isolated. The reaction efficiency was determined by crude ^{1}H NMR yield using CH_2Br_2 as an internal standard in CD_3CN. The corresponding ^{1}H NMR yield spectra for **3ac**, **3ad**, **3ae**, and **3af**, are provided in the NMR spectra section. To validate that the measured ^{1}H NMR yields were accurate, the crude mixtures of **3ac**, **3ad**, **3ae**, and **3af** were subjected to hydrazone derivatization (see the procedure below). It was found that the isolated yields of the hydrazone-derivatized products (**3ac'**, **3ad'**,

3ae', and **3af'**) were within the error of the crude ^{1}H NMR yield measurements. The ^{1}H NMR and ^{13}C NMR spectra of the hydrazone-derivatized products are provided in the characterization and NMR spectra sections.

Hydrazone derivatization for the isolation of volatile ketones:

Upon completion, the reaction mixture featuring 1.16 mmol of the volatile ketone (**3ac**, **3ad**, **3ae**, and **3af)** was mixed with H_2O (5 mL), HCl (600 µL, 6M aq.), then 2,4-dinitrophenylhydrazine (237 mg, 1.19 mmol, 1.03 equiv.) and stirred. The formation of the hydrazone was indicated by the appearance of reddish precipitates. Completion of the reaction was monitored by GCMS in 3 hours. The slurry was diluted with H_2O (50 mL), filtered, and stirred in 6 M HCl (20 ml) for additional 30 minutes. The filtered precipitate was washed with water, redissolved in dichloromethane, and dried with $NaHSO_4$. The crude product was then purified by column chromatography to afford the hydrazone products.

General Procedure B. Standard Anaerobic Oxidations for α-Substituted and Dibenzylic Alcohols.

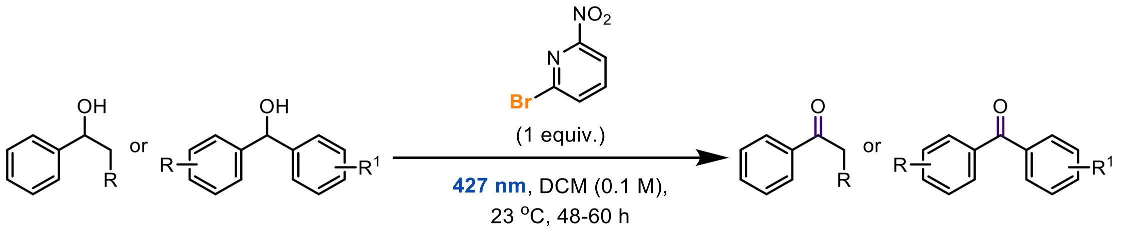

Except when otherwise stated, in an oven-dried 2-dram vial equipped with a stir bar was added 2-bromo-6-nitropyridine (**6**) (102 mg, 0.500 mmol, 1.0 equiv.), target alcohol (0.50 mmol, 1.0 equiv.), and DCM (5.0 mL, 0.10 M) under N_2 gas. The reaction vessel was left to stir at 1000 rpm and irradiated under 427 nm Kessil lamps with a cooling fan, for 48-60 h. Completion of the reaction was determined by GCMS analysis. After the reaction was completed, the solvent was removed under a stream of nitrogen. The crude product was purified by column chromatography (0 – 5% EtOAc/Hexane) to afford the ketone product.

General Procedure C. Standard Anaerobic Oxidations for amines to imines.

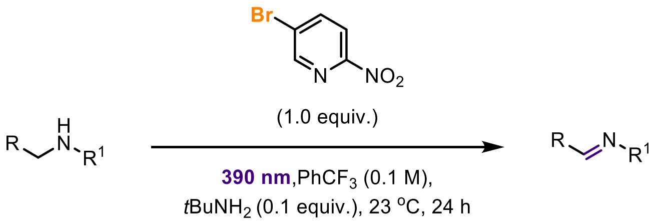

Except when otherwise stated, in an oven-dried 2-dram vial equipped with a stir bar was added 5-bromo-2-nitropyridine (**7**) (102 mg, 0.5 mmol, 1.0 equiv.), target amine (0.5 mmol, 1 equiv.), *t*$BuNH_2$ (0.05 mmol, 0.1 equiv.), and the vial was purged with N_2 flow for 15 min, followed by the addition of dry $PhCF_3$ (5 mL, 0.1 M). The reaction vessel was left to stir at 1000 rpm and

irradiated under 390 nm Kessil lamps with a cooling fan, for 24 h. Completion of the reaction was determined by GCMS analysis. After the reaction was completed, the solvent was removed under a stream of nitrogen. The crude product was purified by column chromatography (0 – 5% NEt_3/Hexane) to afford the imine product.

Analysis of hydrolytically unstable imine products:

Imine products **4c**, **4d**, **4e**, **4g**, **4h**, **and 4i** were found to be hydrolytically unstable. Numerous attempts to isolate the above compounds using NEt_3-treated silica gel, neutral and basic alumina, and Devisil failed to provide any appreciable products. The reaction efficiency for **4c**, **4d**, **4e**, and **4g** was determined by crude ^{1}H NMR yield using CH_2Br_2 as an internal standard in CD_3CN. The reaction efficiency for **4h** and **4i** was determined by crude ^{1}H NMR yield using CH_2Cl_2 as an internal standard in CD_3CN. The corresponding ^{1}H NMR yield spectra for **4c**, **4d**, **4e**, **4g**, **4h**, and **4i** are provided in the NMR spectra section. Unfortunately, attempts to derivatize the crude products were unsuccessful.

General Procedure D. Standard Anaerobic Oxidation for Aldehydes to Acids.

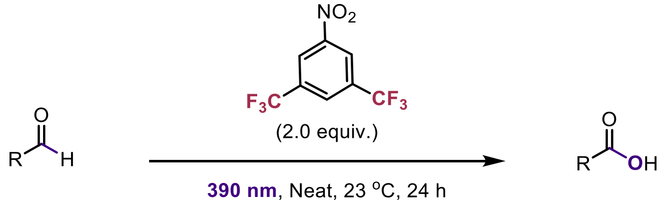

Oxidation for aromatic aldehydes to acids:

Except when otherwise stated, in an oven-dried 0.5-dram vial equipped with a stir bar was purged with N_2 flow for 15 min, 3,5-bis(trifluoromethyl)nitrobenzene (**5**) (170 µL, 1.00 mmol, 2.00 equiv.) and aldehyde (0.50 mmol, 1.0 equiv.) were added. The reaction vessel was left to stir at 1000 rpm and irradiated under 390 nm Kessil lamps with a cooling fan, for 24-36 h. Completion of the reaction was determined by GCMS analysis. After the reaction was completed, the mixture was concentrated down, and the crude product was then purified by column chromatography (0 – 50% EtOAc/Hexane) to afford the carboxylic acid product.

Oxidation for aliphatic aldehydes to acids:

Except when otherwise stated, in a flame- or oven-dried 0.5-dram vial equipped with a stir bar, 3,5-bis(trifluoromethyl)nitrobenzene (**5**) (84 µL, 0.50 mmol, 1.0 equiv.) and aldehyde (0.50 mmol, 1.0 equiv.) were added. The reaction vial was purged with N_2 flow for 15 min followed by the addition of H_2O (0.5 mmol, 1 equiv.), and CH_3CN (400 µL, 1.0 M). The vial was left to stir at 1000 rpm and irradiated under 390 nm Kessil lamps with a cooling fan, for 24-36 h. Completion of the reaction was determined by GCMS analysis. After the reaction was completed, the mixture was concentrated down, and the crude product was then purified by column chromatography (0 – 50% EtOAc/Hexane) to afford the carboxylic acid product.

General Procedure E. Standard Anaerobic Oxidation for imines to amide.

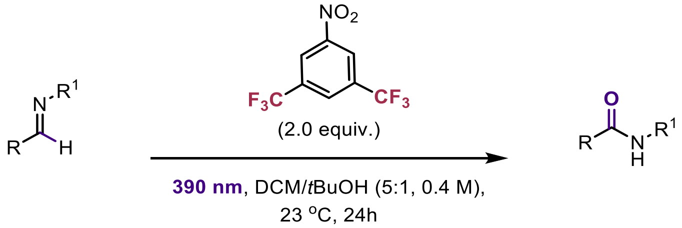

Except when otherwise stated, in a flame- or oven-dried 0.5-dram vial equipped with a stir bar was purged with N_2 flow for 15 min, 3,5-bis(trifluoromethyl)nitrobenzene (**5**) (170 µL, 0.50 mmol, 2.0 equiv.), aldehyde (0.5 mmol, 1.0 equiv.), and amine (0.50 mmol, 1.0 equiv.) were added, followed by the addition of 5:1 DCM/*t*BuOH (1.2 mL, 0.40 M). The reaction mixture was allowed to stir until imine formation was complete as determined by GCMS analysis. Then, the vessel was irradiated under a 390 nm Kessil lamp with a cooling fan, the stir rate was set to 1000 rpm, and the reaction was irradiated for 24-36 h. Completion of the reaction was determined by GCMS analysis. The crude product was then purified by column chromatography (0 – 50% EtOAc/Hexane) to afford the amide product.

General Procedure F. Continuous-Flow Conditions for the Photoinduced Anaerobic Oxidation Reactions.

The setup for the flow reactor used is as follows: 10 ft. of 0.03-inch diameter Fluorinated Ethylene Propylene (FEP) tubing was coiled around a 100 mL glass bottle (**Figure S6**). Two 390 nm Kessil lamps (34 W each) were placed approximately 3.0 cm from the coil of tubing. An electric fan was placed behind to prevent the tubing from overheating. A syringe pump was connected to control the flow rate of the reaction.

To a dry 1-dram vial was added nitroarene (0.75 - 2.0 equiv.) and substrate (1.0 equiv.). The vial was sealed with a septum cap and purged with nitrogen for 5 minutes. Then solvent (2.0 mL) was added, and the mixture was taken up in a 5.0 mL syringe which was attached to the FEP tubing. The flow rate was set using the syringe pump, the blue LEDs were switched on and the reaction flowed through (residence time of 3-5 h). A 2-dram vial was used to collect the reaction solution. After the given reaction time, the mixture was concentrated down and $CDCl_3$ (1.0 mL) and CH_2Br_2 (1.0 equiv.) was added as an internal ^{1}H NMR standard. The NMR yield of the oxidation product was recorded.

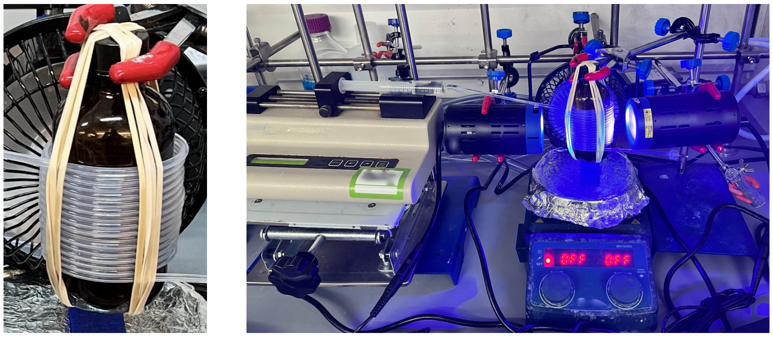

Figure S6. Images of the photochemical continuous-flow setup

Characterization of oxidized products

Characterization data of ketones

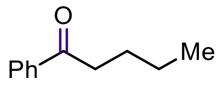

1-phenylpentan-1-one (3a)

Following General Procedure A, prepared from 0.50 mmol of 1-phenylpentan-1-ol (**1a**) and 3.0 equiv. of **5** in 24 h, the title compound was isolated via flash column chromatography (0 – 20% EtOAc/Hexane) as a clear oil (73 mg, 91% yield). All analytical data for **3a** was in accordance with literature data.[3]

^{1}H NMR (500 MHz, $CDCl_3$) (δ, ppm): 7.99 – 7.93 (m, 2H), 7.58 – 7.52 (m, 1H), 7.49 – 7.43 (m, 2H), 3.00 – 2.94 (m, 2H), 1.77 – 1.67 (m, 2H), 1.46 – 1.37 (m, 2H), 0.96 (t, J = 7.4 Hz, 3H).
^{13}C NMR (126 MHz, $CDCl_3$) (δ, ppm): 200.8, 137.3, 133.0, 128.7, 128.2, 38.5, 26.6, 22.6, 14.1.

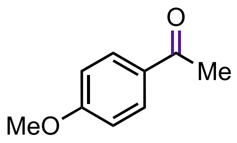

1-(4-methoxyphenyl)ethan-1-one (3b)

Following General Procedure A, prepared from 1.00 mmol of 1-(4-methoxyphenyl)ethan-1-ol (**1b**) in 24 h, the title compound was isolated via flash column chromatography (0 – 5% EtOAc/Hexane) as a yellow solid (141 mg, 98% yield). All analytical data for **3b** was in accordance with literature data.[4]

^{1}H NMR (400 MHz, $CDCl_3$) (δ, ppm): 7.94 (d, J = 8.9 Hz, 2H), 6.93 (d, J = 9.0 Hz, 2H), 3.87 (s, 3H), 2.56 (s, 3H).
^{13}C NMR (101 MHz, $CDCl_3$) (δ, ppm): 196.9, 163.6, 130.7, 130.5, 113.8, 55.6, 26.5.

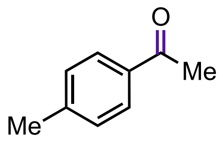

1-(*p*-tolyl)ethan-1-one (3c)

Following General Procedure A, prepared from 0.50 mmol of 1-(*p*-tolyl)ethan-1-ol (**1c**) and 3.0 equiv. of **5** in 36 h. The title compound was isolated via flash column chromatography (0 – 5% EtOAc/Hexane) as a yellow oil (56 mg, 82% yield). All analytical data for **3c** was in accordance with literature data.[5]

^{1}H NMR (500 MHz, CD_3CN) (δ, ppm): 7.86 (d, J = 8.1 Hz, 2H), 7.31 (d, J = 7.9 Hz, 2H), 2.53 (s, 3H), 2.40 (s, 3H).
^{13}C NMR (126 MHz, CD_3CN) (δ, ppm): 198.6, 144.9, 135.8, 130.2, 129.3, 26.9, 21.6.

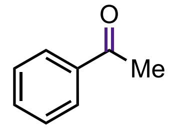

Acetophenone (3d)
Following General Procedure A, prepared from 1.0 mmol of 1-phenylethan-1-ol (**1d**) in 36 h. The title compound was isolated via flash column chromatography (0 – 5% EtOAc/Hexane) as a yellow oil (96 mg, 80% yield). All analytical data for **3d** was in accordance with literature data.[4]

^{1}H NMR (500 MHz, $CDCl_3$) (δ, ppm): 7.96 (dd, *J* = 8.4, 1.3 Hz, 2H), 7.59 – 7.54 (m, 1H), 7.47 (dd, *J* = 8.4, 7.0 Hz, 2H), 2.61 (s, 3H).
^{13}C NMR (126 MHz, $CDCl_3$) (δ, ppm): 198.3, 137.3, 133.3, 128.72, 128.71, 128.5, 128.5, 128.5, 128.4, 26.7.

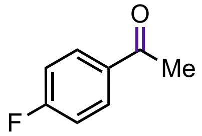

1-(4-Fluorophenyl)ethan-1-one (3e)
Following General Procedure A, prepared from 1.00 mmol of 1-(4-fluorophenyl)ethan-1-ol (**1e**) and 3.00 equiv. of **5** in 48 h. The title compound was isolated via flash column chromatography (0 – 5% EtOAc/Hexane) as a yellow oil (134 mg, 97% yield). All analytical data for **3e** was in accordance with literature data.[6]

^{1}H NMR (400 MHz, $CDCl_3$) (δ, ppm): 8.02 – 7.94 (m, 2H), 7.17 – 7.09 (m, 2H), 2.59 (s, 3H).
^{13}C NMR (101 MHz, $CDCl_3$) (δ, ppm): 196.5, 165.8 (d, *J* = 255.5 Hz), 133.6 (d, *J* = 3.0 Hz), 131.0 (d, *J* = 9.0 Hz), 115.7 (d, *J* = 22.2 Hz), 26.6.
^{19}F NMR (377 MHz, $CDCl_3$) (δ, ppm): -63.13.

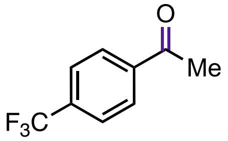

1-(4-(Trifluoromethyl)phenyl)ethan-1-one (3f)
Following General Procedure A, prepared from 0.50 mmol of 1-(4-(trifluoromethyl)phenyl)ethan-1-ol (**1f**) and 2.0 equiv. of **5** in 36 h. The title compound was isolated via flash column chromatography (0 – 5% EtOAc/Hexane) as a yellow oil (65 mg, 70% yield). All analytical data for **3f** was in accordance with literature data.[4]

^{1}H NMR (400 MHz, $CDCl_3$) (δ, ppm): 8.06 (d, *J* = 8.2 Hz, 2H), 7.74 (d, *J* = 8.1 Hz, 2H), 2.65 (s, 3H).
^{13}C NMR (126 MHz, $CDCl_3$) (δ, ppm): 197.1, 139.8, 134.5 (q, *J* = *32*.8 Hz), 128.8, 125.8 (q, *J* = 3.8 Hz), 123.7 (q, *J* = 273.4 Hz), 27.0.

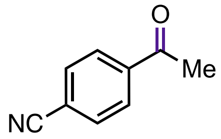

4-Acetylbenzonitrile (3g)

Following General Procedure A, prepared from 0.50 mmol of synthesized 4-(1-hydroxyethyl)benzonitrile (**1g**)[7a] in 36 h. The title compound was isolated via flash column chromatography (0 – 20% EtOAc/Hexane) as a white solid (70 mg, 98% yield). All analytical data for **3g** was in accordance with literature data.[7b]

^{1}H NMR (500 MHz, $CDCl_3$) (δ, ppm): 8.04 (d, J = 8.4 Hz, 2H), 7.78 (d, J = 8.4 Hz, 2H), 2.65 (s, 3H).

^{13}C NMR (126 MHz, $CDCl_3$) (δ, ppm): 196.7, 140.1, 132.7, 128.8, 118.1, 116.6, 26.9.

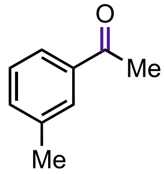

1-(*m*-Tolyl)ethan-1-one (3h)

Following General Procedure A, prepared from 0.50 mmol of 1-(*m*-tolyl)ethan-1-ol (**1h**) and 3.0 equiv. of **5** in 36 h, the title compound was isolated via flash column chromatography (0 – 5% EtOAc/Hexane) as a yellow oil (48 mg, 70% yield). All analytical data for **3h** was in accordance with literature data.[8]

^{1}H NMR (500 MHz, $CDCl_3$) (δ, ppm): 7.79 – 7.73 (m, 2H), 7.40 – 7.33 (m, 2H), 2.59 (s, 3H), 2.41 (s, 3H).

^{13}C NMR (126 MHz, $CDCl_3$) (δ, ppm): 198.6, 138.5, 137.3, 134.0, 128.9, 128.6, 125.7, 26.8, 21.5.

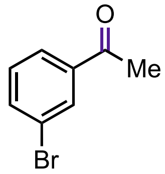

1-(3-Bromophenyl)ethan-1-one (3i)

Following General Procedure A, prepared from 0.50 mmol of 1-(3-bromophenyl)ethan-1-ol (**1i**) and 2.0 equiv. of **5** in 36 h. The title compound was isolated via flash column chromatography (0 – 5% EtOAc/Hexane) as a yellow oil (34 mg, 35% yield). All analytical data for **3i** was in accordance with literature data.[9]

^{1}H NMR (500 MHz, $CDCl_3$) (δ, ppm): 8.08 (q, J = 1.7 Hz, 1H), 7.87 (dq, J = 7.7, 1.5 Hz, 1H), 7.71 – 7.66 (m, 1H), 7.34 (td, J = 7.8, 1.8 Hz, 1H), 2.59 (s, 3H).

^{13}C NMR (126 MHz, $CDCl_3$) (δ, ppm): 196.8, 138.9, 136.1, 131.5, 130.3, 127.0, 123.1, 26.8.

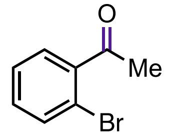

1-(2-Bromophenyl)ethan-1-one (3k)
Following General Procedure A, prepared from 0.50 mmol of 1-(2-bromophenyl)ethan-1-ol (**1k**) and 3.0 equiv. of **5** in 24 h, the title compound was isolated via flash chromatography (0 – 5 % EtOAc/Hexane) as a yellow oil (46 mg, 46% yield). All analytical data for **3k** was in accordance with the literature data.[10]

^{1}H NMR (500 MHz, $CDCl_3$) (δ, ppm): 7.62 (dd, J = 8.1, 1.1 Hz, 1H), 7.47 (dd, J = 7.6, 1.8 Hz, 1H), 7.37 (td, J = 7.5, 1.2 Hz, 1H), 7.30 (td, J = 7.7, 1.8 Hz, 1H), 2.64 (s, 3H).
^{13}C NMR (126 MHz, $CDCl_3$) (δ, ppm): 201.6, 141.6, 134.0, 132.0, 129.1, 127.6, 119.1, 30.5.

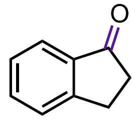

2,3-Dihydro-1*H*-inden-1-one (3l)
Following General Procedure A, prepared from 0.50 mmol of 1,2,3,4-tetrahydronaphthalen-1-ol (**1l**) in 36 h, the title compound was isolated via flash chromatography (0 – 5% EtOAc/Hexane) as a brown oil (67 mg, 51% yield). All analytical data for **3l** was in accordance with the literature data.[11]

^{1}H NMR (400 MHz, $CDCl_3$) (δ, ppm): 7.75 (dt, J = 7.7, 1.0 Hz, 1H), 7.58 (td, J = 7.4, 1.3 Hz, 1H), 7.47 (dp, J = 7.7, 1.0 Hz, 1H), 7.36 (ddt, J = 8.0, 7.2, 0.9 Hz, 1H), 3.18 – 3.10 (m, 2H), 2.72 – 2.64 (m, 2H).
^{13}C NMR (101 MHz, $CDCl_3$) (δ, ppm): 207.2, 155.3, 137.2, 134.7, 127.4, 126.8, 123.8, 36.3, 25.9.

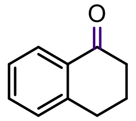

3,4-Dihydronaphthalen-1(2*H*)-one (3m)
Following General Procedure A, prepared from 0.50 mmol of 1,2,3,4-tetrahydronaphthalen-1-ol (**1m**) in 36 h, the title compound was isolated via flash chromatography (0 – 5% EtOAc/Hexane) as a colorless oil (70 mg, 48% yield). All analytical data for **3m** was in accordance with the literature data.[11]

^{1}H NMR (500 MHz, $CDCl_3$) (δ, ppm): 8.03 (dd, J = 7.9, 1.5 Hz, 1H), 7.47 (td, J = 7.5, 1.5 Hz, 1H), 7.31 (t, J = 7.5 Hz, 1H), 7.25 (d, J = 7.9 Hz, 1H), 2.97 (t, J = 6.1 Hz, 2H), 2.66 (dd, J = 7.3, 5.7 Hz, 2H), 2.15 (q, J = 6.3 Hz, 2H).
^{13}C NMR (126 MHz, $CDCl_3$) (δ, ppm): 198.5, 144.6, 133.5, 132.8, 128.9, 127.3, 126.8, 39.3, 29.9, 23.4.

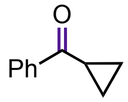

Cyclopropyl(phenyl)methanone (3n)
Following General Procedure A, prepared from 1.00 mmol of cyclopropyl(phenyl)methanol (**1n**) in 36 h, the title compound was isolated via flash column chromatography (0 – 5% EtOAc/Hexane) as a yellow oil (142 mg, 98% yield). All analytical data for **3n** was in accordance with literature data.[12]

^{1}H NMR (400 MHz, $CDCl_3$) (δ, ppm): 8.05 – 7.96 (m, 2H), 7.61 – 7.53 (m, 1H), 7.52 – 7.42 (m, 2H), 2.68 (tt, *J* = 7.8, 4.6 Hz, 1H), 1.28 – 1.22 (m, 2H), 1.08 – 1.01 (m, 2H).
^{13}C NMR (101 MHz, $CDCl_3$) (δ, ppm): 200.8, 138.2, 132.9, 128.6, 128.2, 17.3, 11.8.

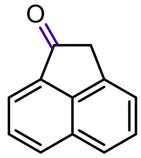

Acenaphthylen-1(2*H*)-one (3o)
Following General Procedure B, prepared from 0.50 mmol of 1,2-dihydroacenaphthylen-1-ol (**1o**) in 36 h, the title compound was isolated via flash column chromatography (0 – 5% EtOAc/Hexane) as a yellow solid (67 mg, 86% yield). All analytical data for **3o** was in accordance with literature data.[13]

^{1}H NMR (400 MHz, $CDCl_3$) (δ, ppm): 8.10 (dd, *J* = 8.1, 0.7 Hz, 1H), 7.97 (dd, *J* = 7.1, 0.8 Hz, 1H), 7.83 (dd, *J* = 8.5, 0.8 Hz, 1H), 7.72 (dd, *J* = 8.1, 7.0 Hz, 1H), 7.60 (dd, *J* = 8.4, 6.8 Hz, 1H), 7.47 (dd, *J* = 6.9, 1.0 Hz, 1H), 3.83 (s, 2H).
^{13}C NMR (126 MHz, $CDCl_3$) (δ, ppm): 203.0, 143.0, 135.1, 134.7, 131.5, 131.0, 128.5, 128.1, 124.0, 121.5, 121.1, 42.1.

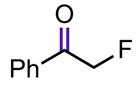

2-Fluoro-1-phenylethan-1-one (3p)
Following General Procedure B, prepared from 0.20 mmol of synthesized 2-fluoro-1-phenylethan-1-ol (**1p**)[14]at 390 nm in 96 h, the title compound was isolated via flash column chromatography (0 – 25% DCM/Hexane) as a colorless liquid (27 mg, 93% yield). All analytical data for **3p** was in accordance with literature data.[15]

^{1}H NMR (400 MHz, $CDCl_3$) (δ, ppm): 7.95 – 7.87 (m, 2H), 7.63 (ddt, *J* = 7.9, 6.9, 1.3 Hz, 1H), 7.57 – 7.45 (m, 2H), 5.53 (d, *J* = 46.9 Hz, 2H).
^{13}C NMR (101 MHz, $CDCl_3$) (δ, ppm): 193.6 (d, *J* = 15.2 Hz), 134.29, 133.89, 129.09, 128.05, 128.02, 83.7 (d, *J* = 183.8 Hz).

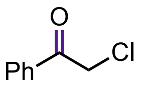

2-Chloro-1-phenylethan-1-one (3q)
Following General Procedure B, prepared from 0.50 mmol of 2-chloro-1-phenylethan-1-ol (**1q**) and 2-bromo-4-nitropyridine in 96 h, the title compound was isolated via flash column chromatography (0 – 25% DCM/Hexane) as a white solid (65 mg, 84% yield). All analytical data for **3q** was in accordance with literature data.[4]

^{1}H NMR (400 MHz, CDCl3) (δ, ppm): 8.11 – 7.90 (m, 2H), 7.75 – 7.57 (m, 1H), 7.55 – 7.38 (m, 2H), 4.72 (s, 2H).
^{13}C NMR (101 MHz, CDCl3) (δ, ppm): 191.1, 134.3, 134.0, 128.9, 128.6, 46.0.

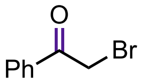

2-Bromo-1-phenylethan-1-one (3r)
Following General Procedure B, prepared from 0.50 mmol of 2-bromo-1-phenylethan-1-ol (**1r**) and 2-bromo-4-nitropyridine in 48 h, the title compound was isolated via flash column chromatography (0 – 25% DCM/Hexane) as a colorless liquid (78 mg, 78% yield). All analytical data for **3r** was in accordance with literature data.[4]

^{1}H NMR (400 MHz, $CDCl_3$) (δ, ppm): 8.02 – 7.96 (m, 2H), 7.67 – 7.57 (m, 1H), 7.53 – 7.45 (m, 2H), 4.46 (s, 2H).
^{13}C NMR (101 MHz, $CDCl_3$) (δ, ppm): 191.4, 134.11, 134.09, 129.1, 129.0, 31.1.

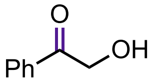

2-Hydroxy-1-phenylethan-1-one (3s)
Following General Procedure A, prepared from 0.50 mmol of 1-phenylethane-1,2-dioland (**1s**) and 1 equiv. of **5** in MeCN/H_2O (1:1, 0.30 M), in 48 h, the title compound was isolated via flash column chromatography (0 – 25% DCM/Hexane) as a white solid (32 mg, 47% yield). All analytical data for **3s** was in accordance with literature data.[4]

^{1}H NMR (400 MHz, $CDCl_3$) (δ, ppm): 7.96 – 7.89 (m, 2H), 7.70 – 7.59 (m, 1H), 7.54 – 7.46 (m, 2H), 4.89 (s, 2H), 3.50 (br, 1H).
^{13}C NMR (101 MHz, $CDCl_3$) (δ, ppm): 198.5, 134.5, 133.5, 129.1, 127.9, 65.6.

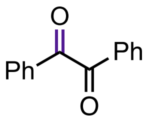

Benzil (3t)
Following General Procedure A, prepared from 0.50 mmol of 2-hydroxy-1,2-diphenylethan-1-one (**1t**) and 1.0 equiv. of **5** in 24h, the title compound was isolated via flash chromatography (0 – 5 %

EtOAc/Hexane) as a yellow solid (67 mg, 64% yield). All analytical data for **3t** was in accordance with the literature data.[16]

^{1}H NMR (400 MHz, $CDCl_3$) (δ, ppm): 8.02 – 7.94 (m, 4H), 7.74 – 7.60 (m, 2H), 7.57 – 7.45 (m, 4H).
^{13}C NMR (101 MHz, $CDCl_3$) (δ, ppm): 194.7, 135.0, 133.2, 130.1, 129.2.

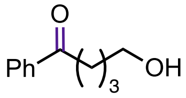

5-Hydroxy-1-phenylpentan-1-one (3u)
Following General Procedure A, prepared from 0.50 mmol of 1-phenylpentane-1,5-diol (**1u**) and and 1 equiv. of **5** in MeCN/H_2O (1:1, 0.30 M), in 36 h, the title compound was isolated via flash column chromatography (0 – 5% Acetone/DCM) as a colorless oil (37 mg, 42% yield). All analytical data for **3u** was in accordance with literature data.[17]

^{1}H NMR (400 MHz, $CDCl_3$) (δ, ppm): 8.05 – 7.91 (m, 2H), 7.59 – 7.53 (m, 1H), 7.50 – 7.41 (m, 2H), 3.68 (t, J = 6.3 Hz, 2H), 3.03 (t, J = 7.1 Hz, 2H), 1.90 – 1.81 (m, 2H), 1.77 – 1.54 (m, 2H).
^{13}C NMR (101 MHz, $CDCl_3$) (δ, ppm): 200.5, 137.1, 133.2, 128.8, 128.23, 128.20, 62.6, 38.3, 32.4, 20.3.

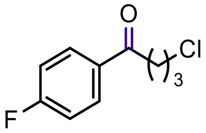

4-Chloro-1-(4-fluorophenyl)butan-1-one (3v)
Following General Procedure A, prepared from 0.500 mmol of synthesized 4-chloro-1-(4-fluorophenyl)butan-1-one (**1v**)[18a] and 3.00 equiv. of **5** in 24 h, the title compound was isolated via flash chromatography (0 – 5% EtOAc/Hexane) as a yellow oil (120 mg, 61% yield). All analytical data for **3v** was in accordance with the literature data.[18b]

^{1}H NMR (400 MHz, $CDCl_3$) (δ, ppm): 8.05 – 7.96 (m, 2H), 7.14 (t, J = 8.6 Hz, 2H), 3.68 (t, J = 6.2 Hz, 2H), 3.15 (t, J = 6.9 Hz, 2H), 2.22 (p, J = 6.7 Hz, 2H).
^{13}C NMR (101 MHz, $CDCl_3$) (δ, ppm): 197.5, 166.0 (d, J = 254.9 Hz), 133.3 (d, J = 3.0 Hz), 130.8 (d, J = 9.3 Hz), 115.9 (d, J = 21.9 Hz), 44.8, 35.3, 26.8.

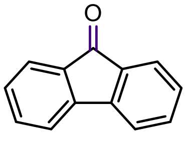

9*H*-Fluoren-9-one (3w)
Following General Procedure B, prepared from 0.50 mmol of 9*H*-fluoren-9-ol (**1w**) and in 36 h, the title compound was isolated via flash column chromatography (0 – 5% EtOAc/Hexane) as a yellow solid (67 mg, 75% yield). All analytical data for **3w** was in accordance with literature data.[13]

^{1}H NMR (500 MHz, $CDCl_3$) (δ, ppm): 7.67 (dd, J = 7.4, 1.0 Hz, 1H), 7.53 (d, J = 7.4 Hz, 1H), 7.49 (td, J = 7.4, 1.2 Hz, 1H), 7.30 (td, J = 7.3, 1.2 Hz, 1H).
^{13}C NMR (126 MHz, $CDCl_3$) (δ, ppm): 194.1, 144.6, 134.8, 134.3, 129.2, 124.6, 120.5.

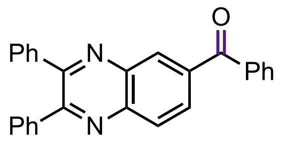

(2,3-Diphenylquinoxalin-6-yl)(phenyl)methanone (3x)
Following General Procedure B, prepared from 0.17 mmol of synthesized (2,3-diphenylquinoxalin-6-yl)(phenyl)methanol (**1x**)[18a, 19a] in 36h, the title compound was isolated via flash column chromatography (0 – 5% EtOAc/Hexane) as a white solid (55 mg, 85% yield). All analytical data for **3x** was in accordance with literature data.[19b]

^{1}H NMR (400 MHz, $CDCl_3$) (δ, ppm): 8.54 (dd, J = 1.7, 0.8 Hz, 1H), 8.32 – 8.25 (m, 2H), 7.93 – 7.89 (m, 2H), 7.67 – 7.62 (m, 1H), 7.59 – 7.50 (m, 6H), 7.43 – 7.31 (m, 6H).
^{13}C NMR (126 MHz, $CDCl_3$) (δ, ppm): 195.8, 155.2, 154.6, 143.0, 140.2, 138.7, 138.6, 138.3, 137.2, 132.8, 132.5, 130.2, 129.9, 129.8, 129.7, 129.7, 129.3, 129.2, 128.6, 128.4, 128.3.

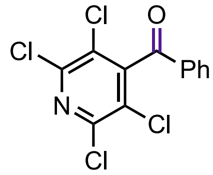

(Perchloropyridin-4-yl)(phenyl)methanone (3y)
Following General Procedure A, prepared from 0.14 mmol of perchloropyridin-4-yl)(phenyl)methanol (**1y**) and 3 eq of nitropyridine **6**, the title compound was isolated via flash chromatography (0 – 5% EtOAc/Hexane) as a white solid (5.0 mg, 11% yield). All analytical data for **3y** was in accordance with the literature data.[20]

^{1}H NMR (600 MHz, $CDCl_3$) (δ, ppm): 7.80 (dd, J = 1.3 Hz, 2H), 7.74-7.68 (m, 1H), 7.55 (dd, J = 8.4, 7.4 Hz, 2H).
^{13}C NMR (126 MHz, $CDCl_3$) (δ, ppm): 188.8, 149.4, 147.0, 135.6, 133.5, 129.7, 129.6, 126.5.
HRMS (ESI-TOF): m/z calculated for $C_{12}H_5Cl_4NO$ [M]+ = 318.9125, found 318.9141.

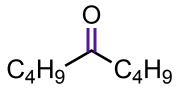

Nonan-5-one (3z)
Following General Procedure A, prepared from 0.500 mmol of nonan-5-ol (**1z**) and 1.10 equiv. of **5** in 96 h, the title compound was isolated via flash chromatography (1 – 5% Acetone/Hexane) as a clear oil (61 mg, 86% yield). All analytical data for **3z** was in accordance with literature data.[21]

^{1}H NMR (500 MHz, $CDCl_3$) (δ, ppm): 2.37 (t, J = 7.5 Hz, 4H), 1.60 – 1.47 (m, 4H), 1.36 – 1.20 (m, 4H), 0.88 (t, J = 7.4 Hz, 6H).
^{13}C NMR (126 MHz, $CDCl_3$) (δ, ppm): 212.0, 42.9, 26.4, 22.8, 14.2.

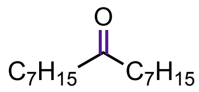

Pentadecan-8-one (3aa)

Following General Procedure A, prepared from 0.50 mmol of 8-pentadecanol (**1aa**) and 3.5 equiv. of **5** in 96 h, the title compound was isolated via flash chromatography (1 – 5% Acetone/Hexane) as a tan oil (45 mg, 78% yield). All analytical data for **3aa** was in accordance with literature data.[22a, 22b]

^{1}H NMR (500 MHz, $CDCl_3$) (δ, ppm): 2.38 (t, J = 7.5 Hz, 4H), 1.56 (dd, J = 13.1, 5.9 Hz, 4H), 1.27 (s, 16H), 0.87 (t, J = 6.9 Hz, 6H).

^{13}C NMR (126 MHz, $CDCl_3$) (δ, ppm): 211.7, 42.7, 31.6, 29.1, 29.0, 23.8, 22.5, 14.0.

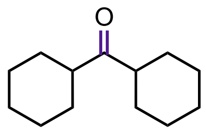

Dicyclohexylmethanone (3ab)

Following General Procedure A, prepared from 0.48 mmol of dicyclohexylmethanol (**1ab**) and 1.1 equiv. of **5**, the title compound was isolated via flash chromatography (1 – 5% Acetone/Hexane) as a pale oil (52 mg, 56% yield). All analytical data for **3ab** was in accordance with literature data.[23]

^{1}H NMR (500 MHz, $CDCl_3$) (δ, ppm): 2.48 (ddd, J = 11.0, 8.2, 2.8 Hz, 2H), 1.81 – 1.73 (m, 8H), 1.70 – 1.63 (m, 2H), 1.39 – 1.13 (m, 10H).

^{13}C NMR (126 MHz, $CDCl_3$) (δ, ppm): 217.6, 49.6, 29.0, 26.3, 26.2.

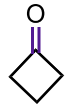

Cyclobutanone (3ac)

Following General Procedure A, prepared from 0.49 mmol of cyclobutanol (**1ac**) and 3.5 equiv. of **5** in 96 h. ^{1}H NMR yield determined using CH_2Br_2 as an internal standard (>99% yield). All analytical data for **3ac** was in accordance with literature data.[24]

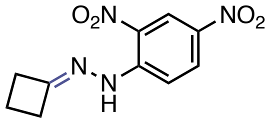

1-cyclobutylidene-2-(2,4-dinitrophenyl)hydrazine (3ac')

Following General Procedure A, prepared from 1.16 mmol of cyclobutanol (**1ac**) and 2.5 equiv. of **5**, the title compound **3ac** was subjected to hydrazone derivatization and isolated as 2,4-dinitropheylhydrazone of cyclopentanone **3ac'**. The title compound was purified by silica gel chromatography (Pentane:EtOAc 9:1) to give a reddish crystalline solid (270 mg) in 93% yield. All analytical data of **3ac'** was in accordance with the reported literature.[25]

^{1}H NMR (500 MHz, $CDCl_3$) δ 10.72 (s, 1H), 9.12 (d, J = 2.6 Hz, 1H), 8.29 (dd, J = 9.6, 2.6 Hz, 1H), 7.87 (d, J = 9.6 Hz, 1H), 3.18 – 3.03 (m, 4H), 2.19 (p, J = 8.1 Hz, 2H).

^{13}C NMR (126 MHz, $CDCl_3$) δ 161.2, 144.7, 137.5, 129.9, 128.6, 123.5, 115.9, 33.8, 31.7, 13.7.

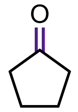

Cyclopentanone (3ad)

Following General Procedure A, prepared from 0.50 mmol of cyclopentanol (**1ad**) and 3.5 equiv. of **5** in 96 h. ^{1}H NMR yield determined using CH_2Br_2 as an internal standard (90% yield). All analytical data for **3ad** was in accordance with literature data.[26]

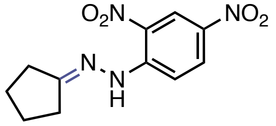

1-cyclopentylidene-2-(2,4-dinitrophenyl)hydrazine(3ad')

Following General Procedure A, prepared from 1.16 mmol of cyclopentanol (**1ad**) and 2.5 equiv. of **5**, the title compound **3ad** was subjected to hydrazone derivatization and isolated as 2,4-dinitropheylhydrazone of cyclopentanone **3ad'**. The title compound was purified by silica gel chromatography (Pentane:EtOAc 9:1.5) to give a yellow crystalline solid (265 mg) in 86% yield. All analytical data of **3ad'** was in accordance with the reported literature.[27]

^{1}H NMR (500 MHz, $CDCl_3$) δ 10.81 (s, 1H), 9.12 (d, J = 2.6 Hz, 1H), 8.28 (dd, J = 9.7, 2.7 Hz, 1H), 7.92 (d, J = 9.6 Hz,1H), 2.58 (t, J = 7.2 Hz, 2H), 2.47 (t, J = 7.4 Hz, 2H), 1.99 (p, J = 7.0 Hz, 2H), 1.88 (p, J = 7.0 Hz, 3H).

^{13}C NMR (126 MHz, $CDCl_3$) δ 168.2, 144.9, 137.4, 129.8, 128.6, 123.5, 116.1, 33.5, 28.0, 24.8, 24.7.

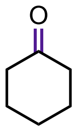

Cyclohexanone (3ae)

Following General Procedure A, prepared from 0.50 mmol of cyclohexanol (**1ae**) and 3.5 equiv. of **5** in 96 h. ^{1}H NMR yield determined using CH_2Br_2 as an internal standard (>99% yield). All analytical data for **3ae** was in accordance with literature data.[26]

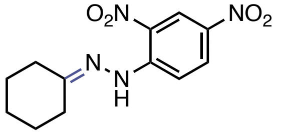

1-cyclohexylidene-2-(2,4-dinitrophenyl)hydrazine(3ae')

Following General Procedure A, prepared from 1.16 mmol of cyclohexanol (**1ae**) and 2.5 equiv. of **5**, the title compound **3ae** was subjected to hydrazone derivatization and isolated as 2,4-

dinitropheylhydrazone of cyclopentanone **3ae'**. The title compound was purified by silica gel chromatography (Pentane:EtOAc 9:1.5) to give a yellow crystalline solid (295 mg) in 91% yield. All analytical data for 2,4-dinitrophenylhydrazone of **3ae'** was in accordance with the reported literature.[28]

^{1}H NMR (500 MHz, $CDCl_3$) δ 11.20 (s, 1H), 9.13 (d, *J* = 2.6 Hz, 1H), 8.29 (dd, *J* = 9.6, 2.6 Hz, 1H), 7.97 (d, *J* = 9.6 Hz, 1H), 2.47 (q, *J* = 6.2 Hz, 4H), 1.79 (dt, *J* = 11.5, 6.2 Hz, 4H), 1.72 (td, *J* = 6.6, 3.3 Hz, 2H) of **5** in 96 h.

^{13}C NMR (126 MHz, $CDCl_3$) δ 161.5, 145.5, 137.6, 130.1, 128.9, 123.8, 116.4, 35.7, 27.3, 27.1, 26.1, 25.6.

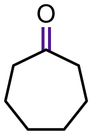

Cycloheptanone (3af)
Following General Procedure A, prepared from 0.50 mmol of cycloheptanol (**1af**) and 3.5 equiv. of **5** in 96 h. ^{1}H NMR yield determined using CH_2Br_2 as an internal standard (>99% yield). All analytical data for **3ae** was in accordance with literature data.[29]

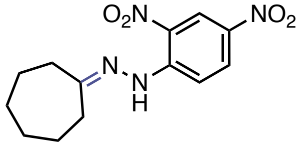

1-cycloheptylidene-2-(2,4-dinitrophenyl)hydrazine(3af')
Following General Procedure A, prepared from 1.16 mmol of cycloheptanol (**1af**) and 2.5 equiv. of **5**, the title compound **3af** was subjected to hydrazone derivatization and isolated as 2,4-dinitropheylhydrazone of cyclopentanone **3af'**. The title compound was recrystallized from pentane to give a bright yellow solid (310 mg) in 91% yield. All analytical data for **3af'** was in accordance with the reported literature.[30]

^{1}H NMR (500 MHz, $CDCl_3$) δ 11.05 (s, 1H), 9.13 (q, *J* = 2.8, 2.0 Hz, 1H), 8.29 (dd, *J* = 9.6, 2.6 Hz, 1H), 7.99 (d, *J* = 9.7 Hz, 1H), 2.66 – 2.60 (m, 2H), 2.60 – 2.53 (m, 2H), 1.91 – 1.83 (m, 2H), 1.76 – 1.70 (m, 2H), 1.69 – 1.62 (m, 4H).

^{13}C NMR (126 MHz, $CDCl_3$) δ 163.96, 145.0, 137.5, 129.8, 128.9, 123.4, 116.3, 37.1, 30.7, 30.1, 30.0, 27.5, 24.2.

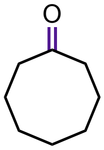

Cyclooctanone (3ag)

Following General Procedure A, prepared from 0.60 mmol of cyclooctanol(**1ag**) and 3.5 equiv. of **5** in 96 h, the title compound was isolated via flash chromatography (1 – 5% Acetone/Hexane) as a clear oil (50 mg, 64% yield). All analytical data for **3ag** was in accordance with literature data.[23, 29]

^{1}H NMR (400 MHz, $CDCl_3$) (δ, ppm): 2.44 – 2.33 (m, 4H), 1.92 – 1.79 (m, 4H), 1.52 (qt, *J* = 5.9, 2.9 Hz, 4H), 1.36 (tt, *J* = 5.6, 2.8 Hz, 2H).
^{13}C NMR (101 MHz, $CDCl_3$) (δ, ppm): 218.1, 41.7, 27.0, 25.5, 24.5.

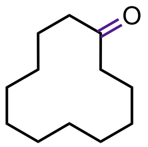

Cyclododecanone (3ah)

Following General Procedure A, prepared from 0.45 mmol of cyclododecanol (**1ah**) and 3.5 equiv. of **5** in 96 h, the title compound was isolated via flash chromatography (1 – 5% Acetone/Hexane) as a pale oil (40 mg, 48% yield). All analytical data for **3ah** was in accordance with literature data.[21]

^{1}H NMR (500 MHz, $CDCl_3$) (δ, ppm): 2.49 – 2.43 (m, 4H), 1.71 (p, *J* = 6.4 Hz, 4H), 1.28 (dd, *J* = 13.2, 5.6 Hz, 14H).
^{13}C NMR (101 MHz, $CDCl_3$) (δ, ppm): 213.1, 40.5, 24.9, 24.7, 24.3, 22.7, 22.5.

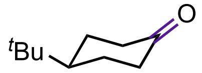

4-(*tert*-Butyl)cyclohexan-1-one (3ai)

Following General Procedure A, prepared from 0.57 mmol of 4-(*tert*-butyl)cyclohexan-1-ol (**1ai**) and 1.1 equiv. of **5** in 24 h, the title compound was isolated via flash chromatography (1 – 5% Acetone/Hexane) as a pale white solid (66 mg, 75% yield). All analytical data for **3ai** was in accordance with literature data.[31]

^{1}H NMR (500 MHz, $CDCl_3$) (δ, ppm): 2.40 (d, *J* = 16.0 Hz, 2H), 2.39 – 2.25 (m, 2H), 2.13 – 2.04 (m, 2H), 1.54 – 1.38 (m, 3H), 0.92 (s, 9H).
^{13}C NMR (126 MHz, $CDCl_3$) (δ, ppm): 213.1, 47.1, 41.7, 32.9, 28.0.

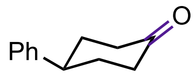

4-Phenylcyclohexan-1-one (3aj)

Following General Procedure A, prepared from 0.49 mmol of 4-phenylcyclohexan-1-ol (**1aj**) and 1.1 equiv. of **5** in 24 h, the title compound was isolated via flash chromatography (1 – 5% Acetone/Hexane) as pale white crystals (46 mg, 54% yield). All analytical data for **3aj** was in accordance with literature data.[32]

^{1}**H NMR** (500 MHz, $CDCl_3$) (δ, ppm): 7.37 – 7.29 (m, 2H), 7.27 – 7.18 (m, 3H), 3.03 (tt, *J* = 12.1, 3.4 Hz, 1H), 2.52 (dd, *J* = 11.7, 5.2 Hz, 4H), 2.28 – 2.19 (m, 2H), 2.03 – 1.88 (m, 2H).
^{13}**C NMR** (126 MHz, $CDCl_3$) (δ, ppm): 211.6, 145.2, 129.1, 127.1, 127.0, 43.2, 41.8, 34.4.

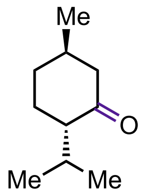

2-Isopropyl-5-methylcyclohexan-1-one (3ak)
Following General Procedure A, prepared from 0.60 mmol of 2-isopropyl-5-methylcyclohexan-1-ol (**1ak**) in 24 h. Title compound menthone was prepared using 1.0 equiv. of 2-chloro 4-nitropyridine. The crude reaction mixture was concentrated on *vacuo* and added a minimal amount of diethyl ether. The precipitates were filtered and added 2 N HCl in diethyl ether (0.5 ml). Precipitates were filtered again, and the desired compound was isolated via flash chromatography (0 – 2.5% Acetone/Hexane) as a white solid (49 mg, 50% yield). All analytical data for **3ak** was in accordance with literature data.[33]

^{1}**H NMR** (500 MHz, $CDCl_3$) (δ, ppm): 2.33 (ddd, *J* = 12.9, 3.8, 2.2 Hz, 1H), 2.17 – 2.08 (m, 1H), 2.08 – 1.98 (m, 2H), 1.95 (d, *J* = 12.8 Hz, 1H), 1.86 (ddd, *J* = 19.1, 8.0, 3.8 Hz, 2H), 1.43 – 1.26 (m, 2H), 0.99 (d, *J* = 6.4 Hz, 3H), 0.89 (d, *J* = 6.8 Hz, 3H), 0.83 (d, *J* = 6.8 Hz, 3H).
^{13}**C NMR** (126 MHz, $CDCl_3$) (δ, ppm): 212.6, 56.0, 51.0, 35.6, 34.0, 28.0, 26.0, 22.4, 21.3, 18.8.

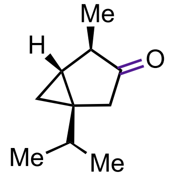

4-Phenylcyclohexan-1-one (3al)
Following General Procedure A, prepared from 0.50 mmol of synthesized 4-phenylcyclohexan-1-one (**1al**)[34] and 3.5 equiv. of **5** in 24 h. The title compound was isolated via flash chromatography (1 – 5% Acetone/Hexane) as a clear oil (41 mg, 54% yield). All analytical data for **3al** was in accordance with literature data.[35]

^{1}**H NMR** (400 MHz, $CDCl_3$) (δ, ppm): 2.54 (ddd, *J* = 18.8, 2.6, 1.3 Hz, 1H), 2.21 (qd, *J* = 7.5, 1.4 Hz, 1H), 2.06 (d, *J* = 18.8 Hz, 1H), 1.35 (dt, *J* = 13.7, 6.8 Hz, 1H), 1.15 (d, *J* = 7.5 Hz, 3H), 1.08 (dd, *J* = 8.1, 4.0 Hz, 1H), 1.00 (d, *J* = 6.7 Hz, 3H), 0.95 (d, *J* = 6.8 Hz, 3H), 0.75 (ddd, *J* = 8.1, 5.6, 2.4 Hz, 1H), 0.12 (dd, *J* = 5.7, 4.0 Hz, 1H).
^{13}**C NMR** (126 MHz, $CDCl_3$) (δ, ppm): 221.7, 47.5, 39.9, 33.1, 31.4, 29.8, 25.7, 20.2, 19.9, 18.9, 18.4.

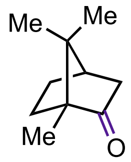

(1*S*,4*R*)-1,7,7-Trimethylbicyclo[2.2.1]heptan-2-one (Camphor) (3am)
Following General Procedure A, prepared from 0.50 mmol of (1*S*)-1,7,7-trimethylbicyclo[2.2.1]heptan-2-ol (borneol) (**1al**) and 3.5 equiv. of **5** in 90 h. The title compound was isolated via flash chromatography (1 – 5% Acetone/Hexane) as a white solid (67 mg, 81% yield). All analytical data for **3am** was in accordance with literature data.[36]

^{1}H NMR (500 MHz, $CDCl_3$) (δ, ppm): 2.34 (dt, *J* = 18.2, 3.9 Hz, 1H), 2.08 (t, *J* = 4.5 Hz, 1H), 1.94 (tq, *J* = 12.0, 4.1 Hz, 1H), 1.83 (d, *J* = 18.2 Hz, 1H), 1.67 (td, *J* = 13.3, 12.8, 3.7 Hz, 1H), 1.45 – 1.28 (m, 2H), 0.95 (s, 3H), 0.90 (s, 3H), 0.82 (s, 3H).
^{13}C NMR (126 MHz, $CDCl_3$) (δ, ppm): 220.2, 58.1, 47.2, 43.7, 43.5, 30.3, 27.5, 20.2, 19.6, 9.7.

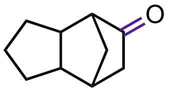

(3a*R*,4*S*,7*S*,7a*R*)-Octahydro-5H-4,7-methanoinden-5-one (3an)
Following General Procedure A, prepared from 0.49 mmol **(3a*R*,4*S*,7*S*,7a*R*)-octahydro-5H-4,7-methanoinden-5-ol** and 1.1 equiv. of **5** in 24 h. The title compound was isolated via flash chromatography (1 – 5% Acetone/Pentane) as a pale-yellow oil (59 mg, 81% yield). All analytical data for **3an** was in accordance with literature data.[37]

^{1}H NMR (400 MHz, $CDCl_3$) (δ, ppm): 2.44 – 2.34 (m, 2H), 2.12 (q, *J* = 7.0 Hz, 2H), 2.07 – 1.91 (m, 3H), 1.82 – 1.71 (m, 3H), 1.52 (dp, *J* = 10.8, 1.5 Hz, 1H), 1.42 – 1.29 (m, 1H), 1.15 – 1.02 (m, 2H).
^{13}C NMR (101 MHz, $CDCl_3$) (δ, ppm): 218.0, 54.2, 46.7, 44.4, 41.8, 39.6, 32.2, 31.5, 31.3, 27.9.

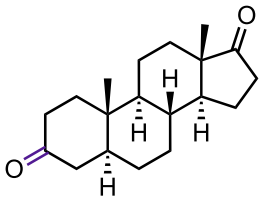

(8*R*,9*S*,10*S*,13*S*,14*S*)-10,13-Dimethyltetradecahydro-3*H*-cyclopenta[a]phenanthrene-3,17(2*H*)-dione (3ao)
Following General Procedure A, prepared from 0.45 mmol of (8*R*,9*S*,10*S*,13*S*,14*S*)-3-hydroxy-10,13-dimethylhexadecahydro-17H-cyclopenta[a]phenanthren-17-one and 2.0 equiv. of **5** in 24 h with minimal amount of dry and degassed DCM, the title compound was purified via flash

chromatography (1 – 15% EtOAc/Hexane) as a white solid (120 mg, 86% yield). All analytical data for **3ao** was in accordance with literature data.[29]

^{1}H NMR (500 MHz, $CDCl_3$) (δ, ppm): 2.49 – 2.24 (m, 4H), 2.14 – 1.99 (m, 3H), 1.98 – 1.90 (m, 1H), 1.83 (dt, J = 12.9, 3.5 Hz, 2H), 1.69 (dd, J = 13.6, 2.9 Hz, 1H), 1.65 – 1.48 (m, 3H), 1.47 – 1.22 (m, 7H), 1.03 (s, 3H), 0.88 (s, 3H), 0.79 (td, J = 12.0, 3.8 Hz, 1H).
^{13}C NMR (126 MHz, $CDCl_3$) (δ, ppm): 220.9, 211.5, 53.8, 51.1, 47.6, 46.5, 44.5, 38.3, 38.0, 35.7, 34.8, 31.3, 30.4, 28.5, 21.7, 20.6, 13.7, 11.4.

Characterization data of imines

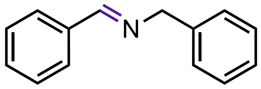

(*E*)-*N*-Benzyl-1-phenylmethanimine (4a)
Following General Procedure C, prepared from 0.50 mmol of dibenzylamine (**2a**) in 24 h, the title compound was purified by flash column chromatography (5% NEt_3/Hexane) as a colorless oil (69 mg, 70% yield). All analytical data for **4a** is in accordance with literature data.[38]

^{1}H NMR (500 MHz, $CDCl_3$) (δ, ppm): 8.41 (s, 1H), 7.82 – 7.75 (m, 2H), 7.45 – 7.39 (m, 3H), 7.35 (d, J = 4.6 Hz, 4H), 7.31 – 7.22 (m, 1H), 4.84 (s, 2H).
^{13}C NMR (126 MHz, $CDCl_3$) (δ, ppm): 163.5, 139.4, 136.3, 130.9, 128.7, 128.6, 128.4, 128.1, 127.1, 65.2.

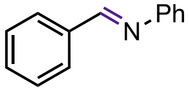

(*E*)-*N*-1-Diphenylmethanimine (4b)
Following General Procedure C, prepared from 0.50 mmol of *N*,1-diphenylmethanamine (**2b**) in 24 h, the title compound was purified by flash column chromatography (5% NEt_3/Hexane) as a yellow oil (40 mg, 44% yield). All analytical data for **4b** is in accordance with literature data.[38]

^{1}H NMR (500 MHz, $CDCl_3$) (δ, ppm): 8.47 (s, 1H), 7.95 – 7.87 (m, 2H), 7.53 – 7.44 (m, 3H), 7.44 – 7.37 (m, 2H), 7.28 – 7.21 (m, 3H).
^{13}C NMR (126 MHz, $CDCl_3$) (δ, ppm): 160.6, 152.2, 136.4, 131.5, 129.3, 129.0, 128.9, 126.1, 121.0.

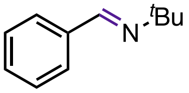

(*E*)-*N*-*tert*-Butyl-1-phenylmethanimine (4c)
Following General Procedure C, prepared from 0.50 mmol of *N*-*tert*-butyl-1-phenylmethanimine (**2c**). ^{1}H NMR yield determined using CH_2Br_2 as an internal standard (62% yield). All analytical data for **4c** is in accordance with literature data.[39]

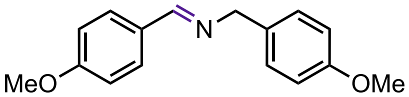

***(E)*-*N*-(4-Methoxybenzyl)-1-(4-methoxyphenyl)methanimine (4d)**

Following General Procedure C, prepared from 0.50 mmol of bis(4-methoxybenzyl)amine (**2d**) in 24 h. ^{1}H NMR yield determined using CH_2Br_2 as an internal standard (43% yield). All analytical data for **4d** was in accordance with literature data.[40]

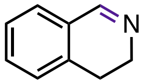

3,4-Dihydroisoquinoline (4e)

Following General Procedure C, prepared from 0.50 mmol of 1,2,3,4-tetrahydroisoquinoline (**2e**), in 1.0 mL of $PhCF_3$ for 24h. ^{1}H NMR yield determined using CH_2Br_2 as an internal standard (>99% yield, 4.6:1 of 3,4-dihydroisoquinoline to isoquinoline). All analytical data for **4e** was in accordance with literature data.[41]

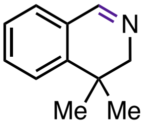

4,4-Dimethyl-3,4-dihydroisoquinoline (4f)

Following General Procedure C, prepared from 0.50 mmol of 4,4-dimethyl-1,2,3,4-tetrahydroisoquinoline (**2f**) in 24h, the title compound was purified by flash column chromatography (5% NEt_3/Hexane) as a pale-yellow solid (69 mg, 86% yield). All analytical data for **4f** is in accordance with the literature data.[42]

^{1}H NMR (400 MHz, $CDCl_3$) (δ, ppm): 8.37 (s, 1H), 7.42 (ddd, J = 7.7, 5.9, 2.8 Hz, 1H), 7.38 – 7.33 (m, 1H), 7.32 – 7.27 (m, 2H), 3.62 (s, 2H), 1.24 (s, 6H).
^{13}C NMR (101 MHz, $CDCl_3$) (δ, ppm): 160.7, 145.6, 131.9, 127.8, 126.8, 123.6, 61.1, 31.6, 26.5.

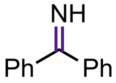

Diphenylmethanamine (4g)

Following General Procedure C, prepared from 0.50 mmol of diphenylmethanamine (**2g**) in 24 h. ^{1}H NMR yield determined using CH_2Br_2 as an internal standard (85% yield). All analytical data for **4g** was in accordance with literature data.[43]

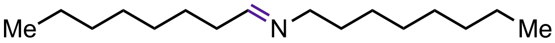

(*E*)-*N*-Octyloctan-1-imine (4h)

Following General Procedure C, prepared from 0.50 mmol of dioctylamine (**2h**) in 24 h. ^{1}H NMR yield determined using CH_2Cl_2 as an internal standard (40% yield). All analytical data for **4h** was in accordance with literature data.[40]

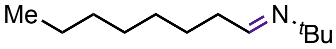

(*E*)-*N*-(*tert*-Butyl)octan-1-imine (4i)
Following General Procedure C, prepared from 0.50 mmol of synthesized *N*-(*tert*-butyl)octan-1-amine (**2i**)[44a] in 24 h. ^{1}H NMR yield determined using CH_2Cl_2 as an internal standard (22% yield). All analytical data for **4i** was in accordance with literature data.[44b]

Characterization data of carboxylic acids

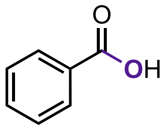

Benzoic acid (10a)
Following General Procedure D, prepared from 0.50 mmol of benzaldehyde (**8a**) in 24 h, the title compound was isolated via flash chromatography (0 – 50% EtOAc/Hexane) as a white solid (53 mg, 87% yield). All analytical data for **10a** was in accordance with literature data.[45]

^{1}H NMR (500 MHz, $CDCl_3$) (δ, ppm): 11.75 (s, 1H), 8.13 (d, *J* = 7.3 Hz, 2H), 7.62 (t, *J* = 7.4 Hz, 1H), 7.49 (t, *J* = 7.7 Hz, 2H).
^{13}C NMR (101 MHz, $CDCl_3$) (δ, ppm): 171.8, 134.0, 130.4, 129.4, 128.7.

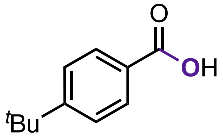

4-(*tert*-Butyl)benzoic acid (10b)Following General Procedure D, prepared from 0.50 mmol of 4-(*tert*-butyl)benzaldehyde (**8b**) in 36 h, the title compound was isolated via flash chromatography (0 – 50% EtOAc/Hexane) as a white solid (60 mg, 67% yield). All analytical data for **10b** was in accordance with literature data.[46]

^{1}H NMR (400 MHz, $CDCl_3$) (δ, ppm): 8.06 (d, *J* = 8.2 Hz, 2H), 7.50 (d, *J* = 7.9 Hz, 2H), 1.36 (s, 3H).
^{13}C NMR (101 MHz, $CDCl_3$) (δ, ppm): 172.5, 157.8, 130.3, 126.7, 125.6, 35.4, 31.4, 31.3.

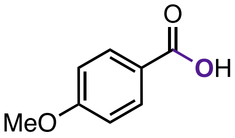

4-Methoxybenzoic acid (10c)
Following General Procedure D, prepared from 0.50 mmol of 4-methoxybenzaldehyde (**8c**) in 36 h, the title compound was isolated via flash chromatography (0 – 50% EtOAc/Hexane) as a white solid (52 mg, 69% yield). All analytical data for **10c** was in accordance with literature data.[45]

^{1}H NMR (500 MHz, $CDCl_3$) (δ, ppm): 8.07 (d, *J* = 8.7 Hz, 2H), 6.95 (d, *J* = 8.6 Hz, 2H), 3.88 (s, 3H).

^{13}C NMR (126 MHz, $CDCl_3$) (δ, ppm): 171.8, 164.2, 132.5, 121.8, 113.9, 55.6.

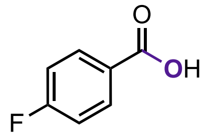

4-Fluorobenzoic acid (10d)

Following General Procedure D, prepared from 0.50 mmol of 4-fluorobenzaldehyde (**8d**) in 24 h, The title compound was isolated via flash chromatography (0 – 50% EtOAc/Hexane) as a white solid (60 mg, 85% yield). All analytical data for **10d** was in accordance with the literature data.[45]

^{1}H NMR (500 MHz, $CDCl_3$) (δ, ppm): 8.18 – 8.10 (m, 2H), 7.15 (t, J = 8.6 Hz, 2H).
^{13}C NMR (126 MHz, $CDCl_3$) (δ, ppm): 171.1, 166.5 (d, J = 255.4 Hz), 133.1 (d, J = 9.6 Hz), 125.6 (d, J = 3.1 Hz), 115.9 (d, J = 22.1 Hz).

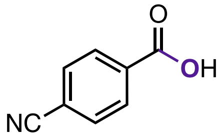

4-Cyanobenzoic acid (10e)

Following General Procedure D, prepared from 0.50 mmol of 4-cyanobenzaldehyde (**8e**) in 24 h, title compound was isolated via flash chromatography (0 – 50% EtOAc/Hexane) as a white solid (64 mg, 87% yield). All analytical data for **10e** was in accordance with the literature data.[47]

^{1}H NMR (500 MHz, $CDCl_3$) (δ, ppm): 8.21 (d, J = 8.4 Hz, 2H), 7.80 (d, J = 8.4 Hz, 2H).
^{13}C NMR (126 MHz, $CDCl_3$) (δ, ppm): 170.0, 133.0, 132.5, 130.9, 117.9, 117.5.

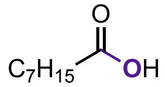

Octanoic acid (10f)

Following General Procedure D, prepared from 0.50 mmol of octanal (**8f**) with 1.0 equiv. of **5**, and 1.0 equiv. H_2O in MeCN (1.0M) in 12 h, the title compound was isolated via flash chromatography (0 – 50% EtOAc/Hexane) a colorless oil (35 mg, 49% yield). All analytical data for **10f** was in accordance with literature data.[48]

^{1}H NMR (400 MHz, $CDCl_3$) (δ, ppm): 2.35 (t, J = 7.5 Hz, 2H), 1.63 (p, J = 7.4 Hz, 2H), 1.40 – 1.21 (m, 8H), 0.92 – 0.83 (m, 3H).
^{13}C NMR (126 MHz, $CDCl_3$) (δ, ppm): 180.7, 34.3, 31.8, 29.2, 29.0, 24.8, 22.7, 14.2.

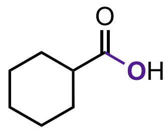

Cyclohexanecarboxylic acid (10g)

Following General Procedure D, prepared from 0.50 mmol of cyclohexanecarbaldehyde (**8g**) with 1.0 equiv. of **5**, and 1.0 equiv. H_2O in MeCN (1.0M) in 12 h, the title compound was isolated via flash chromatography (0 – 50% EtOAc/Hexane) as a yellow oil (42 mg, 65% yield). All analytical data for **10g** was in accordance with literature data.[48]

^{1}H NMR (500 MHz, $CDCl_3$) (δ, ppm): 11.71 (s, 1H), 2.33 (tt, *J* = 11.3, 3.7 Hz, 1H), 1.93 (dt, *J* = 12.9, 3.9 Hz, 2H), 1.76 (dp, *J* = 11.1, 3.8 Hz, 2H), 1.64 (dd, *J* = 11.0, 4.7 Hz, 1H), 1.45 (qd, *J* = 11.6, 3.4 Hz, 2H), 1.35 – 1.17 (m, 3H).
^{13}C NMR (126 MHz, $CDCl_3$) (δ, ppm): 182.8, 43.1, 28.9, 25.8, 25.5.

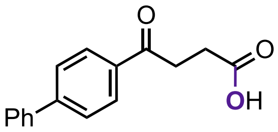

4-([1,1'-Biphenyl]-4-yl)-4-oxobutanoic acid (Fenbufen) (10h)

Following General Procedure D, prepared from 0.50 mmol of 4-([1,1'-biphenyl]-4-yl)-4-oxobutanal (**8h**) with 1.0 equiv. of **5**, and 1.0 equiv. H_2O in MeCN (1.0M) in 12 h, the title compound was isolated via flash chromatography (0 – 50% EtOAc/Hexane) as a white solid (70 mg, 56% yield). All analytical data for **10h** was in accordance with literature data.[49]

^{1}H NMR (400 MHz, DMSO-d_6) (δ, ppm): 12.21 (s, 1H), 8.07 (d, *J* = 8.1 Hz, 2H), 7.83 (d, *J* = 8.2 Hz, 2H), 7.75 (d, *J* = 7.3 Hz, 2H), 7.51 (t, *J* = 7.5 Hz, 2H), 7.43 (dd, *J* = 8.4, 6.1 Hz, 1H), 3.29 (t, *J* = 6.2 Hz, 2H), 2.61 (t, *J* = 6.2 Hz, 2H).
^{13}C NMR (101 MHz, DMSO-d_6) (δ, ppm): 198.1, 173.8, 144.5, 138.9, 135.3, 129.1, 128.6, 128.4, 127.0, 126.9, 33.2, 27.9.

Characterization data of amides

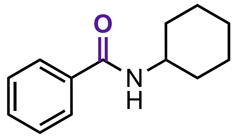

***N*-Cyclohexylbenzamide (11a)**

Following General Procedure E, prepared from cyclohexanamine (0.50 mmol, 1.00 equiv.) and benzaldehyde (0.50 mmol, 1.00 equiv.) in 24 h, the title compound was isolated via flash chromatography (0 – 50% EtOAc/Hexane) as a yellow solid (86 mg, 85% yield). All analytical data for **11a** was in accordance with literature data.[50]

^{1}H NMR (400 MHz, $CDCl_3$) (δ, ppm): 7.75 (d, *J* = 7.2 Hz, 2H), 7.48 (t, *J* = 7.3 Hz, 1H), 7.42 (t, *J* = 7.3 Hz, 2H), 5.97 (s, 1H, NH), 3.98 (tdt, *J* = 11.4, 7.9, 3.9 Hz, 1H), 2.03 (dq, *J* = 12.3, 3.9 Hz, 2H), 1.75 (dp, *J* = 11.8, 3.9 Hz, 2H), 1.66 (dp, *J* = 11.5, 4.0, 3.3 Hz, 1H), 1.43 (qt, *J* = 12.5, 3.5 Hz, 2H), 1.34 – 1.13 (m, 3H).
^{13}C NMR (101 MHz, $CDCl_3$) (δ, ppm): 166.8, 135.3, 131.4, 128.7, 127.0, 48.8, 33.4, 25.7, 25.0.

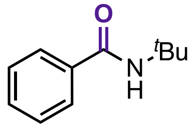

N-(*tert*-Butyl)benzamide (11b)
Following General Procedure E, prepared from *tert*-butylamine (0.50 mmol, 1.00 equiv.) and benzaldehyde (0.50 mmol, 1.00 equiv.) in 24 h, the title compound was isolated via flash chromatography (0 – 50% EtOAc/Hexane) as a white solid (72 mg, 81% yield). All analytical data for **11b** was in accordance with literature data.[51]

^{1}H NMR (400 MHz, $CDCl_3$) (δ, ppm): 7.75 – 7.68 (m, 2H), 7.53 – 7.36 (m, 3H), 5.93 (s, 1H, NH), 1.48 (s, 9H).
^{13}C NMR (101 MHz, $CDCl_3$) (δ, ppm): 167.1, 136.1, 131.2, 128.6, 126.8, 51.8, 29.0.

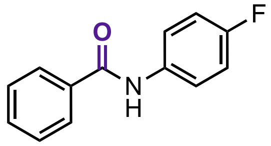

N-(4-Fluorophenyl)benzamide (11c)
Following General Procedure E, prepared from 4-fluoroaniline (0.50 mmol, 1.00 equiv.) and benzaldehyde (0.50 mmol, 1.00 equiv.) in 24 h, the title compound was isolated via flash chromatography (0 – 50% EtOAc/Hexane) as a yellow solid (73 mg, 68% yield). All analytical data for **11c** was in accordance with literature data.[52]

^{1}H NMR (500 MHz, $CDCl_3$) (δ, ppm): 7.86 (d, J = 7.6 Hz, 2H), 7.81 (s, 1H, NH), 7.60 (dd, J = 8.7, 4.9 Hz, 2H), 7.55 (d, J = 7.3 Hz, 1H), 7.49 (t, J = 7.6 Hz, 2H), 7.06 (t, J = 8.4 Hz, 2H).
^{13}C NMR (126 MHz, $CDCl_3$) (δ, ppm): 165.9, 159.7 (d, J = 244.1 Hz), 134.9, 134.0 (d, J = 2.9 Hz), 132.1, 129.0, 127.1, 122.2 (d, J = 7.9 Hz), 115.9 (d, J = 22.4 Hz).

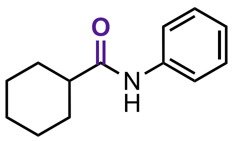

N-Phenylcyclohexanamide (11d)
Following General Procedure E, prepared from aniline (0.50 mmol, 1.00 equiv.) and cyclohexanecarbaldehyde (0.50 mmol, 1.00 equiv.) in 24 h, the title compound was isolated via flash chromatography (0 – 50% EtOAc/Hexane) as a white solid (72 mg, 71% yield). All analytical data for **11d** was in accordance with literature data.[52]

^{1}H NMR (400 MHz, $CDCl_3$) (δ, ppm): 7.52 (d, J = 7.4 Hz, 2H), 7.31 (dd, J = 8.6, 7.3 Hz, 2H), 7.09 (t, J = 7.4 Hz, 1H), 2.23 (tt, J = 11.7, 3.5 Hz, 1H), 1.96 (d, J = 13.0 Hz, 2H), 1.85 (dd, J = 13.0, 3.4 Hz, 2H), 1.75 – 1.65 (m, 1H), 1.62 – 1.48 (m, 2H), 1.39 – 1.18 (m, 2H).
^{13}C NMR (101 MHz, $CDCl_3$) (δ, ppm): 174.4, 138.2, 129.1, 124.2, 119.8, 46.8, 29.8, 25.8.

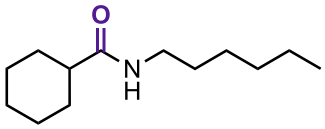

N-Hexylcyclohexanecarboxamide (11e)

Following General Procedure E, prepared from hexylamine (0.50 mmol, 1.00 equiv.) and cyclohexanecarbaldehyde (0.50 mmol, 1.00 equiv.) in 24 h, the title compound was isolated via flash chromatography (0 – 50% EtOAc/Hexane) as a white solid (65 mg, 62% yield). All analytical data for **11e** was in accordance with literature data.[51]

^{1}H NMR (500 MHz, $CDCl_3$) (δ, ppm): 5.51 (s, 1H, NH), 3.21 (q, J = 6.7 Hz, 2H), 2.04 (tt, J = 11.8, 3.5 Hz, 1H), 1.83 (d, J = 13.1 Hz, 2H), 1.77 (d, J = 10.6 Hz, 2H), 1.68 – 1.62 (m, 1H), 1.51 – 1.35 (m, 4H), 1.36 – 1.09 (m, 9H), 0.86 (t, J = 6.6 Hz, 3H).
^{13}C NMR (126 MHz, $CDCl_3$) (δ, ppm):176.2, 45.8, 39.5, 31.6, 29.9, 29.8, 26.7, 25.9, 22.7, 14.1.

Photo-Flow reaction data

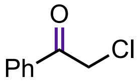

2-Chloro-1-phenylethan-1-one (3q)

Following General Procedure F, using 2-chloro-1-phenylethan-1-ol (0.20 mmol, 1.00 equiv.) and 2-bromo-6-nitropyridine (0.15 mmol, 0.75 equiv.) in trifluorotoluene (2.0 mL). The residence time was 5 h. The ^{1}H NMR yield was determined using CH_2Br_2, yield = 82%.

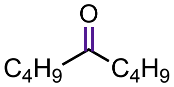

5-Nonanone (3z)

Following General Procedure F, using nonan-5-ol (0.20 mmol, 1.00 equiv.) and 3,5-bis(trifluoromethyl)nitrobenzene (0.40 mmol, 2.00 equiv.) in MeCN/*t*BuOH (5:1, 2.0 mL). The residence time was 5 h. The ^{1}H NMR yield was determined using CH_2Br_2, yield = 97%.

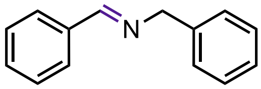

(*E*)-*N*-Benzyl-1-phenylmethanimine (4a)

Following General Procedure F, using dibenzylamine (0.20 mmol, 1.00 equiv.) and 5-bromo-2-nitropyridine (0.40 mmol, 2.00 equiv.) in DCM (2.0 mL). The residence time was 3 h. The ^{1}H NMR yield was determined using CH_2Br_2, yield = 75%.

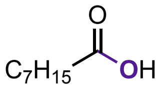

Octanoic acid (10f)

Following General Procedure F, using octanal (0.20 mmol, 1.00 equiv.) and 3,5-bis(trifluoromethyl)nitrobenzene (0.20 mmol, 1.00 equiv.) in MeCN/H_2O (10:1, 2.0 mL). The residence time was 4 h. The ^{1}H NMR yield was determined using CH_2Br_2, yield = 58%.

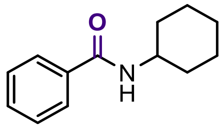

***N*-Cyclohexylbenzamide (11a)**

Following General Procedure F, using cyclohexanamine (0.20 mmol, 1.00 equiv.), benzaldehyde (0.20 mmol, 1.00 equiv.) and 3,5-bis(trifluoromethyl)nitrobenzene (0.20 mmol, 1.00 equiv.) in DCM/*t*BuOH (5:1, 2.0 mL). The mixture was first pre-stirred for 4 h at room temperature to form *N*-cyclohexyl-1-phenylmethanimine in situ which was then subject to flow photochemical conditions. The residence time was 5 h. The ^{1}H NMR yield was determined using CH_2Br_2, yield = 82%.

Mechanism studies

Kinetic isotope effect studies

Benzylic C(sp^3) Anaerobic oxidation.

Independent Rate Measurement. To an oven-dried, 0.5-dram vial, equipped with a stir bar was added of 3,5-Bis(trifluoromethyl)nitrobenzene (16 mg, 1.0 equiv., 0.060 mmol), LiOAc (1 mg, 0.20 equiv., 0.12 mmol), and synthesized 1-phenylethan-1,2,2,2-d$_4$-1-ol[53] (38 mg, 5.0 equiv., 0.30 mmol). In a separate oven-dried, 0.5-dram vial, a second vial equipped with a stir bar was added of 3,5-Bis(trifluoromethyl)nitrobenzene (16 mg, 1.0 equiv., 0.060 mmol), LiOAc (1 mg, 0.20 equiv., 0.12 mmol), and 1-phenylethan-ol (37 mg, 5.0 equiv., 0.30 mmol). Each reaction vessel was subjected to 390 nm light for 1 h. A crude ^{1}H NMR was taken for each vessel and the ratio of the relative abundance was observed ((δ, ppm): 7.51). Each KIE measurement is the average of two independent experiments.

Intermolecular Competition Rate Measurement. To an oven-dried, 0.5-dram vial, equipped with a stir bar was added of 3,5-Bis(trifluoromethyl)nitrobenzene (16 mg, 1.0 equiv., 0.060 mmol), LiOAc (1 mg, 0.20 equiv., 0.12 mmol), and prepared 1-phenylethan-1,2,2,2-d$_4$-1-ol[2] (38 mg, 5.0 equiv., 0.30 mmol) and 1-phenylethan-ol (37 mg, 5.0 equiv., 0.30 mmol). The reaction vessel was subjected to 390 nm light for 1 h. A crude ^{1}H NMR was taken using CH_2Br_2 as an internal standard ((δ, ppm): 2.57). The KIE measurement is the average of two independent experiments.

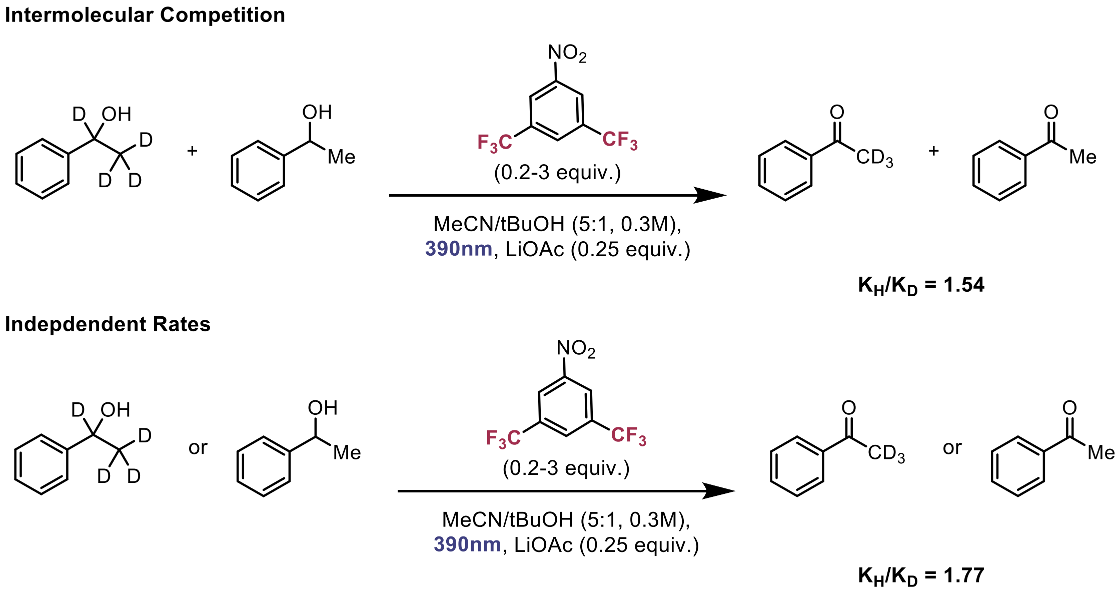

Figure S7. Intermolecular competition and independent rate measurements to determine KIE.

Table S11. Experimental KIE data for the reaction with **6** and **1d** and **1d-d$_4$**.

**Independent**	Standard	**3d** (7.51)	**3d-d$_3$** (7.51)	**3d**/ Standard	**3d-d$_3$**/ Standard	**KIE**
**Run 1**	2.00	0.38	-	0.19	-	
**Run 2**	2.00	0.45	-	0.23	-	
**Run 1**	2.00	-	0.26	--	0.13	1.46
**Run 2**	2.00	-	0.22	--	0.11	2.09
					**Average**	**1.77**

**Competition**	**(3d+ 3d-d$_3$)$_{obs}$** (2.57)	**3d** (2.57)	**(3d-d$_3$)$_{cal}$**	**KIE**
**Run 1**	2.00	1.82	1.18	1.54
**Run 2**	2.00	1.82	1.18	1.54
			**Average**	**1.54**

α-C(Sp^3)-O–D/H Anaerobic oxidation.

Independent Rate Measurement. To an oven-dried, 0.5-dram vial, equipped with a stir bar was added of 3,5-Bis(trifluoromethyl)nitrobenzene (65 mg, 1.0 equiv., 0.25 mmol), and 1-phenylethan-1-ol (62 mg, 2.0 equiv., 0.20 mmol) in 0.5 mL of Acetone-d$_6$ and 0.5 mL of D_2O and left stirring until the OH peak of 1-phenylethan-1-ol was not detected by ^{1}H NMR, indicating formation of O–D. In a separate oven-dried, 0.5-dram vial, a second vial equipped with a stir bar was added of 3,5-Bis(trifluoromethyl)nitrobenzene (65 mg, 1.0 equiv., 0.25 mmol), and 1-phenylethan-1-ol (61.6 mg, 2.00 equiv., 0.20 mmol) in 0.5 mL of Acetone-d$_6$ and 0.5 mL of H_2O and left stirring for the same amount of time as the prior was used to confirm retention of the O–H peak. The reaction vessels were then subjected to 390 nm light for 1 h. A crude ^{1}H NMR was taken using CH_2Br_2 as an internal standard ((δ, ppm): 7.51). The KIE measurement is the average of two independent experiments.

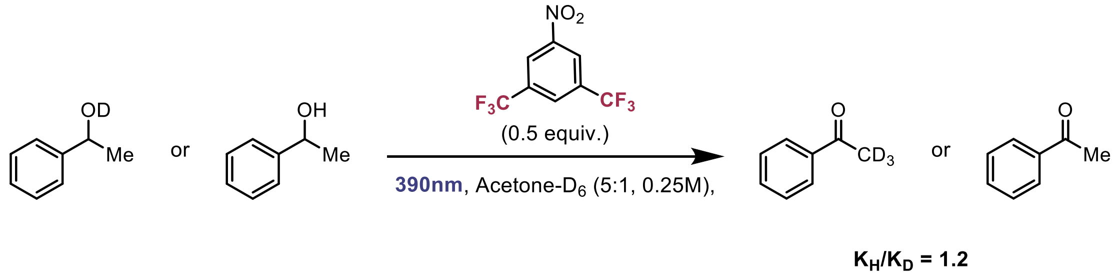

Figure S8. Independent rate measurements to determine KIE for O–D vs O–H.

Table S12. Experimental KIE data for the reaction of with **6** and **1d** and **1d-d₁**.

**Independent**	Standard	**3d** (7.51)	**3d-d₃** (7.51)	**3d**/ Standard	**3d-d₃**/ Standard	**KIE**
**Run 1**	1.00	0.50	-	0.50	-	
**Run 2**	2.00	0.59	-	0.59	-	
**Run 1**	2.00	-	0.59	--	0.59	1.18
**Run 2**	2.00	-	0.72	--	0.72	1.22
					**Average**	**1.20**

Overall Results. To shed light on whether abstraction of a proton at either carbon or oxygen was occurring, first Kinetic Isotope Effects (KIEs) of PhCH(OH)Me were determined using independent rate measurements and intermolecular competition experiments (Table S11). Independent anaerobic oxidations of $PhCD(OH)CD_3$ revealed a KIE for the C(sp^3)–H abstraction step, $k_H/k_D = 1.77$. Measurement of the competition experiments also showed an isotope effect for the benzylic ($k_H/k_D = 1.54$). These results indicate that for benzylic secondary alcohols, HAT at the α-C–H bond is possibly rate-limiting. Intermolecular competition experiments with PhCH(OD)Me and PhCH(OH)Me were not assessed due to the possible rapid exchange between alcohols of the H/D, which would lead to inconclusive results. However, Independent rate measurements were obtained, showing $k_H/k_D = 1.2$. This KIE is within the range for a secondary KIE of C(sp^3) to C(sp^2) transformations. This indicates that O–H/D bond breakage does occur after C–H breakage.

Radical clock study

Anaerobic Oxidation of a Radical Clock.

Anaerobic oxidation with radical clock, **18**[54] (112 mg, 1.00 equiv., 0.500 mmol). To a 0.5-dram oven-dried vial equipped with a stir bar was added 3,5-Bis(trifluoromethyl)nitrobenzene (389 mg, 3.00 equiv., 1.50 mmol) and LiOAc (8 mg, 0.25 equiv., 0.125 mmol) in a 5:1 MeCN/*t*BuOH mixture under N_2. The mixture was stirred at 1000 rpm under 390 nm light for 36 h. ^1H NMR spectrum was recorded using CH_2Br_2 as an internal standard. Products **20** and **21** were detected and their NMRs are in accordance with literature values.[55a, 55b]

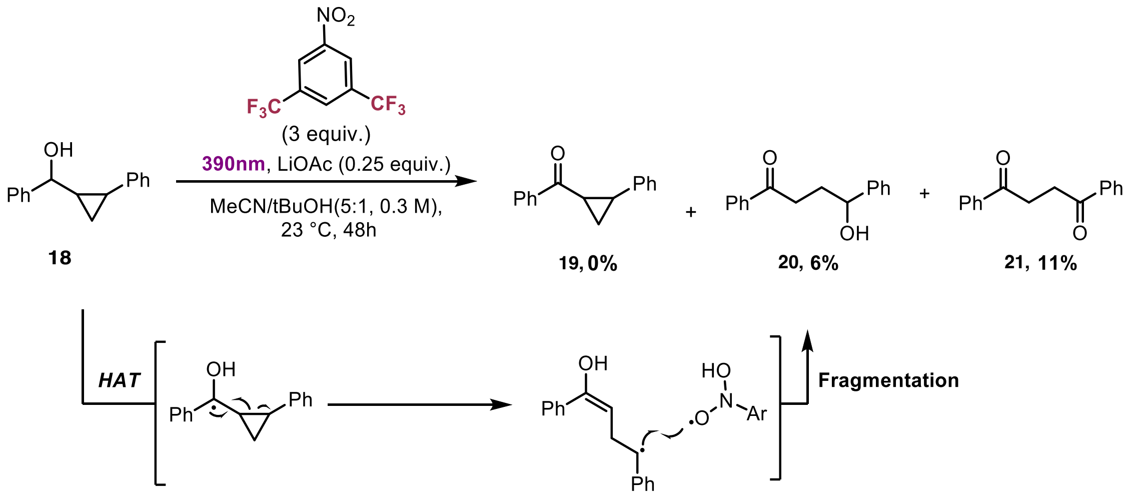

Figure S9. Radical clock subjected to the general reaction conditions.

Results. Support for a radically initiated process, at the α-C(sp^3) center for these oxidations was provided from a radical clock study using phenyl(2-phenylcyclopropyl)methanol (Figure S9). The anaerobic oxidation revealed a mixture of 4-hydroxy-1,4-diphenylbutan-1-one (**20**) ((δ, ppm): 3.13-3.11) and 1,4-diphenylbutane-1,4-dione (**21**) ((δ, ppm): 3.47) as major products. These products likely occur after an HAT event, which ring opens to form a benzylic carbon-centered-radical that can be captured by the photoexcited-nitroarene, resulting in hydroxylation, a recombination step similar to previously reported methods. No ketone product (**19**) was observed.

^{1}H NMR (500 MHz, $CDCl_3$) of phenyl(2-phenylcyclopropyl)methanol (18)

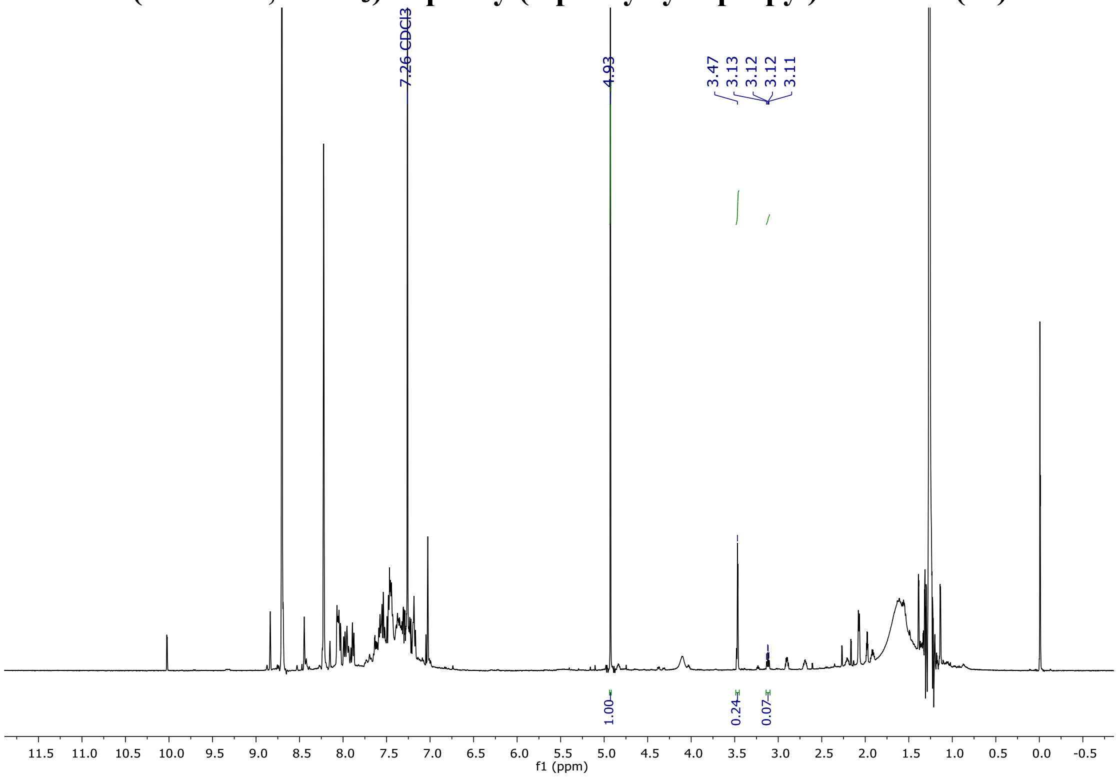

Labeling study

Anaerobic Oxidation of an ^{18}O-labelled Alcohol.

The anaerobic oxidation of ^{18}O-enriched phenylethane-1-ol (**1d-^{18}O**)[56] was carried out with 3.0 equiv. of 3,5-Bis(trifluoromethyl)nitrobenzene under General Procedure A for 24 h. Based on GCMS analysis of the reaction mixtures, product **3d-^{18}O** was solely found as opposed to **3d**, indicating ^{18}O was retained. ^{1}H NMR yield obtained using CH_2Br_2 as an internal standard showed 70% of product formation.

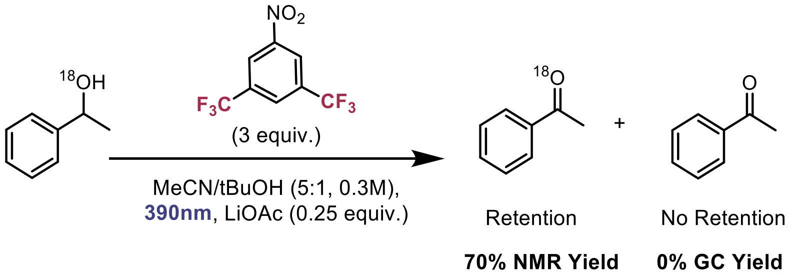

GC chromatograms of reactions with 1d-^{18}O.

Commercial Phenylethan-1-ol, **1d**

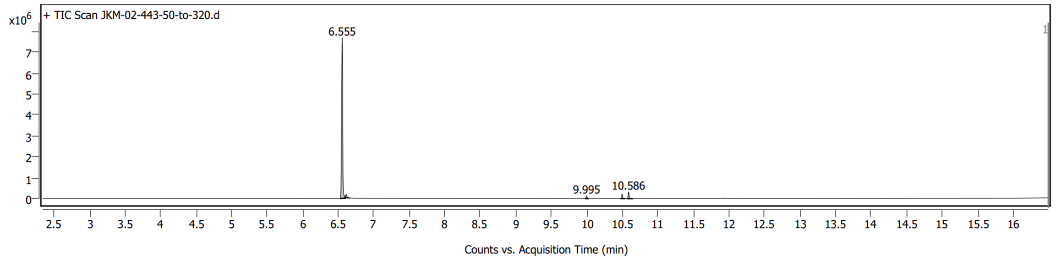

Synthesized Phenylethan-1-ol-d$_1$, **1d-^{18}O**

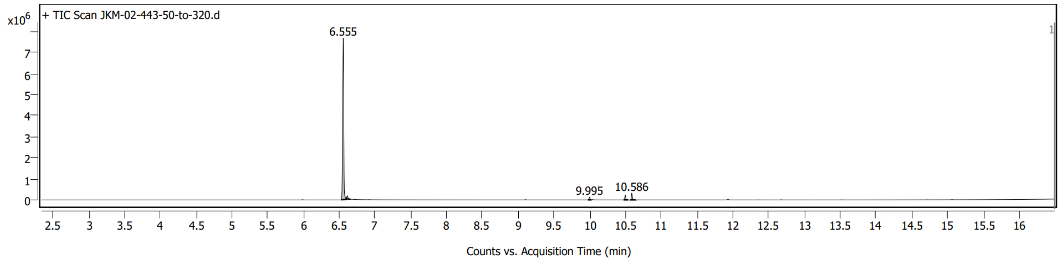

Reaction run with **1d-^{18}O**

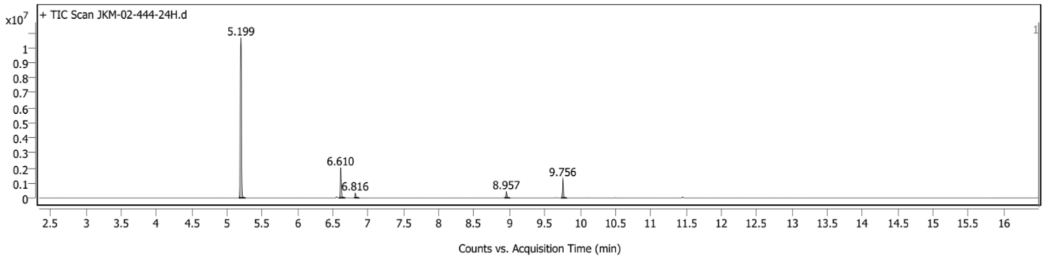

Figure S10. GCMS spectra of labeling study reactions with commercial Phenylethan-1-ol for comparison.

GCMS spectra of 1d, prepared 1d-^{18}O, and reaction run with 1d-^{18}O.

Commercial Phenylethan-1-ol, **1d**

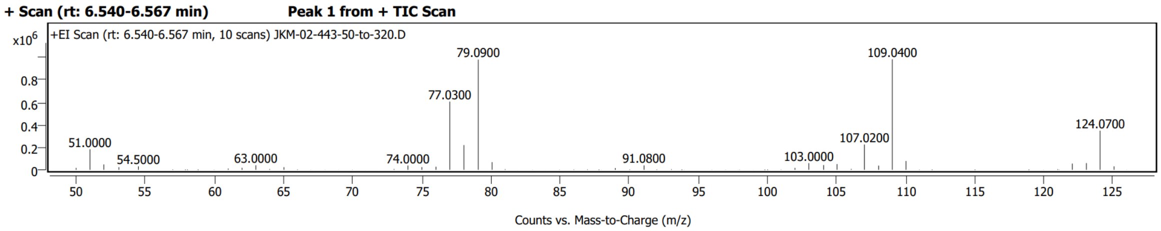

Synthesized Phenylethan-1-ol-d$_1$, **1d-^{18}O**

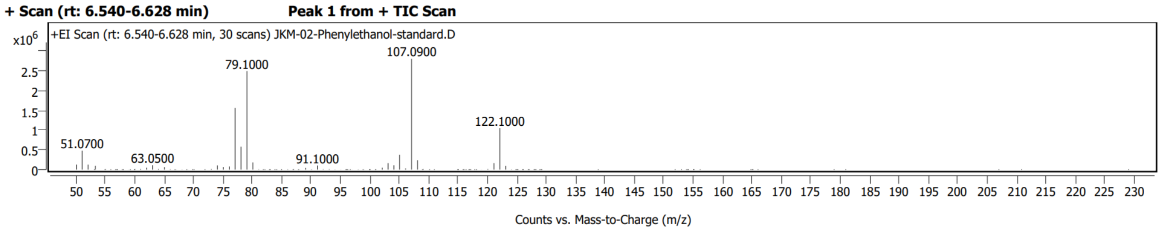

Reaction run with **1d-^{18}O**

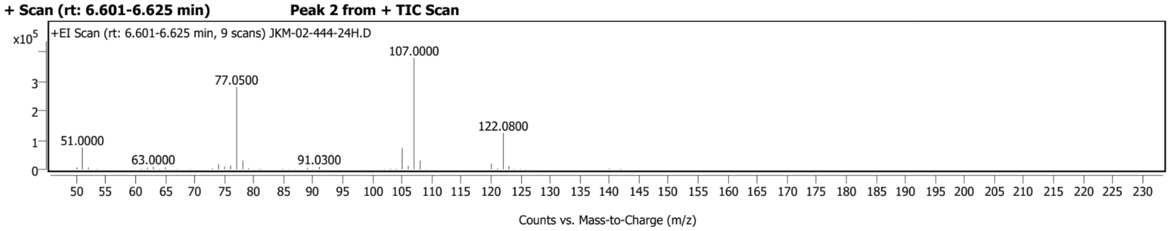

Figure S11. GCMS spectra of labeling studies reactions with commercial phenylethan-1-ol for comparison.

Hammett plot study

Independent experiments. The anaerobic oxidation of *para*-substituted phenylethane-1-ol (**1c-g**) were carried out independently. In an oven-dried vial equipped with a stir bar was charged alcohol (0.2 mmol, 1.0 equiv.), 3,5-Bis(trifluoromethyl)nitrobenzene (155 mg, 3 equiv., 0.6 mmol), and lithium acetate (3 mg, 0.2 equiv., 0.04 mmol) with MeCN/*t*BuOH (5:1, 0.3 M) under N_2. The reaction mixture was then irradiated under 390 nm light for 2 h. ^{1}H NMR yield obtained using CH_2Br_2 as an internal standard. Reactions were taken in duplicates.

Competition experiments. The anaerobic oxidation of *para*-substituted phenylethan-1-ol (**1c-g**) with phenylethane-1-ol were carried out independently. In an oven-dried vial equipped with a stir bar was charged *para*-substituted alcohol (0.2 mmol, 1.0 equiv.), phenylethane-1-ol (24 mg, 1.0 equiv., 0.2 mmol), 3,5-Bis(trifluoromethyl)nitrobenzene (155 mg, 3.00 equiv., 0.600 mmol), and lithium acetate (3 mg, 0.2 equiv., 0.04 mmol) with MeCN/*t*BuOH (5:1, 0.3 M) under N_2. The reaction mixture was then irradiated under 390 nm light for 2 h. ^{1}H NMR yield obtained using CH_2Br_2 as an internal standard. Reactions were taken in duplicates.

Results. For several *para*-substituted α-methyl benzyl alcohols, Hammett studies were conducted via independent rate measurements (Figure S12, Left) and competition experiments (Figure S12, Right). Of the varying Hammett Parameter (σ_p), independent rate measurements did not show significant electronic dependence among some of the substituents, but competition experiments did. The Hammett plots reveal preferential oxidation of electron-rich alcohols (ρ = -0.45). This observed Hammett is possible from loss of electron density and build-up of positive charge at the C(sp^3)–H adjacent to the hydroxyl in the transition state, which has been observed in previous studies.

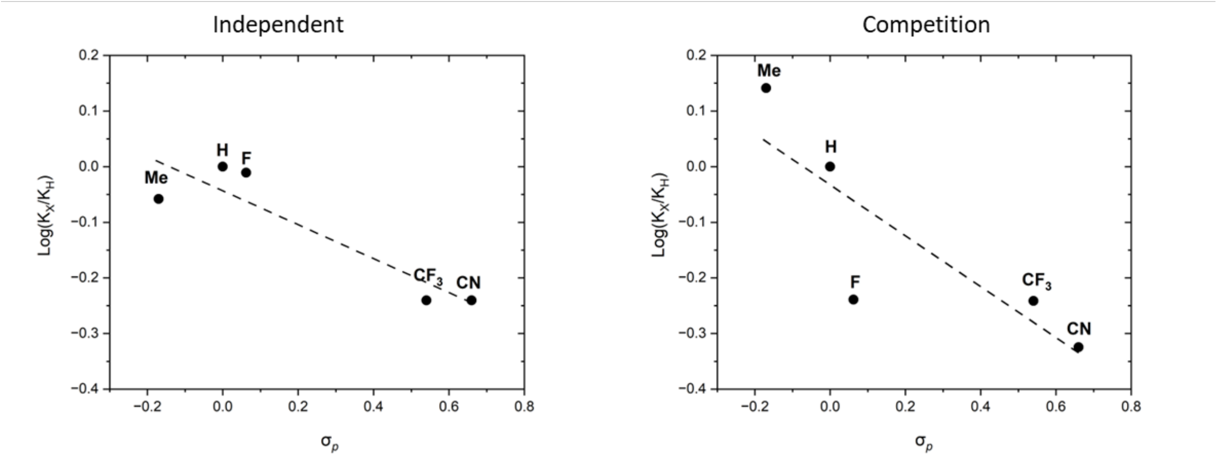

Figure S12. Hammett plots showing averaged log(K_H/K_X) vs. Hammett parameter for both independent (left) and competition (right) experiments.

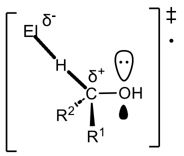

Figure S13. Representative loss of electron density as an electrophilic radical approaches the C(sp^3)–H adjacent to the hydroxyl group in the transition state.

Pinacol probe study

Anaerobic Oxidation of a Pinacol Probe.

A carbocation formation was tested for the anaerobic oxidation of alcohols by subjecting a synthesized pinacol probe, 2-phenylbutane-2,3-diol[57] to General Procedure A. In an oven-dried vial equipped with a stir bar was charged 2-phenylbutane-2,3-diol (133 mg, 1.00 equiv., 0.800 mmol), 3,5-Bis(trifluoromethyl)nitrobenzene (207 mg, 1.00 equiv., 0.800 mmol), and lithium acetate (13 mg, 0.25 equiv., 0.20 mmol) with MeCN/*t*BuOH (5:1, 0.7 M) under N_2. The reaction mixture was then irradiated under 390 nm light for 18 h. The reaction was monitored by GCMS, and after completion, the crude reaction mixture was concentrated down and the product isolated by column chromatography.

Results. Methyl shift of by the pinacol probe was not observed, indicating the reaction likely does not proceed through a carbocation intermediate.

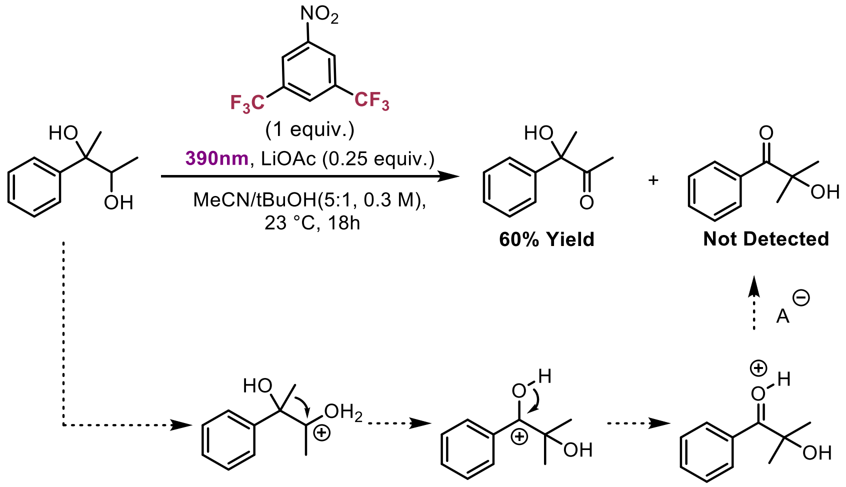

Figure S14. Pinacol Probe results after being subjected to Conditions A.

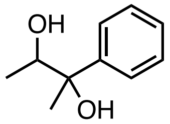

2-Phenylbutane-2,3-diol

To a solution of Acetoin (617 mg, 1.0 equiv., 7.00 mmol) in dry THF (5 mL) under argon atmosphere at -78 °C, a solution of phenyl lithium dibutyl ether (706 mg, 4.42 mL, 1.9 molar, 1.2 equiv., 8.40 mmol) was added dropwise. The resulting mixture was then stirred at room temperature for 40 minutes, quenched with water (10 mL), and extracted with EtOAc (3 × 10 mL). The combined organic layers were dried over Na_2SO_4, filtered, and concentrated under reduced pressure. The residue was purified by flash chromatography (25 % EtOAc/Hexane) to give a colorless oil, 531 mg (yield: 86%). All analytical data for the title compound was in accordance with literature data.[57]

^{1}H NMR (500 MHz, $CDCl_3$) (δ, ppm): 7.39 – 7.33 (m, 2H), 7.30 – 7.22 (m, 2H), 7.21 – 7.14 (m, 1H), 3.87 (q, *J* = 6.4 Hz, 1H), 2.61 (s, 1H), 2.10 (s, 1H), 1.39 (s, 3H), 1.03 (d, *J* = 6.5 Hz, 3H).
^{13}C NMR (126 MHz, $CDCl_3$) (δ, ppm): 145.9, 128.4, 127.3, 125.6, 76.9, 74.3, 23.3, 16.6.

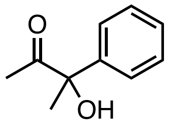

3-Hydroxy-3-phenylbutan-2-one
Following General Procedure A, prepared from 0.50 mmol of synthesized 2-phenylbutane-2,3-diol in 24 h. The title compound was isolated via flash chromatography (1 – 5% Acetone/Hexane). All analytical data for the title compound was in accordance with literature data.[58]

^{1}H NMR (400 MHz, CD_3CN) (δ, ppm): 7.50 – 7.42 (m, 2H), 7.42 – 7.33 (m, 2H), 7.35 – 7.26 (m, 1H), 4.34 (s, 1H), 2.04 (s, 3H), 1.65 (d, *J* = 1.1 Hz, 3H).
^{13}C NMR (101 MHz, CD_3CN) (δ, ppm): 210.9, 143.6, 129.4, 128.5, 126.5, 81.1, 25.5, 24.2.

Light on/off study

3,5-Bis-trifluoromethylnitrobenzene (69 mg, 0.25 mmol, 1.0 equiv.), 1-(4-methoxyphenyl)ethan-1-ol (76 mg, 0.50 mmol, 2.0 equiv.), and deuterated-MeCN (0.40 mL, 250 mM) were added under nitrogen to an NMR tube. Under a PhotoNMR setup, a fiber optic cable connected to a 395 nm lamp was added to the reaction, and ^{1}H NMR experiments were taken every 90 seconds. Product growth was monitored by the integration of the peak from 6.94-7.02 ppm; starting material conversion was measured from the integration of the peak from 6.80-6.92 ppm. An internal standard of CH_2Cl_2 was used. The lack of product formation in the dark during these studies is evidence against a radical chain mechanism.

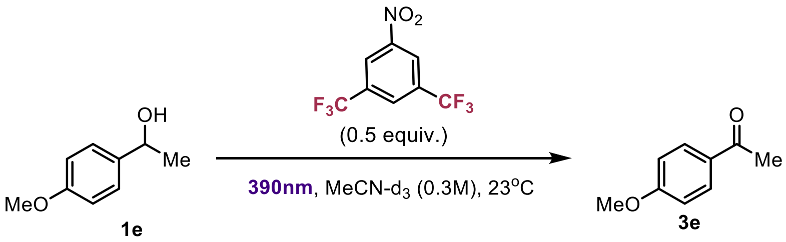

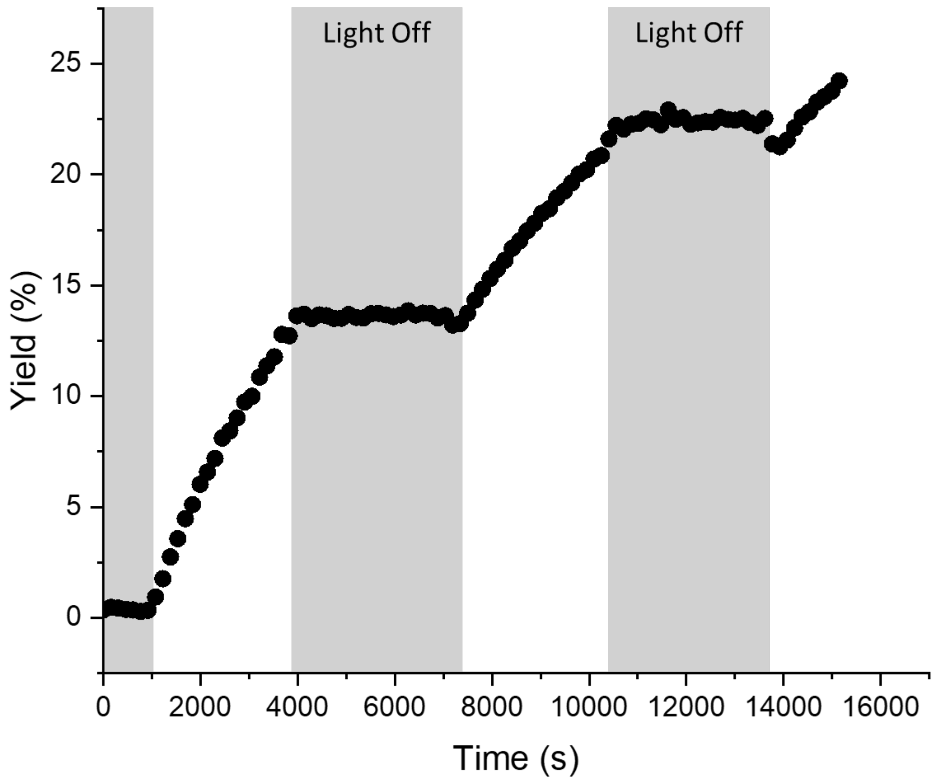

Figure S15. A Photo-NMR, light on/off study for the production of **3b** at 395 nm.

Discussion of mechanistic studies

We proposed that the oxidation reaction is initiated by hydrogen atom transfer (HAT) of the α-C(sp^3)–H bond with the photoexcited state of the nitroarene. To verify this, kinetic isotope effect (KIE) studies of **1d**/**1d-d₉** were conducted (Scheme S16A). KIE values of 1.77 and 1.54 were obtained for parallel and intermolecular studies, respectively. Next, a parallel study of the second HAT step was investigated with **1d**/**1d-d₁** and resulted in a secondary KIE of 1.2 (Scheme S16B). These studies suggest that the first HAT participates in the rate-limiting step of the transformation. To support the radical nature of the transformation, radical clock **18** was subjected to the reaction conditions (Scheme S16C). Although low conversion of 23% was observed, the oxidation product with the cyclopropane moiety preserved was not observed **19**. Only the ring opening over-oxidation products **19** and **21** were detected. The presence of **19** indicates that radical recombination of the formed alkyl radical and the formed *N*-hydroxy-*N*-phenylhydroxylamine radical via oxygen atom transfer (OAT) from the nitroarene could be possible in the oxidation reaction. To distinguish if a recombination event is operative, ^{18}O-labeled substrate **1d-^{18}O** was tested (Scheme S16D). Upon exposure to the reaction conditions, the ^{18}O-incorporated retention product **3d-^{18}O** was obtained with no scrambling product detected. Hence, supporting that the oxidation of C(sp^3)–heteroatom systems do not occur through radical recombination but rather a double HAT event.

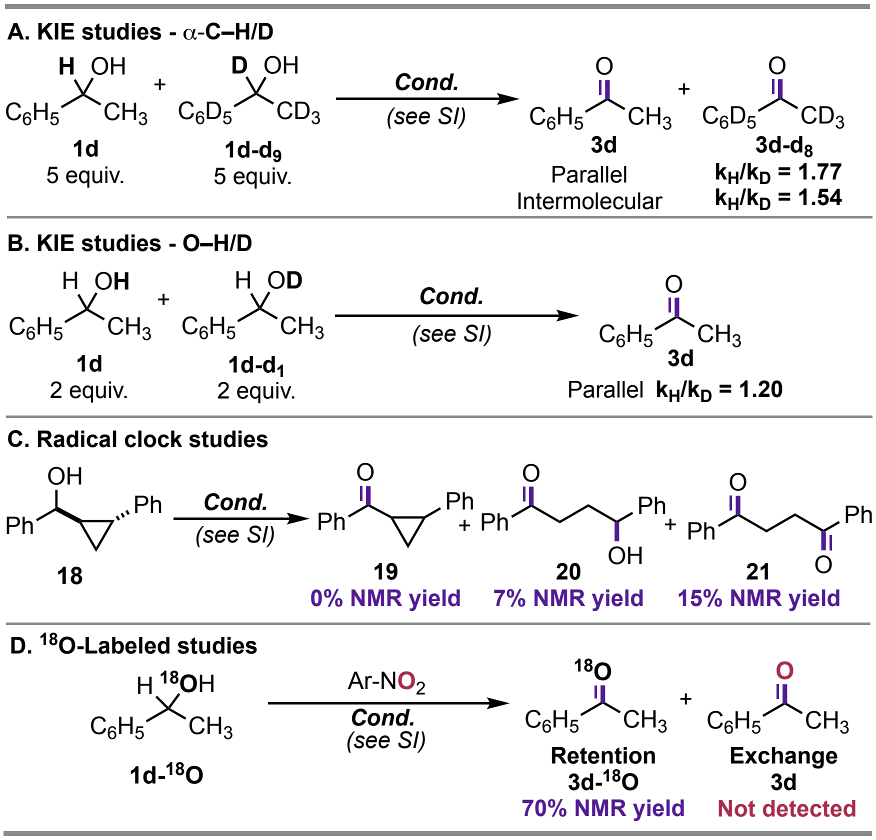

Figure S16. Summary of mechanistic studies

NMR spectra

^{1}H NMR (500 MHz, CDCl$_3$) of 1-phenylpentan-1-one (3a)

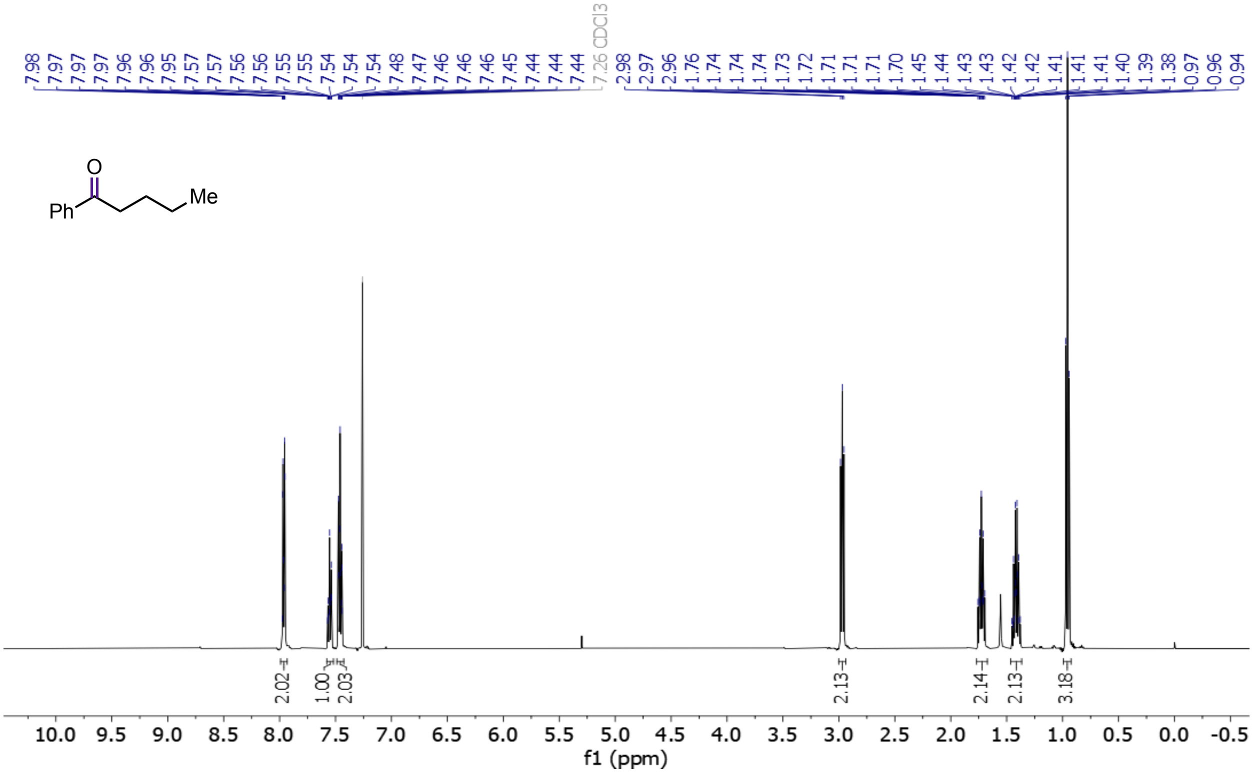

^{13}C NMR (126 MHz, CDCl$_3$) of 1-phenylpentan-1-one (3a)

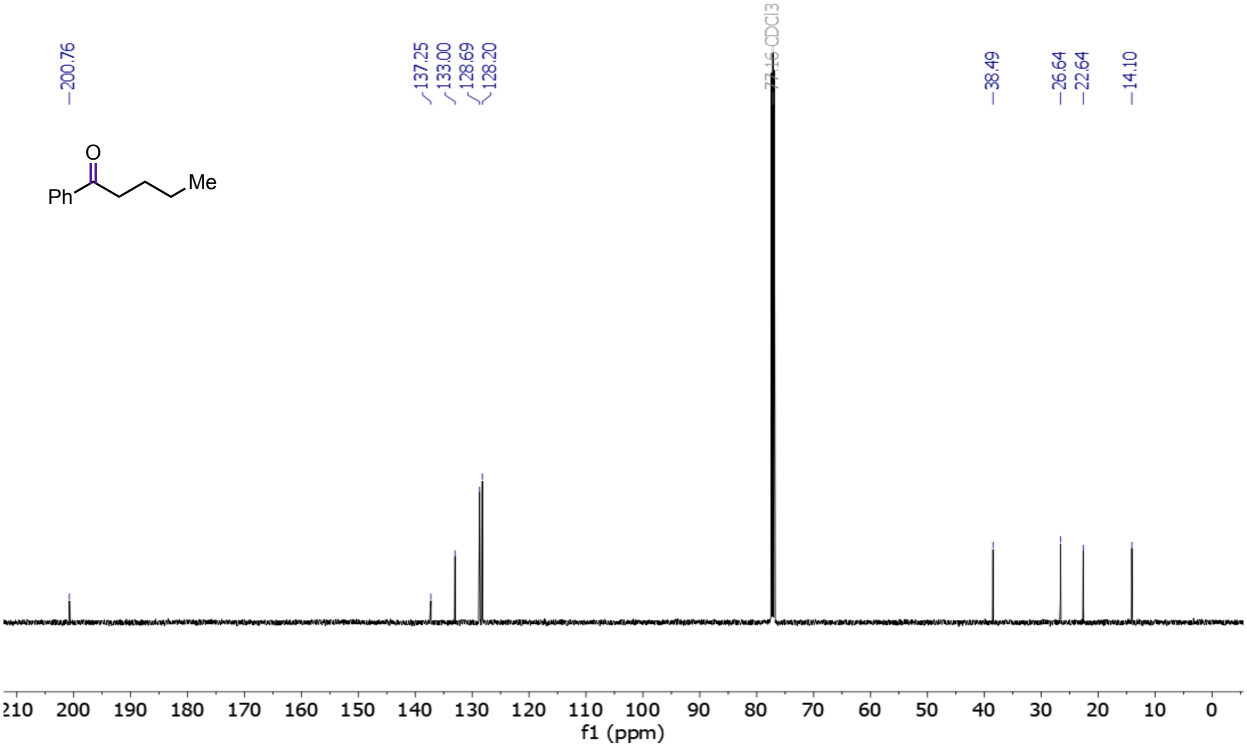

^{1}H NMR (400 MHz, $CDCl_3$) of 1-(4-methoxyphenyl)ethan-1-one (3b)

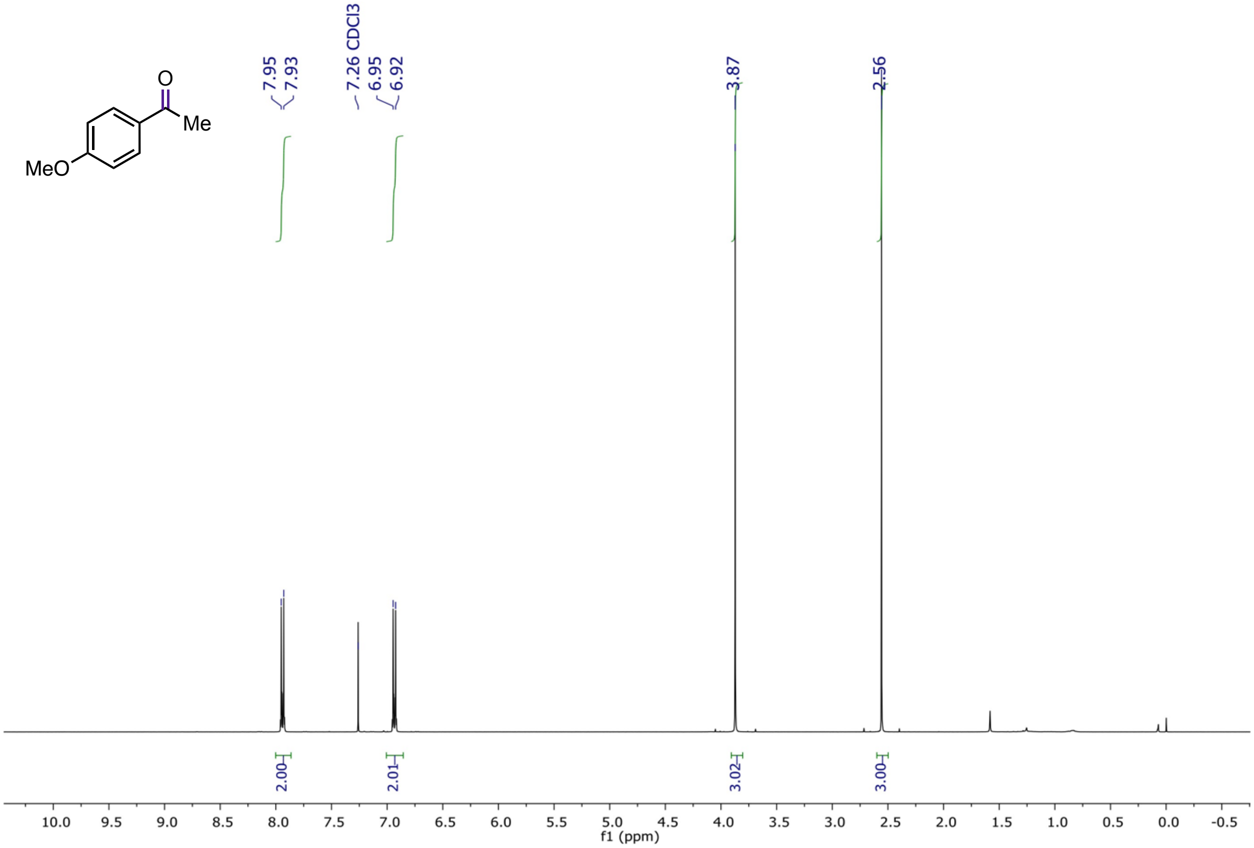

^{13}C NMR (101 MHz, $CDCl_3$) of 1-(4-methoxyphenyl)ethan-1-one (3b)

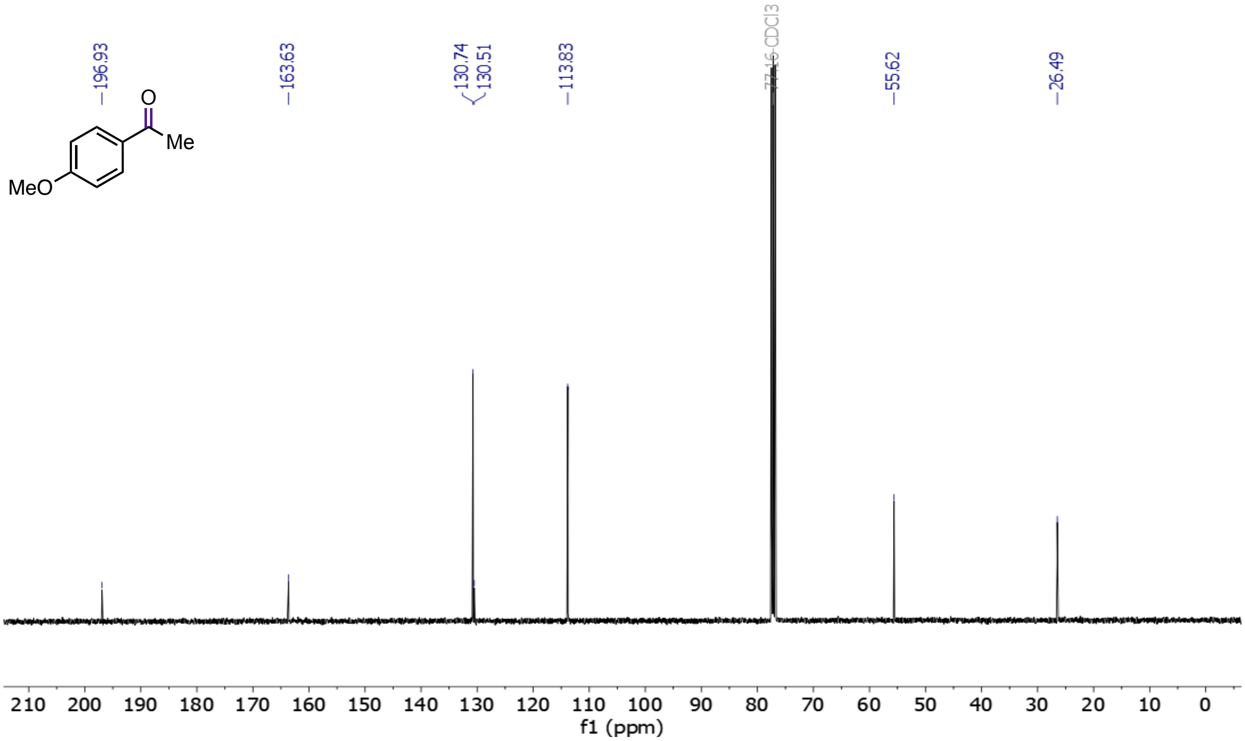

^{1}H NMR (500 MHz, CD_3CN) of 1-(*p*-tolyl)ethan-1-one (3c)

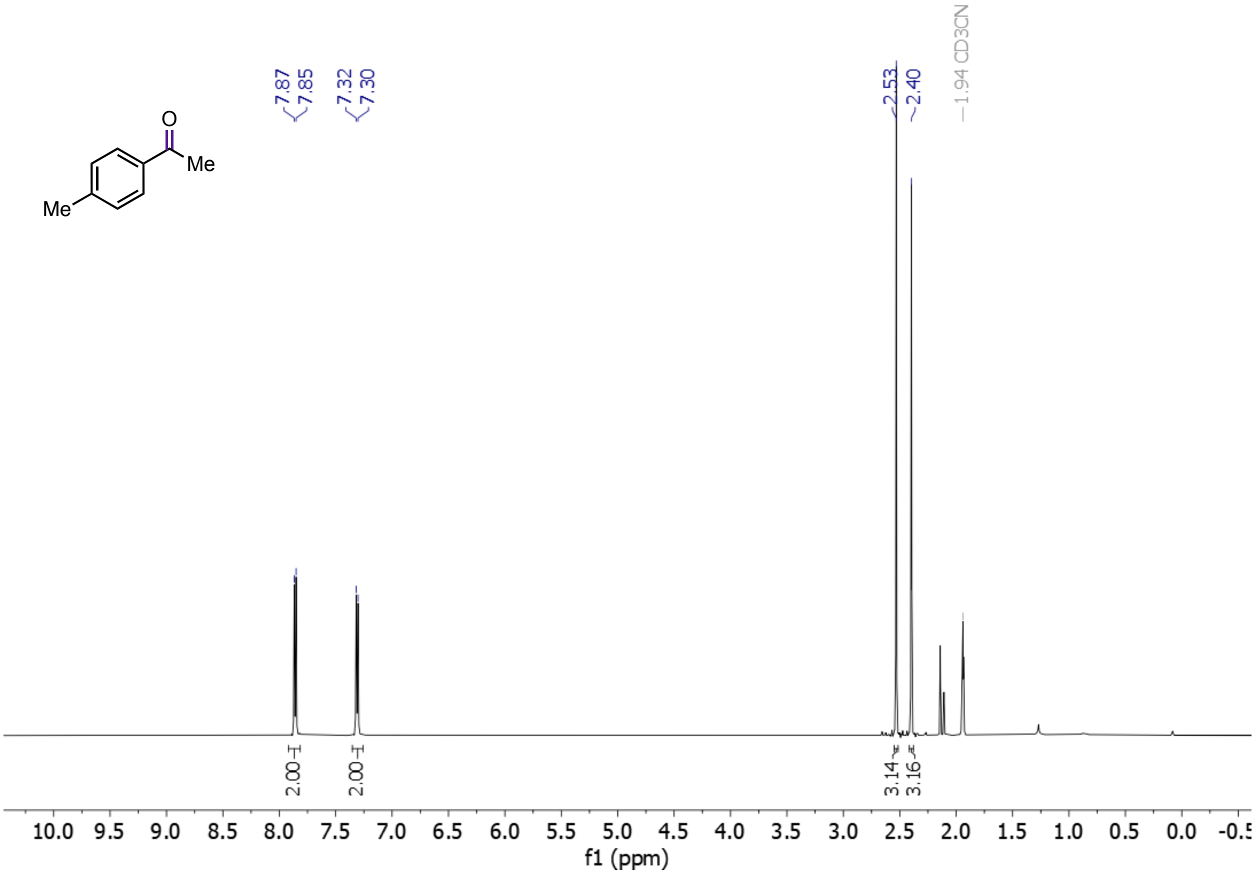

^{13}C NMR (126 MHz, CD_3CN) of 1-(*p*-tolyl)ethan-1-one (3c)

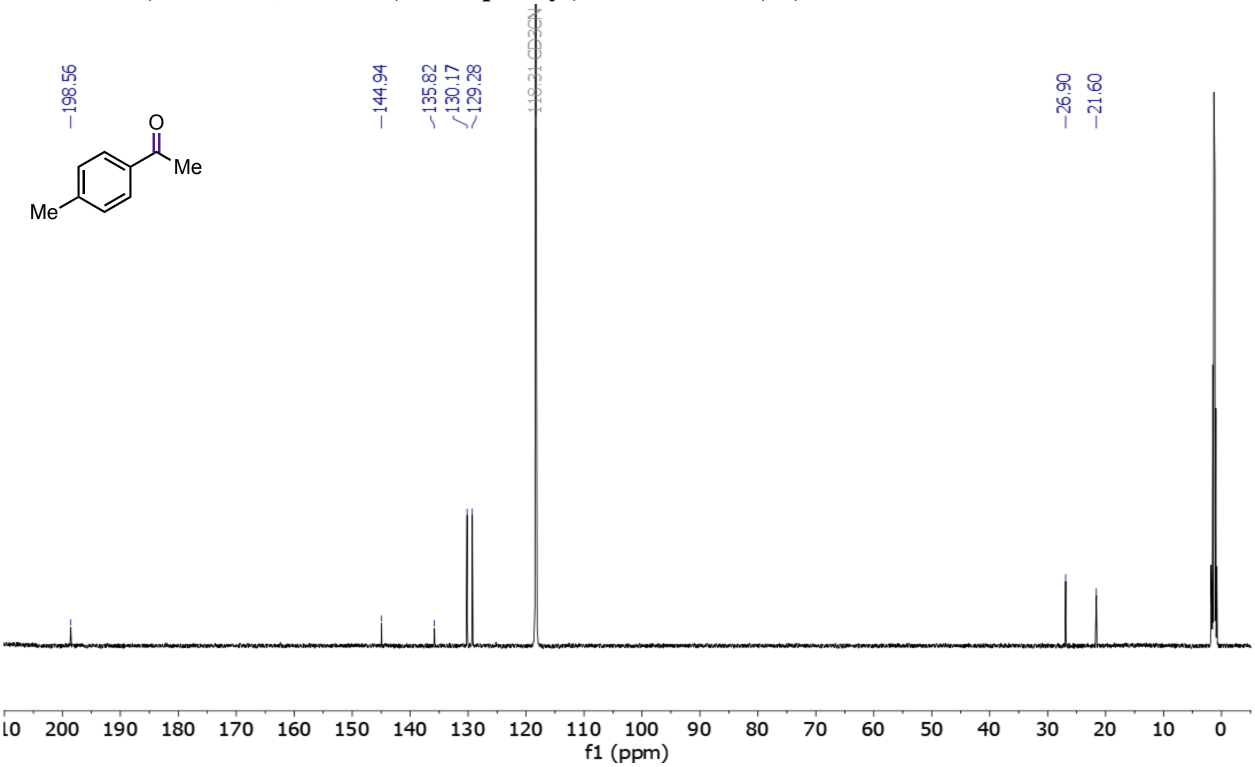

^{1}H NMR (500 MHz, $CDCl_3$) of Acetophenone (3d)

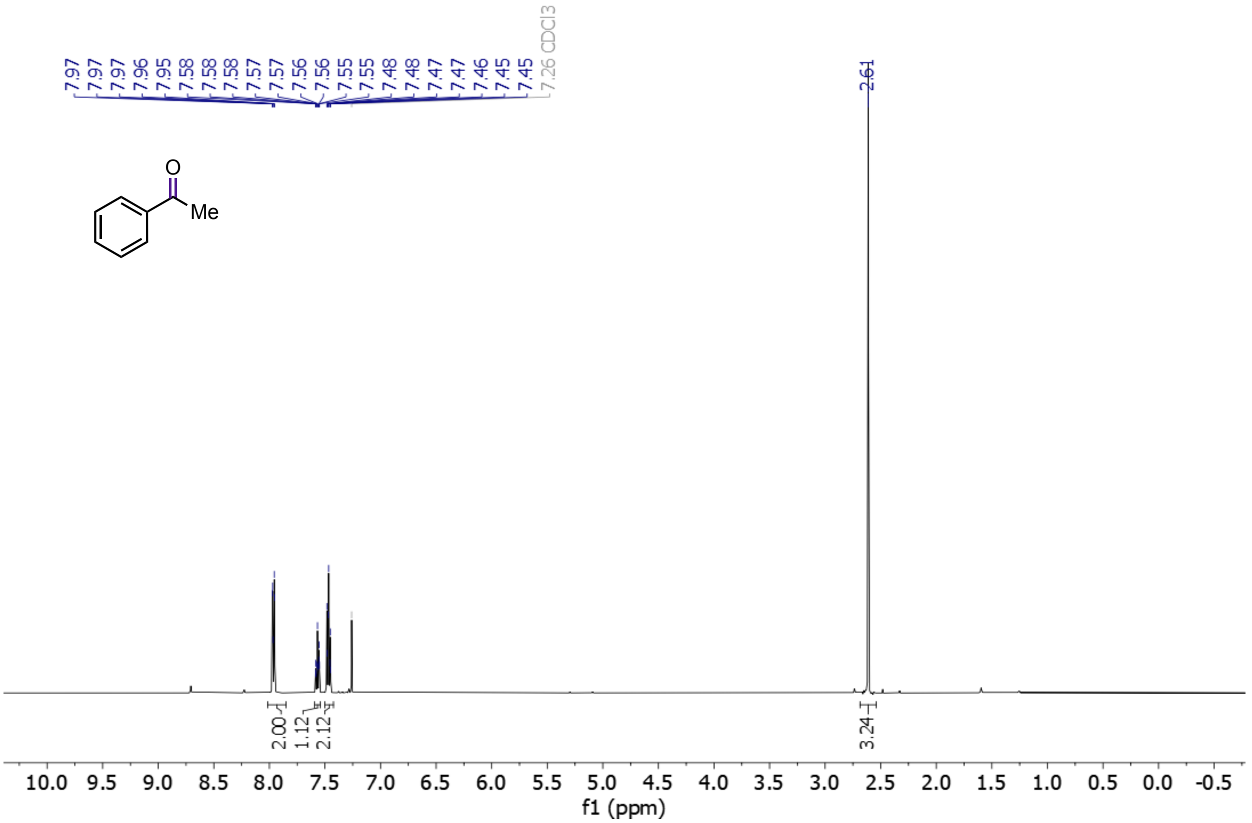

^{13}C NMR (126 MHz, $CDCl_3$) of Acetophenone (3d)

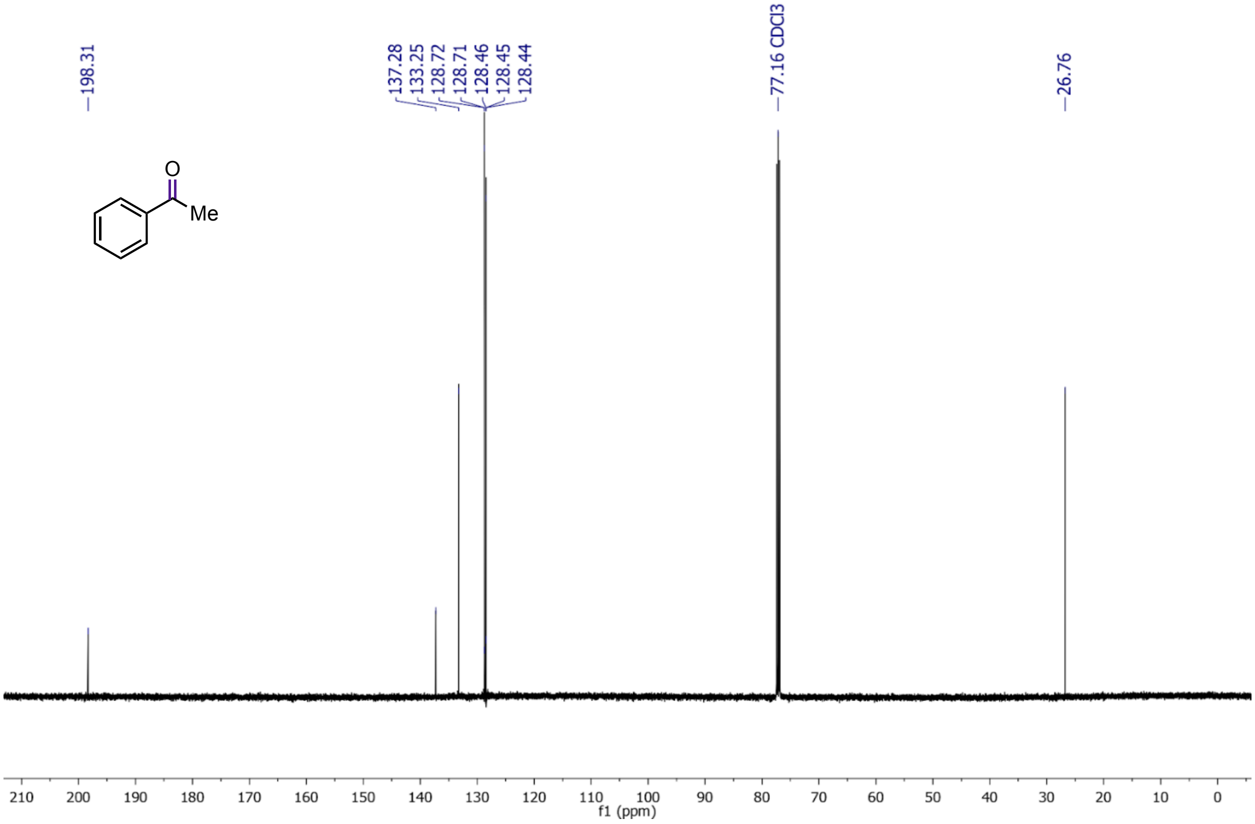

^{1}H NMR (400 MHz, $CDCl_3$) of 1-(4-fluorophenyl)ethan-1-one (3e)

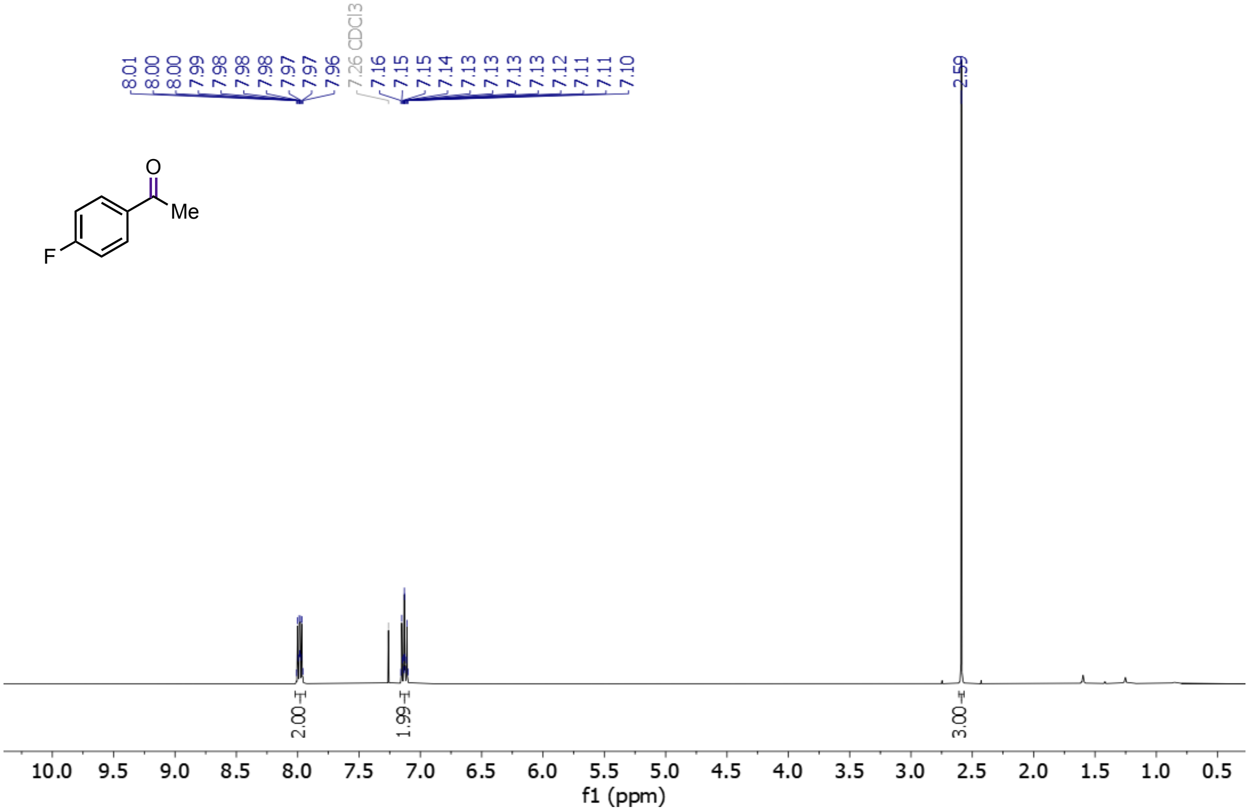

^{13}C NMR (101 MHz, $CDCl_3$) of 1-(4-fluorophenyl)ethan-1-one (3e)

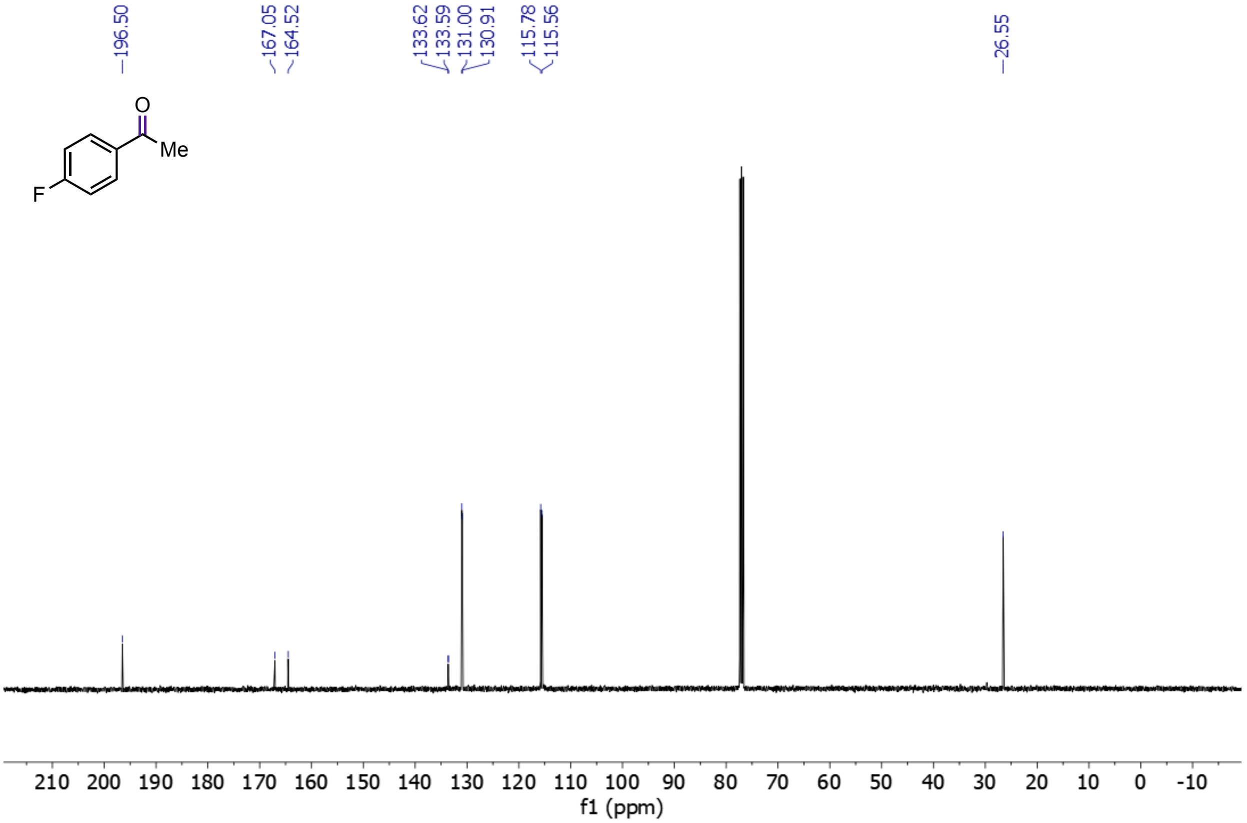

^{19}F NMR (377 MHz, $CDCl_3$) of 1-(4-fluorophenyl)ethan-1-one (3e)

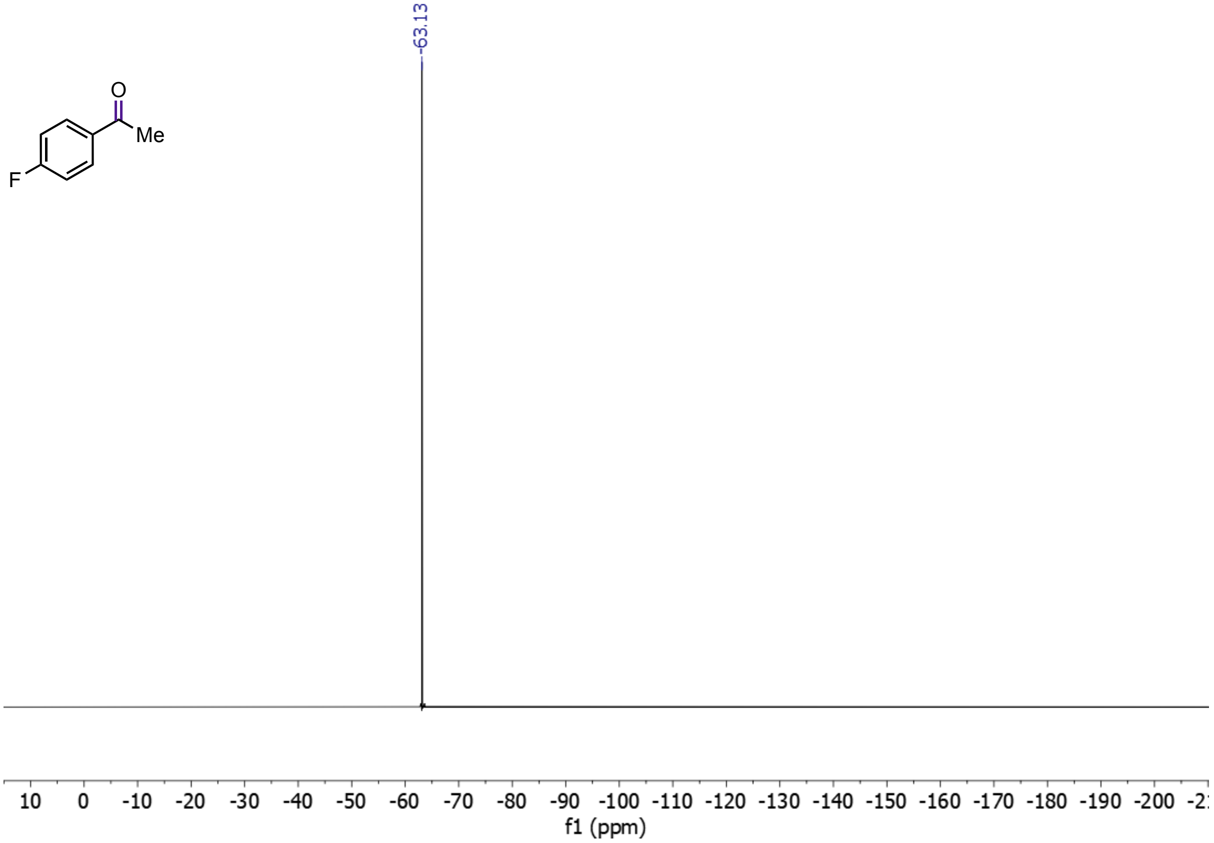

^{1}H NMR (400 MHz, $CDCl_3$) of 1-(4-(trifluoromethyl)phenyl)ethan-1-one (3f)

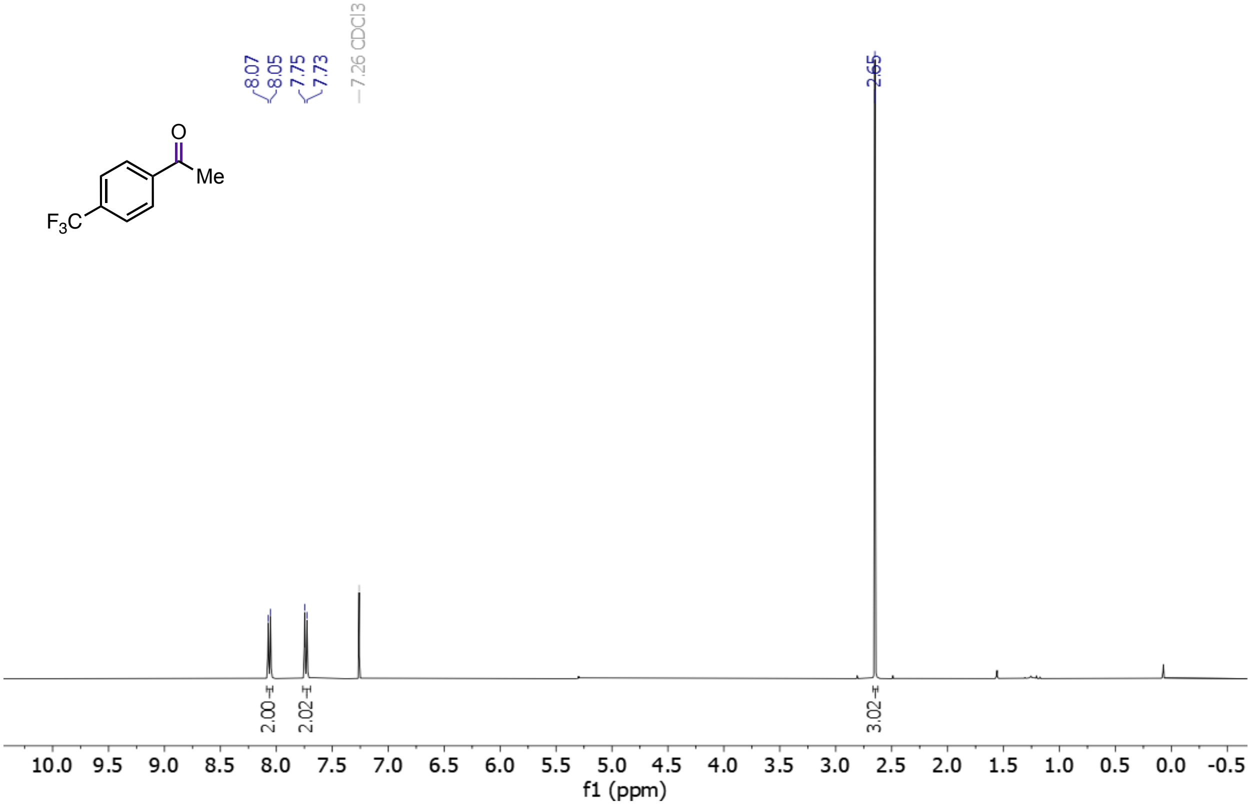

^{13}C NMR (126 MHz, $CDCl_3$) of 1-(4-(trifluoromethyl)phenyl)ethan-1-one (3f)

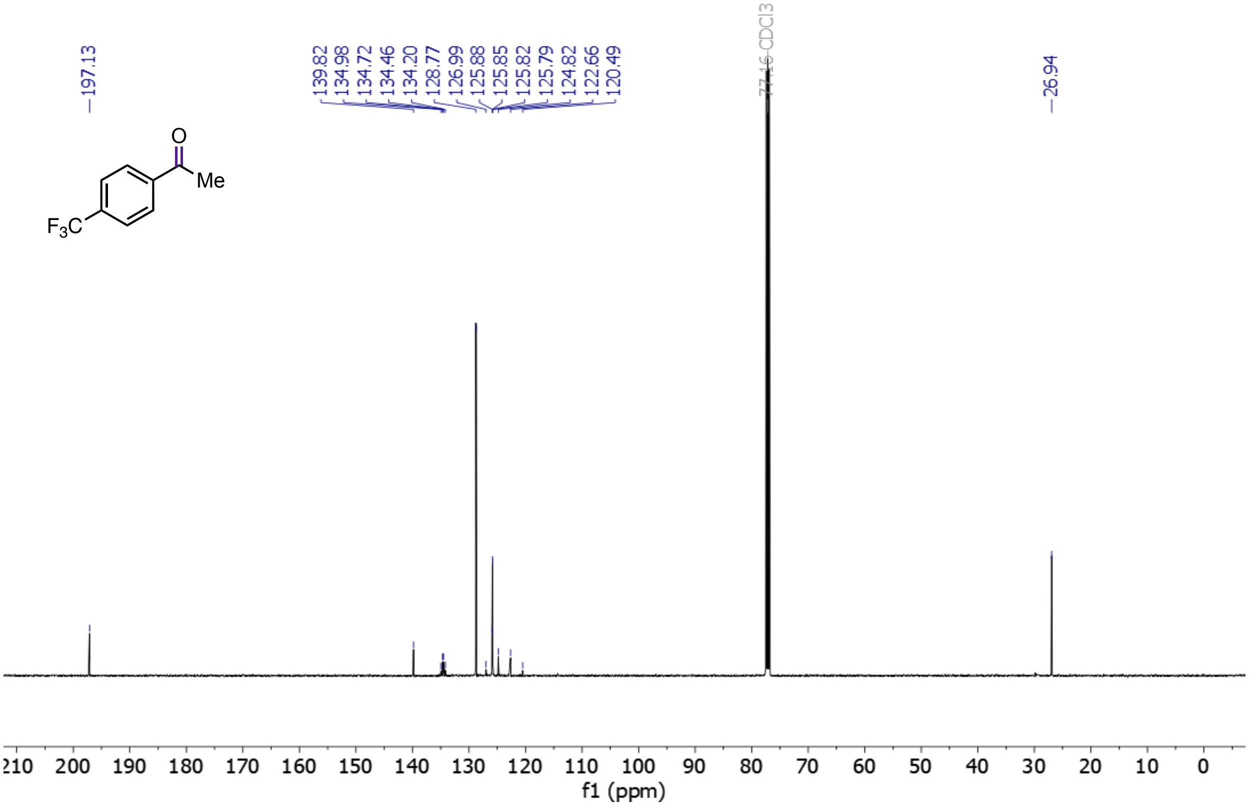

^{1}H NMR (500 MHz, $CDCl_3$) of 4-acetylbenzonitrile (3g)

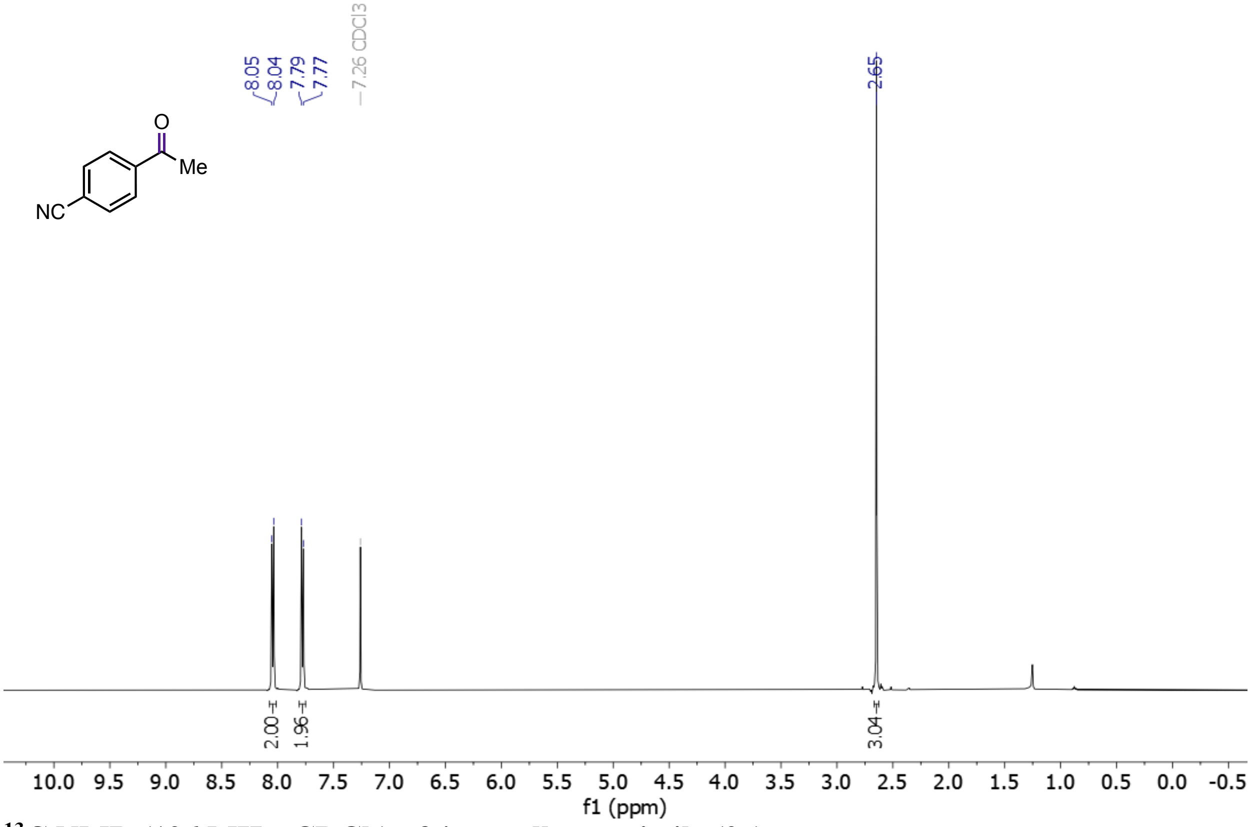

^{13}C NMR (126 MHz, $CDCl_3$) of 4-acetylbenzonitrile (3g)

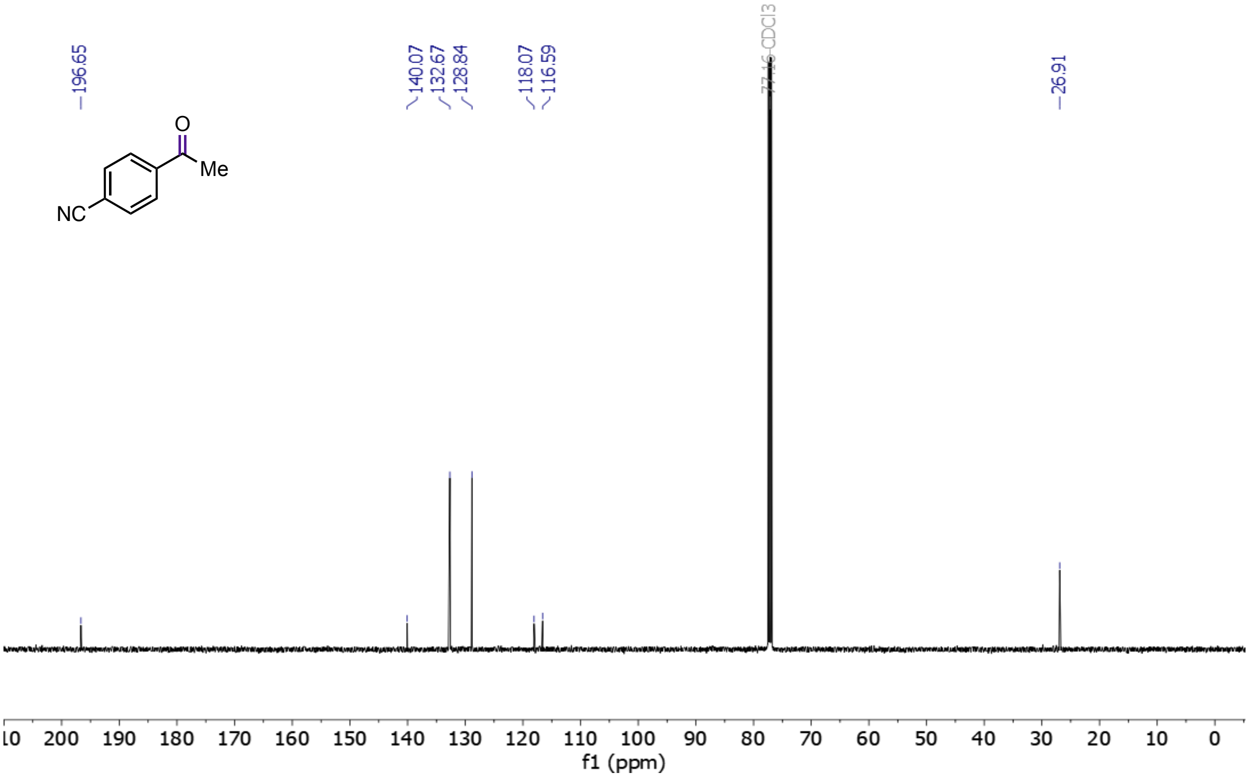

^{1}H NMR (500 MHz, $CDCl_3$) of 1-(*m*-tolyl)ethan-1-one (3h)

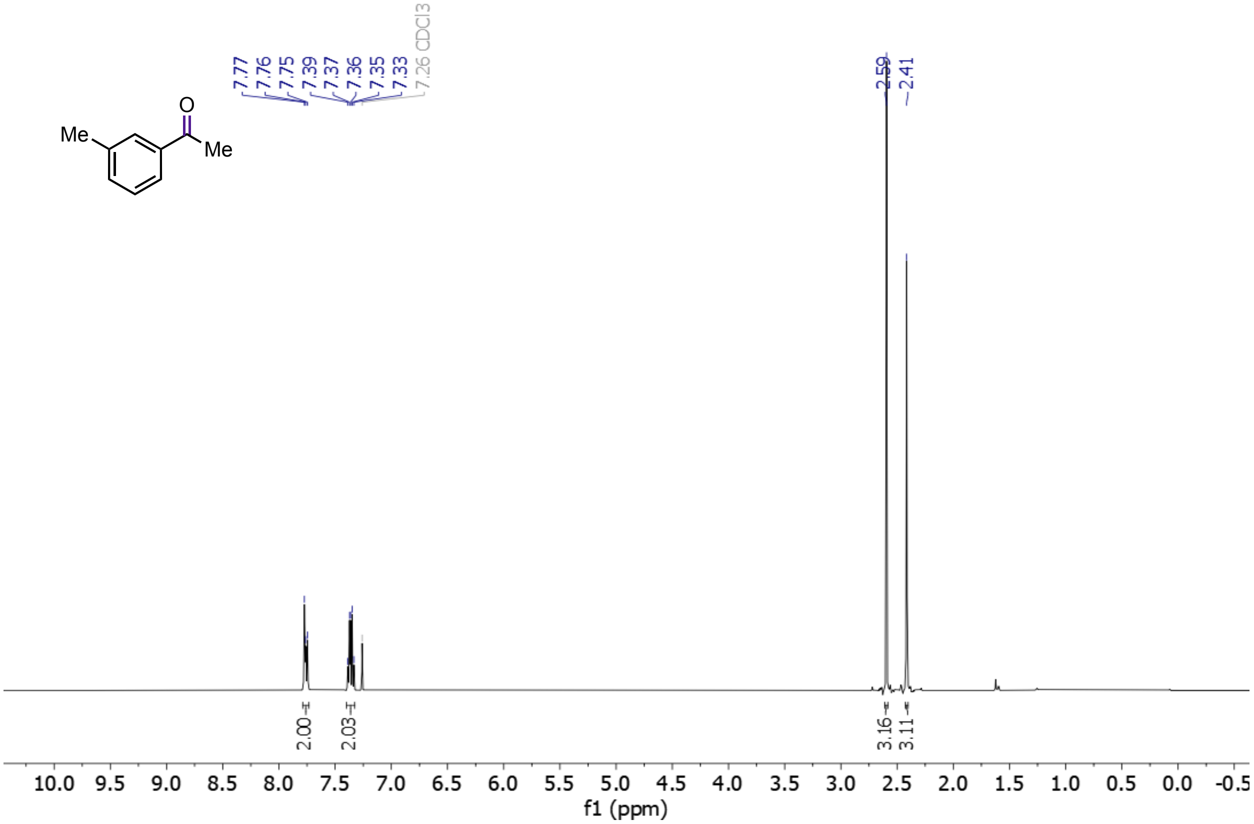

^{13}C NMR (126 MHz, $CDCl_3$) of 1-(*m*-tolyl)ethan-1-one (3h)

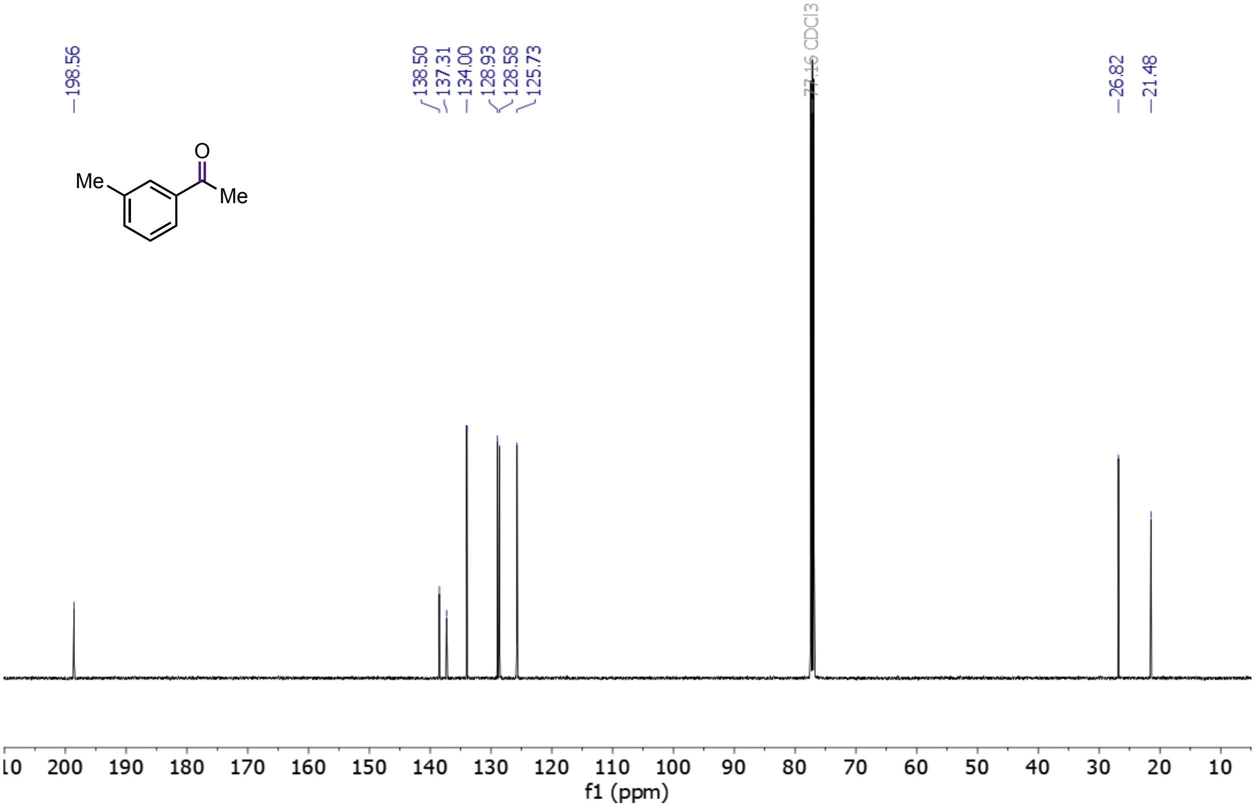

^{1}H NMR (500 MHz, $CDCl_3$) of 1-(3-bromophenyl)ethan-1-one (3i)

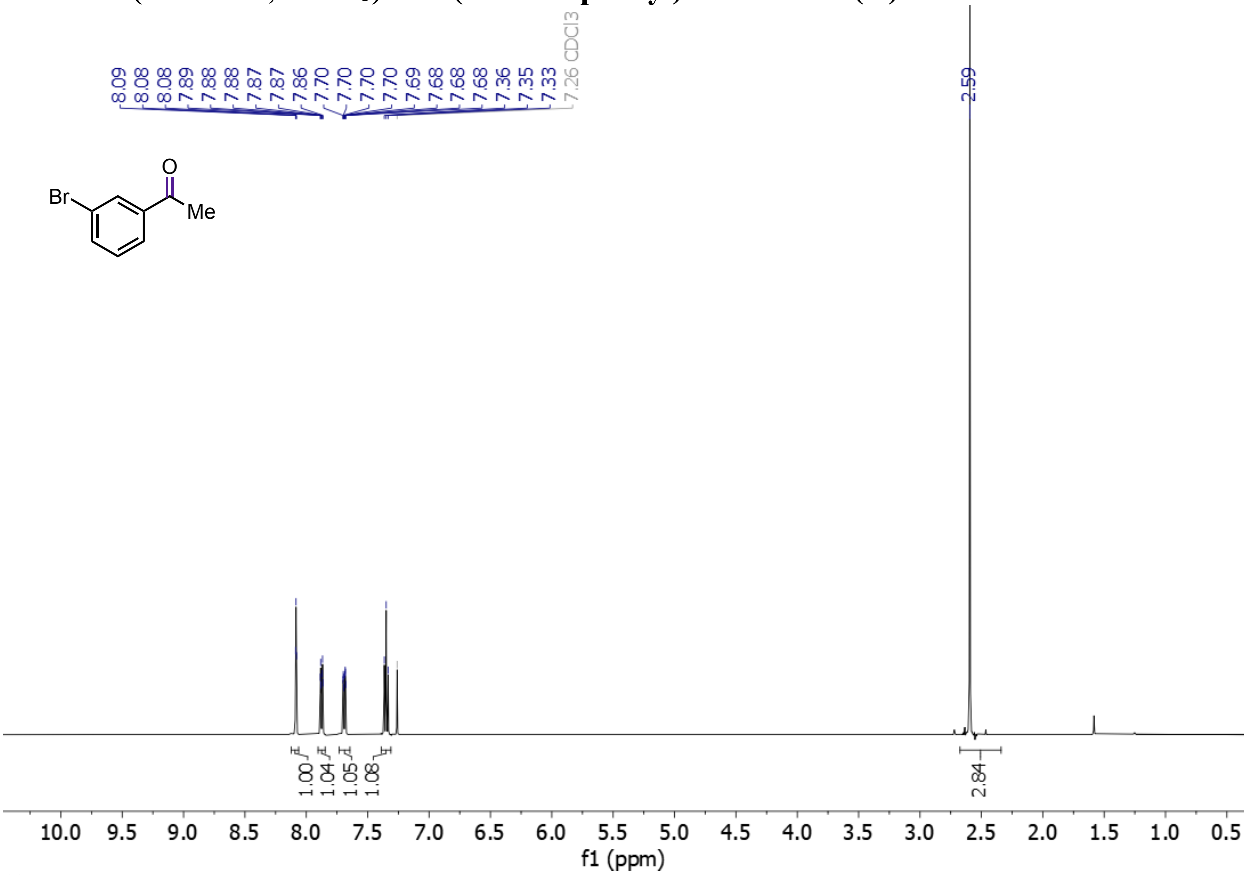

^{13}C NMR (126 MHz, $CDCl_3$) of 1-(3-bromophenyl)ethan-1-one (3i)

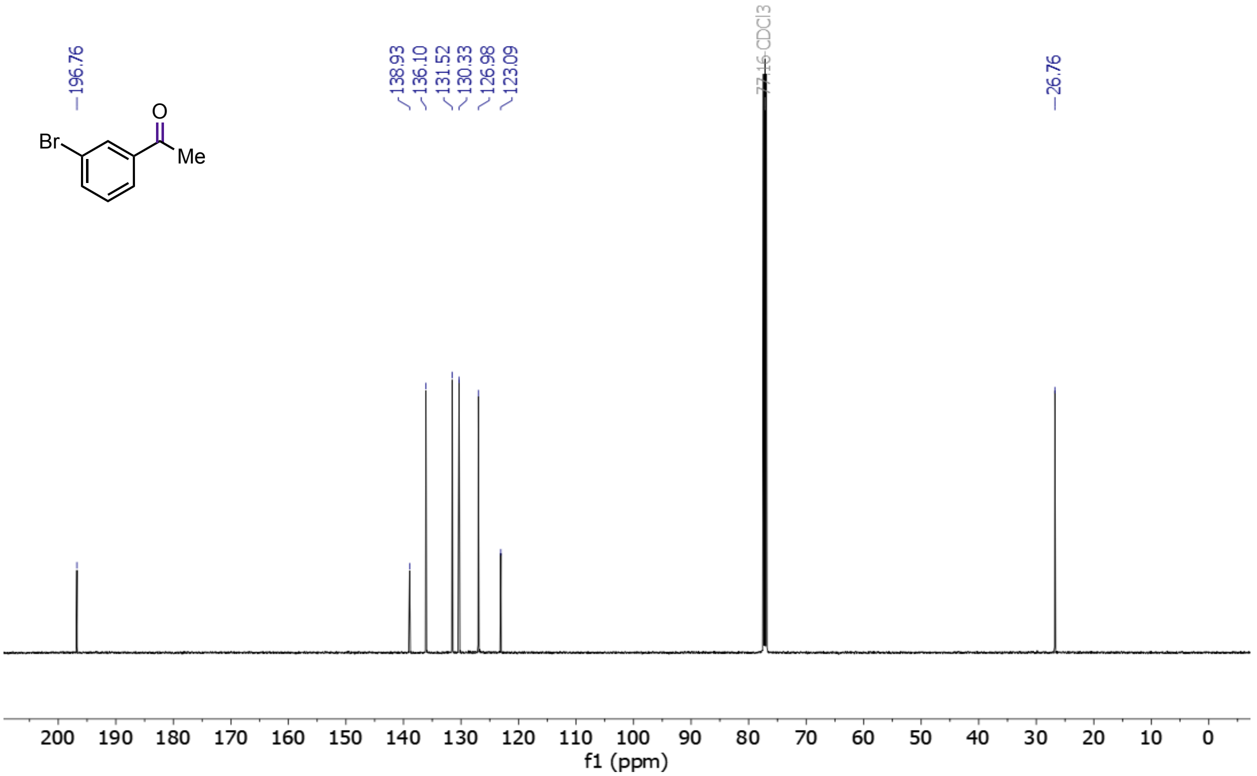

^{1}H NMR (500 MHz, $CDCl_3$) of 1-(2-bromophenyl)ethan-1-one (3k)

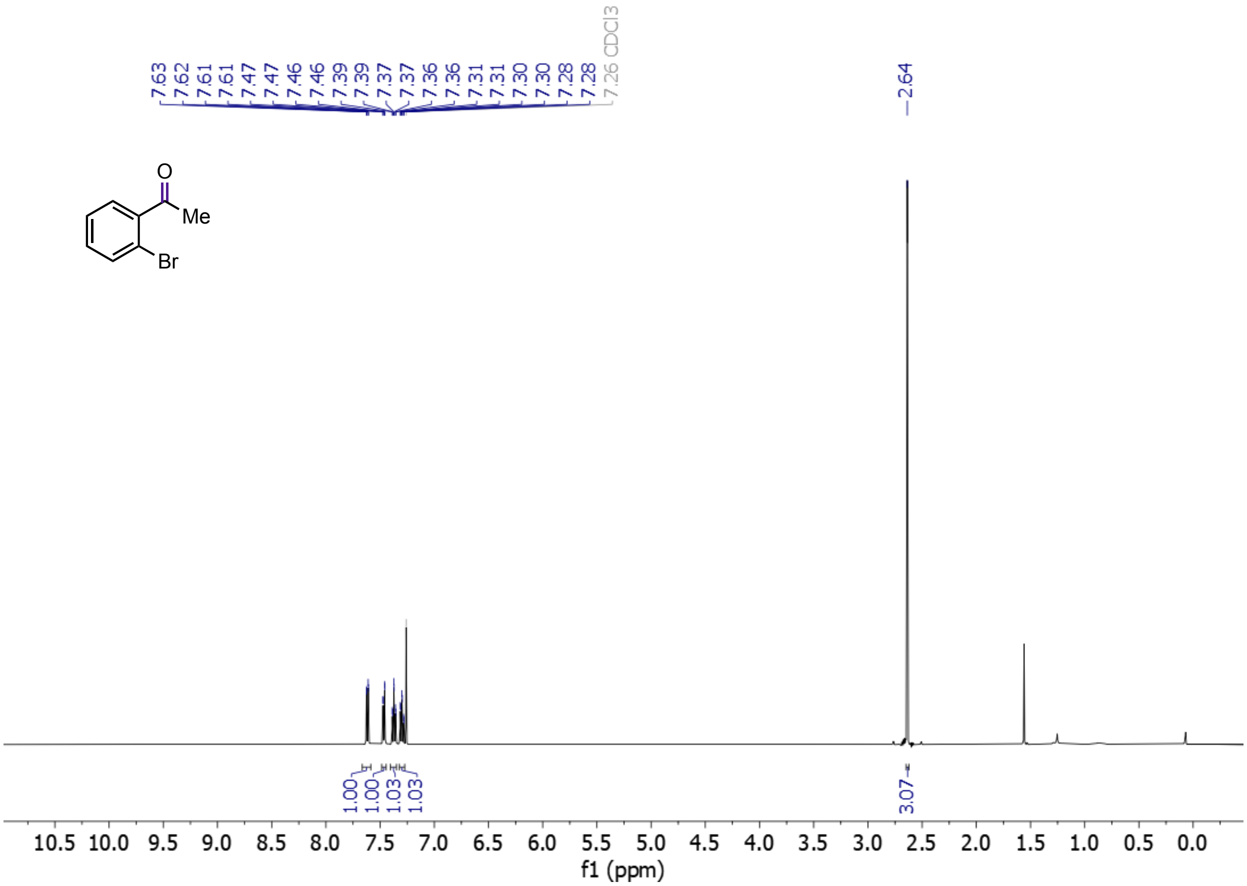

^{13}C NMR (126 MHz, $CDCl_3$) of 1-(2-bromophenyl)ethan-1-one (3k)

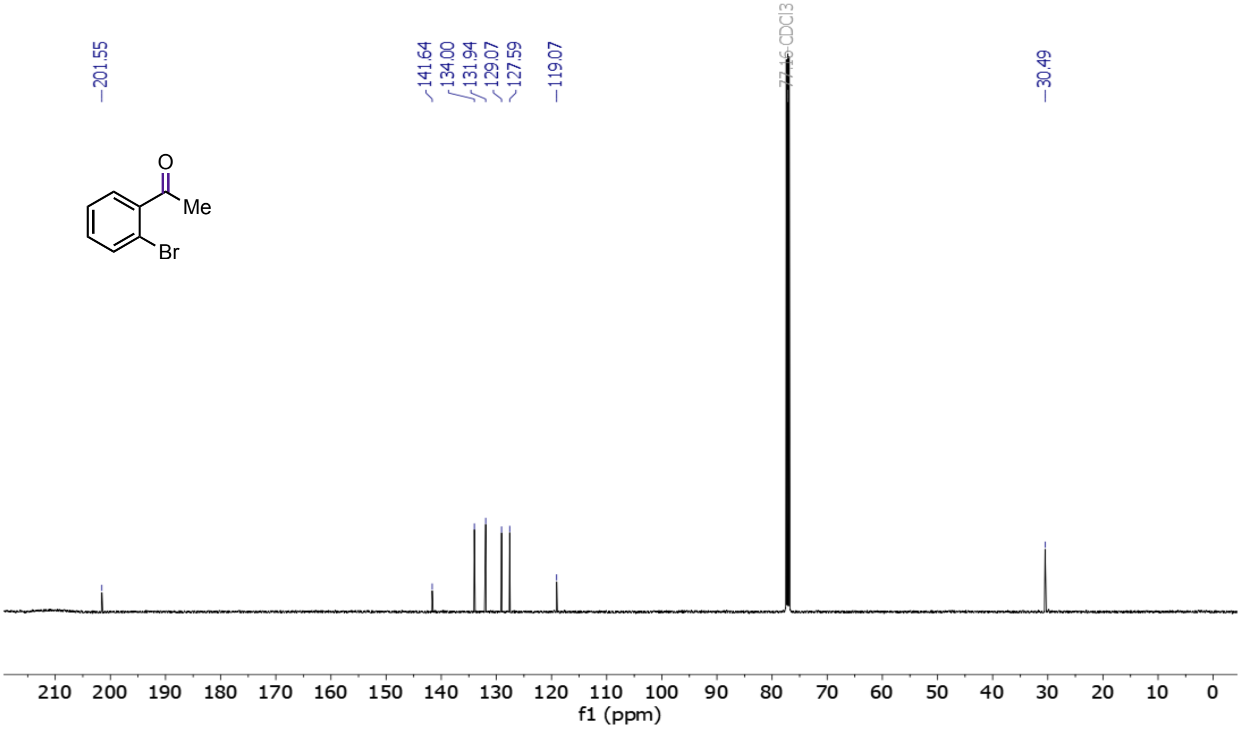

^{1}H NMR (400 MHz, $CDCl_3$) of 2,3-dihydro-1*H*-inden-1-one (3l)

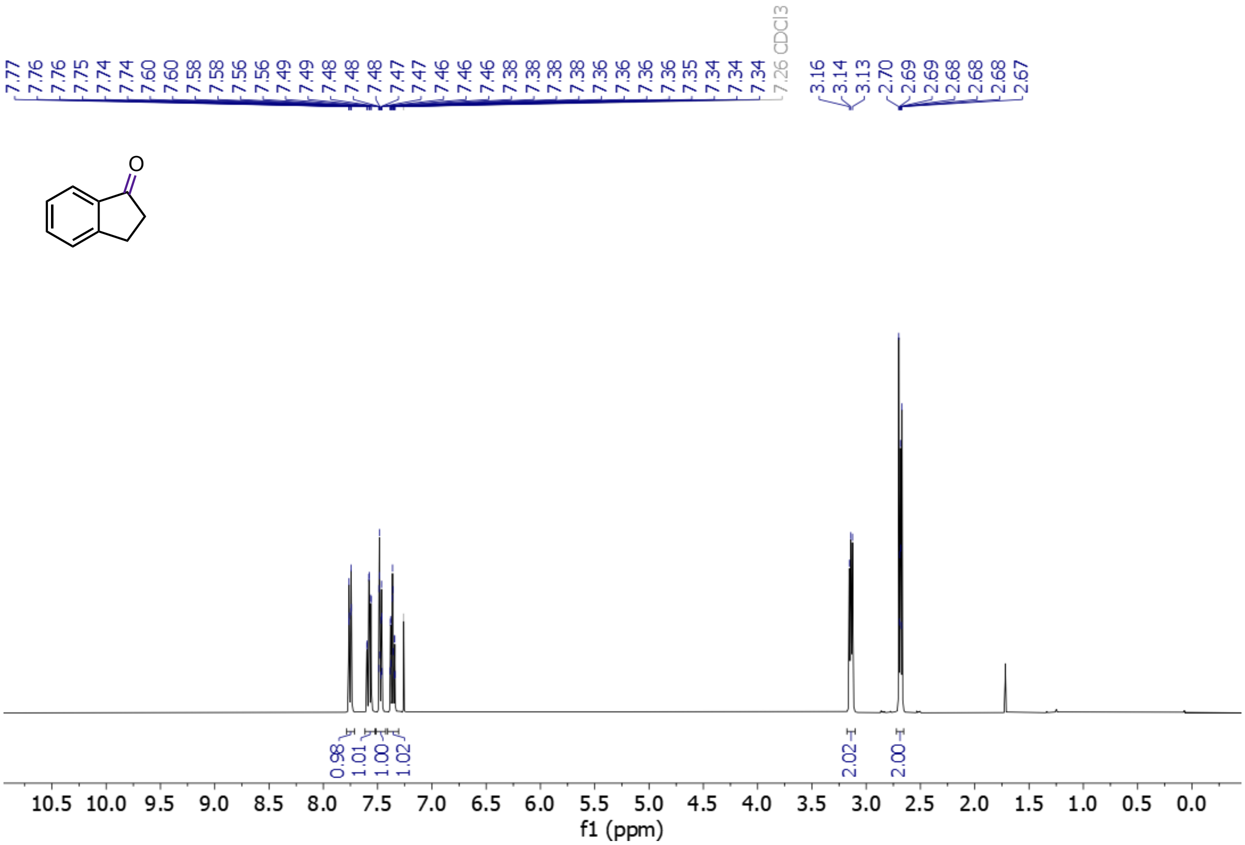

^{13}C NMR (101 MHz, $CDCl_3$) of 2,3-dihydro-1*H*-inden-1-one (3l)

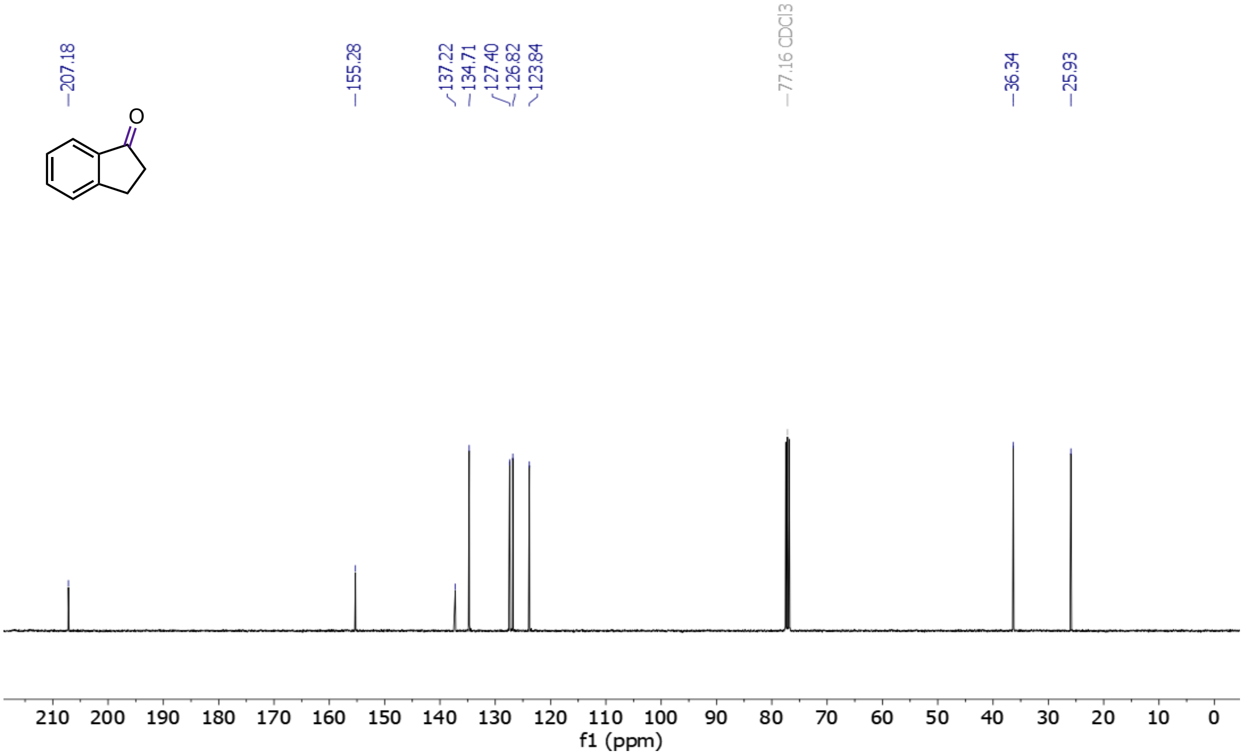

^{1}H NMR (500 MHz, $CDCl_3$) of 3,4-dihydronaphthalen-1(2*H*)-one (3m)

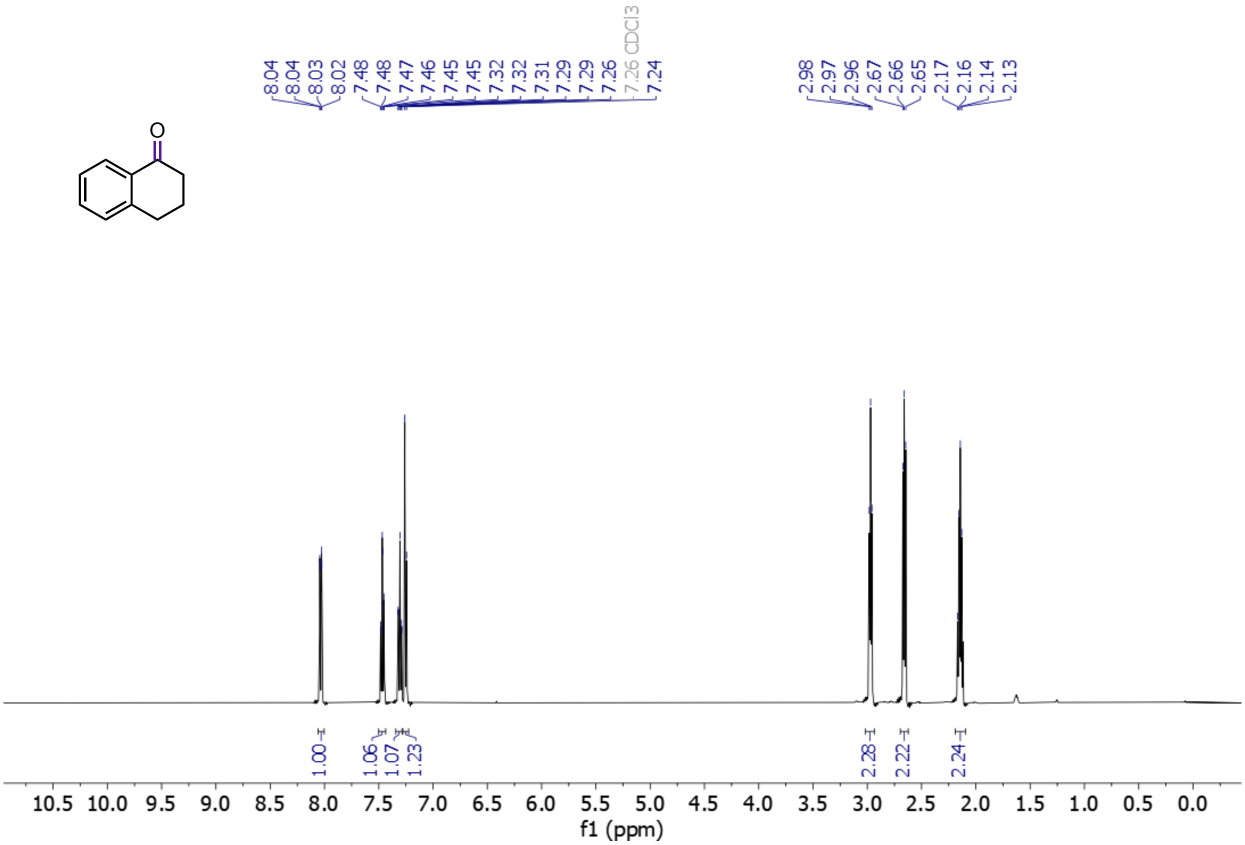

^{13}C NMR (126 MHz, $CDCl_3$) of 3,4-dihydronaphthalen-1(2*H*)-one (3m)

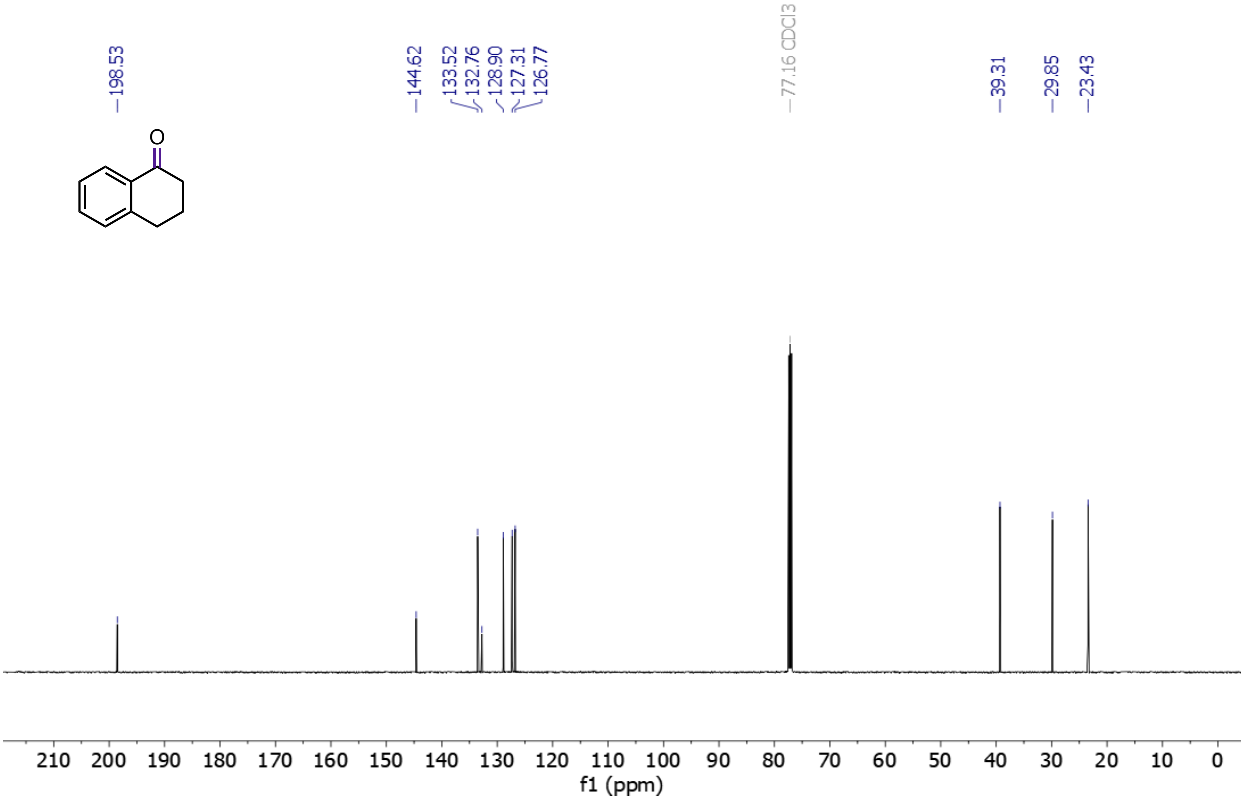

^{1}H NMR (400 MHz, $CDCl_3$) of cyclopropyl(phenyl)methanone (3n)

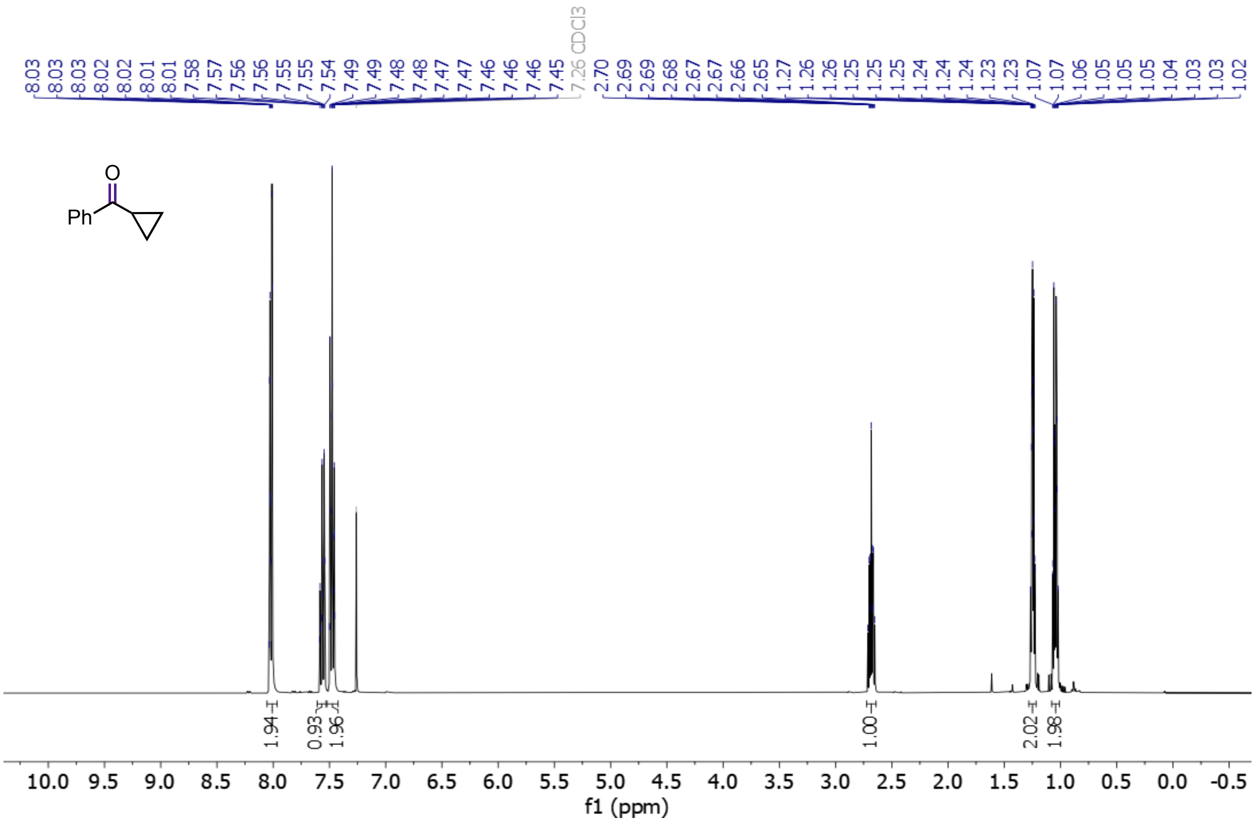

^{13}C NMR (101 MHz, $CDCl_3$) of cyclopropyl(phenyl)methanone (3n)

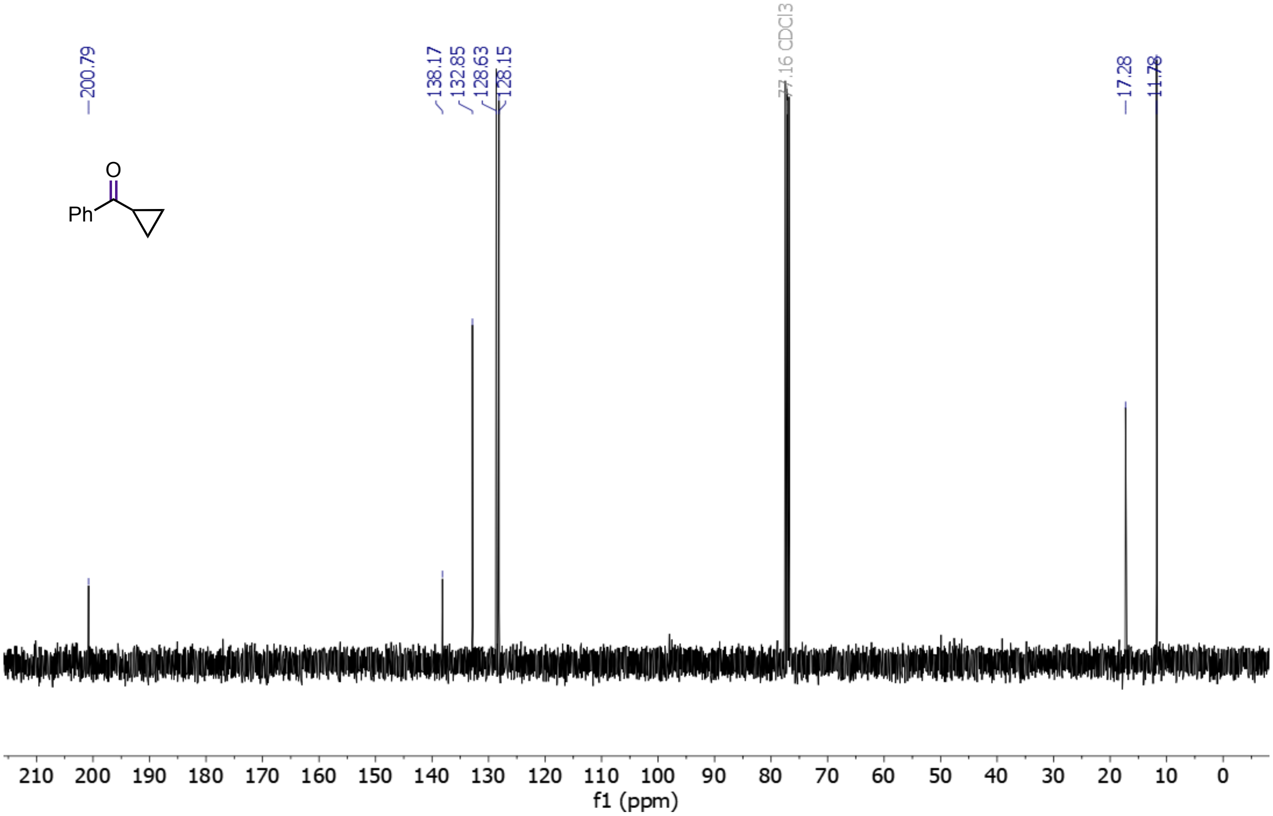

^{1}H NMR (400 MHz, $CDCl_3$) Acenaphthylen-1(*2H*)-one (3o)

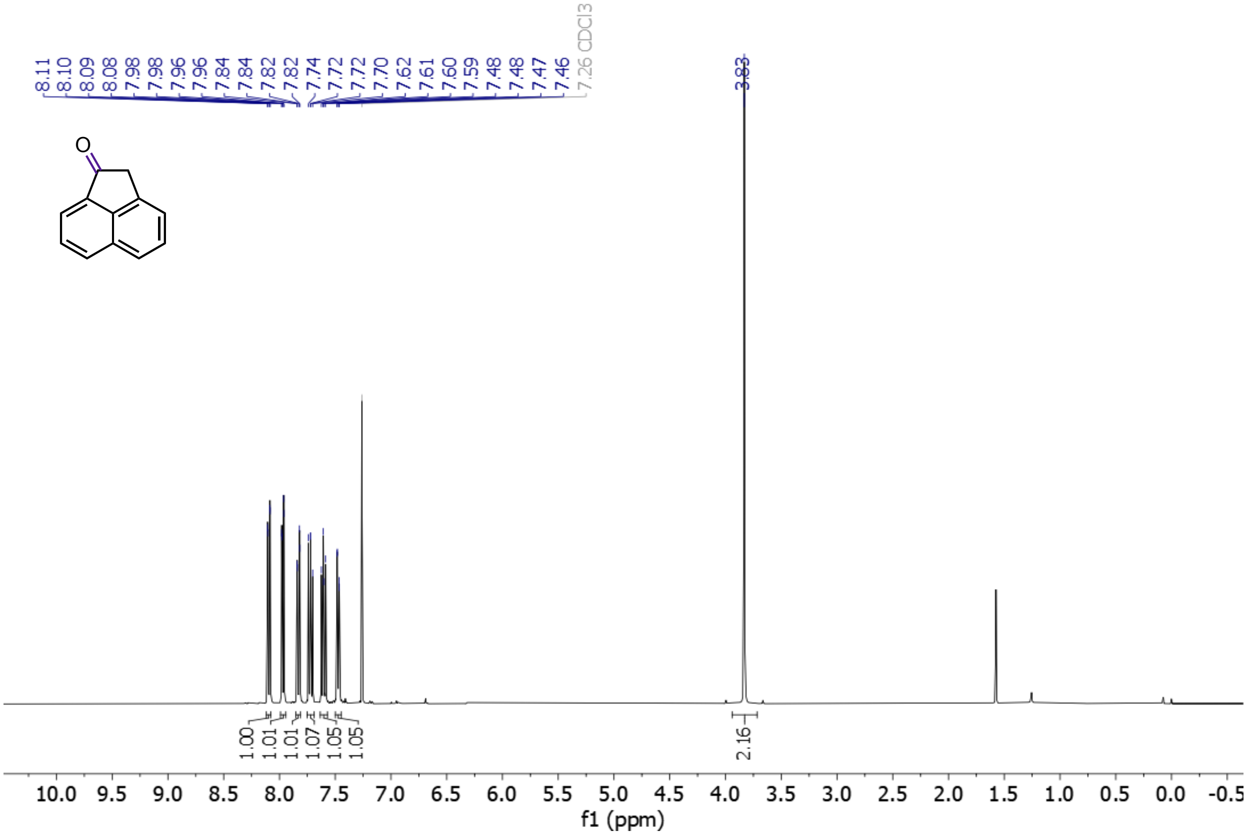

^{13}C NMR (126 MHz, $CDCl_3$) Acenaphthylen-1(*2H*)-one (3o)

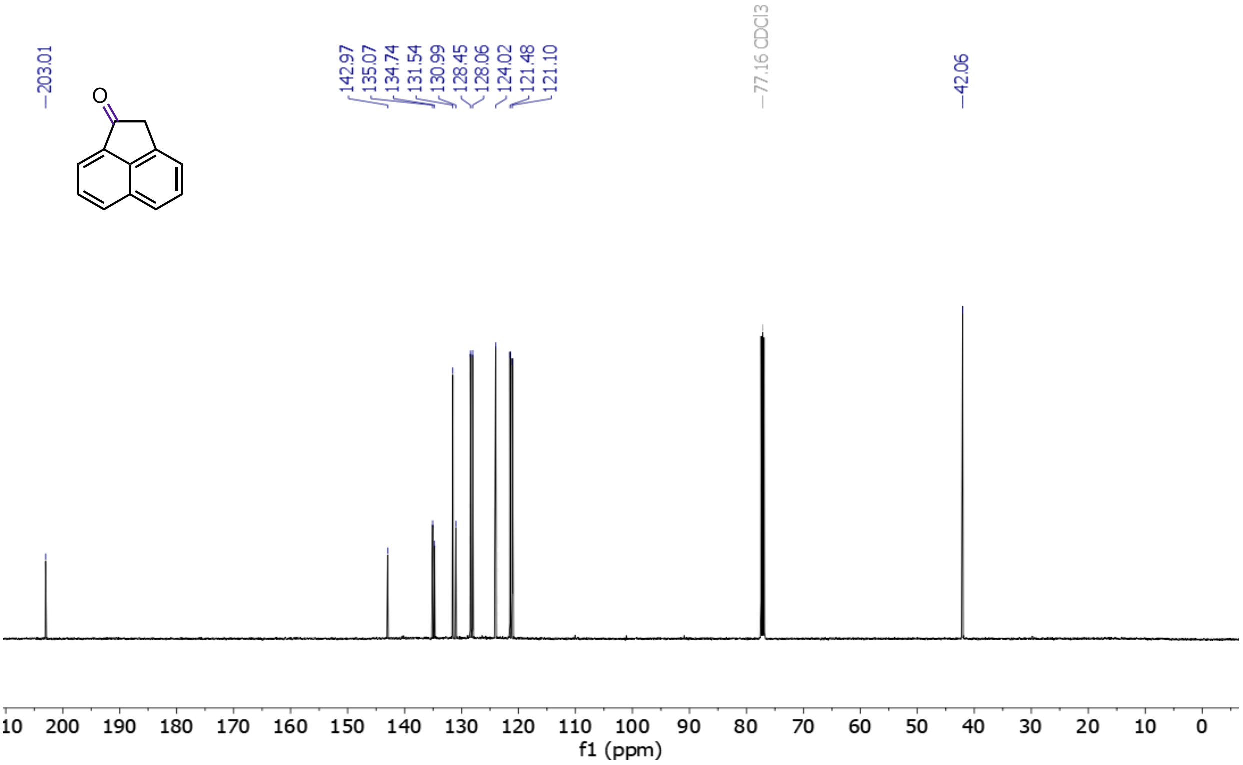

^{1}H NMR (400 MHz, $CDCl_3$) of 2-fluoro-1-phenylethan-1-one (3p)

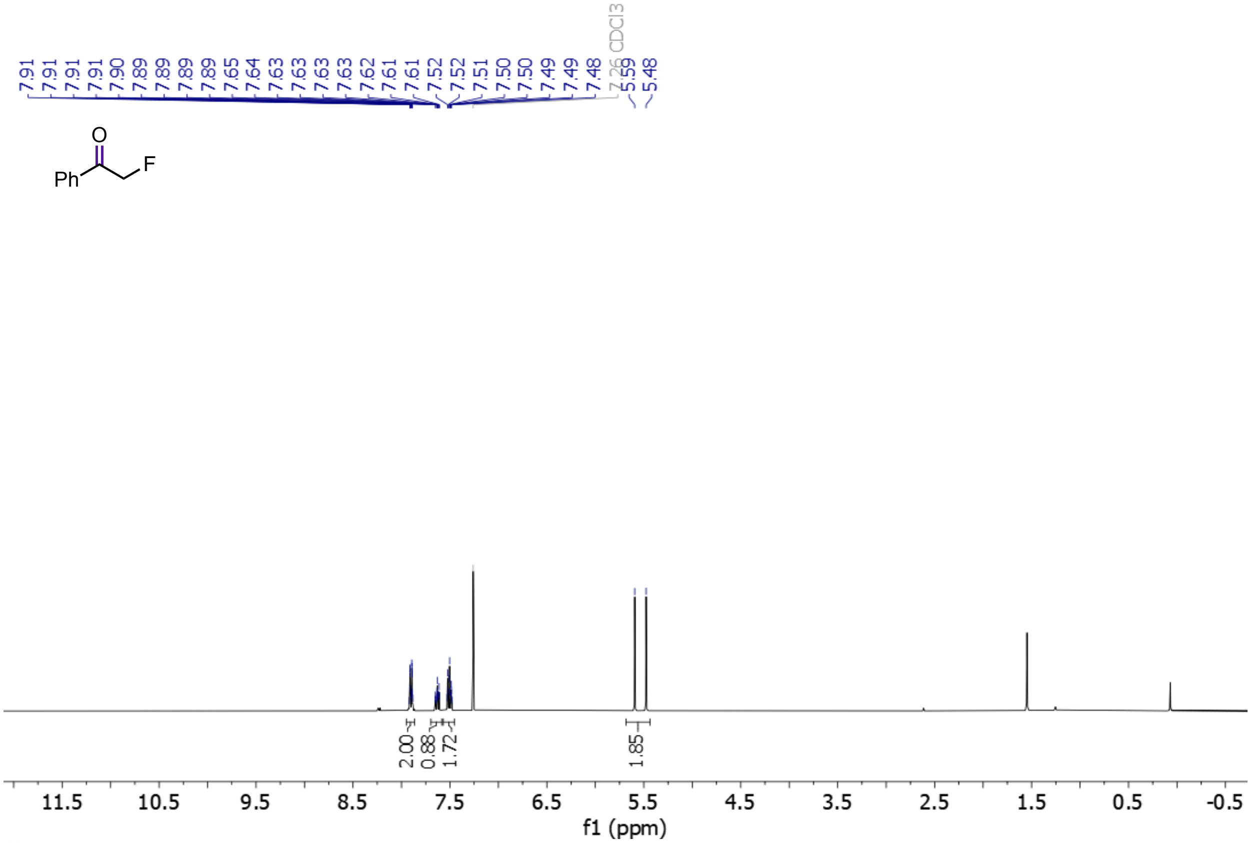

^{13}C NMR (101 MHz, $CDCl_3$) of 2-fluoro-1-phenylethan-1-one (3p)

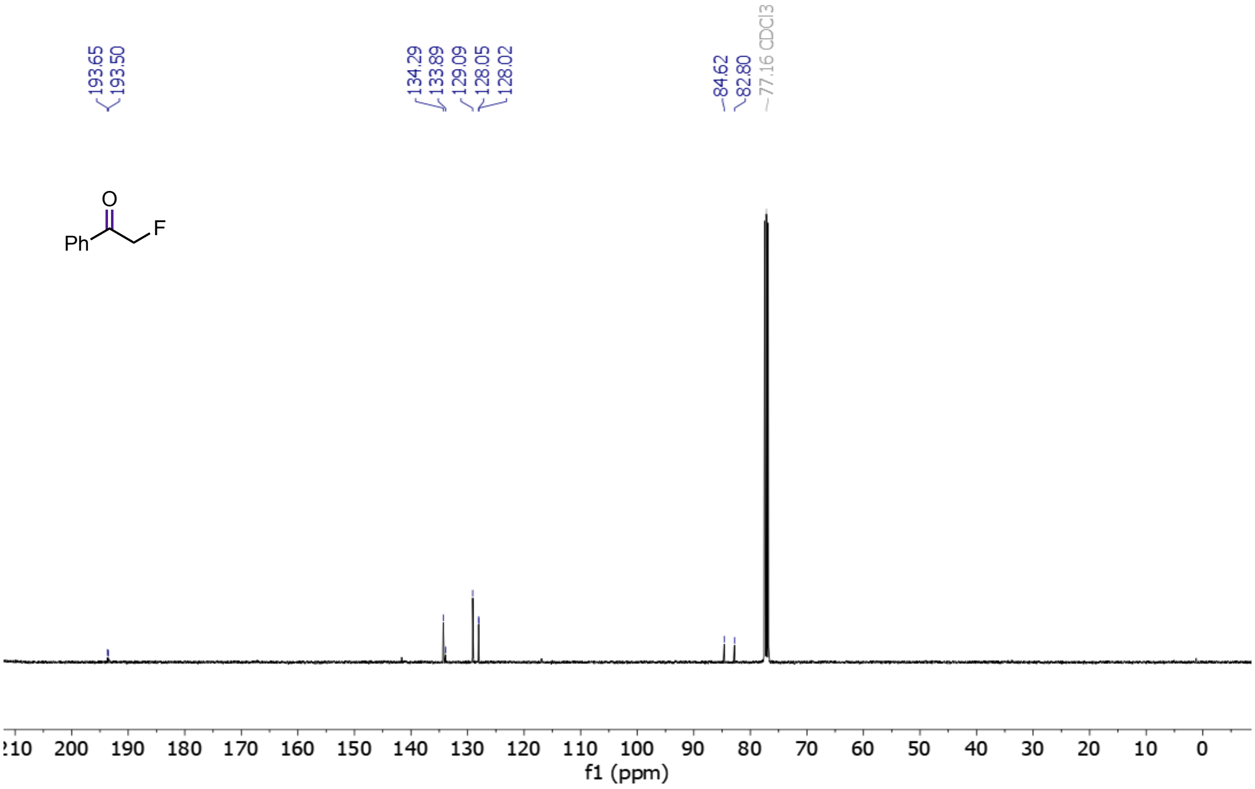

^{1}H NMR (400 MHz, $CDCl_3$) of 2-chloro-1-phenylethan-1-one (3q)

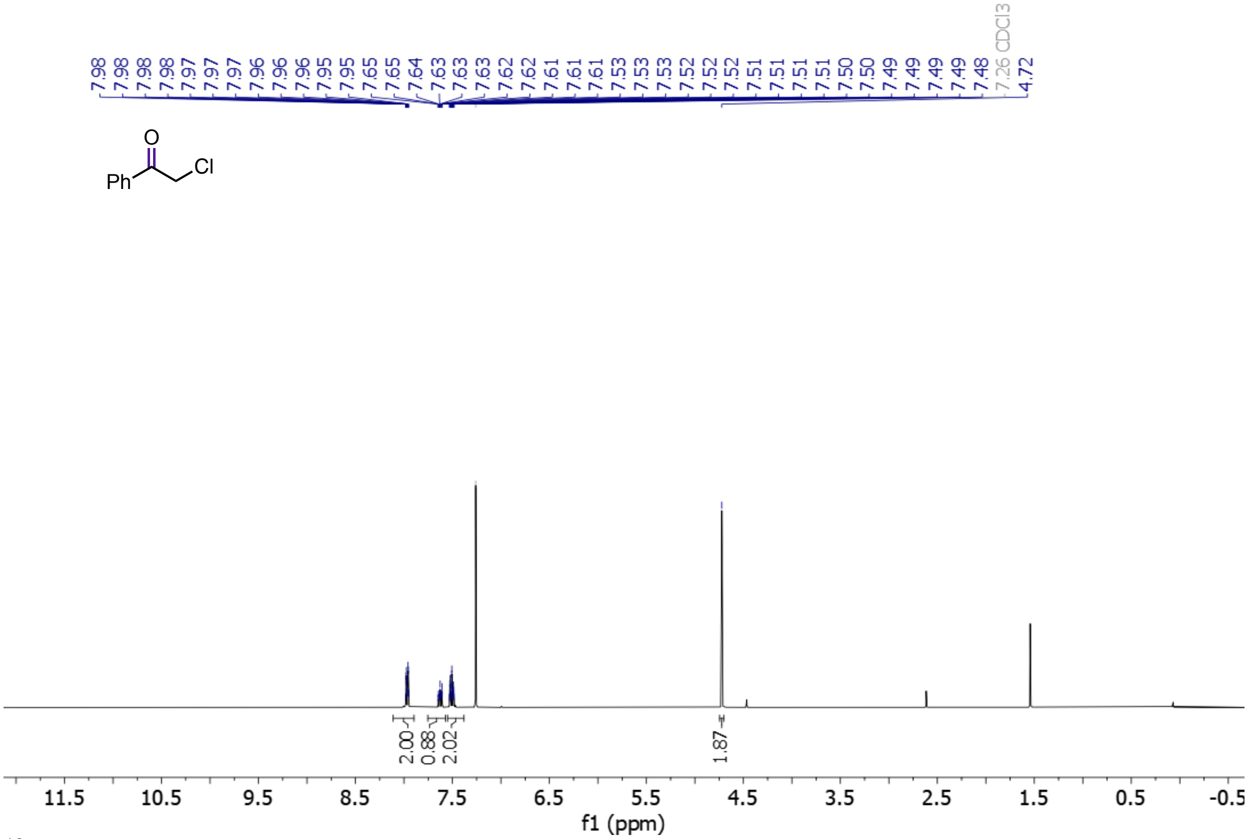

^{13}C NMR (101 MHz, $CDCl_3$) of 2-chloro-1-phenylethan-1-one (3q)

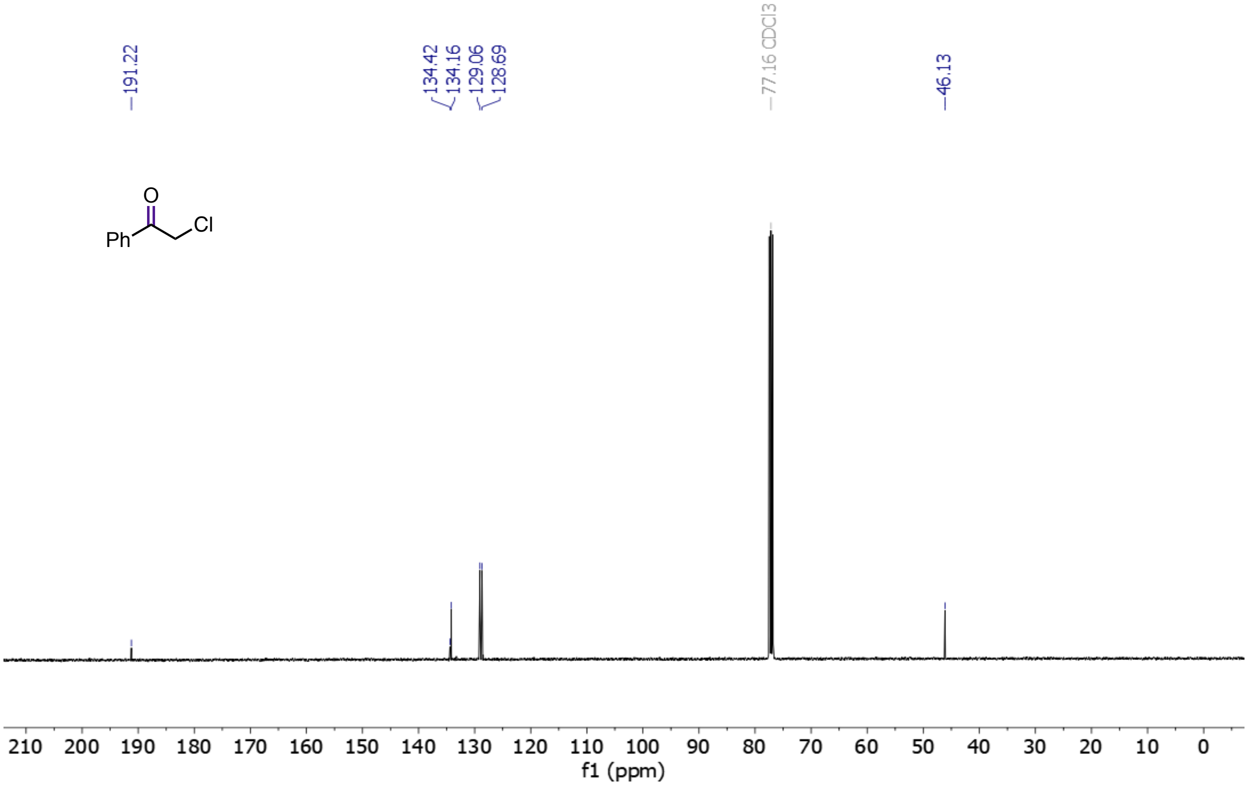

^{1}H NMR (400 MHz, $CDCl_3$) of 2-bromo-1-phenylethan-1-one (3r)

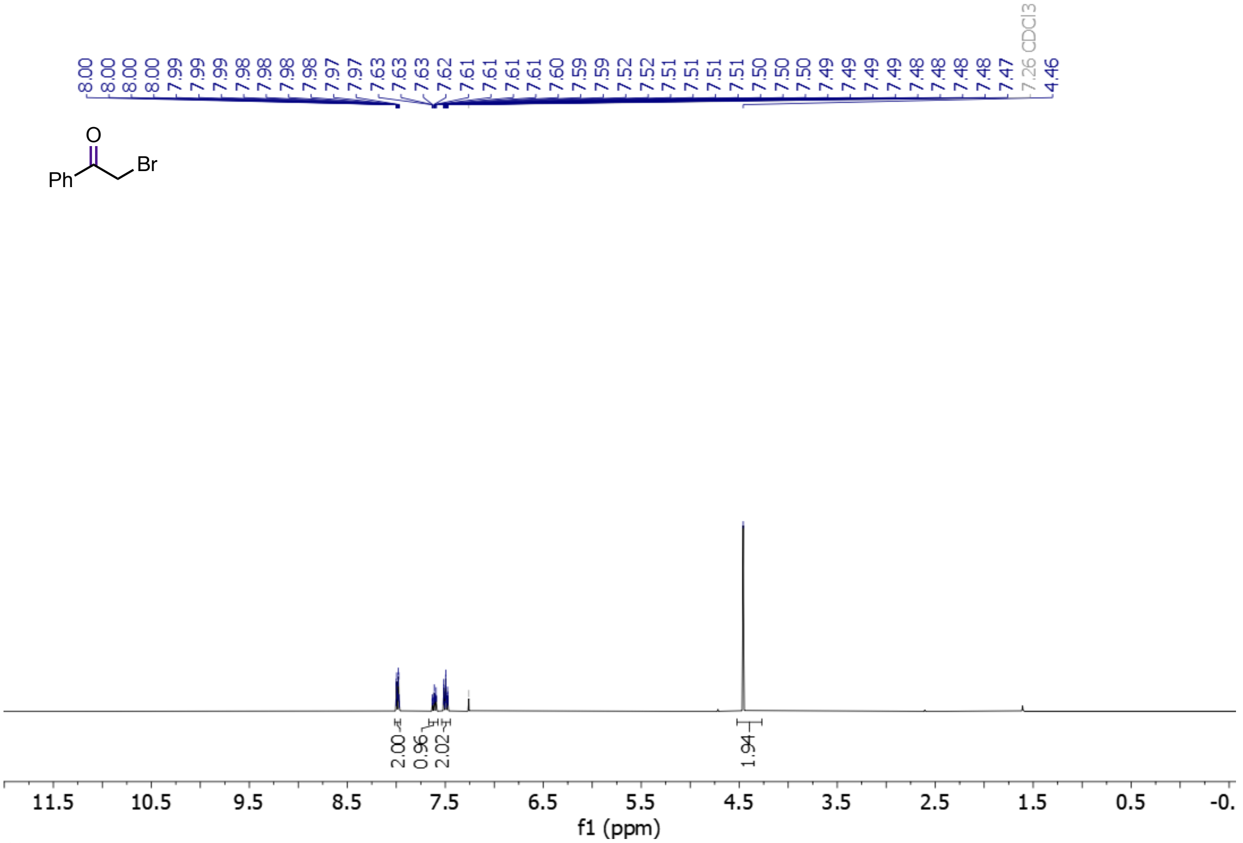

^{13}C NMR (101 MHz, $CDCl_3$) of 2-bromo-1-phenylethan-1-one (3r)

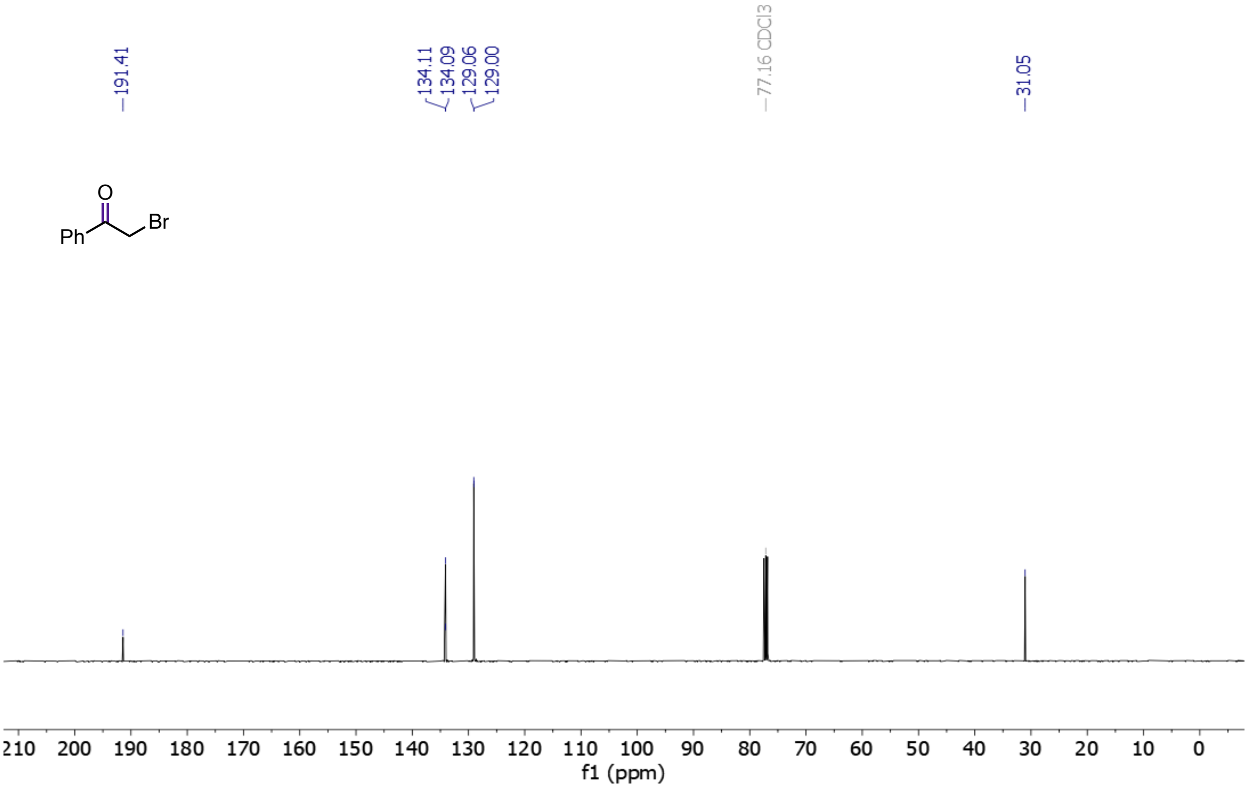

^{1}H NMR (400 MHz, CDCl$_3$) of 2-hydroxy-1-phenylethan-1-one (3s)

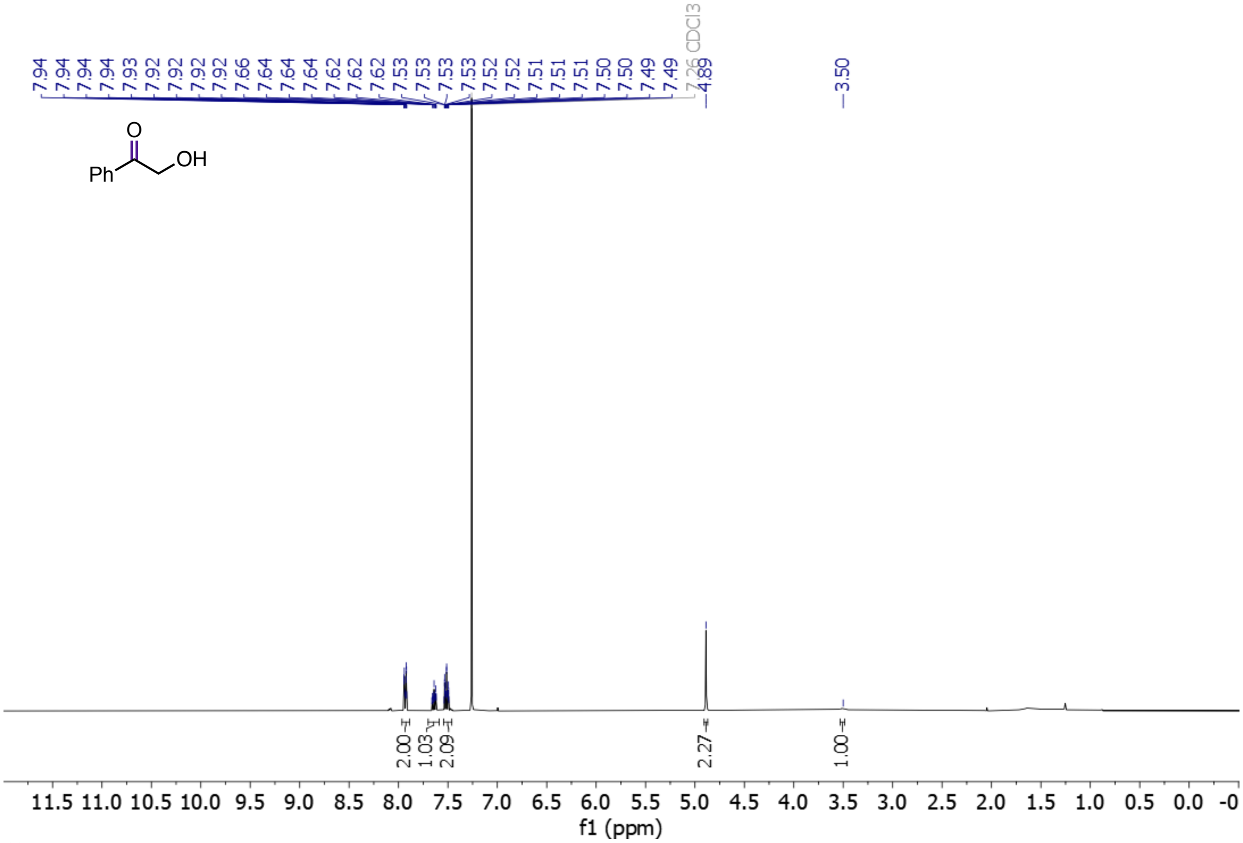

^{13}C NMR (101 MHz, CDCl$_3$) of 2-hydroxy-1-phenylethan-1-one (3s)

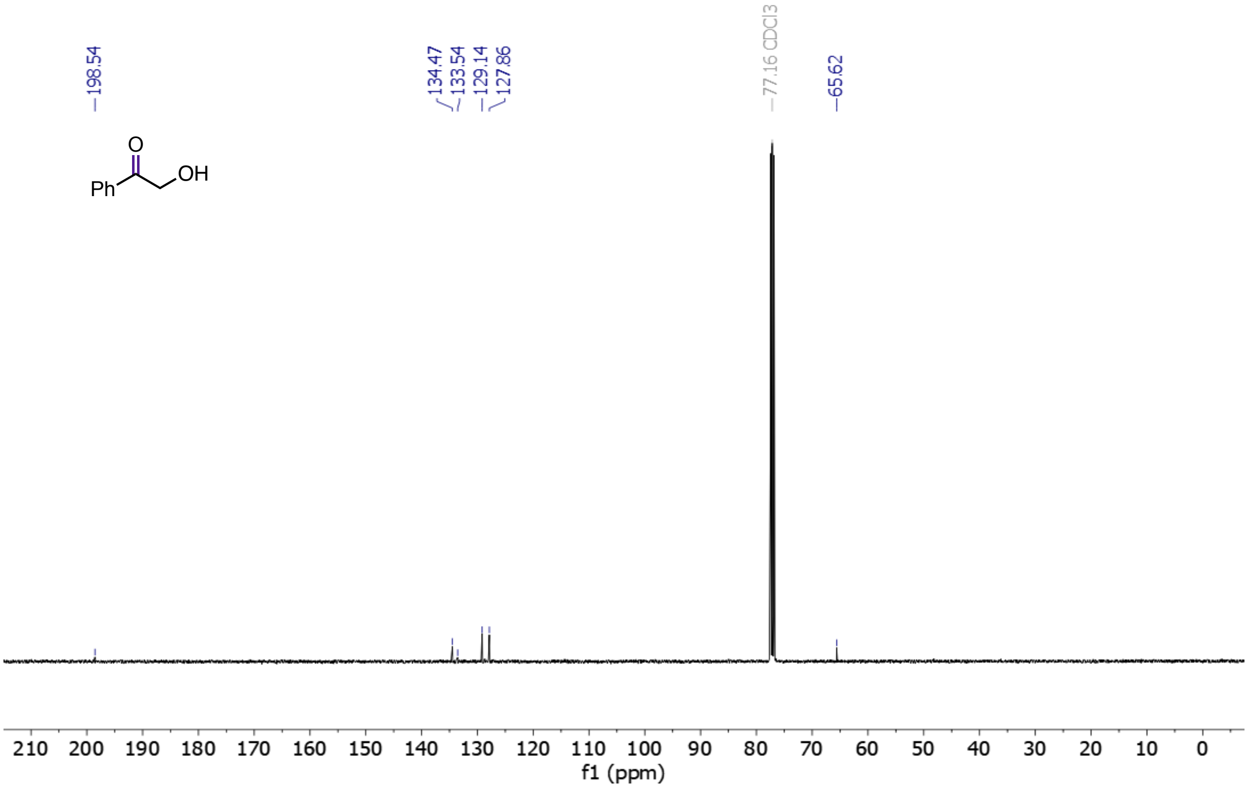

^{1}H NMR (400 MHz, CDCl$_3$) of Benzil (3t)

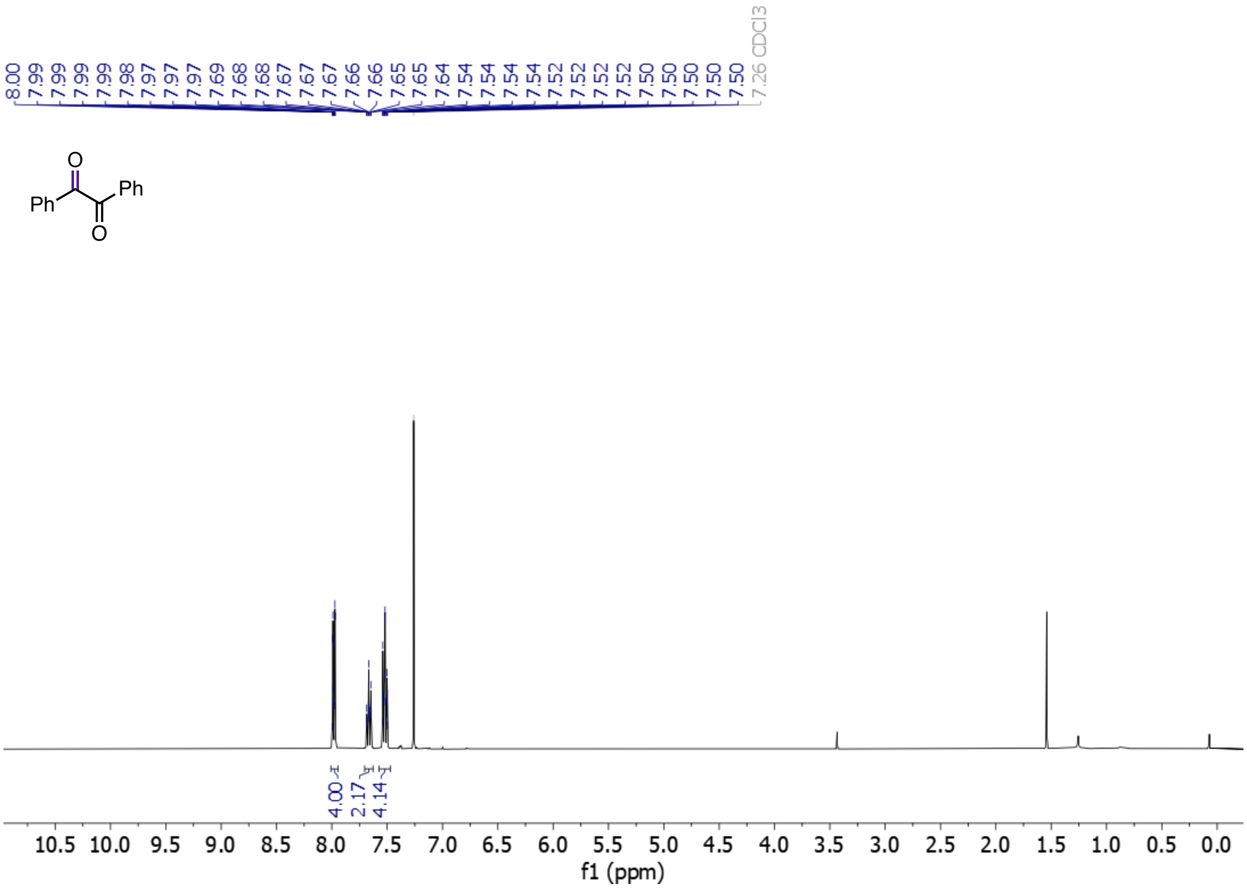

^{13}C NMR (101 MHz, CDCl$_3$) of Benzil (3t)

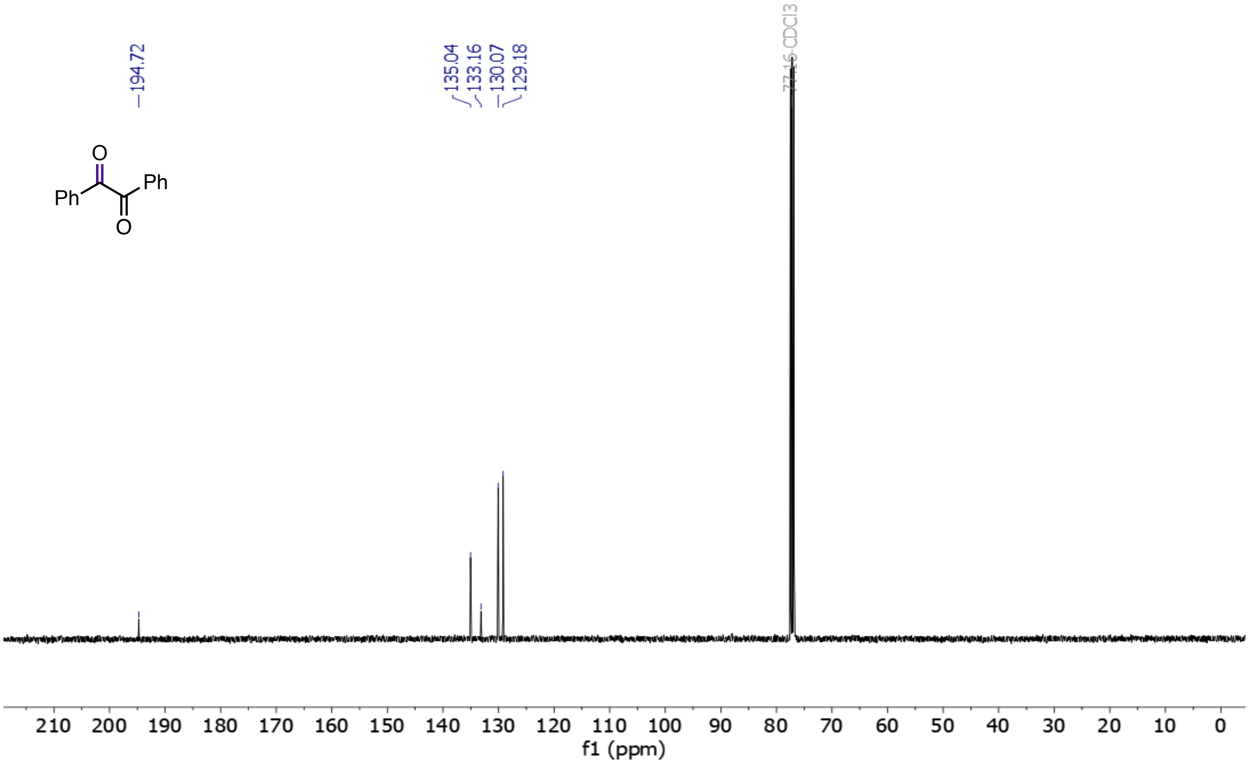

^{1}H NMR (400 MHz, $CDCl_3$) of 5-hydroxy-1-phenylpentan-1-one (3u)

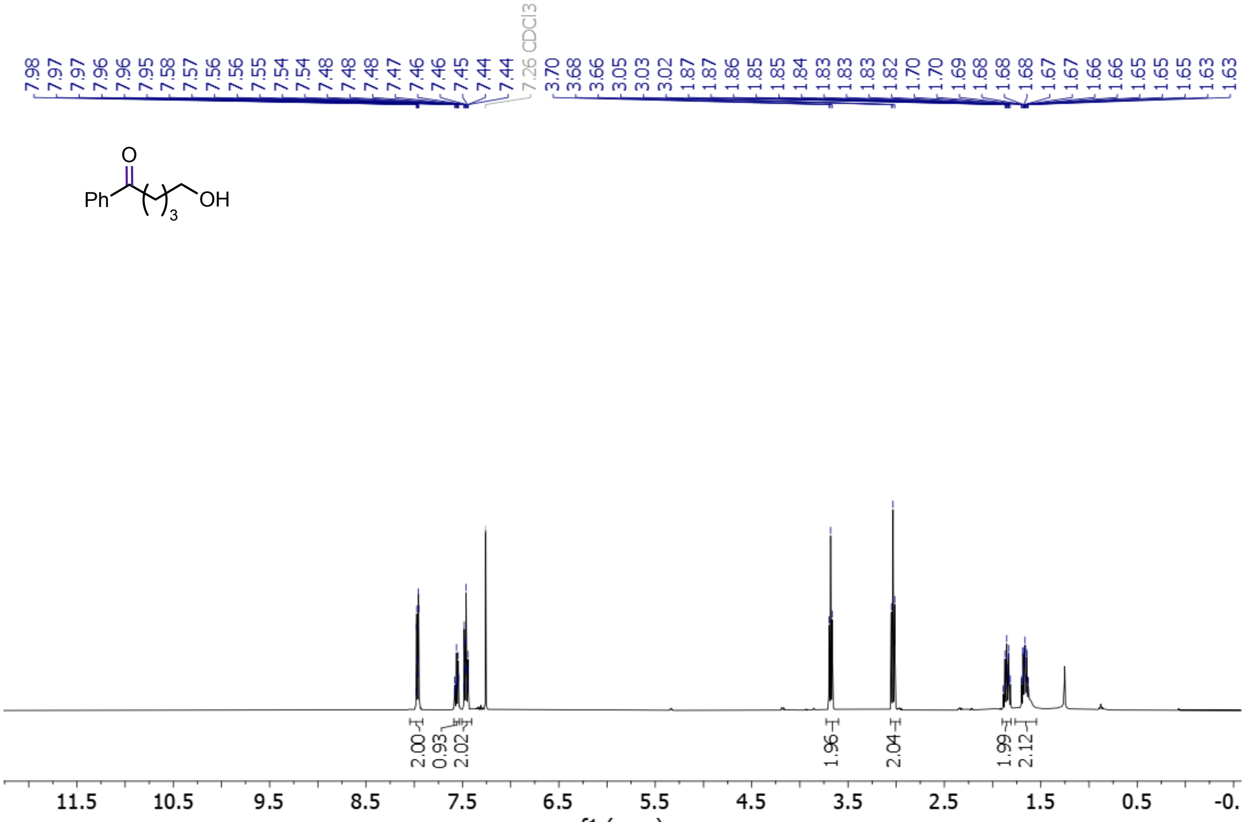

^{13}C NMR (101 MHz, $CDCl_3$) of 5-hydroxy-1-phenylpentan-1-one (3u)

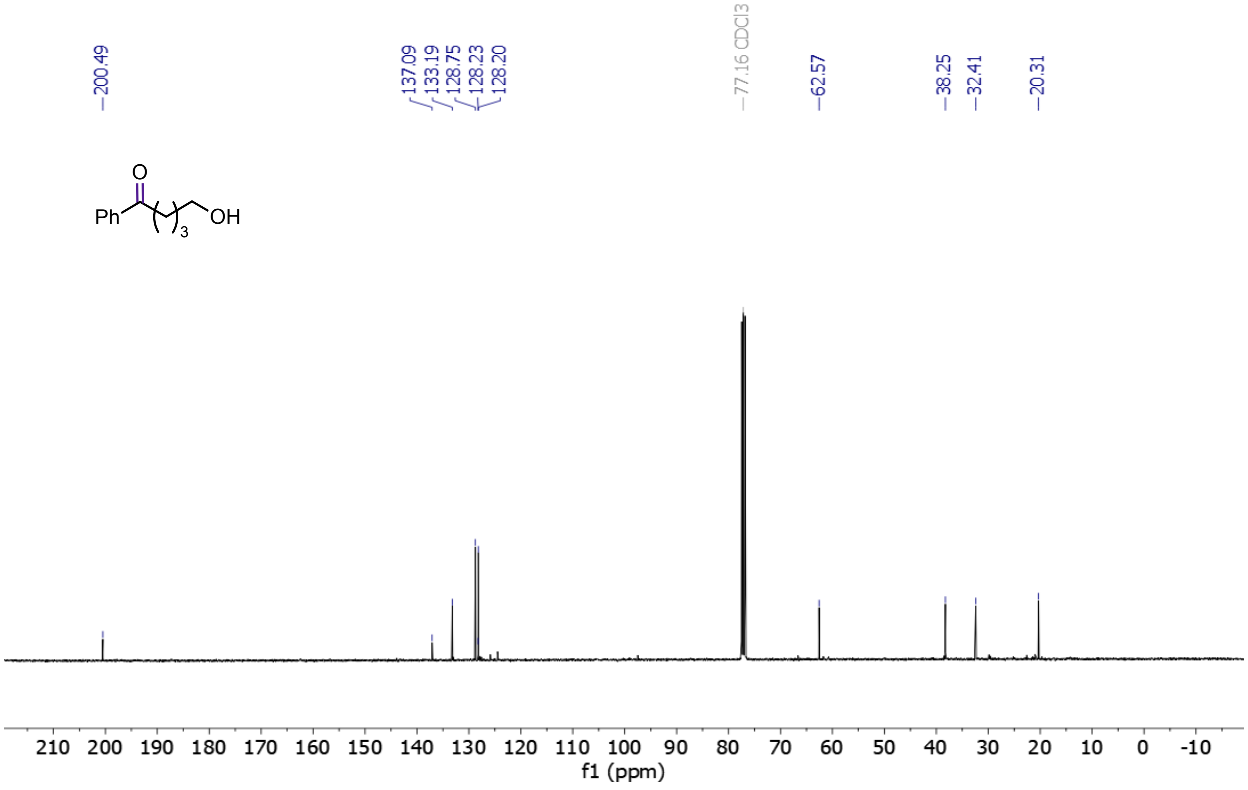

^{1}H NMR (400 MHz, CDCl₃) of 4-chloro-1-(4-fluorophenyl)butan-1-one (3v)

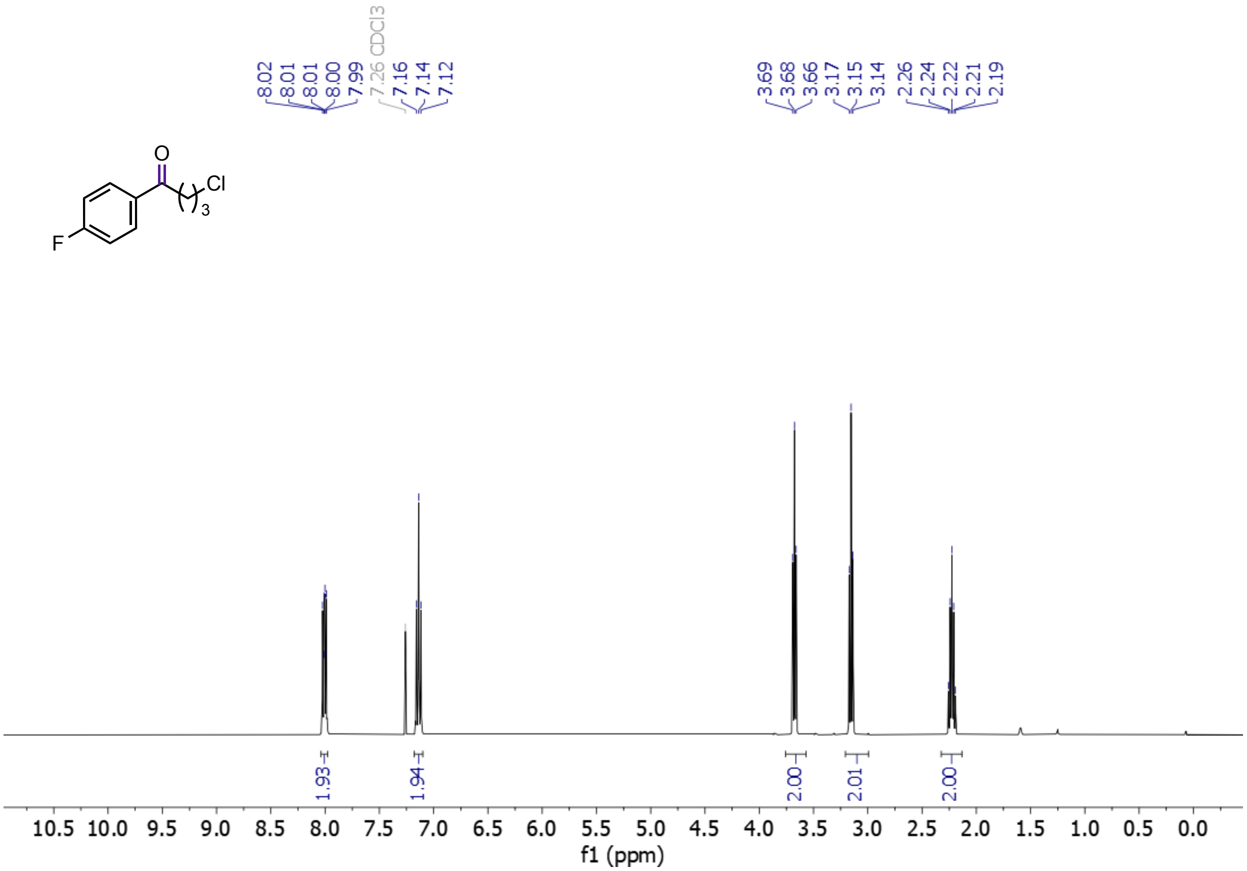

^{13}C NMR (101 MHz, CDCl₃) of 4-Chloro-1-(4-fluorophenyl)butan-1-one (3v)

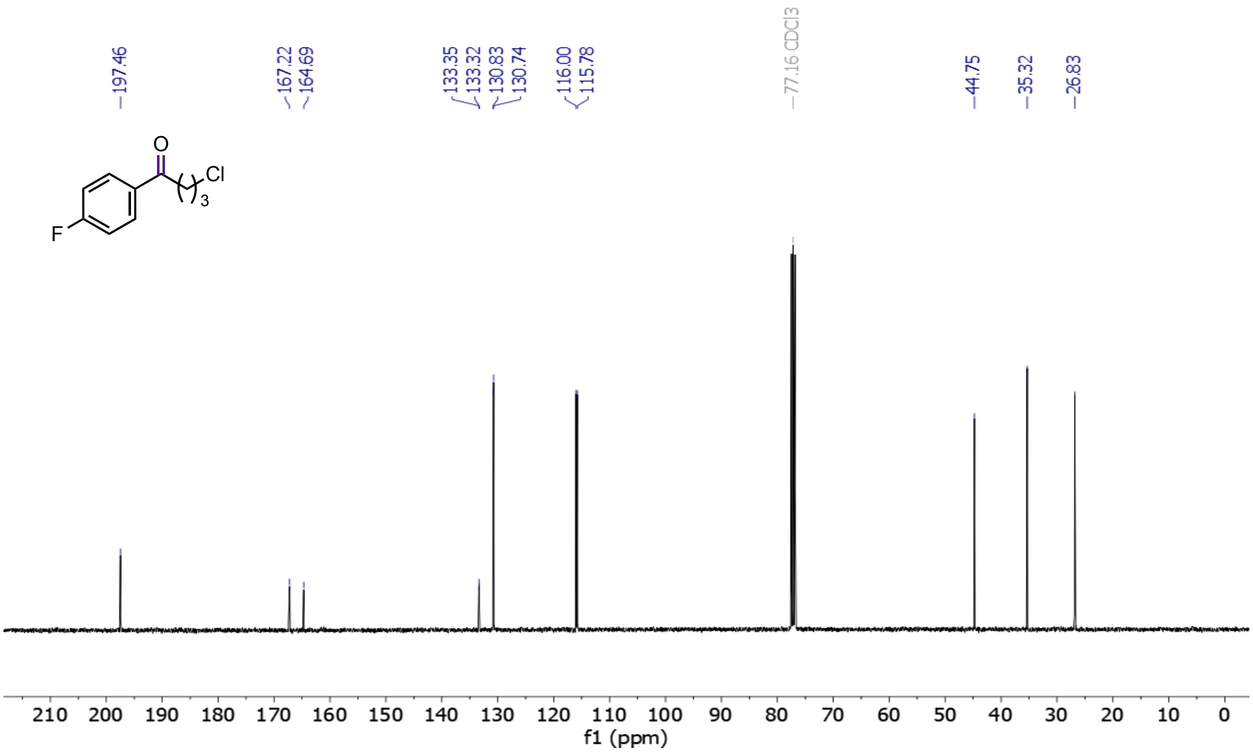

^{1}H NMR (500 MHz, $CDCl_3$) of 9*H*-fluoren-9-one (3w)

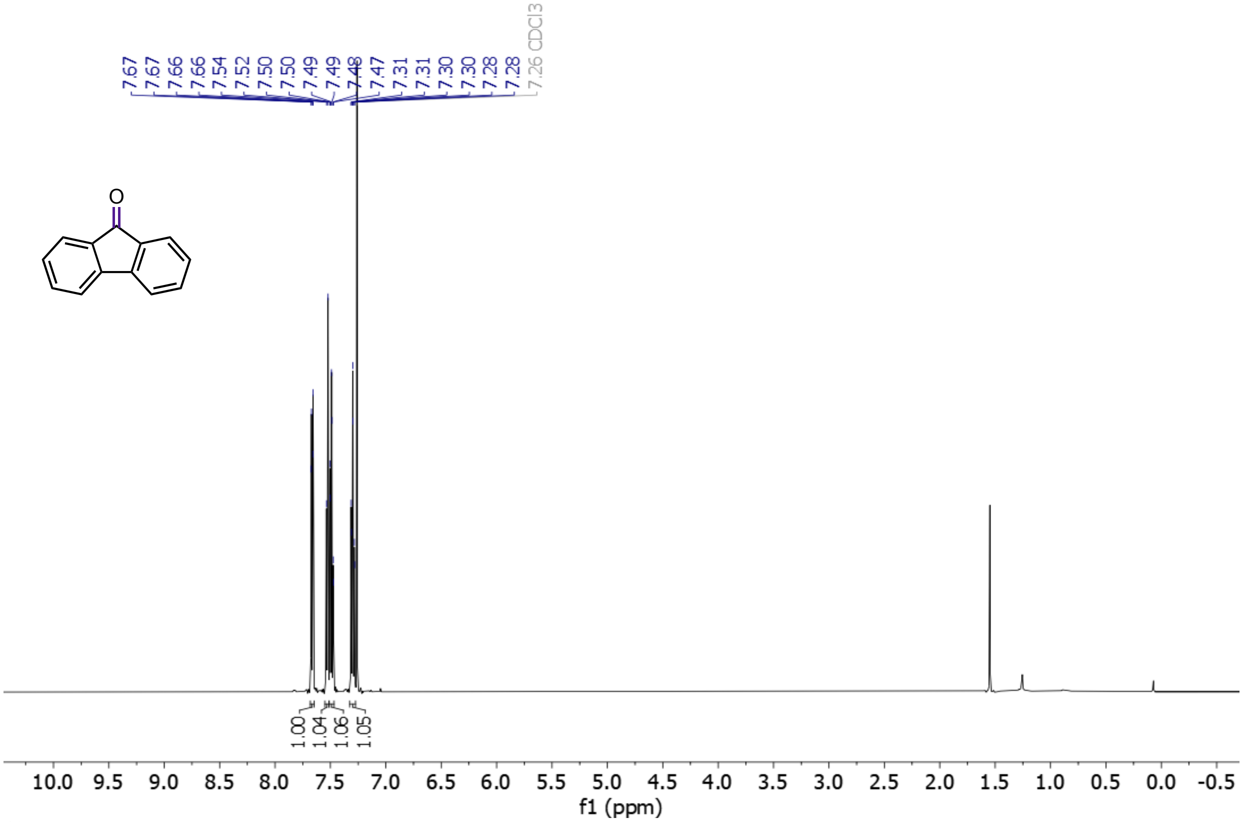

^{13}C NMR (126 MHz, $CDCl_3$) of 9*H*-fluoren-9-one (3w)

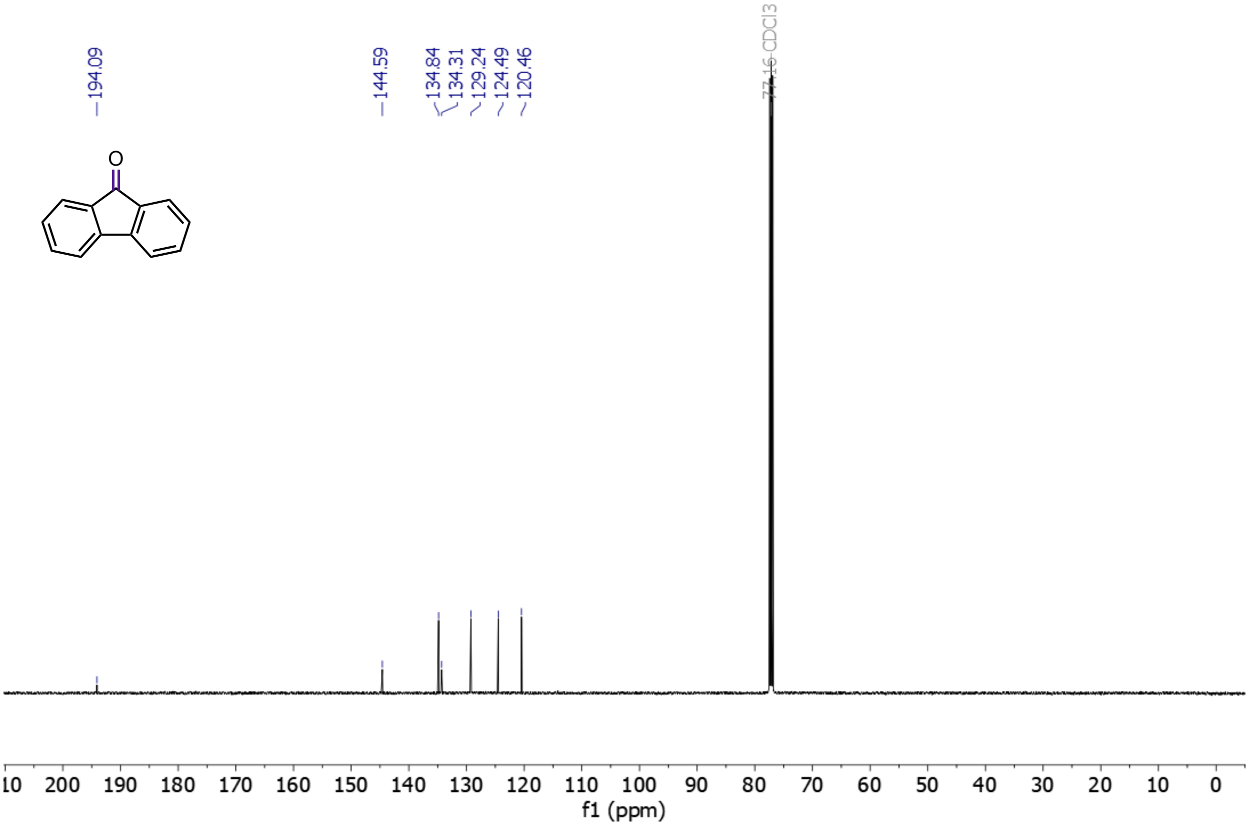

^{1}H NMR (400 MHz, $CDCl_3$) of (2,3-diphenylquinoxalin-6-yl)(phenyl)methanone (3x)

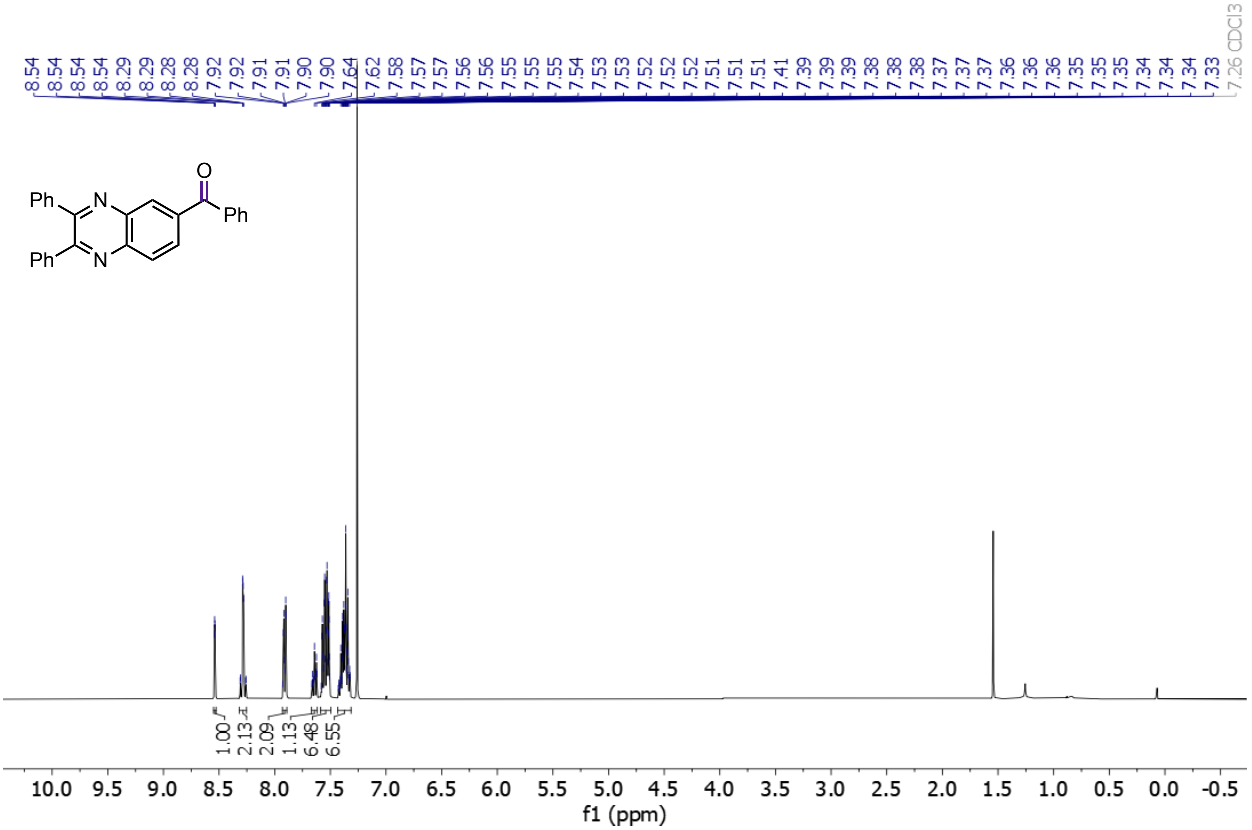

^{13}C NMR (126 MHz, $CDCl_3$) of (2,3-diphenylquinoxalin-6-yl)(phenyl)methanone (3x)

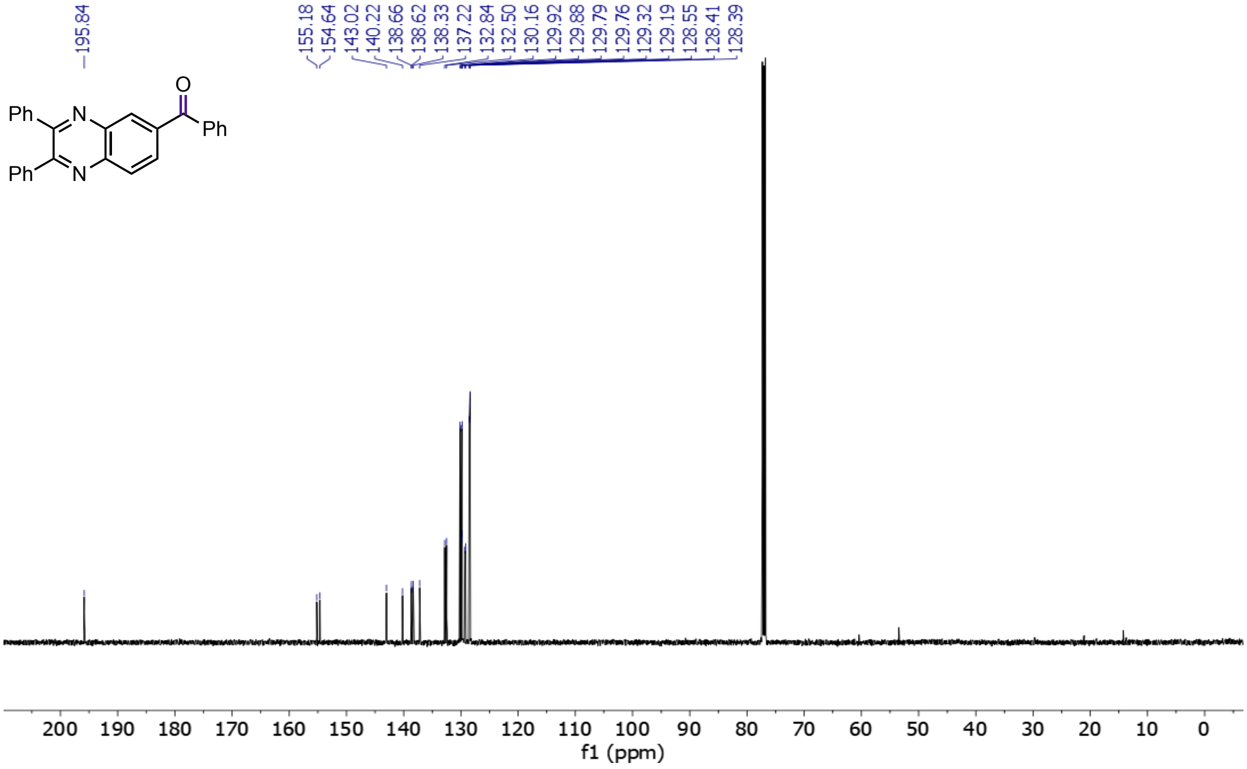

^{1}H NMR (600 MHz, $CDCl_3$) of (perchloropyridin-4-yl)(phenyl)methanone (3y)

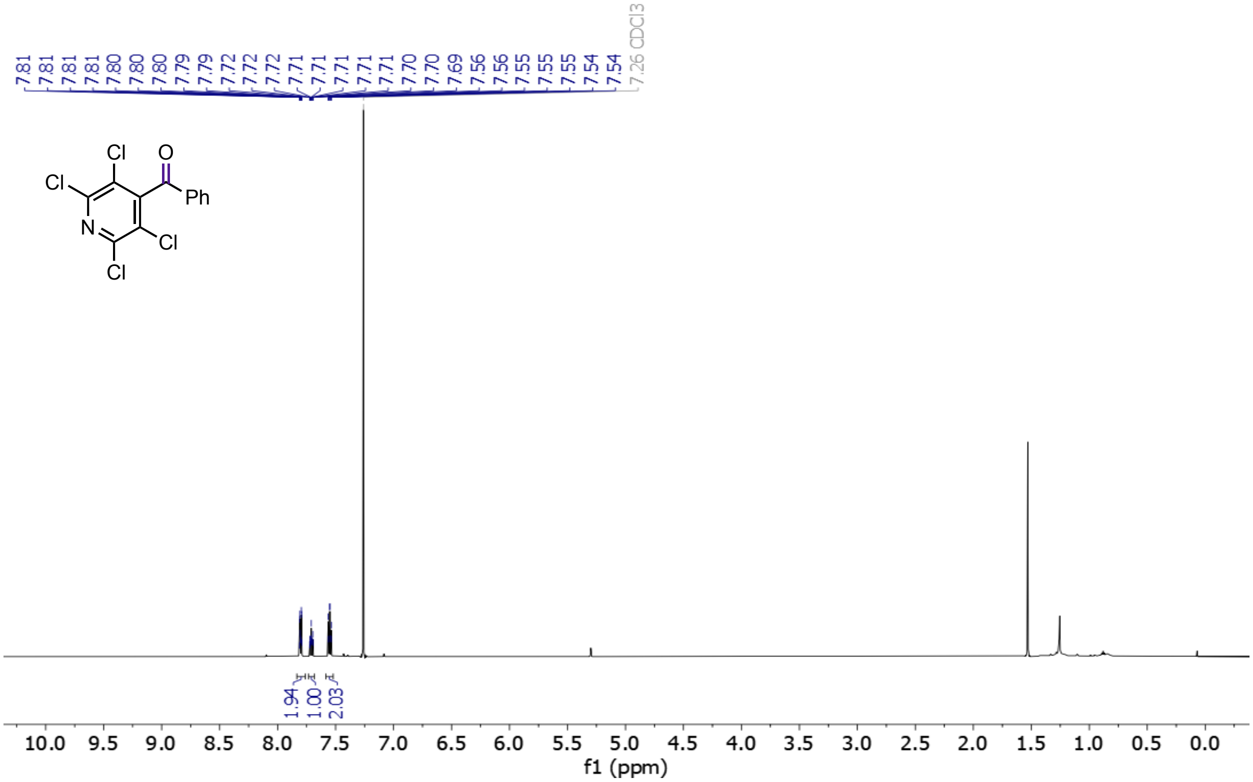

^{13}C NMR (126 MHz, $CDCl_3$) of (perchloropyridin-4-yl)(phenyl)methanone (3y)

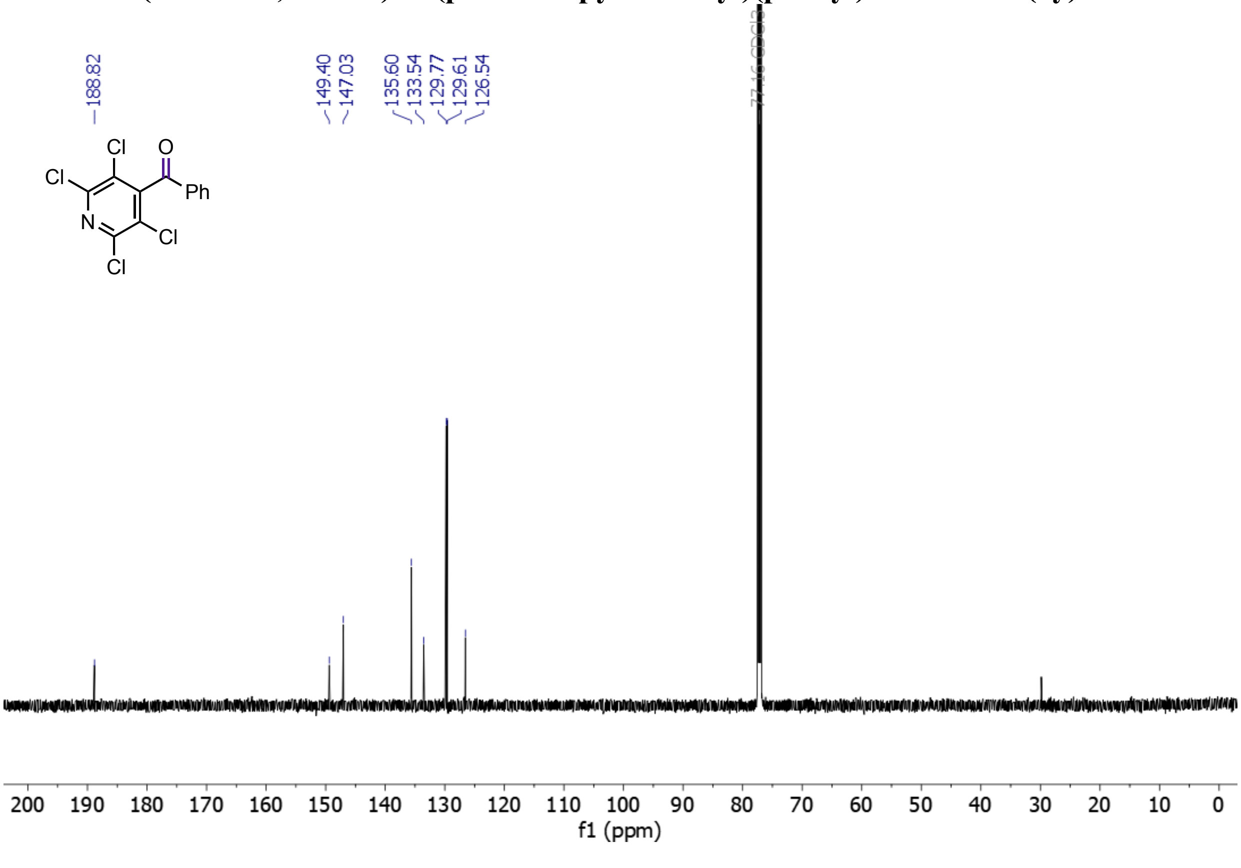

^{1}H NMR (500 MHz, $CDCl_3$) of nonan-5-one (3z)

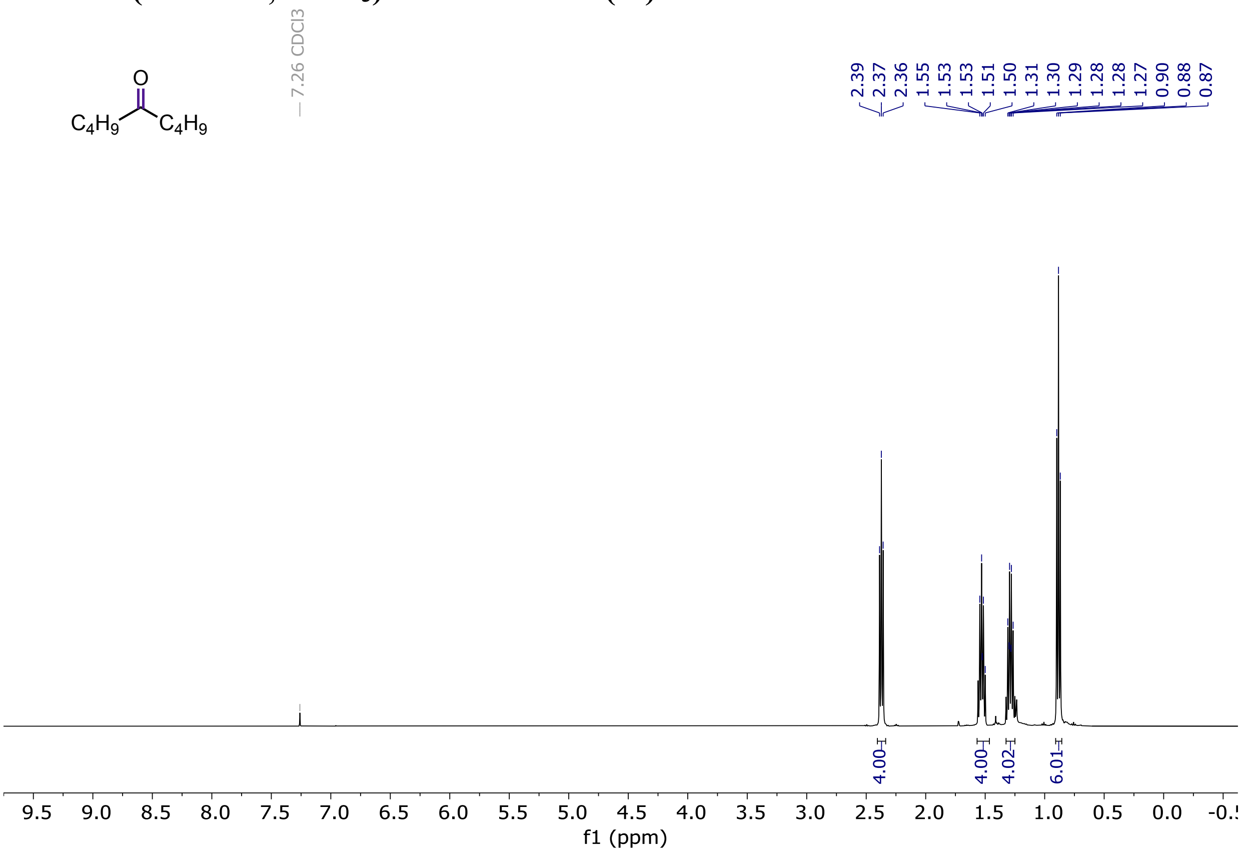

^{13}C NMR (126 MHz, $CDCl_3$) of nonan-5-one (3z)

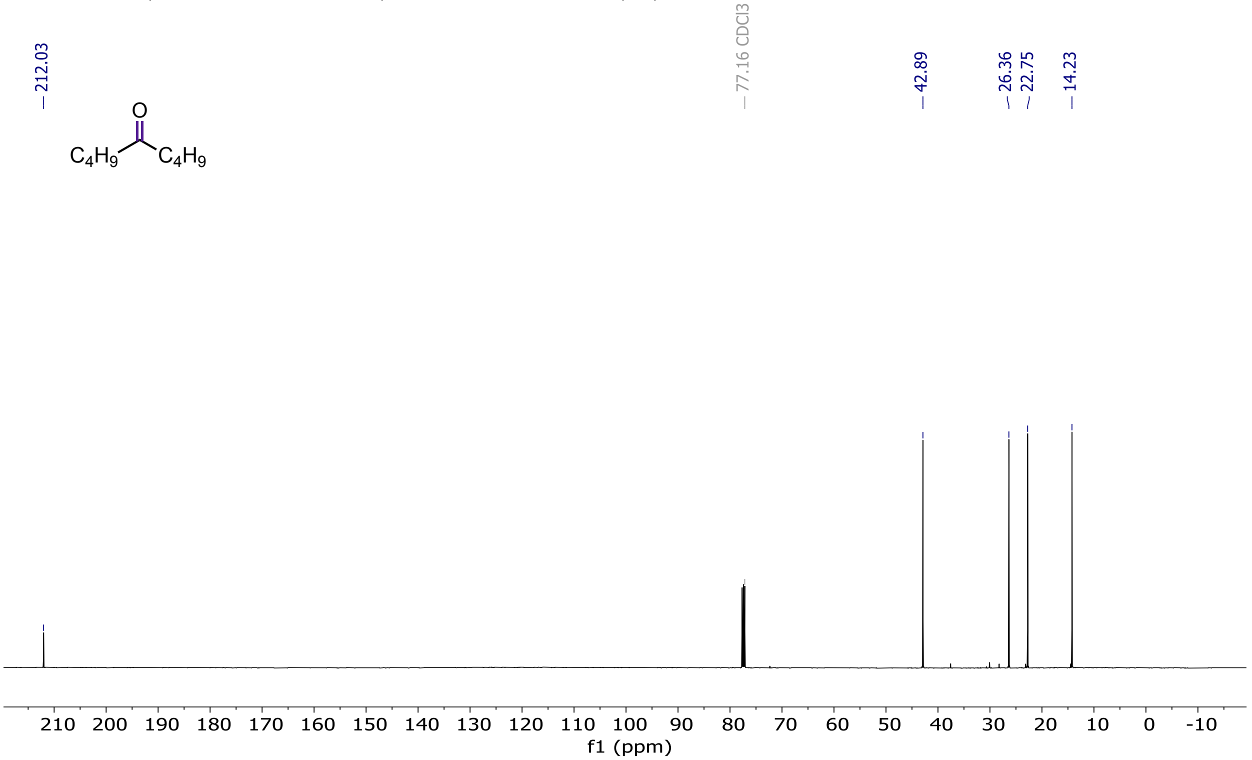

^{1}H NMR (500 MHz, $CDCl_3$) of pentadecan-8-one (3aa)

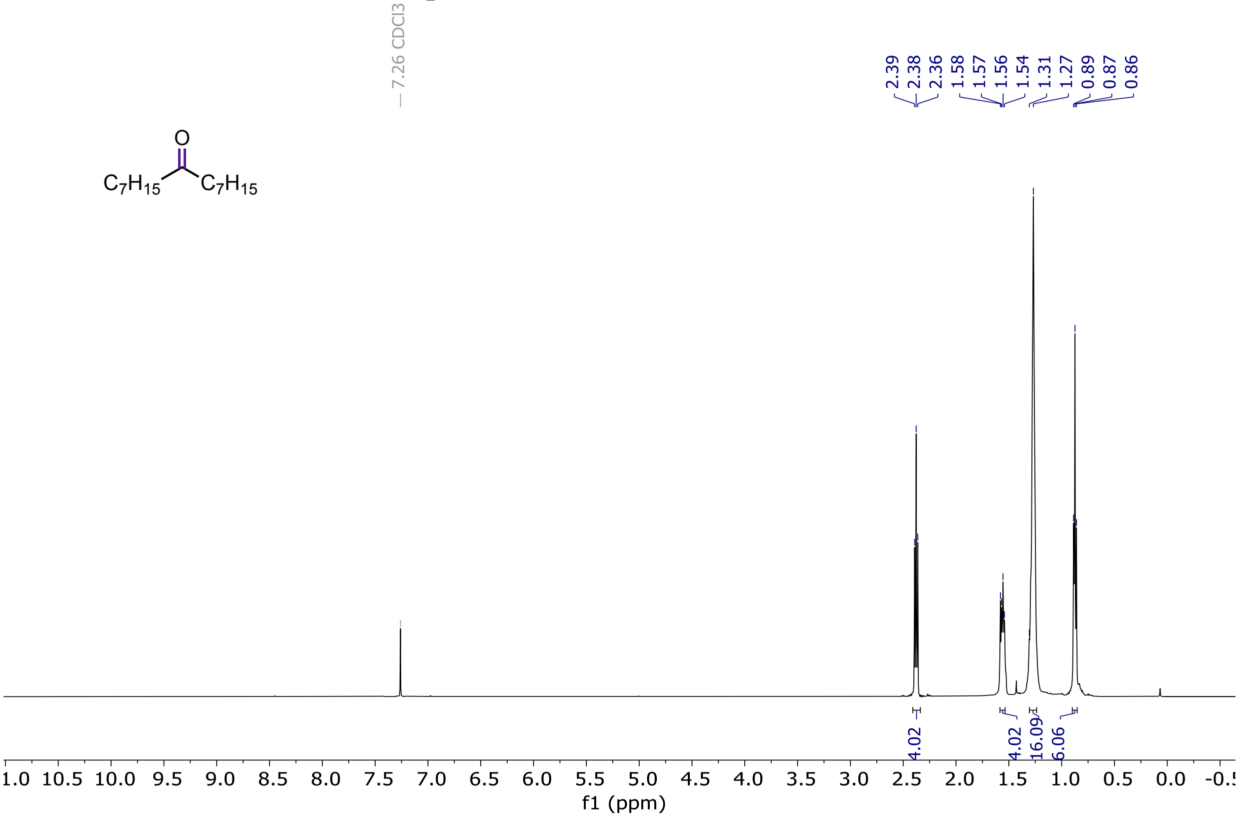

^{13}C NMR (126 MHz, $CDCl_3$) of pentadecan-8-one (3aa)

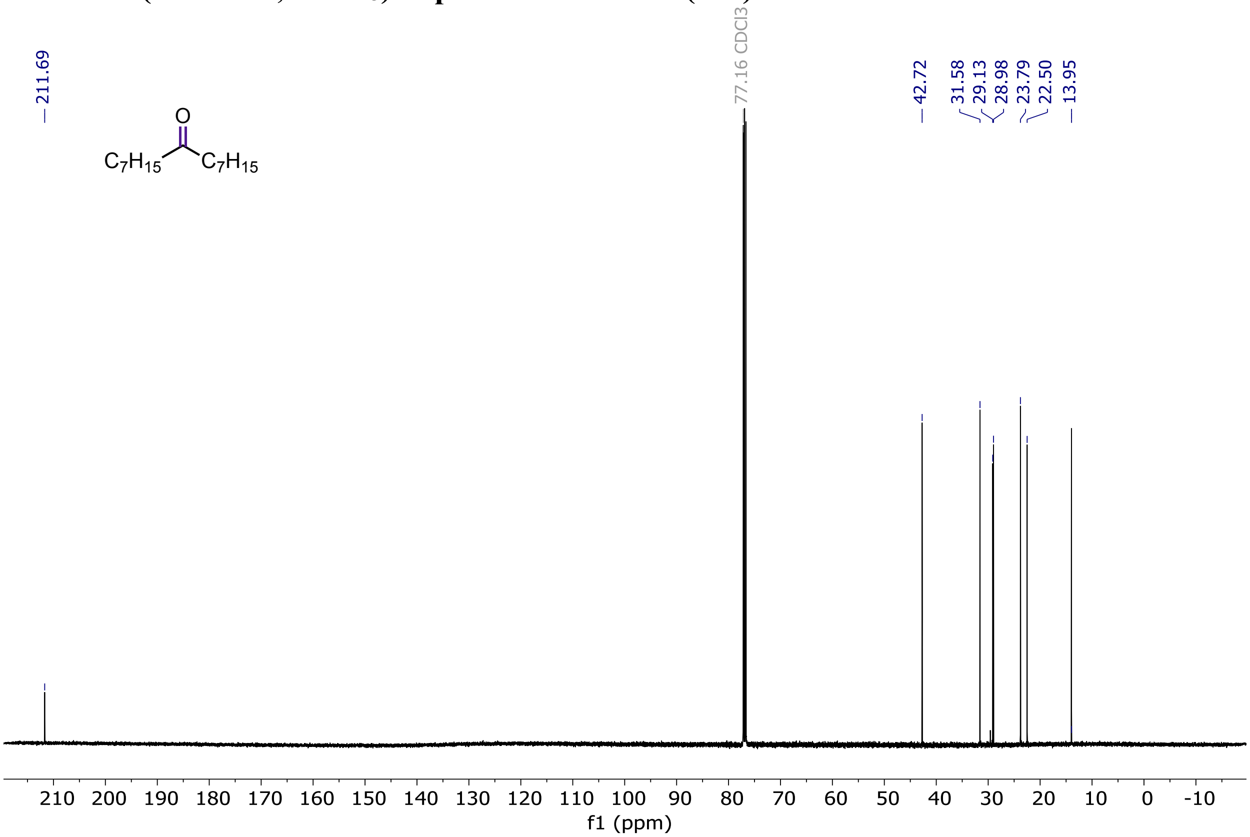

^{1}H NMR (500 MHz, $CDCl_3$) of Dicyclohexylmethanone (3ab)

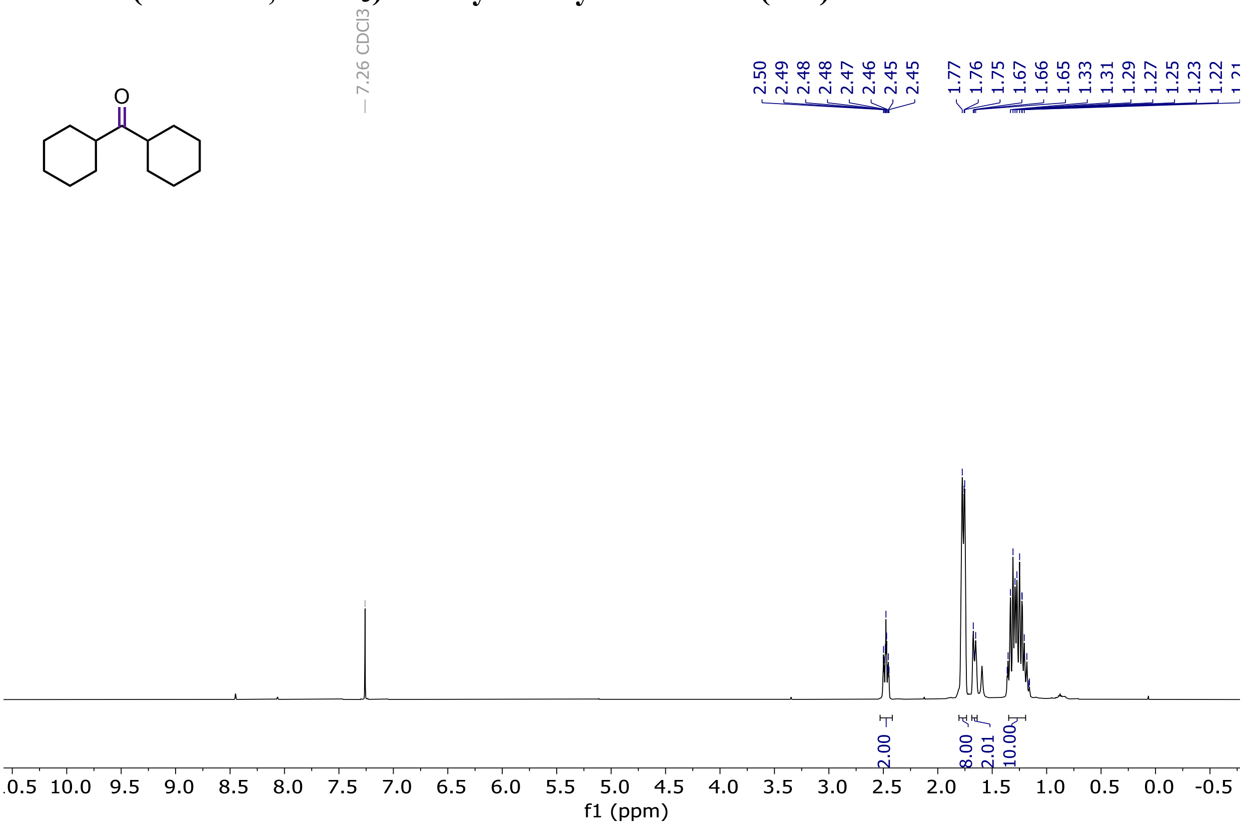

^{13}C NMR (126 MHz, $CDCl_3$) of Dicyclohexylmethanone (3ab)

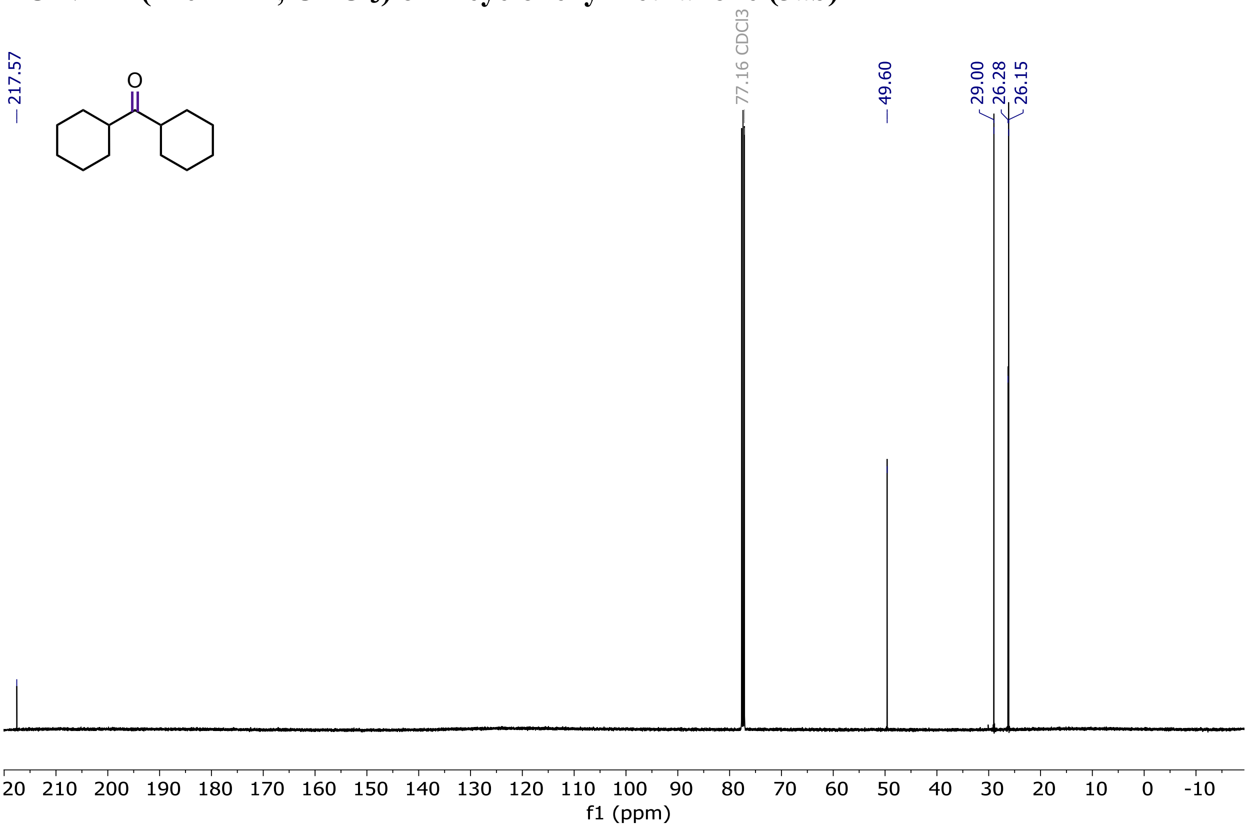

¹H NMR (400 MHz, CDCl₃) of 1-cyclobutylidene-2-(2,4-dinitrophenyl)hydrazine (3ac’)

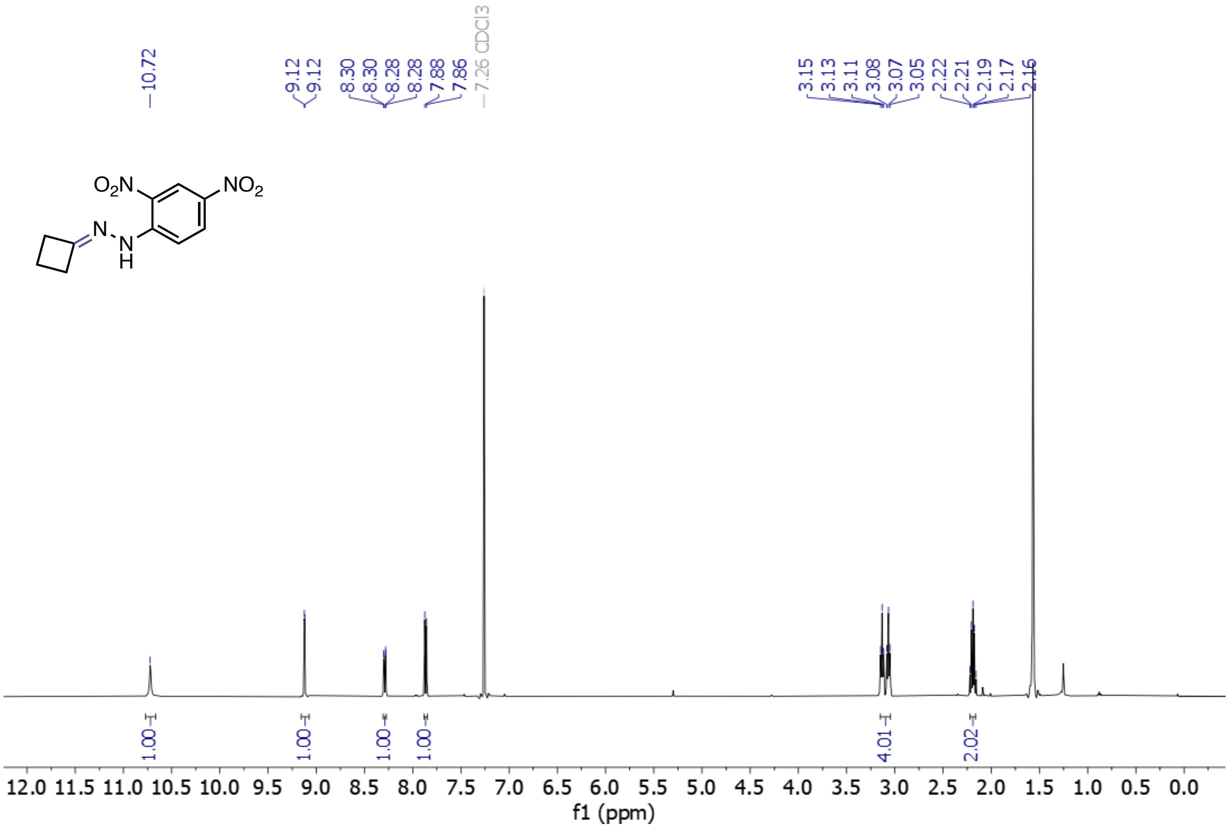

¹³C NMR (101 MHz, CDCl₃) of 1-cyclobutylidene-2-(2,4-dinitrophenyl)hydrazine (3ac’)

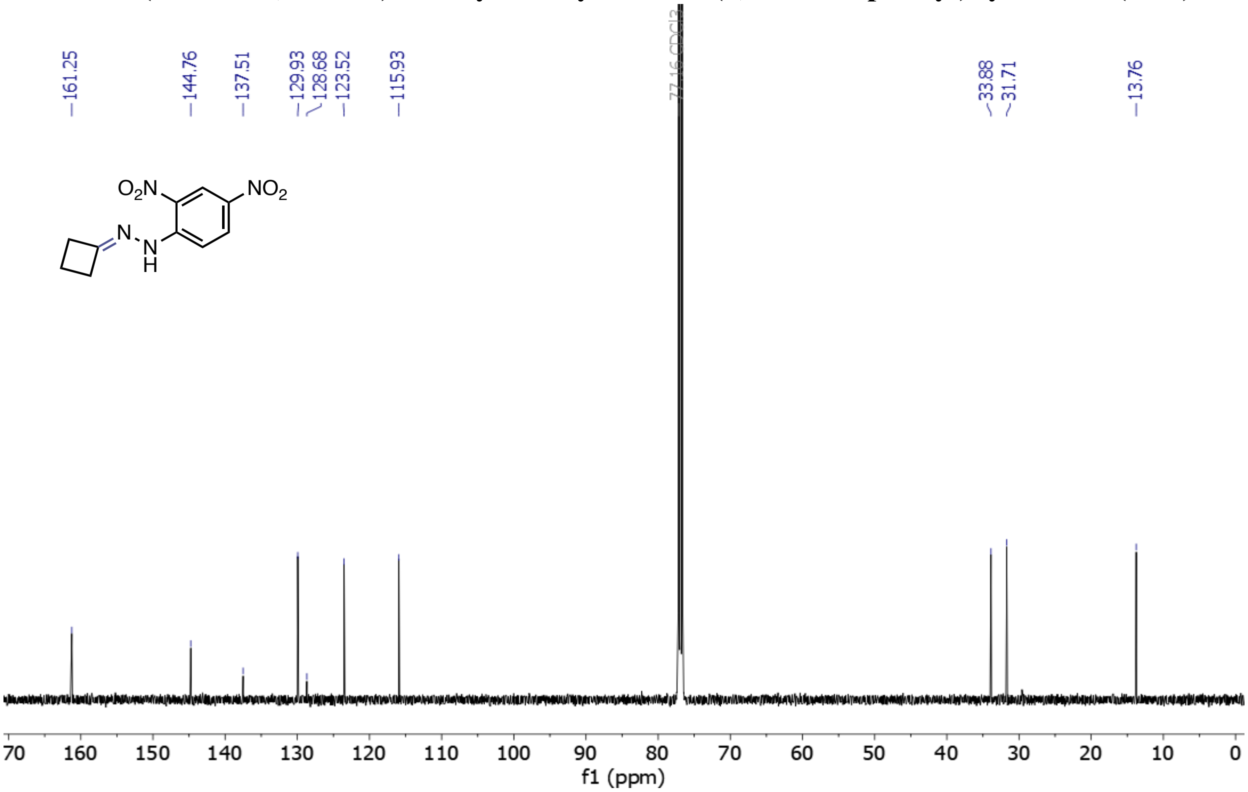

^{1}H NMR (400 MHz, $CDCl_3$) of 1-cyclopentylidene-2-(2,4-dinitrophenyl)hydrazine (3ad')

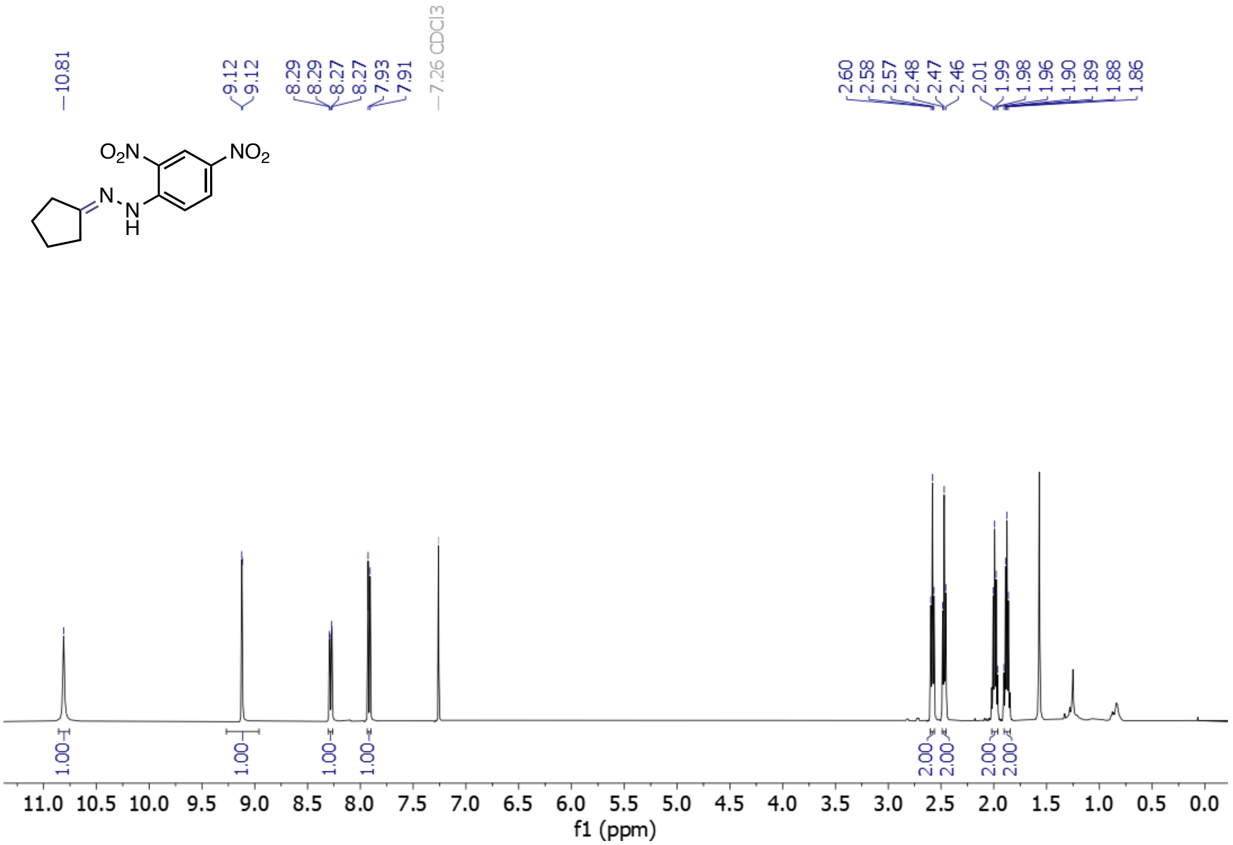

^{13}C NMR (101 MHz, $CDCl_3$) of 1-cyclopentylidene-2-(2,4-dinitrophenyl)hydrazine (3ad')

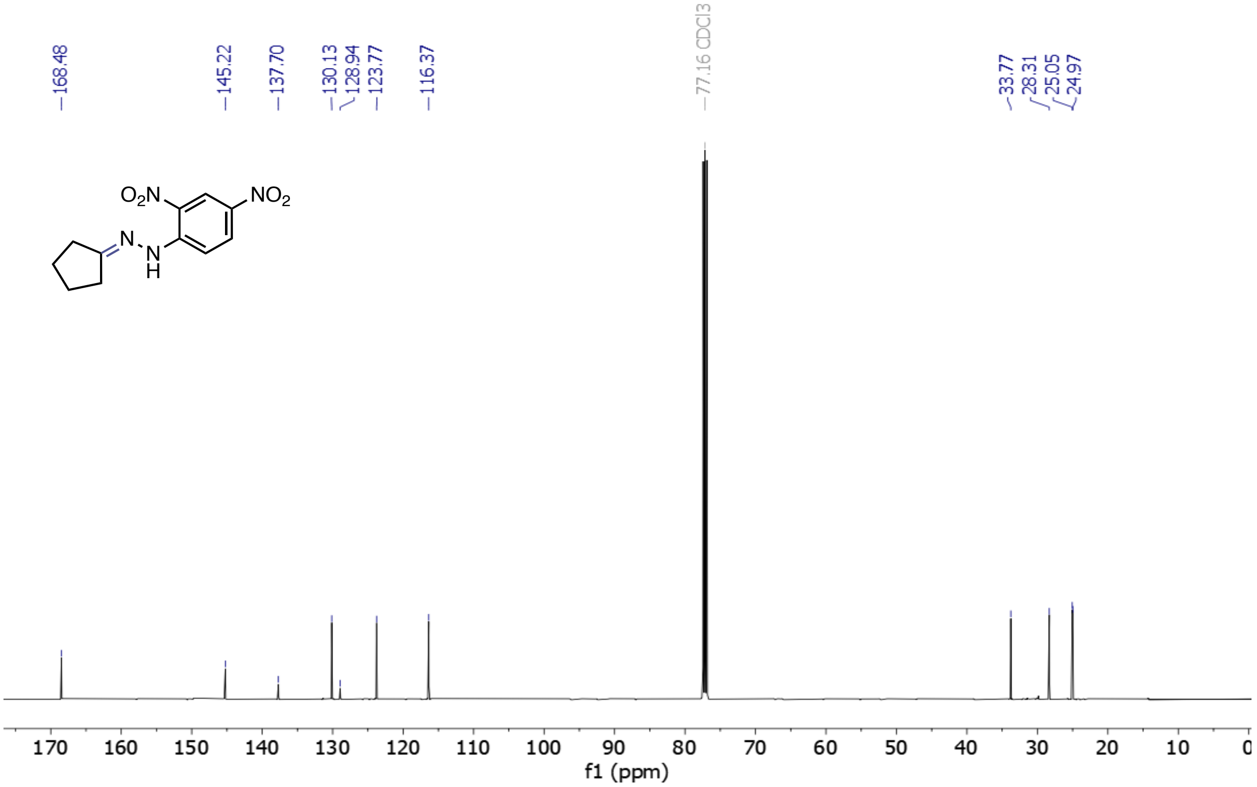

^{1}H NMR (400 MHz, CDCl₃) of 1-cyclohexylidene-2-(2,4-dinitrophenyl)hydrazine (3ae’)

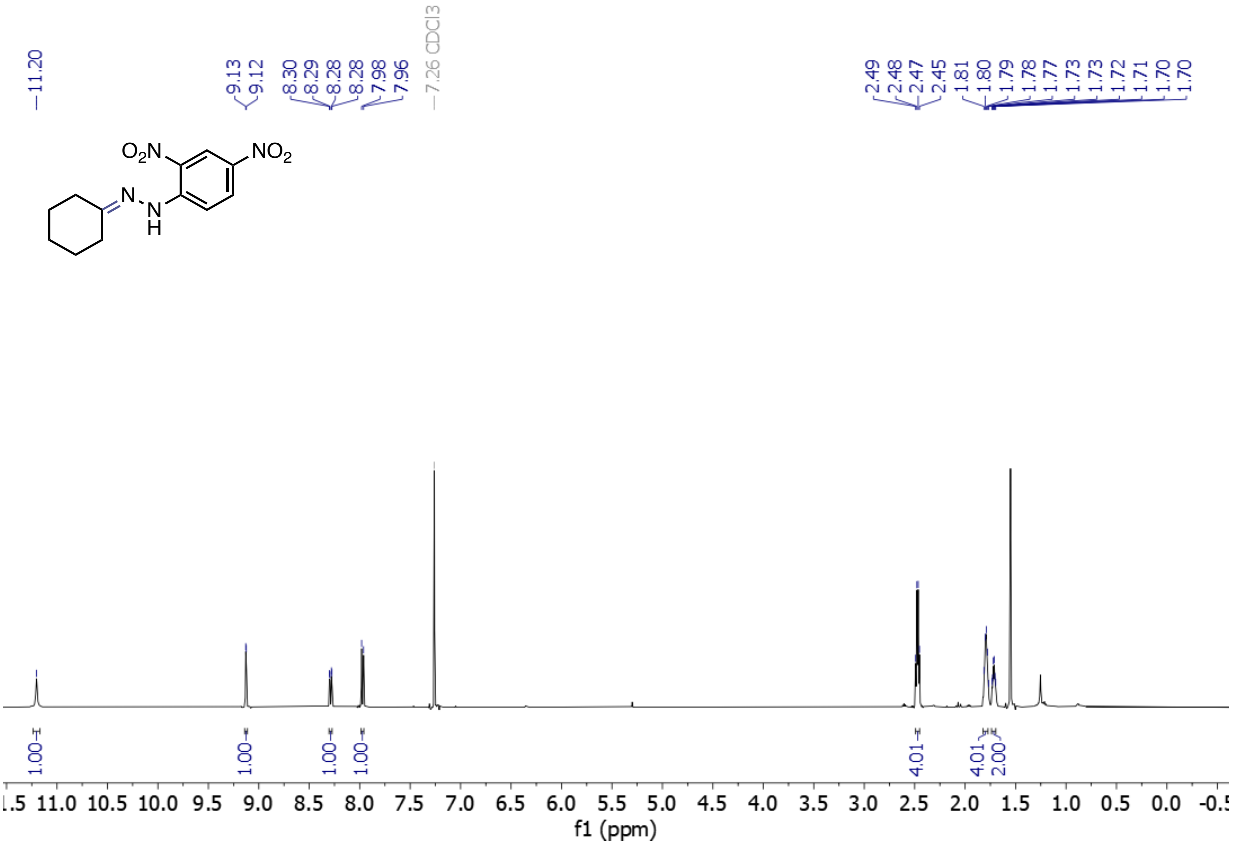

^{13}C NMR (101 MHz, CDCl₃) of 1-cyclohexylidene-2-(2,4-dinitrophenyl)hydrazine (3ae’)

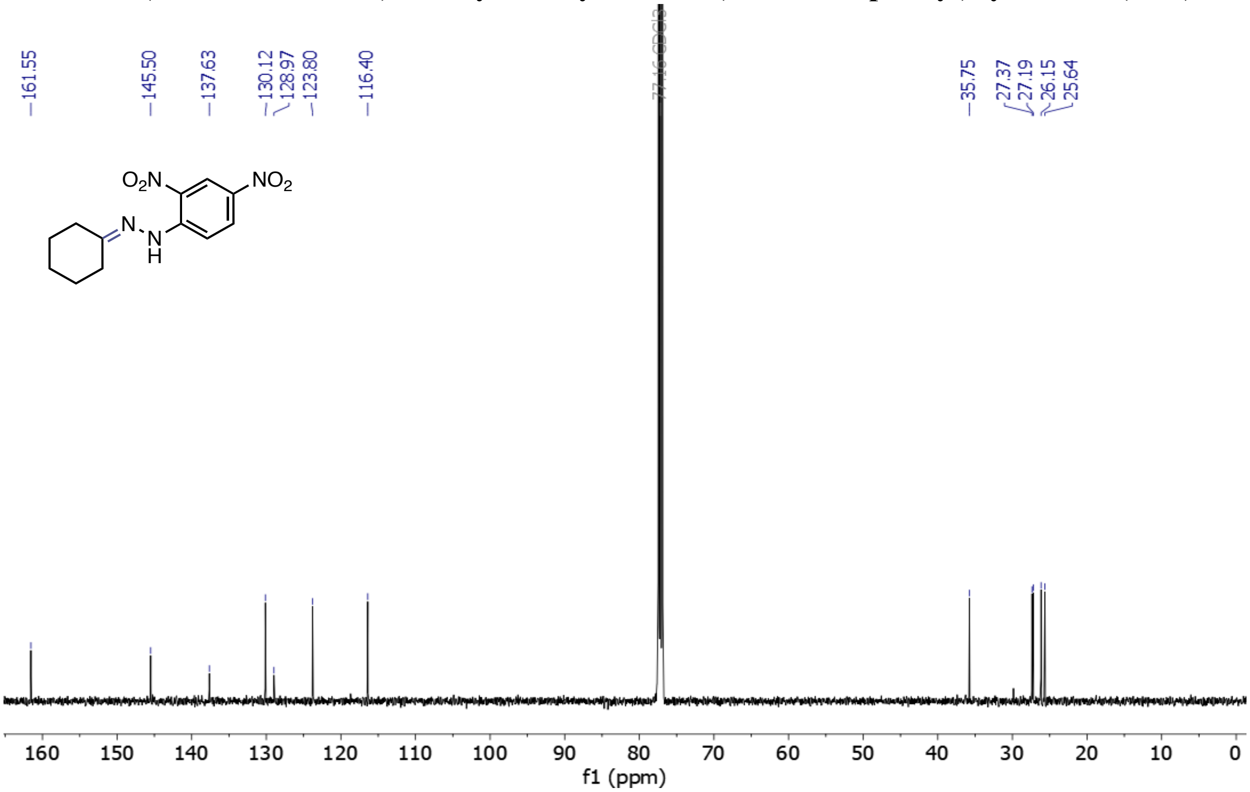

^{1}H NMR (400 MHz, $CDCl_3$) of 1-cycloheptylidene-2-(2,4-dinitrophenyl)hydrazine (3af')

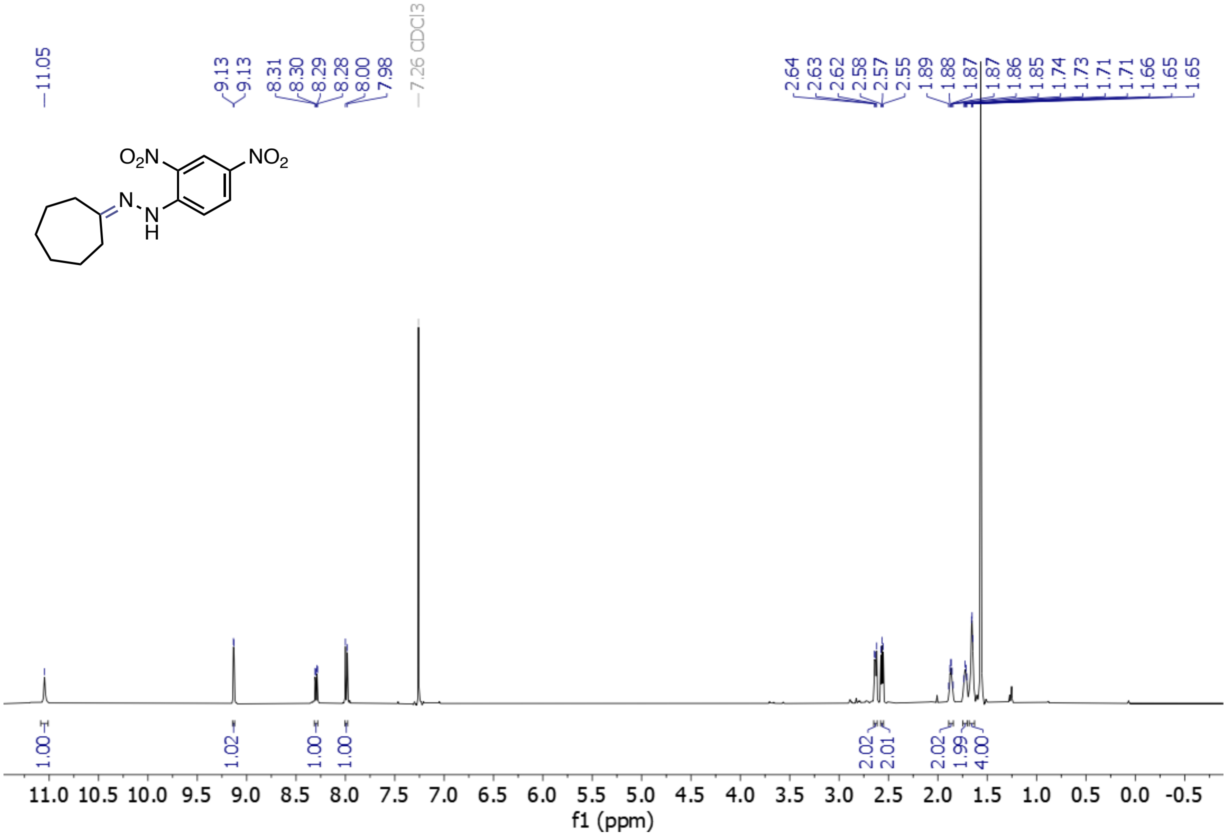

^{13}C NMR (101 MHz, $CDCl_3$) of 1-cycloheptylidene-2-(2,4-dinitrophenyl)hydrazine (3af')

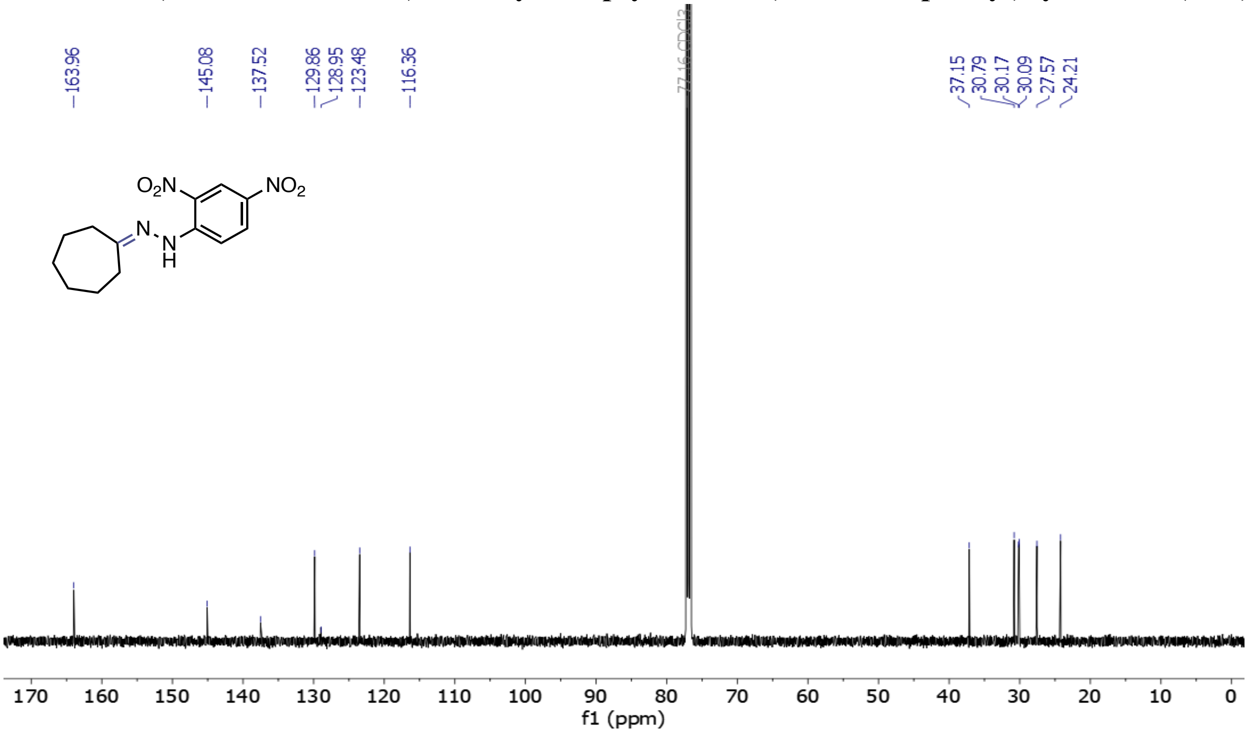

^{1}H NMR (400 MHz, $CDCl_3$) of Cyclooctanone (3ag)

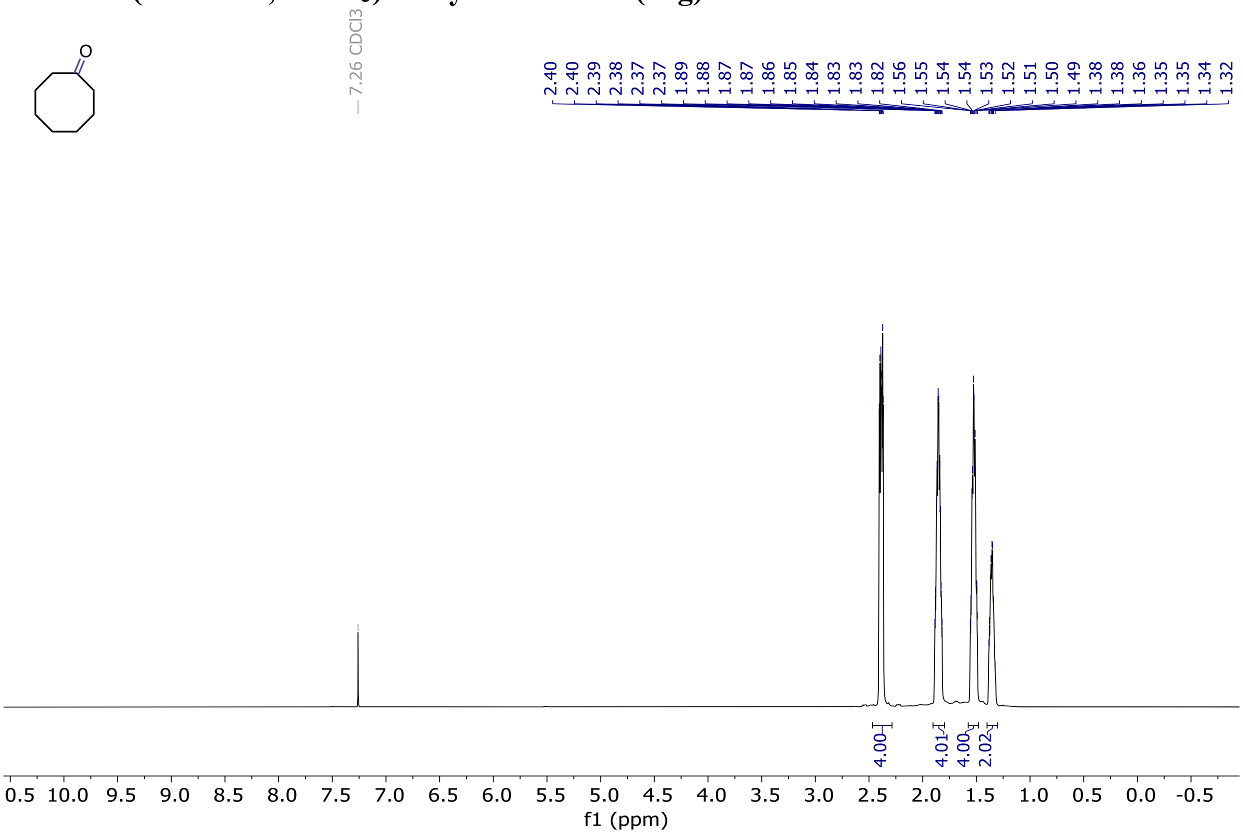

^{13}C NMR (101 MHz, $CDCl_3$) of Cyclooctanone (3ag)

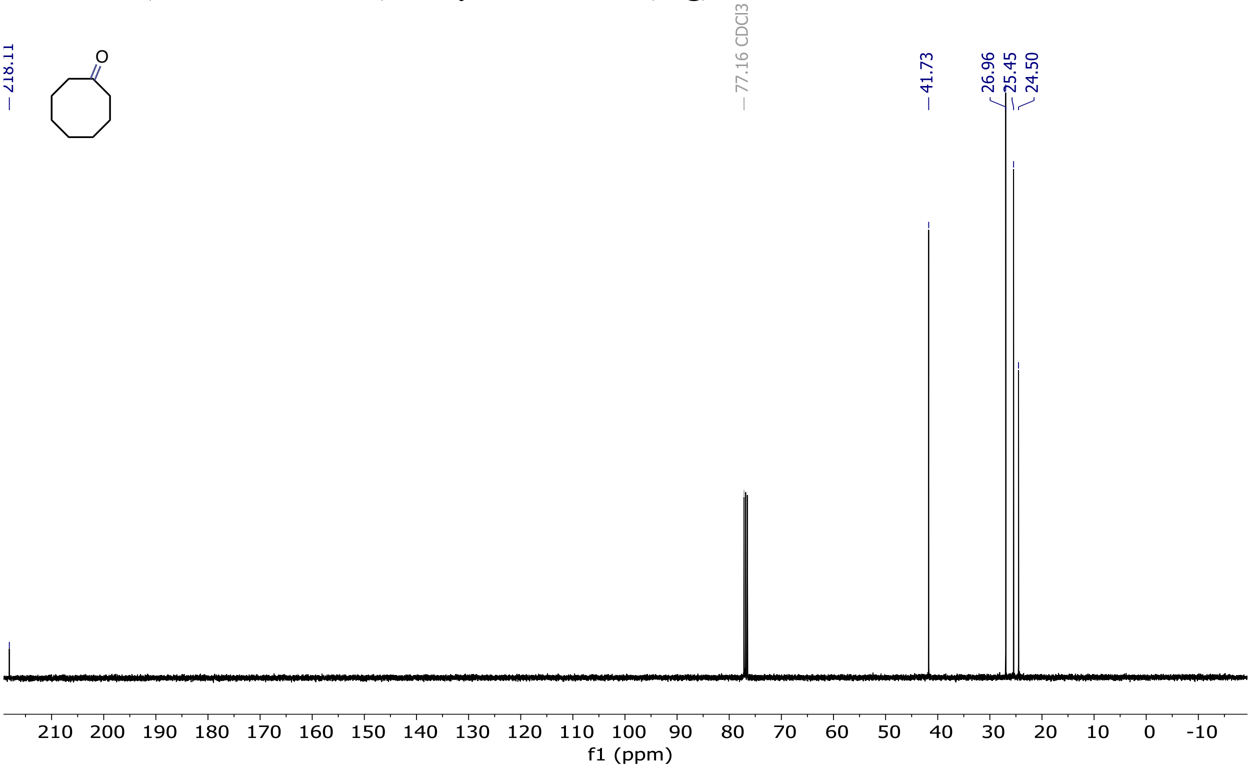

^{1}H NMR (500 MHz, $CDCl_3$) of Cyclododecanone (3ah)

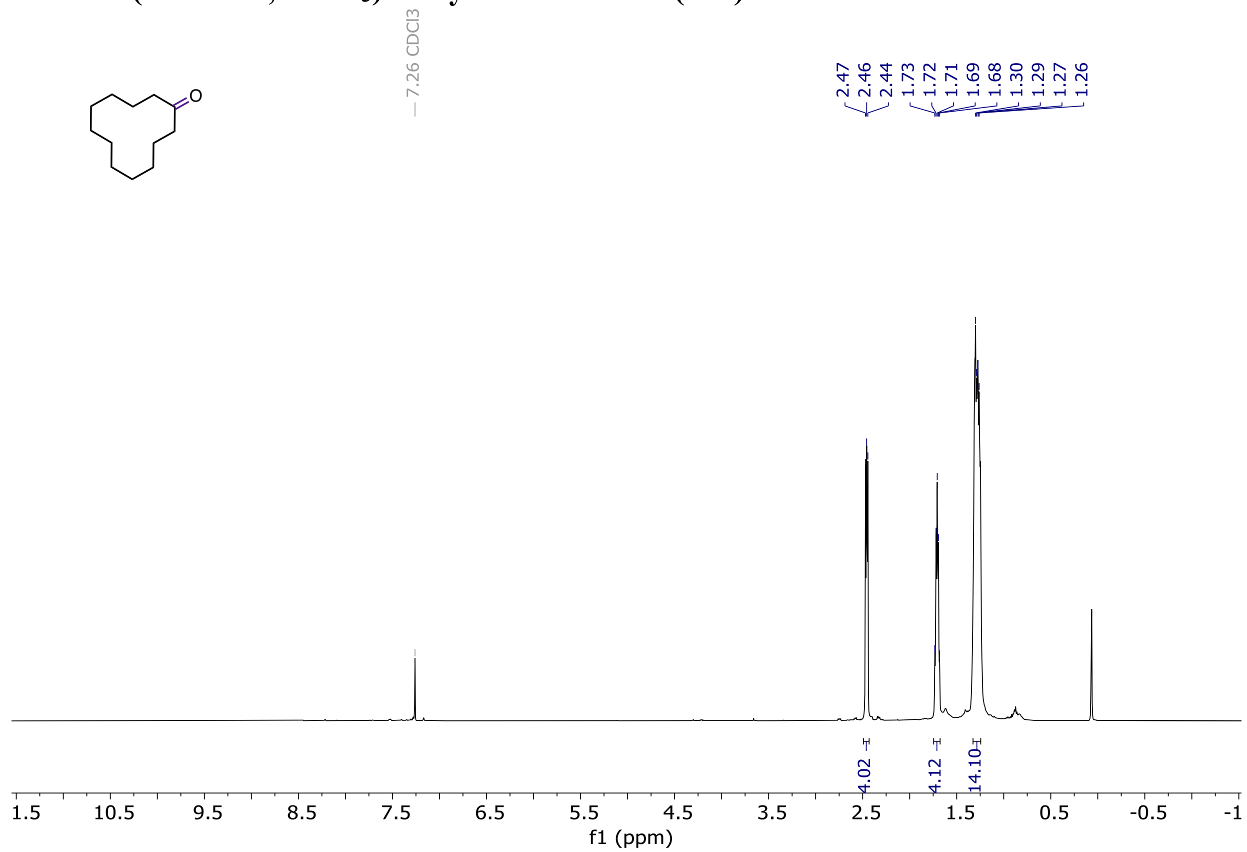

^{13}C NMR (101 MHz, $CDCl_3$) of Cyclododecanone (3ah)

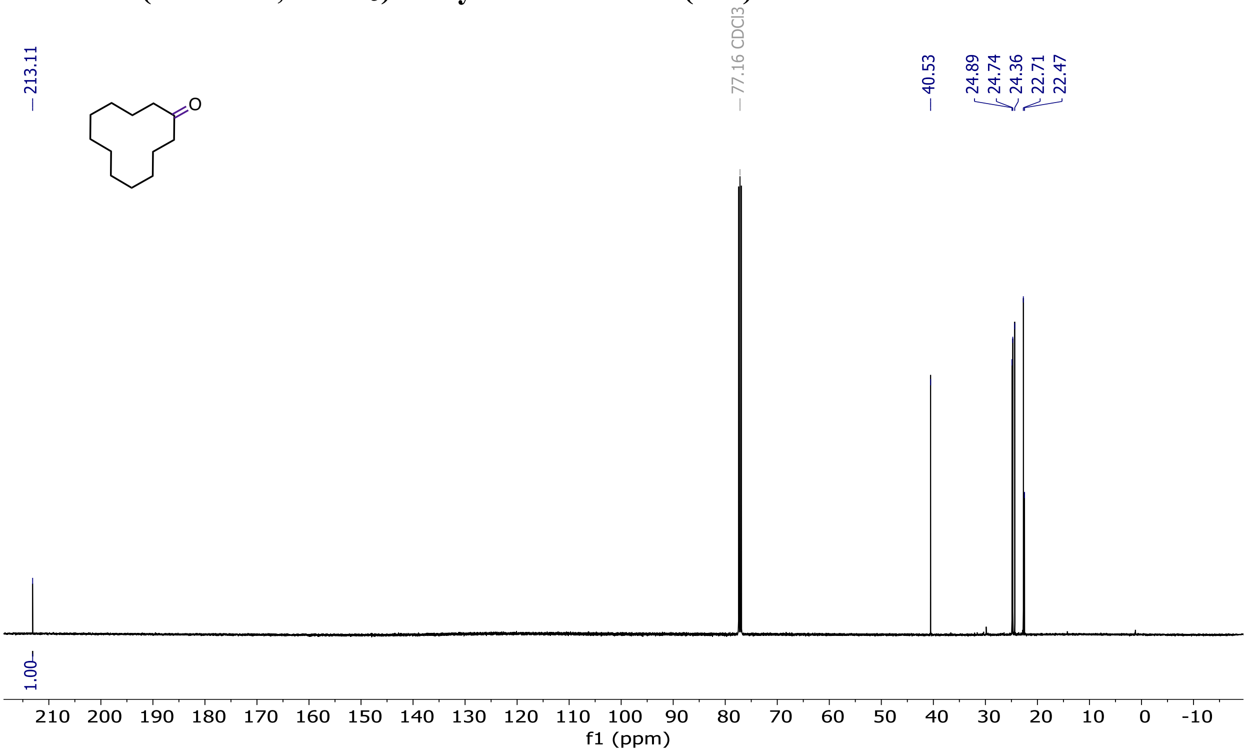

^{1}H NMR (500 MHz, $CDCl_3$) of 4-(tert-butyl)cyclohexan-1-one (3ai)

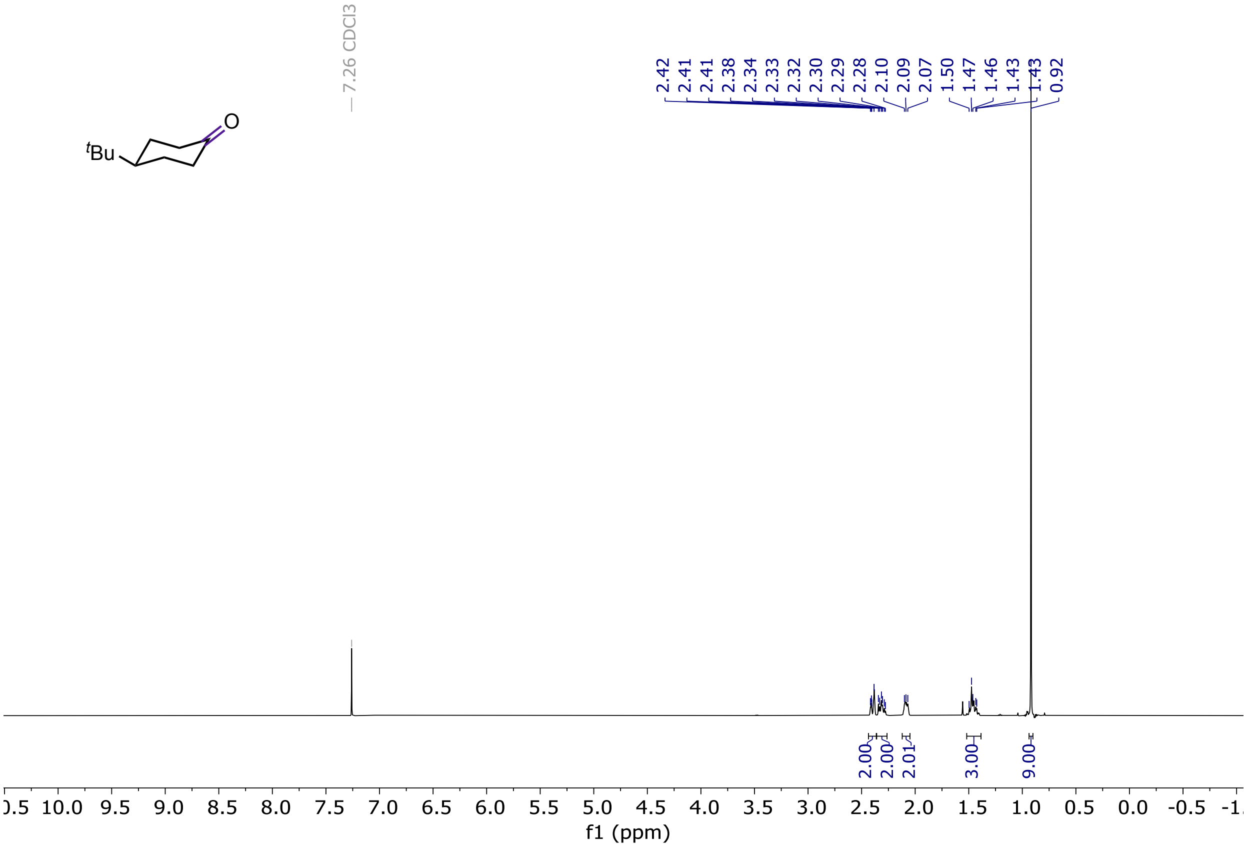

^{13}C NMR (126 MHz, $CDCl_3$) of 4-(tert-butyl)cyclohexan-1-one (3ai)

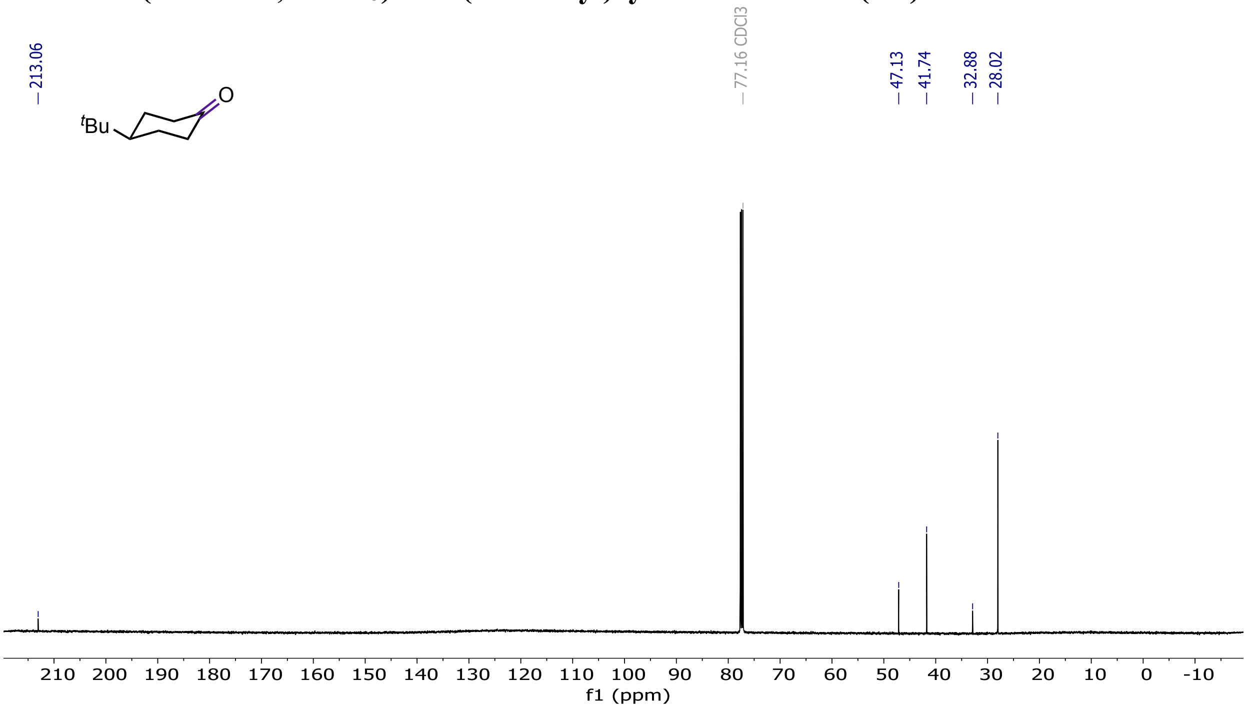

^{1}H NMR (500 MHz, CDCl$_3$) of 4-phenylcyclohexan-1-one (3aj)

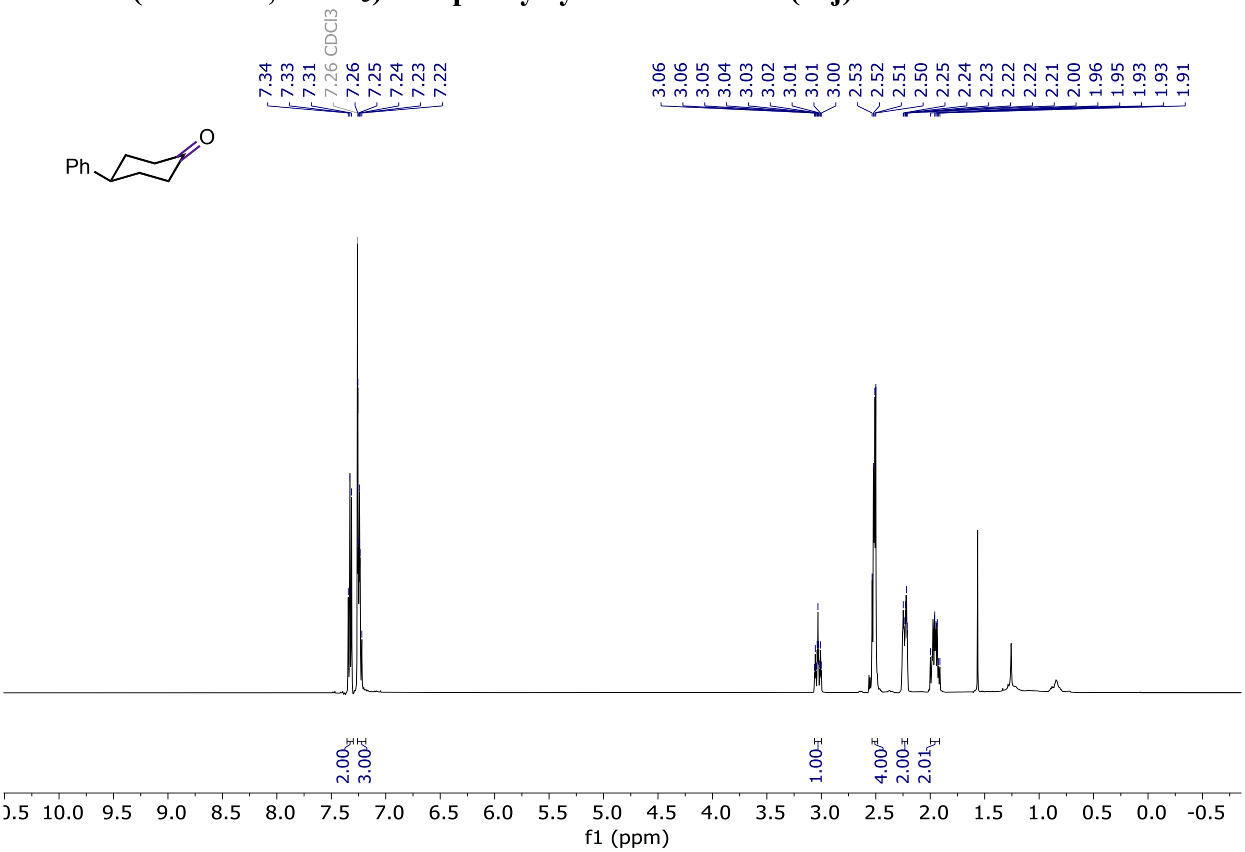

^{13}C NMR (126 MHz, CDCl$_3$) of 4-phenylcyclohexan-1-one (3aj)

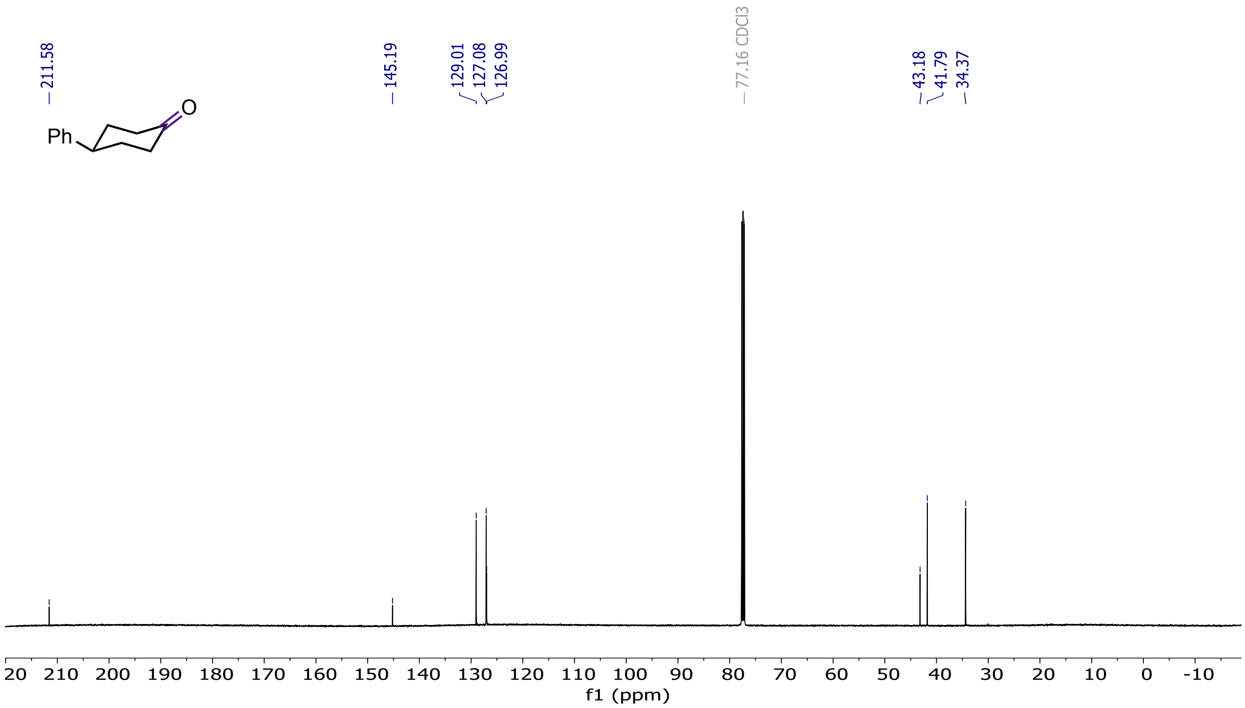

^{1}H NMR (500 MHz, $CDCl_3$) of (2R,5R)-2-isopropyl-5-methylcyclohexan-1-one (3ak)

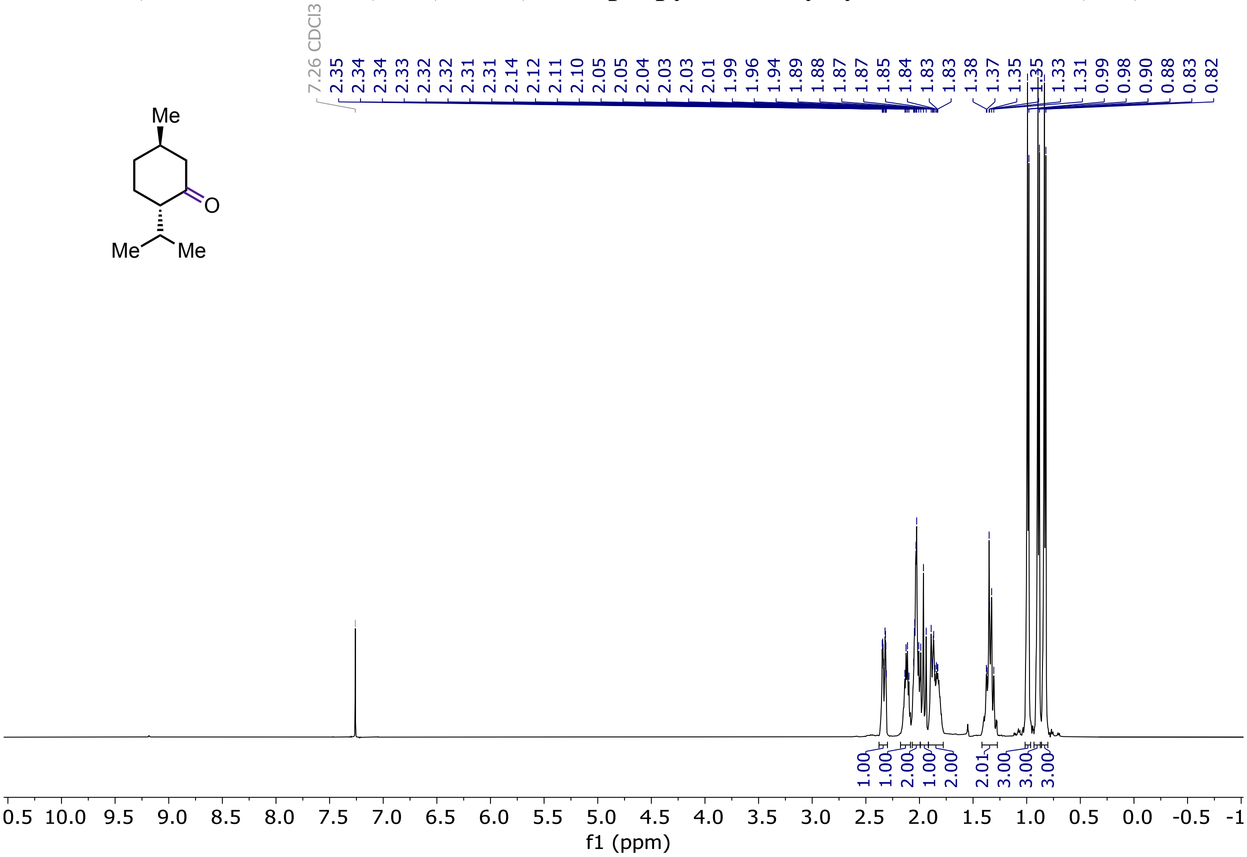

^{13}C NMR (126 MHz, $CDCl_3$) of (2R,5R)-2-isopropyl-5-methylcyclohexan-1-one (3ak)

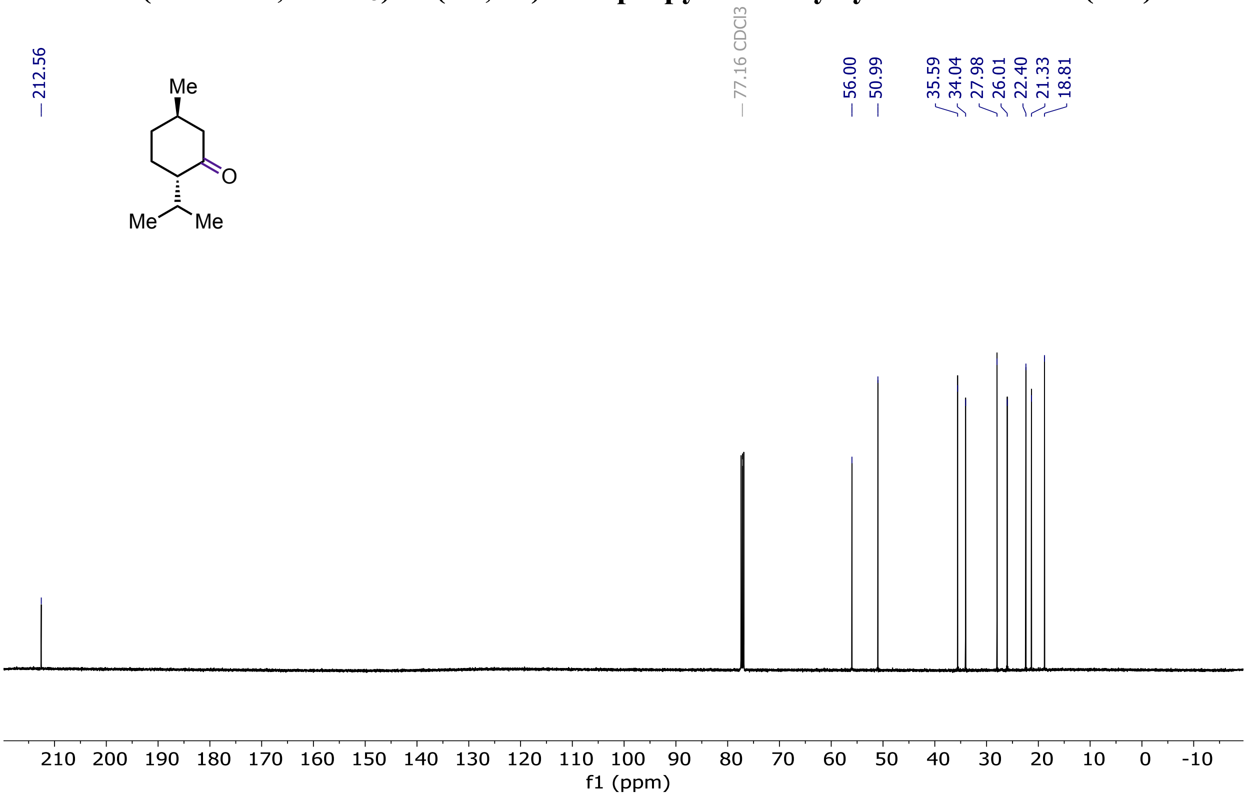

^{1}H NMR (400 MHz, CDCl$_3$) of (1S,4R,5R)-1-isopropyl-4-methylbicyclo[3.1.0]hexan-3-one (3al)

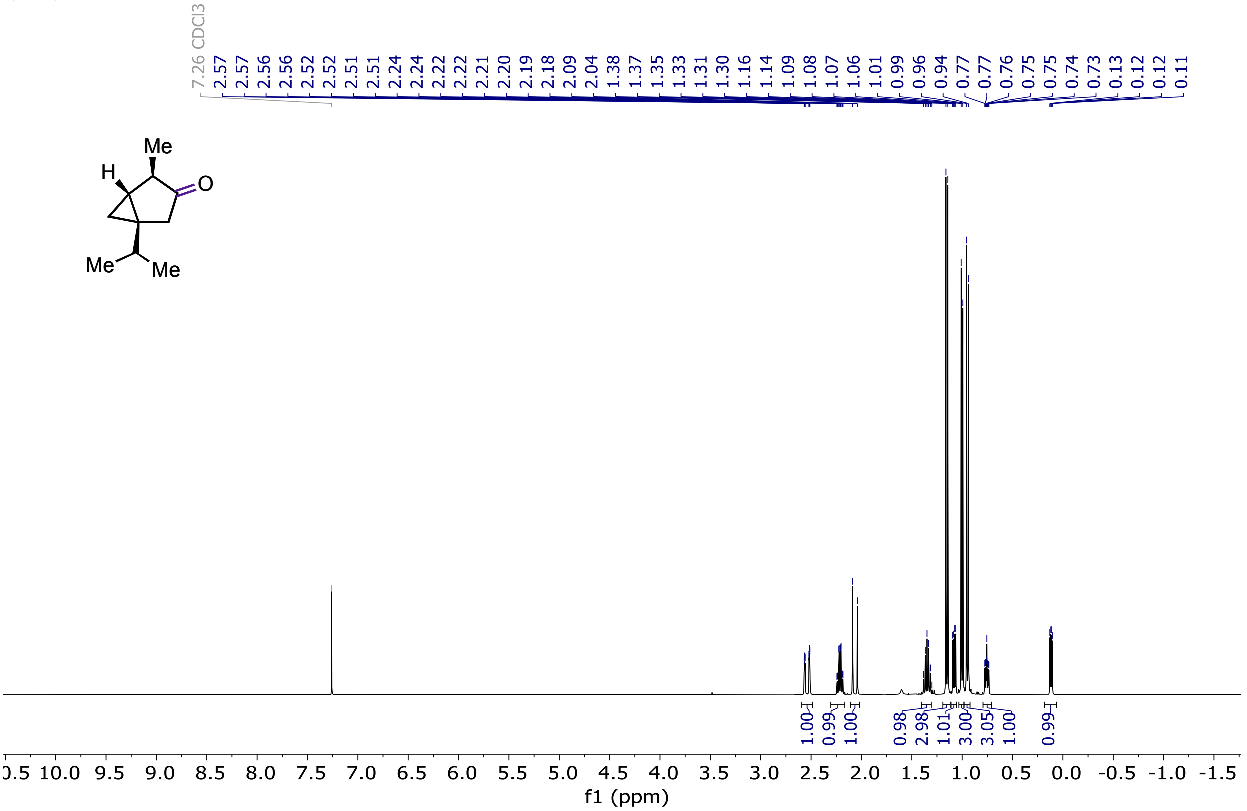

^{13}C NMR (126 MHz, CDCl$_3$) of (1S,4R,5R)-1-isopropyl-4-methylbicyclo[3.1.0]hexan-3-one (3al)

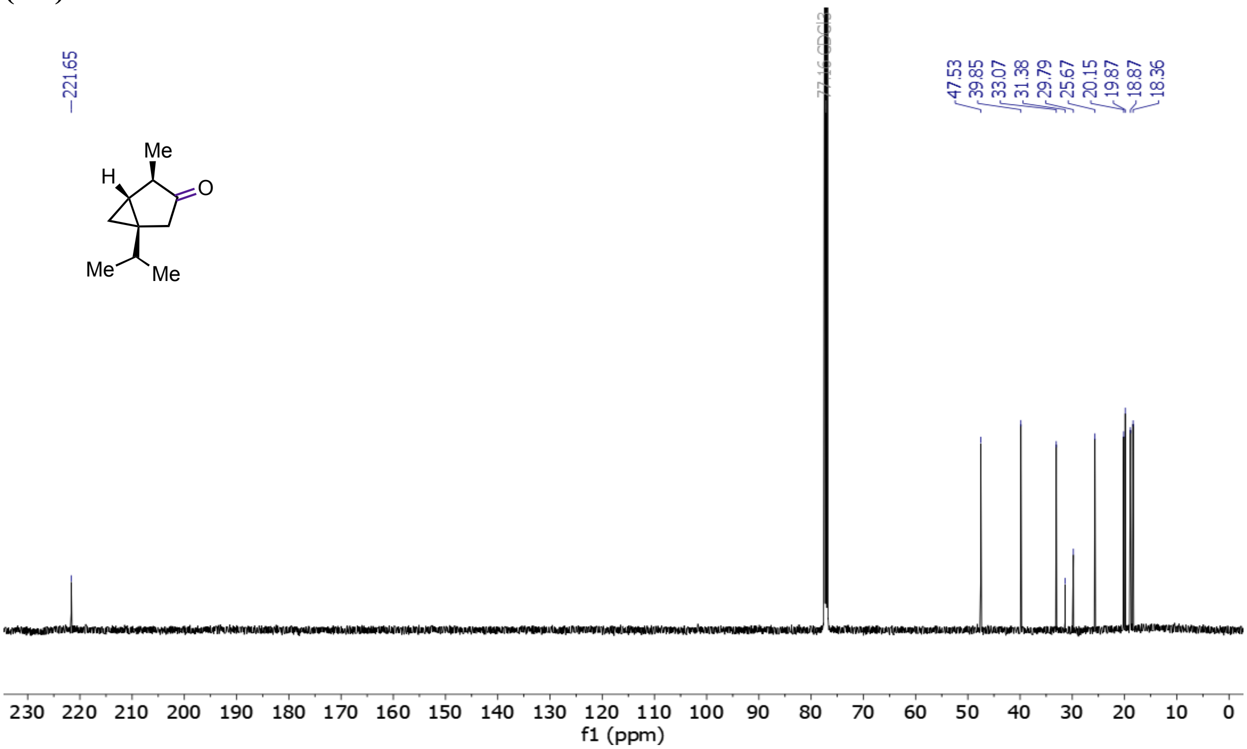

^{1}H NMR (500 MHz, $CDCl_3$) of (1S,4R)-1,7,7-trimethylbicyclo[2.2.1]heptan-2-one (3am)

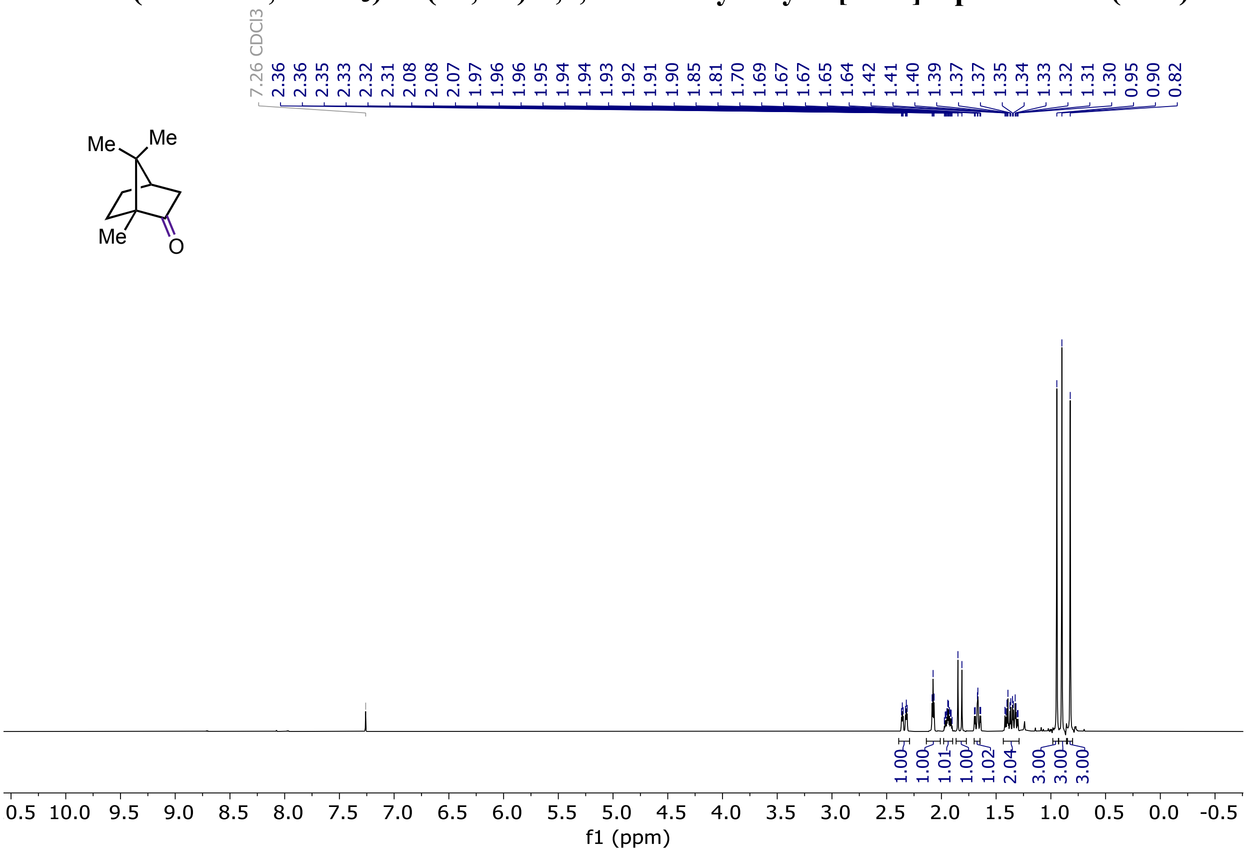

^{13}C NMR (126 MHz, $CDCl_3$) (1S,4R)-1,7,7-trimethylbicyclo[2.2.1]heptan-2-one (3am)

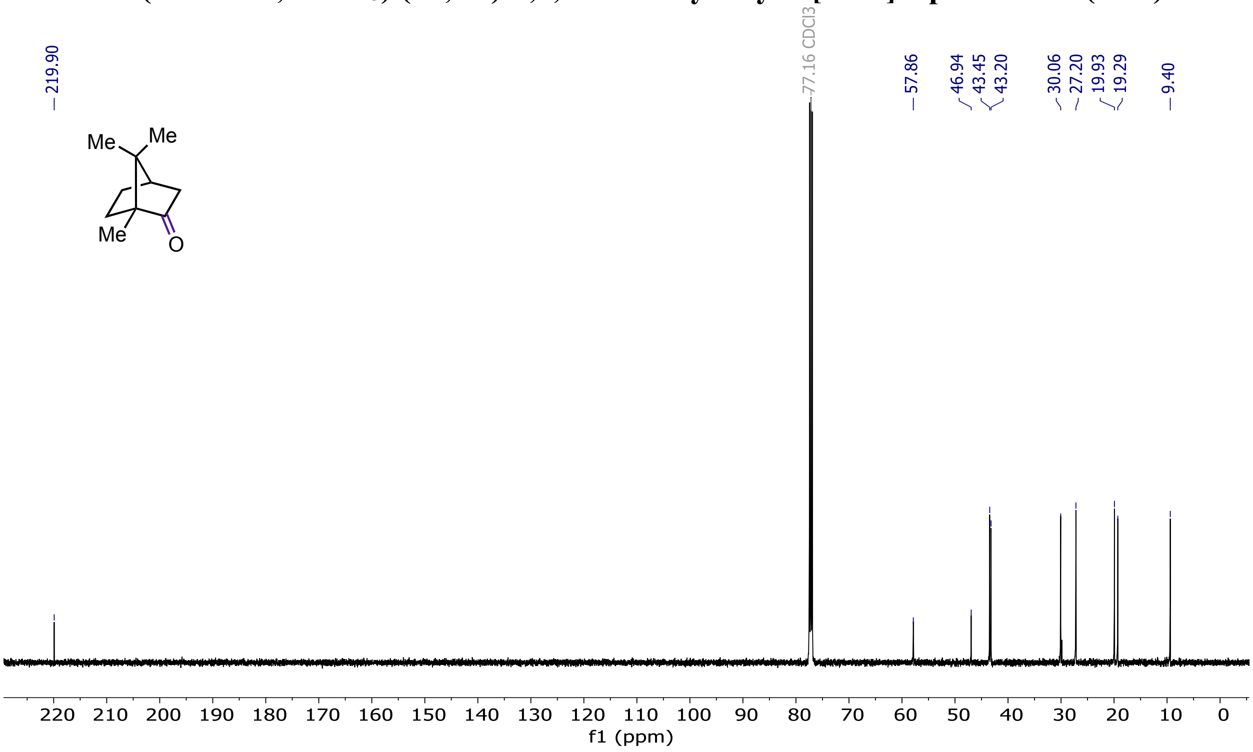

¹H NMR (400 MHz, CDCl₃) of (3aR,4S,7S,7aR)-octahydro-5H-4,7-methanoinden-5-one (3an)

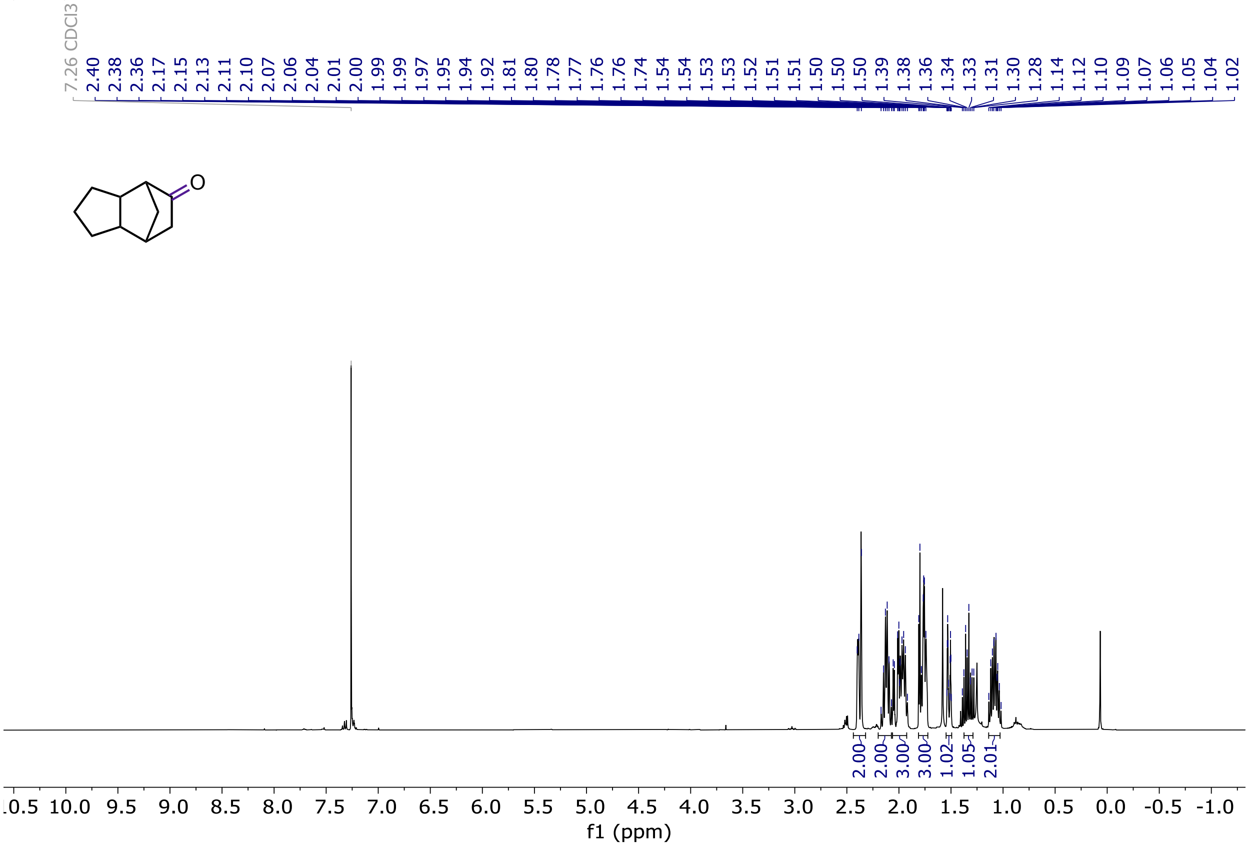

¹³C NMR (101 MHz, CDCl₃) of (3a*R*,4*S*,7*S*,7a*R*)-octahydro-5H-4,7-methanoinden-5-one (3an)

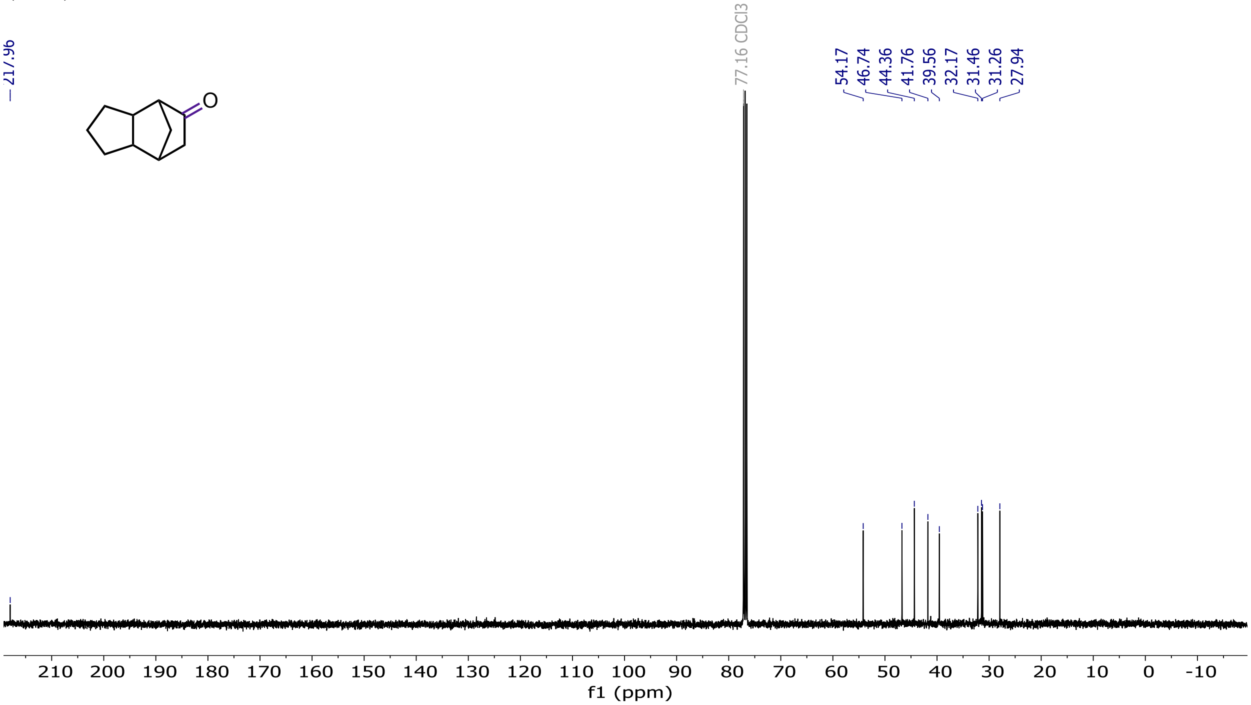

^{1}H NMR (500 MHz, CDCl$_3$) of (8*R*,9*S*,10*S*,13*S*,14*S*)-10,13-dimethyltetradecahydro-3*H*-cyclopenta[a]phenanthrene-3,17(2*H*)-dione (3ao)

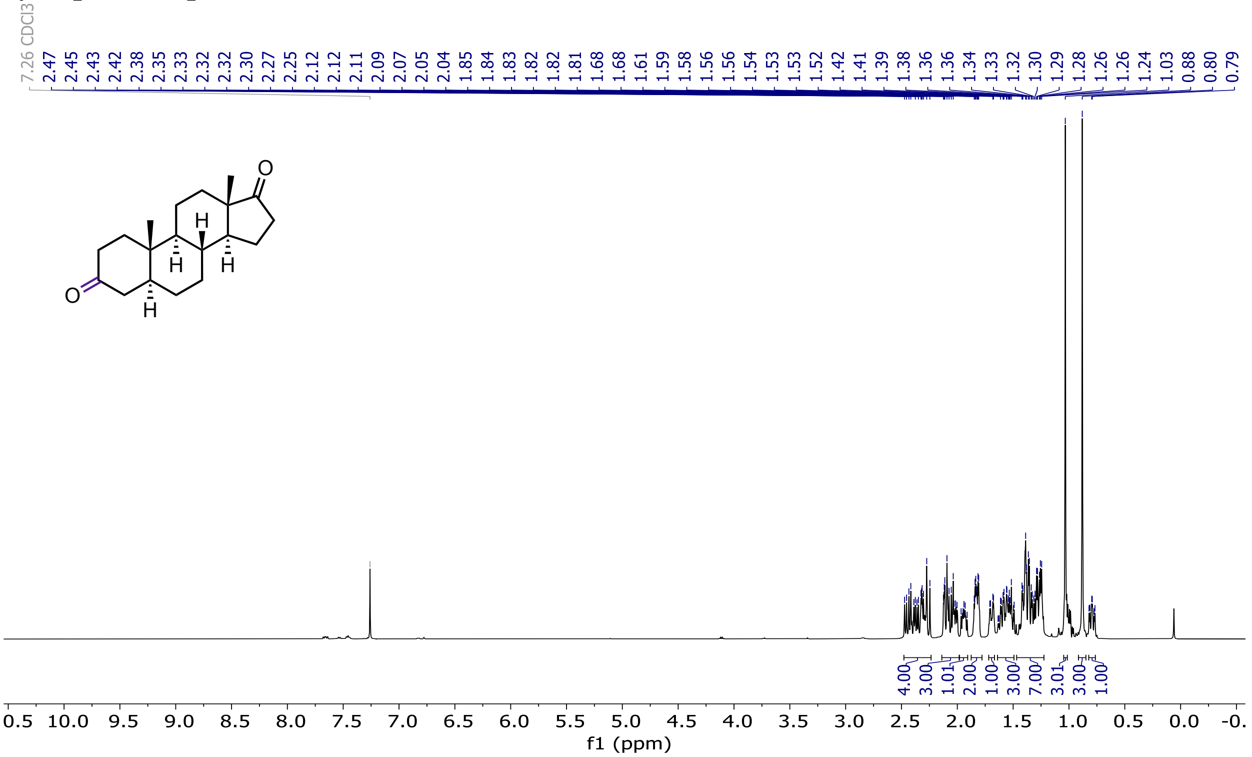

^{13}C NMR (126 MHz, CDCl$_3$) of (8*R*,9*S*,10*S*,13*S*,14*S*)-10,13-dimethyltetradecahydro-3*H*-cyclopenta[a]phenanthrene-3,17(2*H*)-dione (3ao)

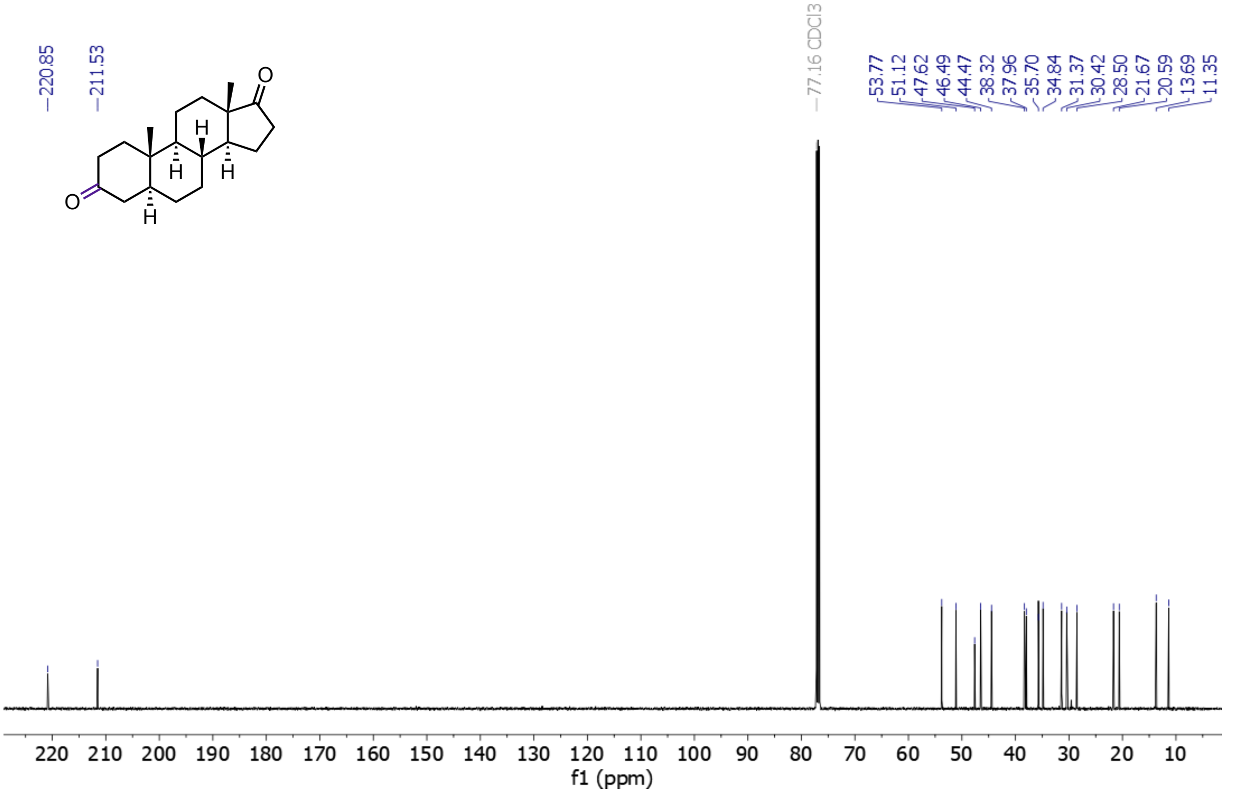

^{1}H NMR (500 MHz, CDCl$_3$) of (*E*)-*N*-Benzyl-1-phenylmethanimine (4a)

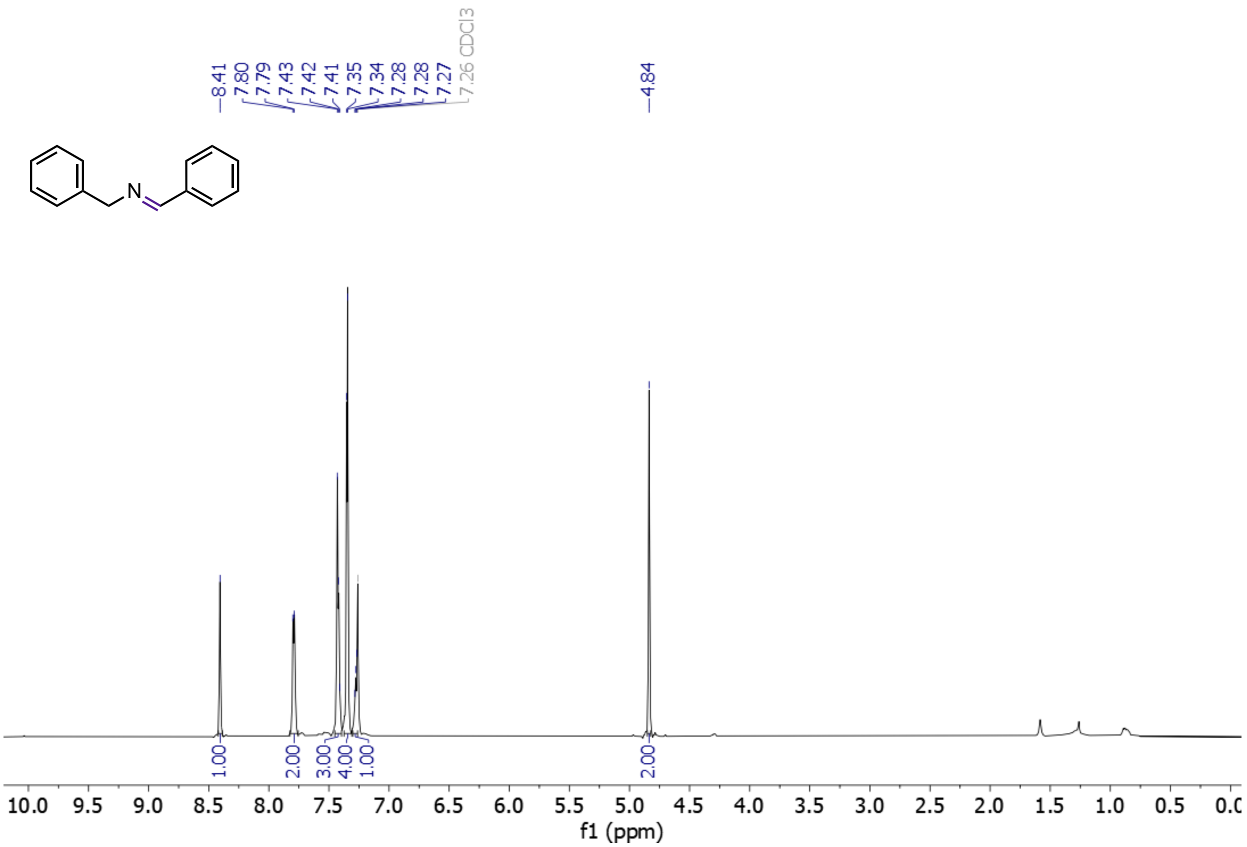

^{13}C NMR (126 MHz, CDCl$_3$) of (*E*)-*N*-Benzyl-1-phenylmethanimine (4a)

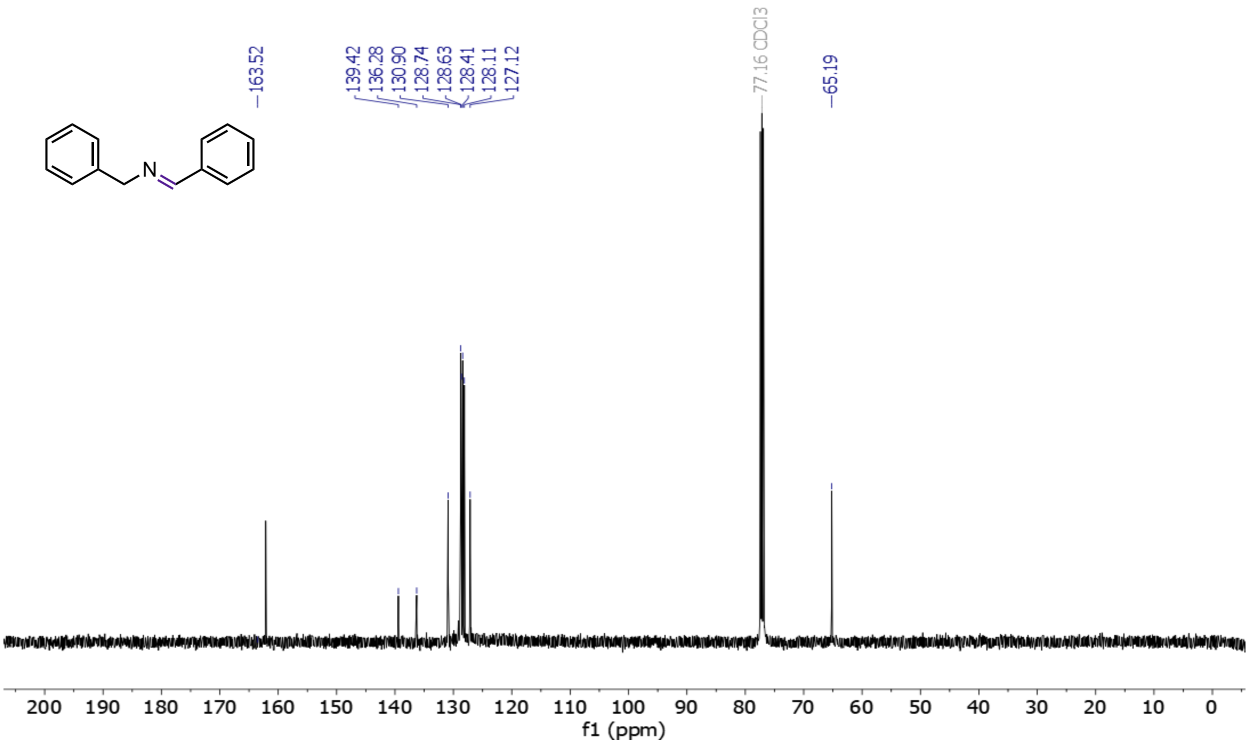

^{1}H NMR (500 MHz, $CDCl_3$) of (*E*)-*N*-1-diphenylmethanimine (4b)

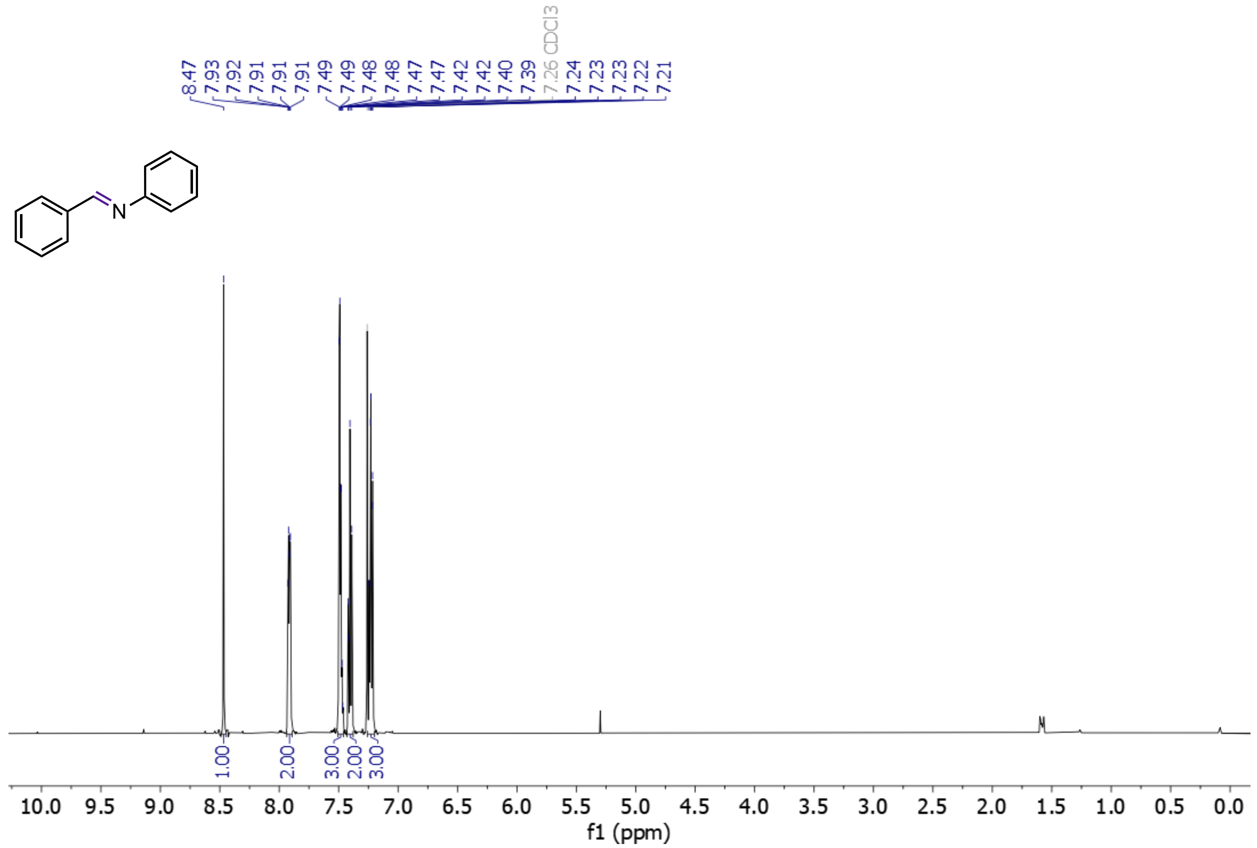

^{3}C NMR (126 MHz, $CDCl_3$) of (*E*)-*N*-1-diphenylmethanimine (4b)

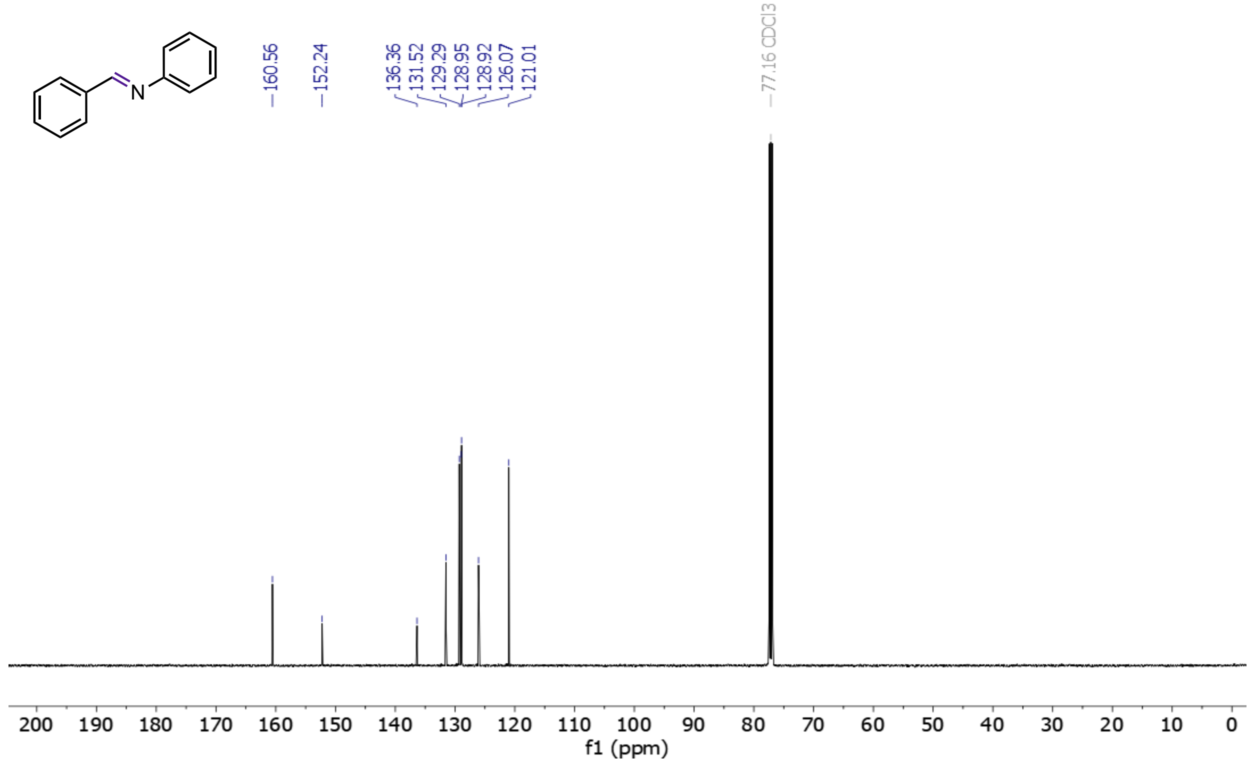

^{1}H NMR (500 MHz, CDCl$_3$) for 4,4-dimethyl-3,4-dihydroisoquinoline (4f)

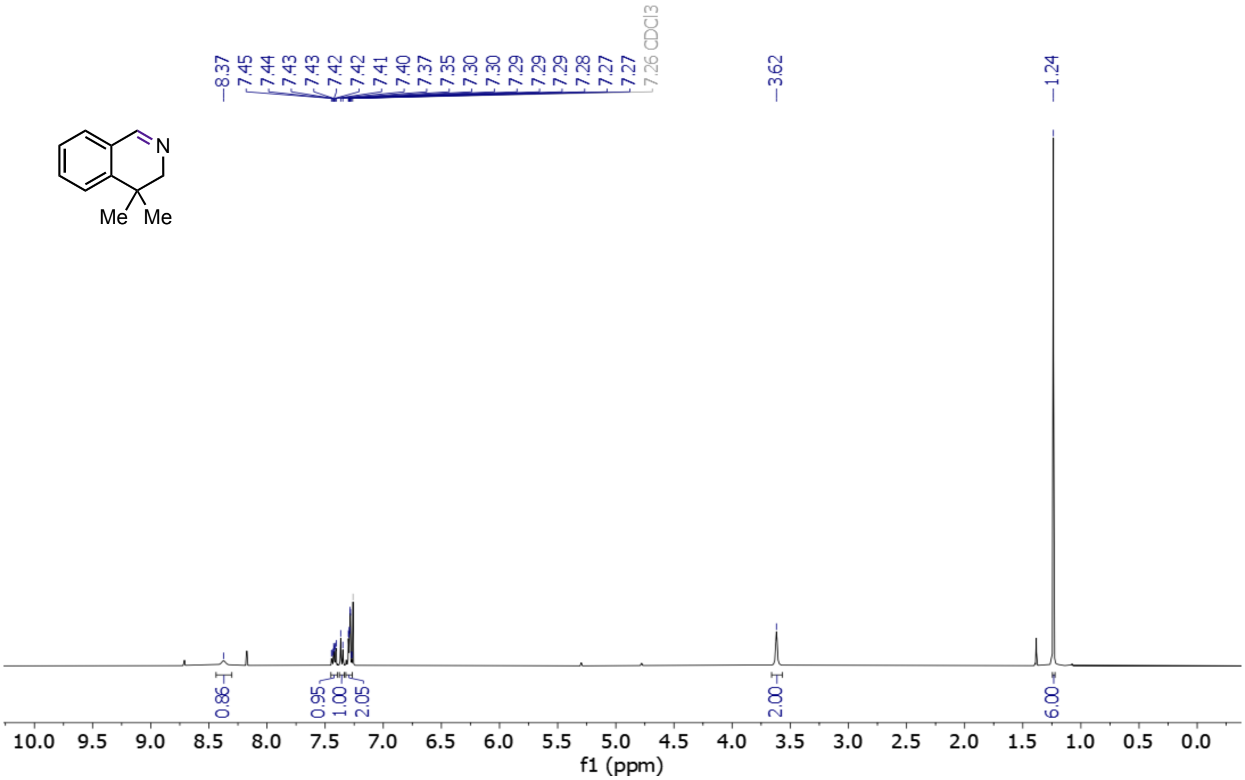

^{13}C NMR (101 MHz, CDCl$_3$) of 4,4-dimethyl-3,4-dihydroisoquinoline (4f)

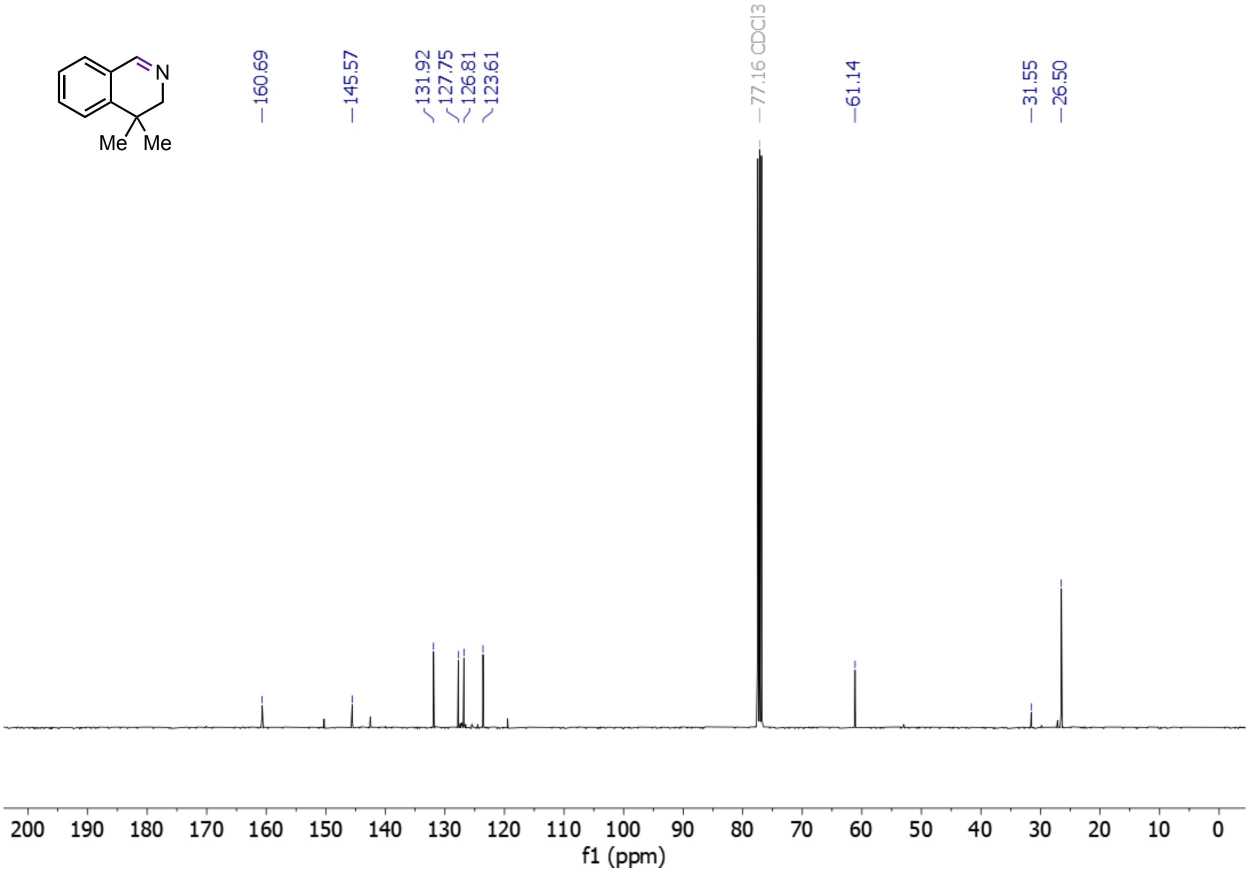

^{1}H NMR (500 MHz, CDCl3) of Benzoic acid (10a)

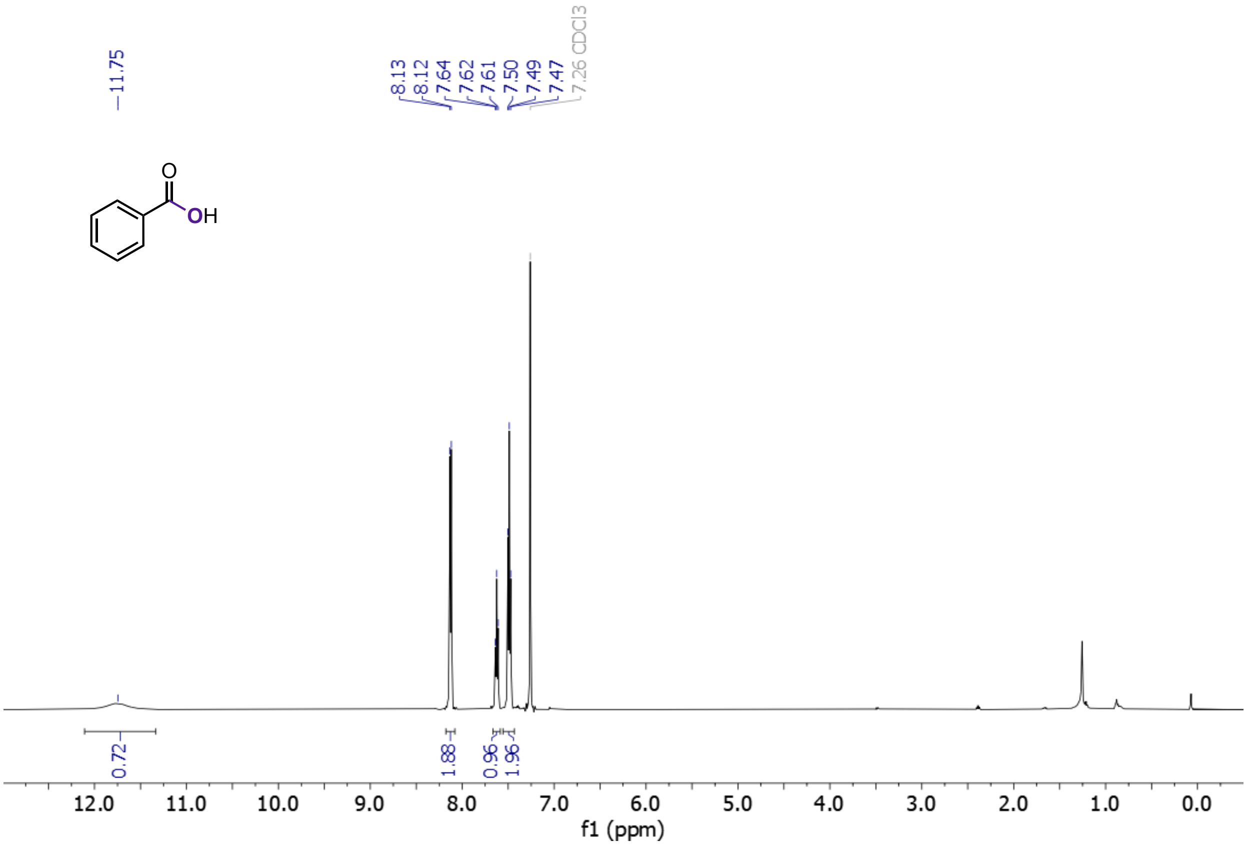

^{13}C NMR (101 MHz, CDCl3) of Benzoic acid (10a)

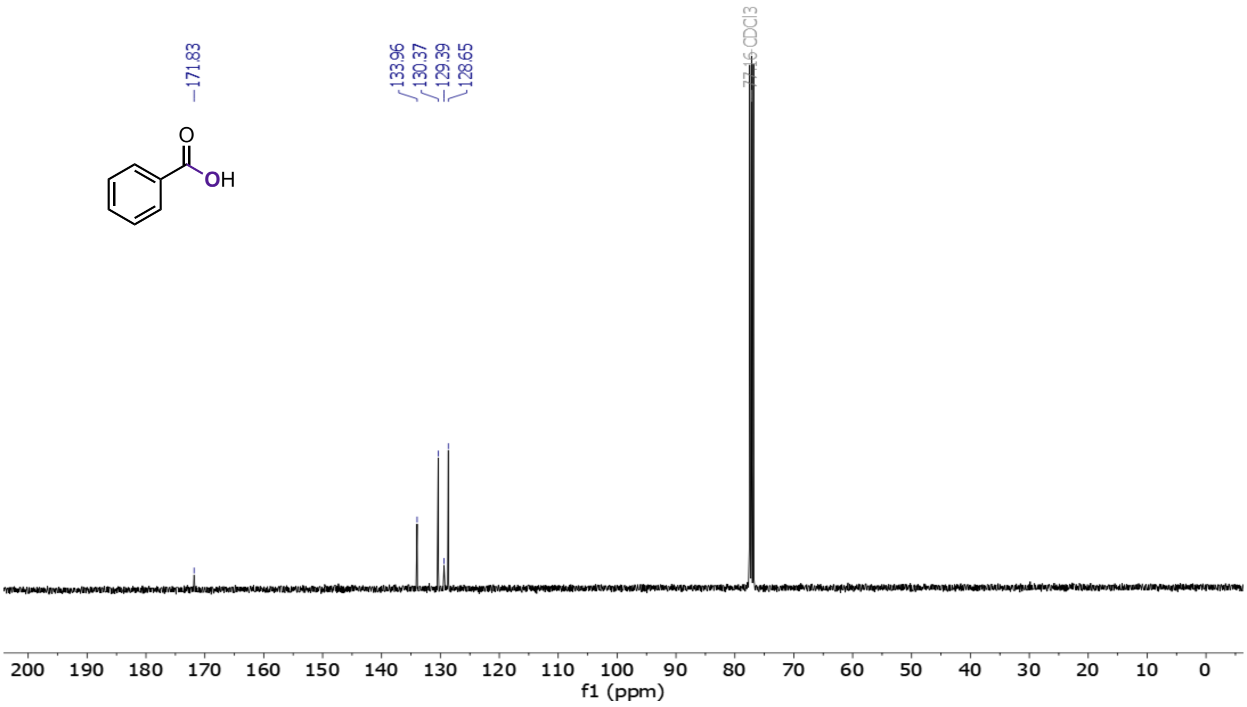

^{1}H NMR (400 MHz, $CDCl_3$) of 4-(*tert*-Butyl)benzoic acid (10b)

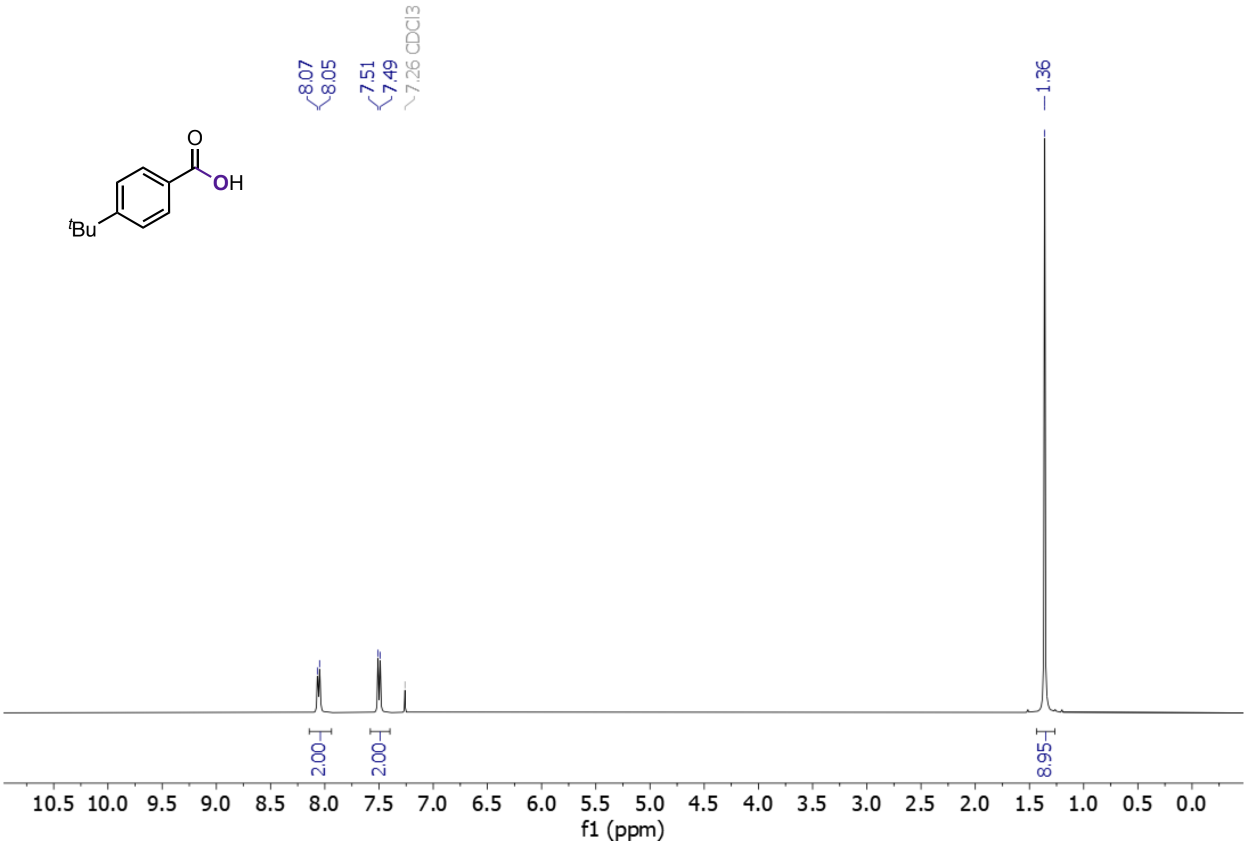

^{13}C NMR (101 MHz, $CDCl_3$) of 4-(*tert*-Butyl)benzoic acid (10b)

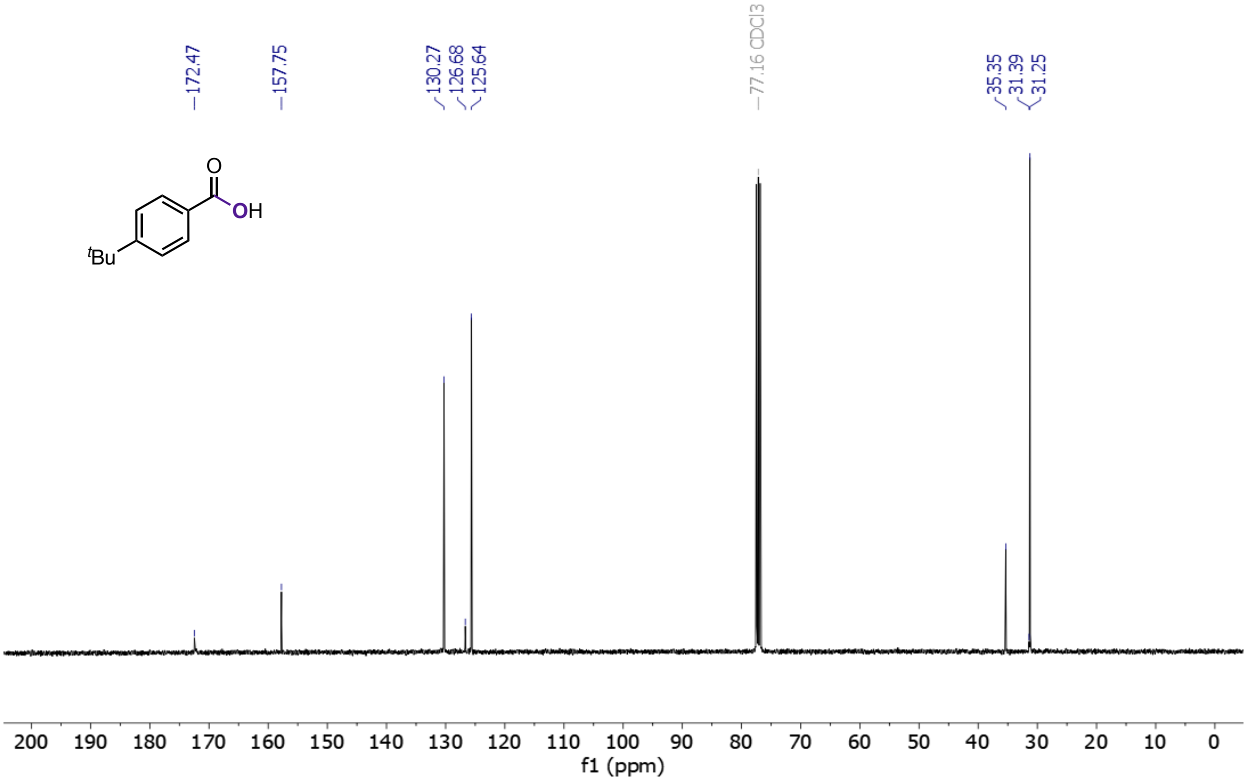

^{1}H NMR (500 MHz, $CDCl_3$) of 4-Methoxybenzoic acid (10c)

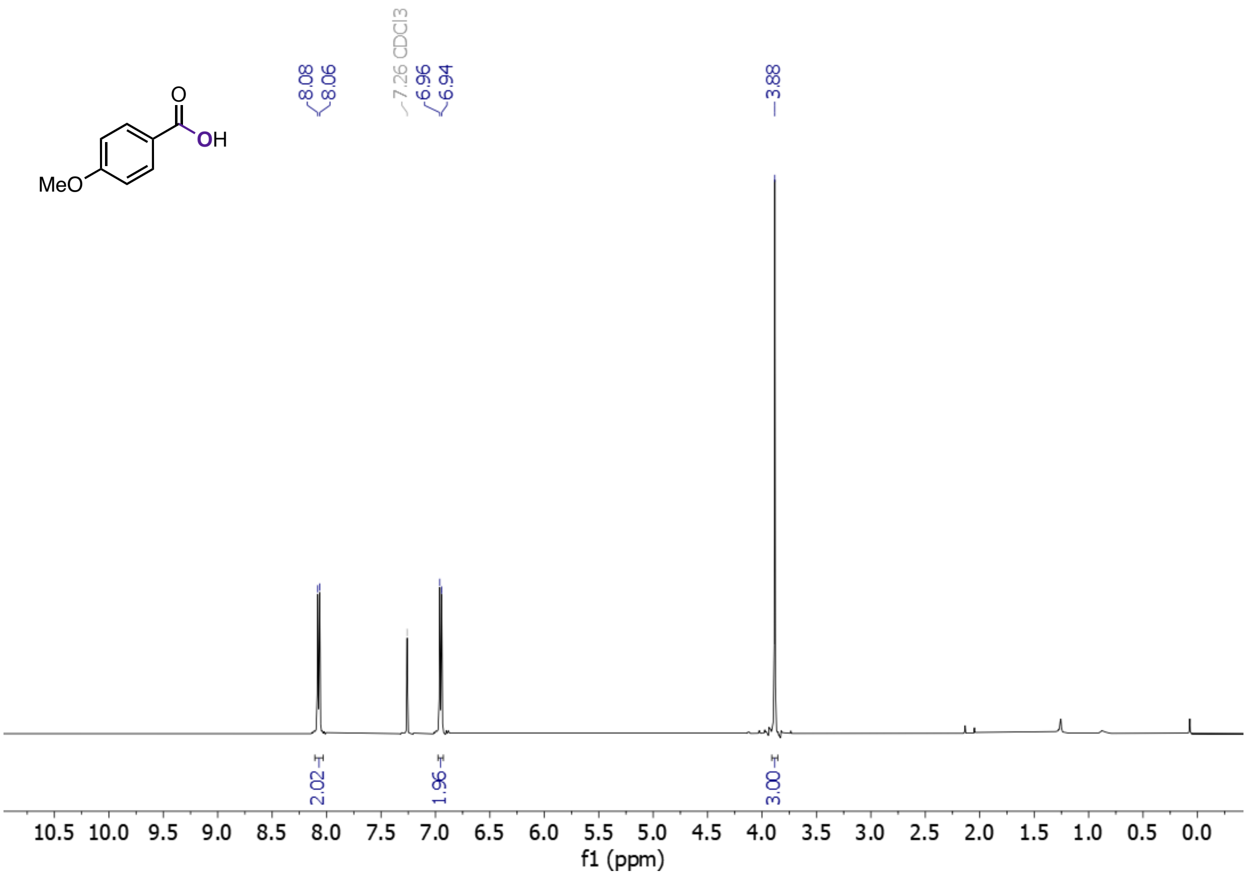

^{13}C NMR (126 MHz, $CDCl_3$) of 4-Methoxybenzoic acid (10c)

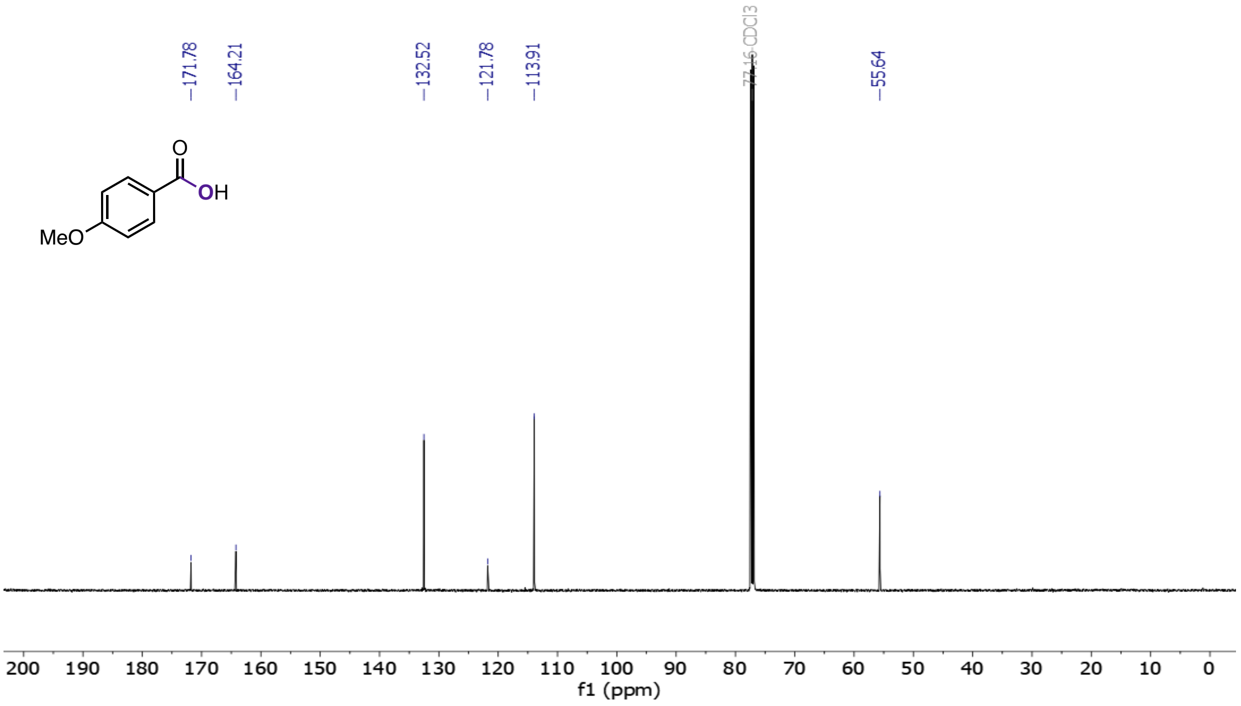

^{1}H NMR (500 MHz, $CDCl_3$) of 4-Fluorobenzoic acid (10d)

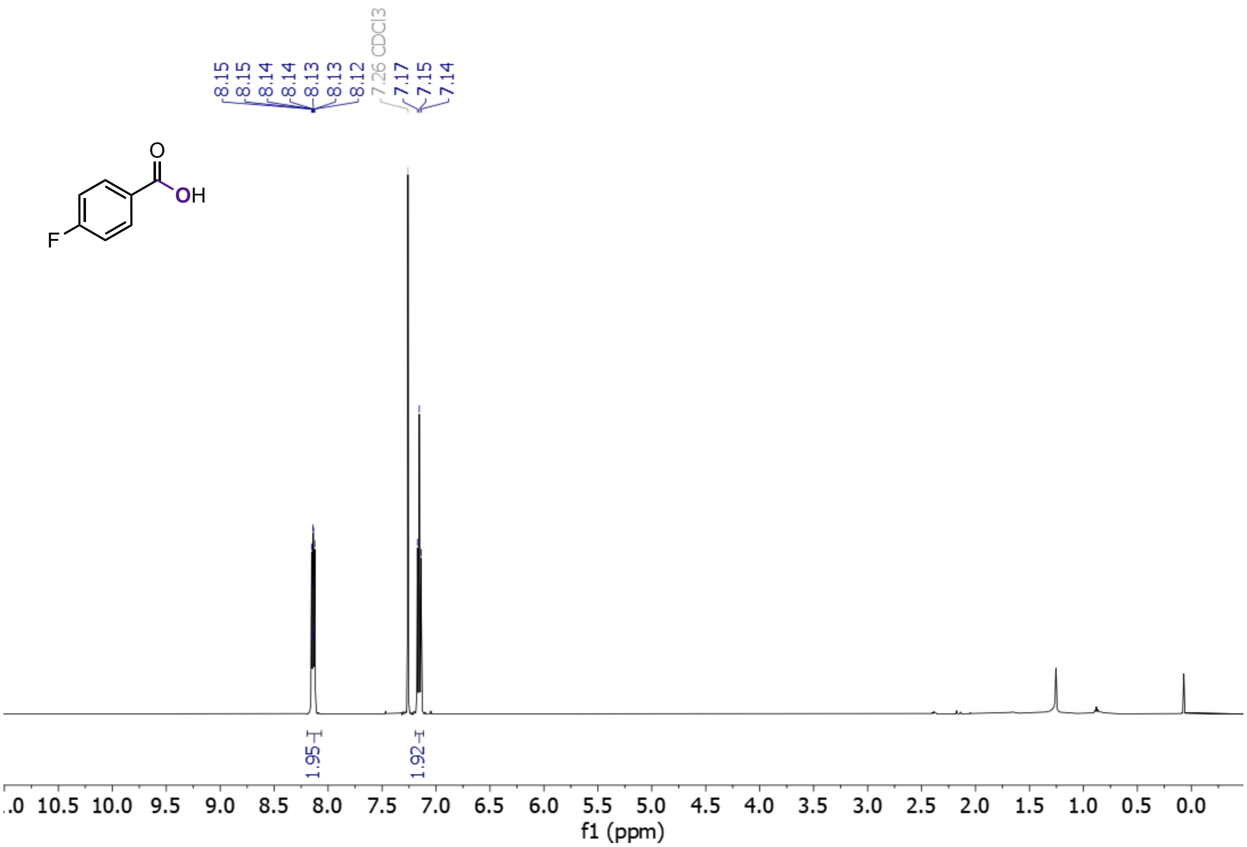

^{13}C NMR (126 MHz, $CDCl_3$) of 4-Fluorobenzoic acid (10d)

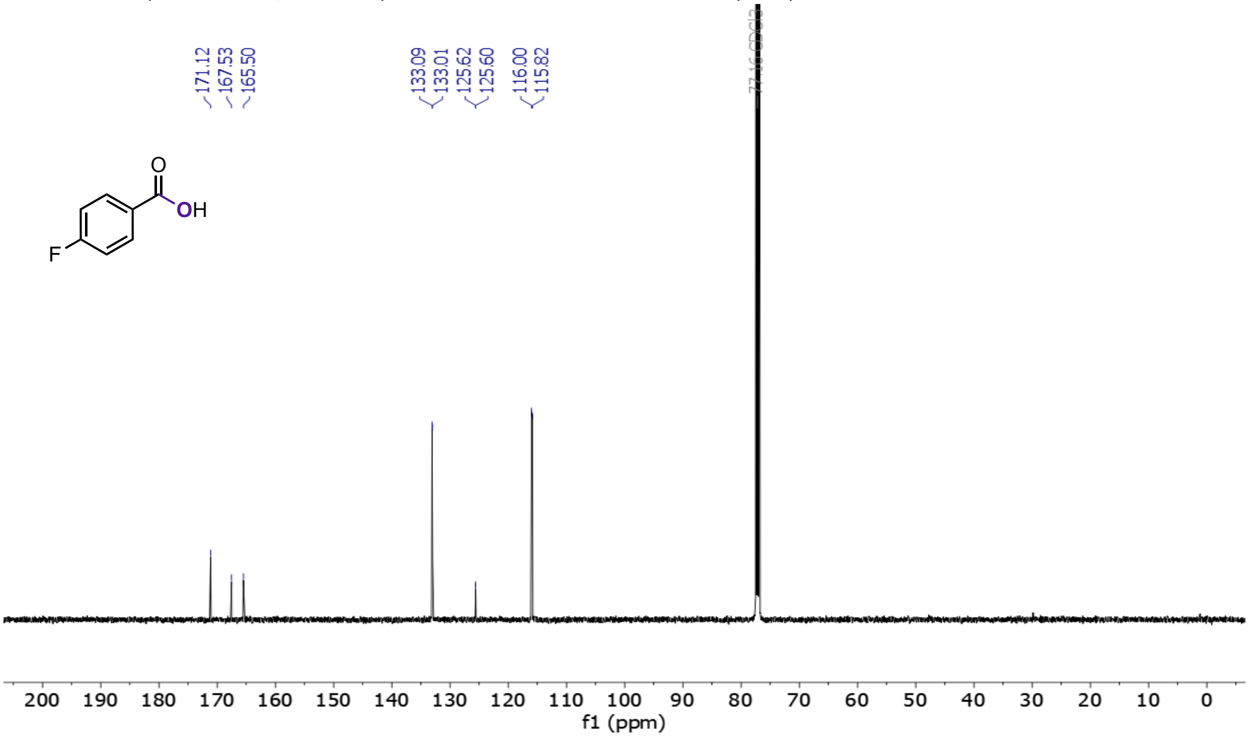

^1H NMR (500 MHz, $CDCl_3$) of 4-Cyanobenzoic acid (10e)

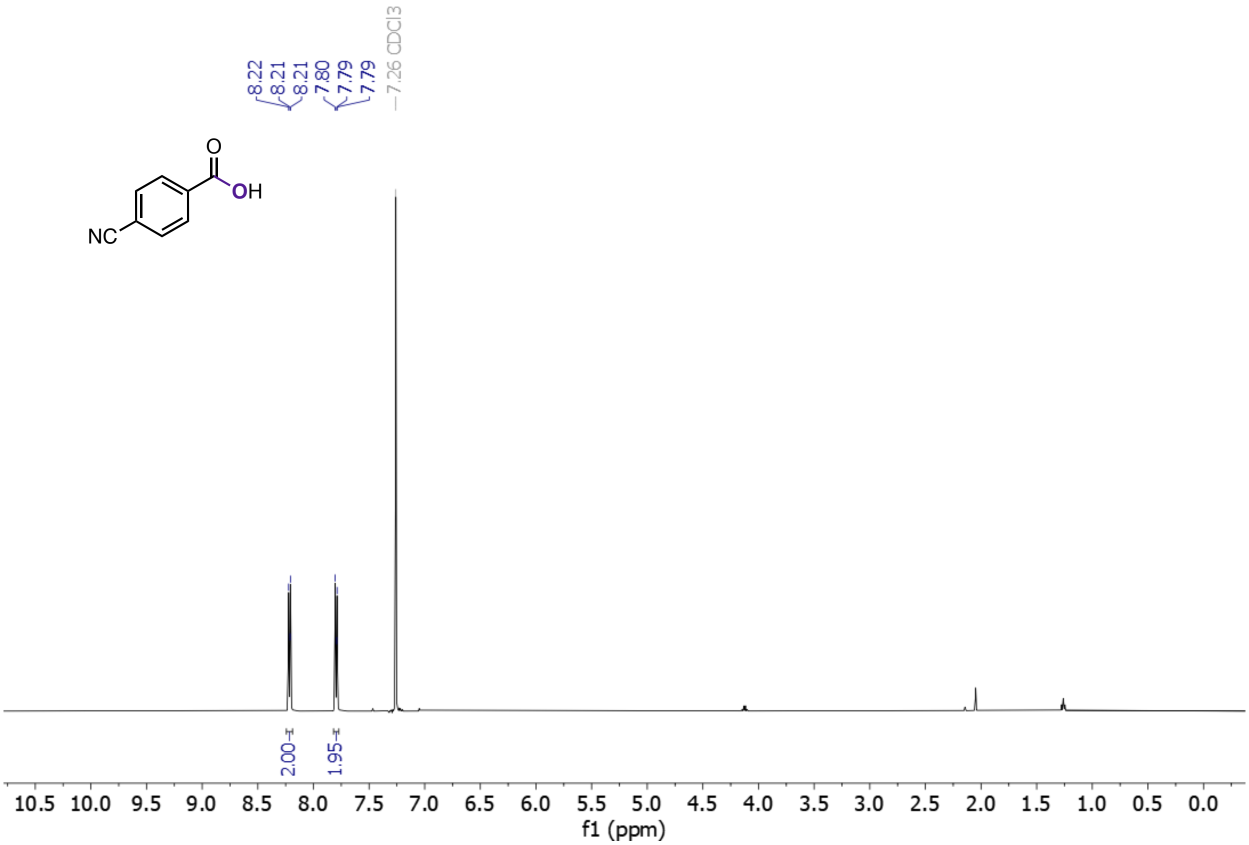

^{13}C NMR (126 MHz, $CDCl_3$) of 4-Cyanobenzoic acid (10e)

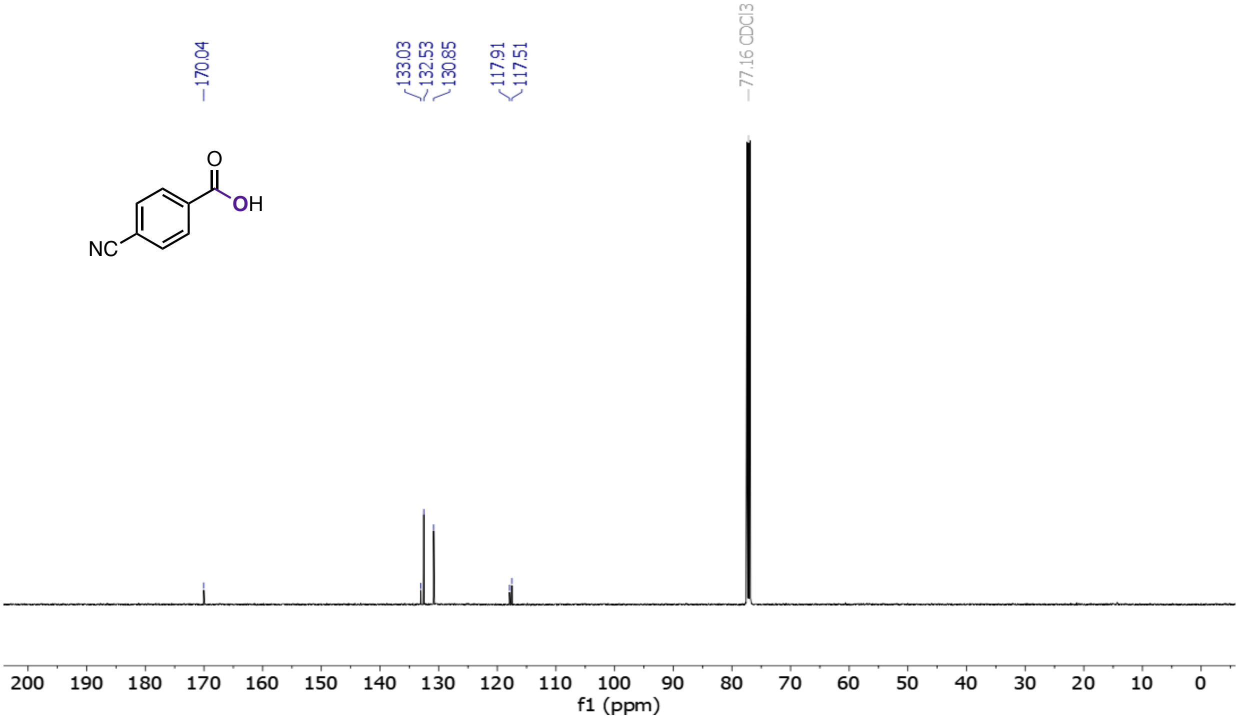

^{1}H NMR (400 MHz, $CDCl_3$) of Octanoic acid (10f)

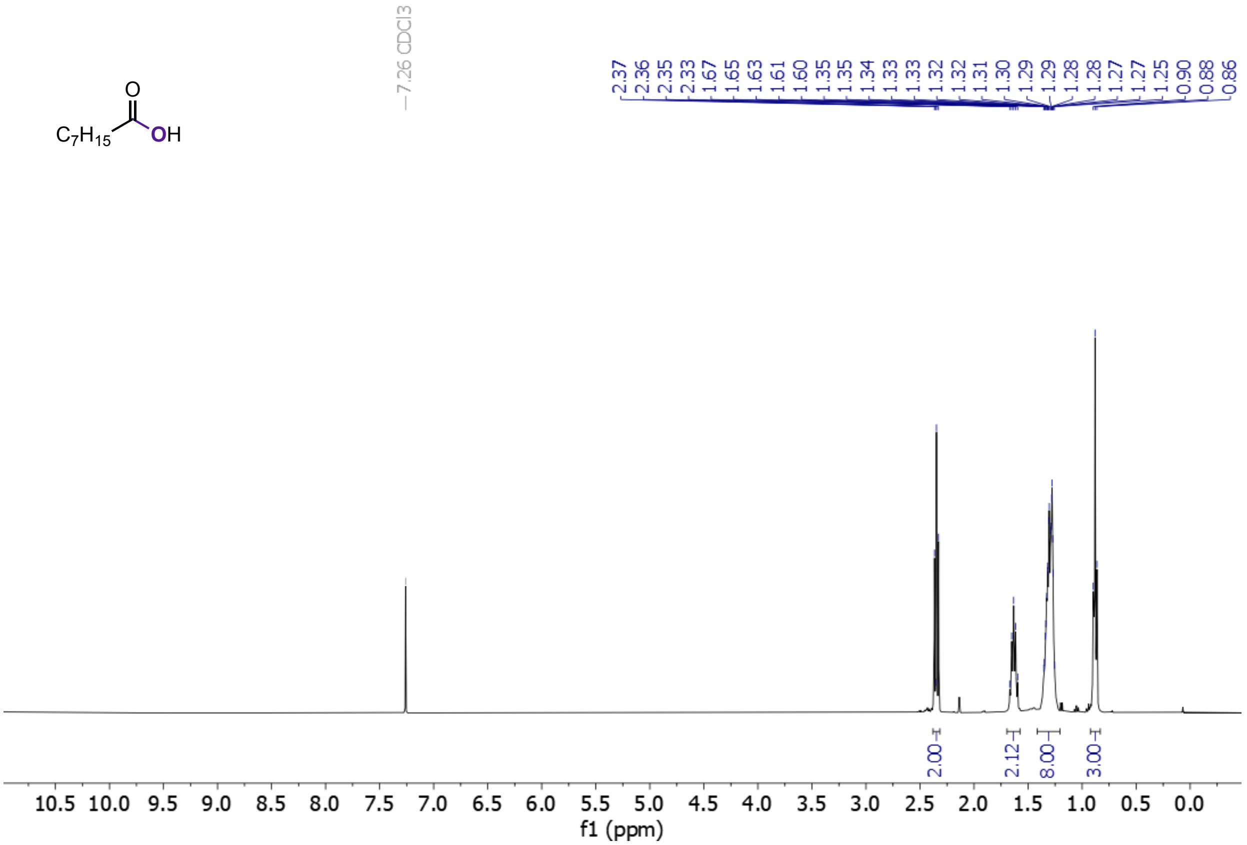

^{13}C NMR (126 MHz, $CDCl_3$) of Octanoic acid (10f)

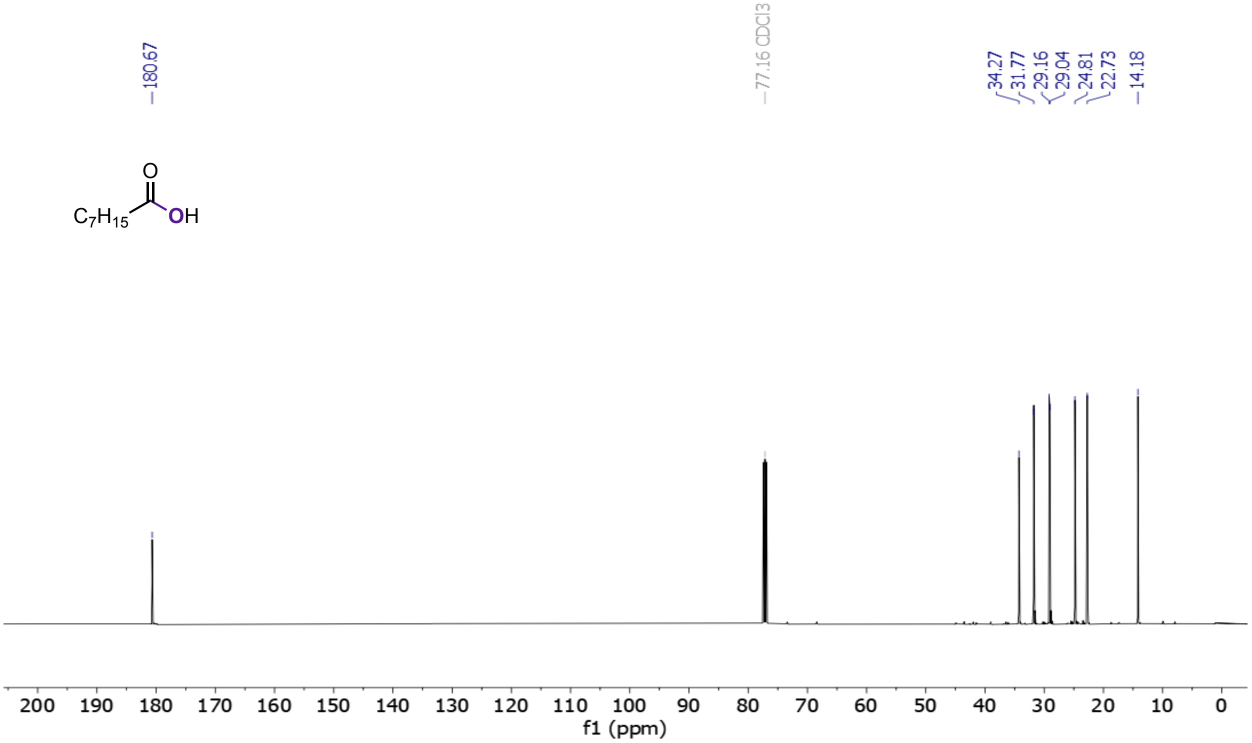

^{1}H NMR (500 MHz, $CDCl_3$) of Cyclohexanecarboxylic acid (10g)

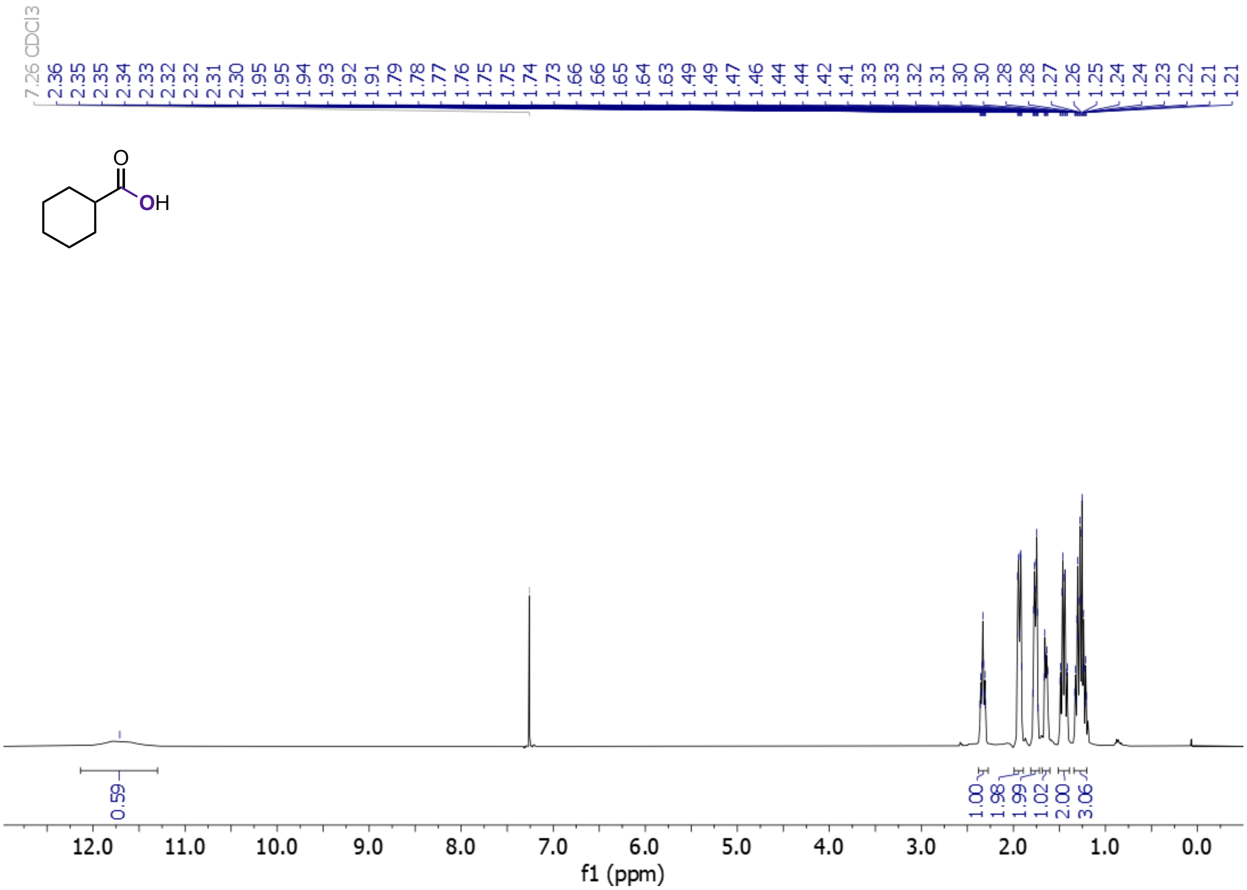

^{13}C NMR (126 MHz, $CDCl_3$) of Cyclohexanecarboxylic acid (10g)

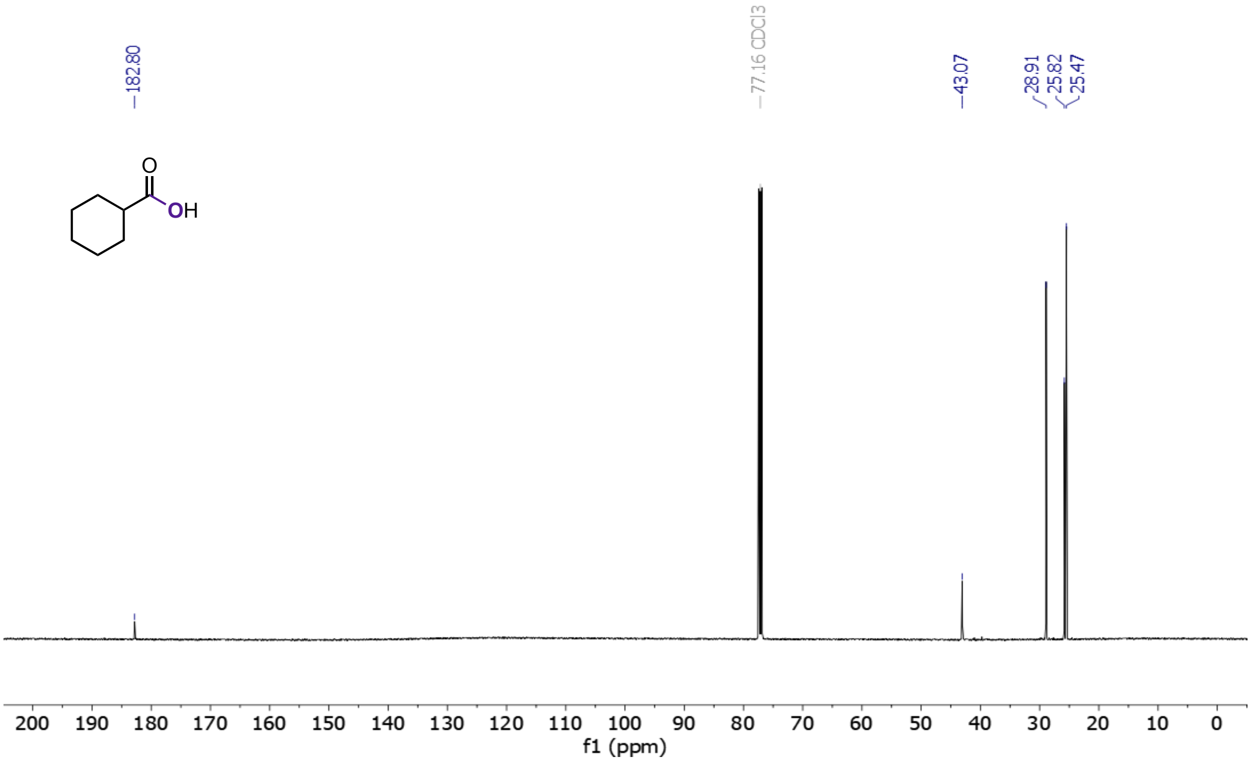

^{1}H NMR (400 MHz, DMSO-d_6) of 4-([1,1'-Biphenyl]-4-yl)-4-oxobutanoic acid (Fenbufen)(10h)

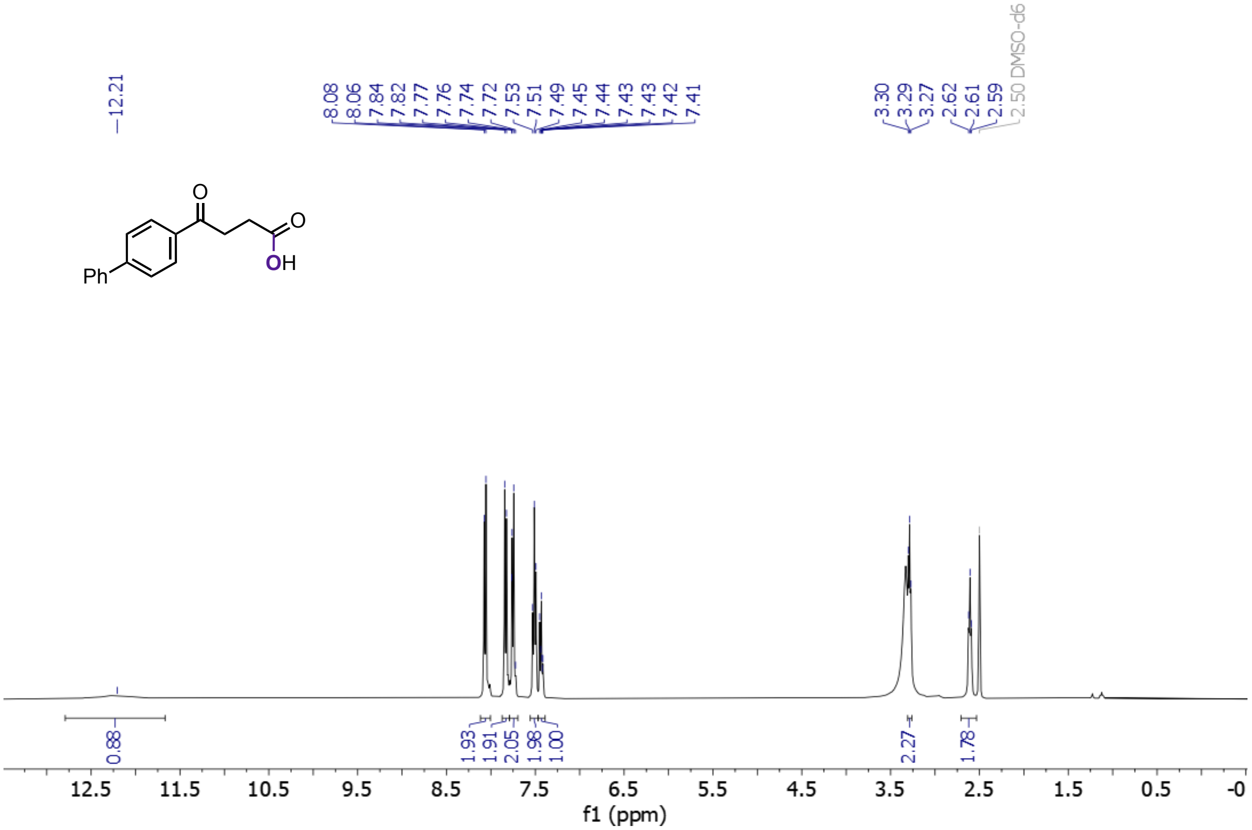

^{13}C NMR (101 MHz, DMSO-d_6) of 4-([1,1'-Biphenyl]-4-yl)-4-oxobutanoic acid (Fenbufen)(10h)

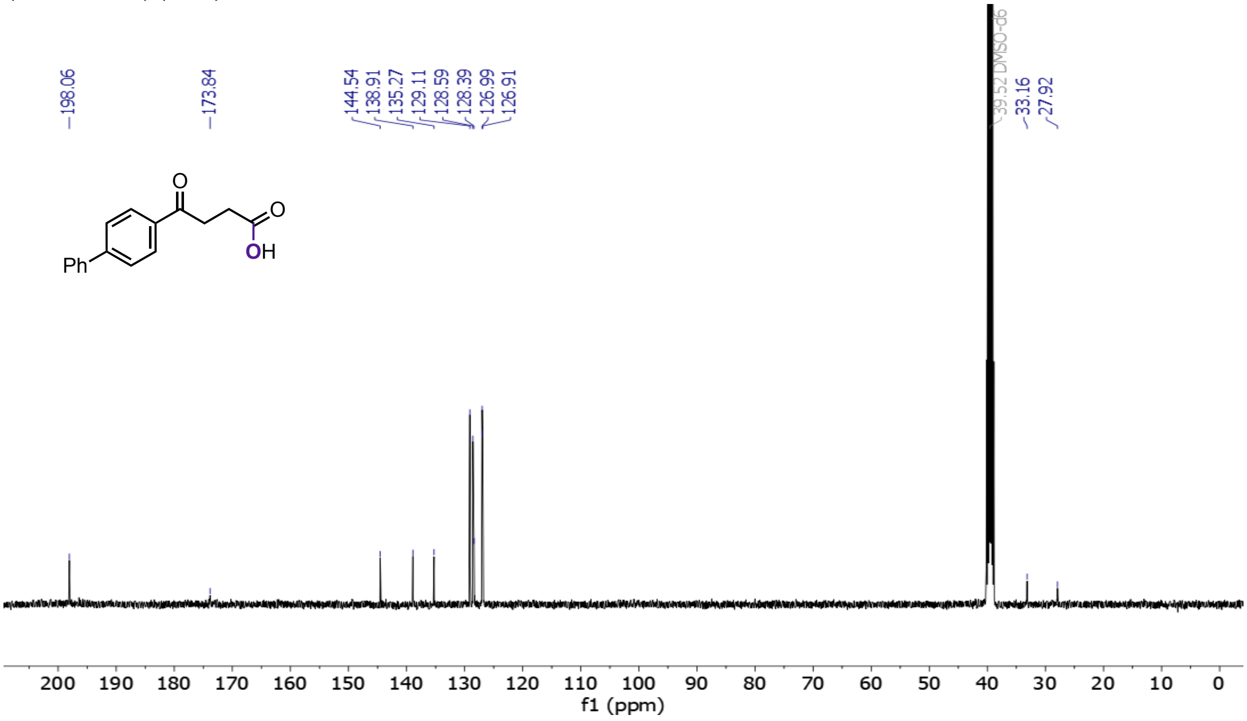

^{1}H NMR (400 MHz, $CDCl_3$) of *N*-Cyclohexylbenzamide (11a)

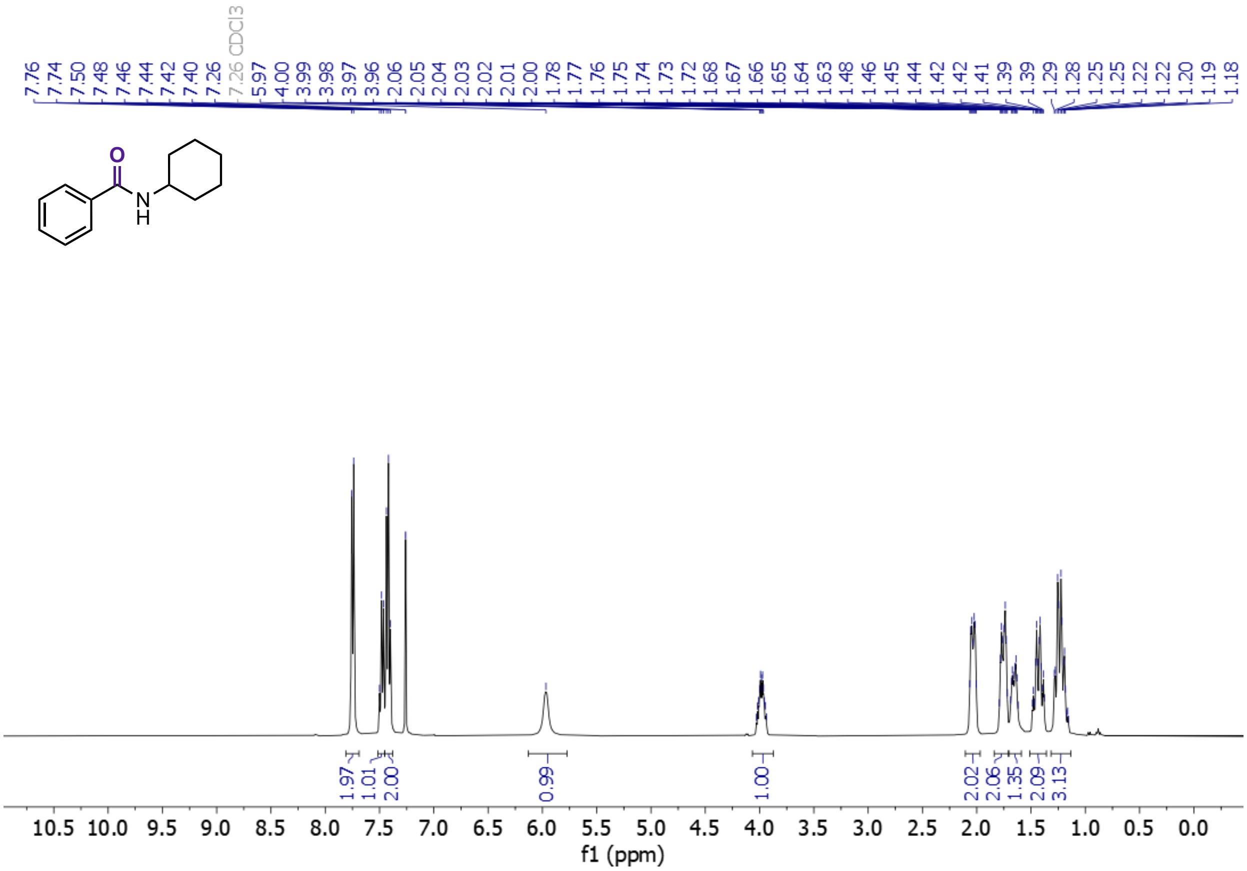

^{13}C NMR (101 MHz, $CDCl_3$) of *N*-Cyclohexylbenzamide (11a)

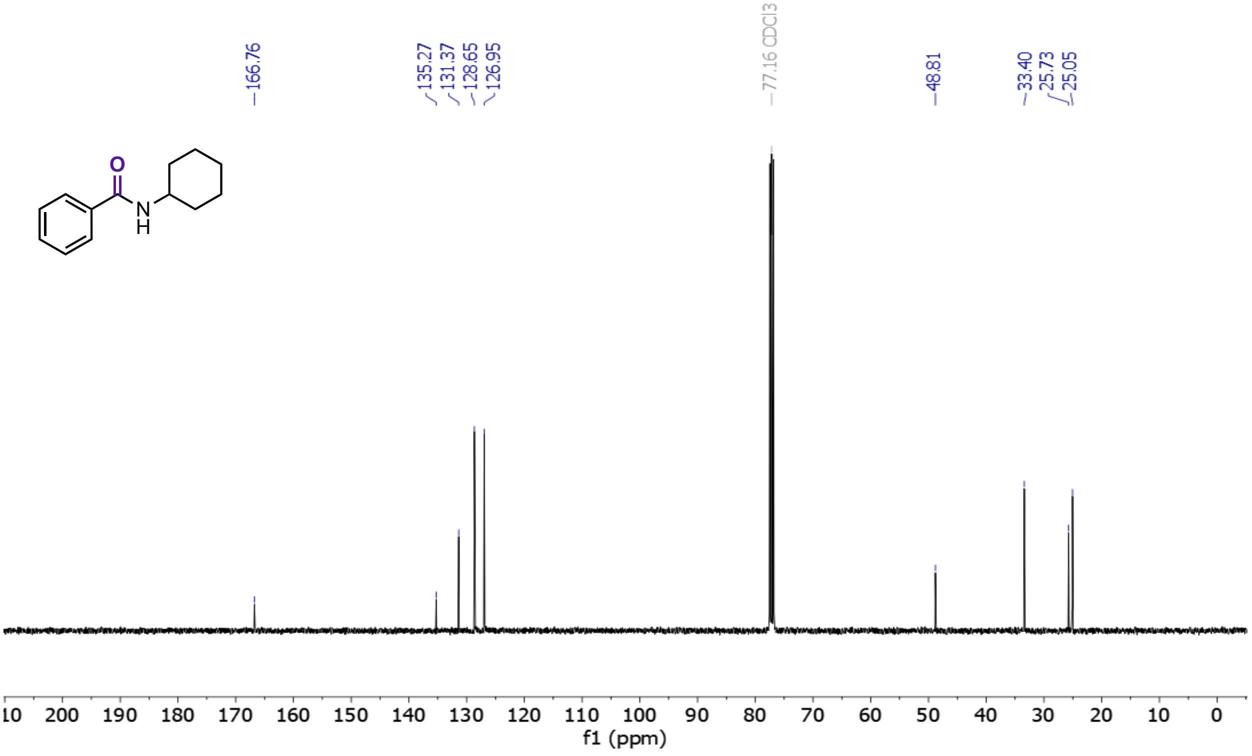

^{1}H NMR (400 MHz, $CDCl_3$) of *N*-(*tert*-Butyl)benzamide (11b)

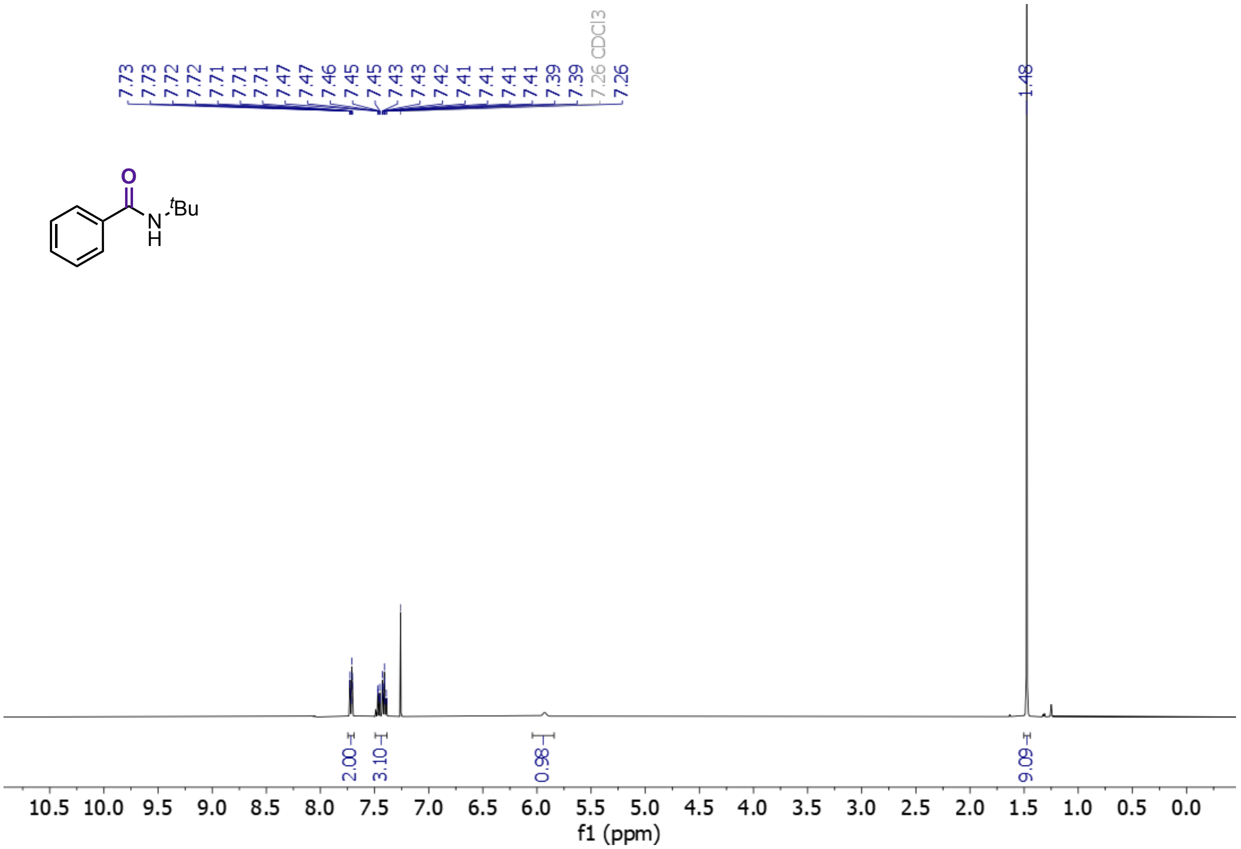

^{13}C NMR (101 MHz, $CDCl_3$) of *N*-(*tert*-Butyl)benzamide (11b)

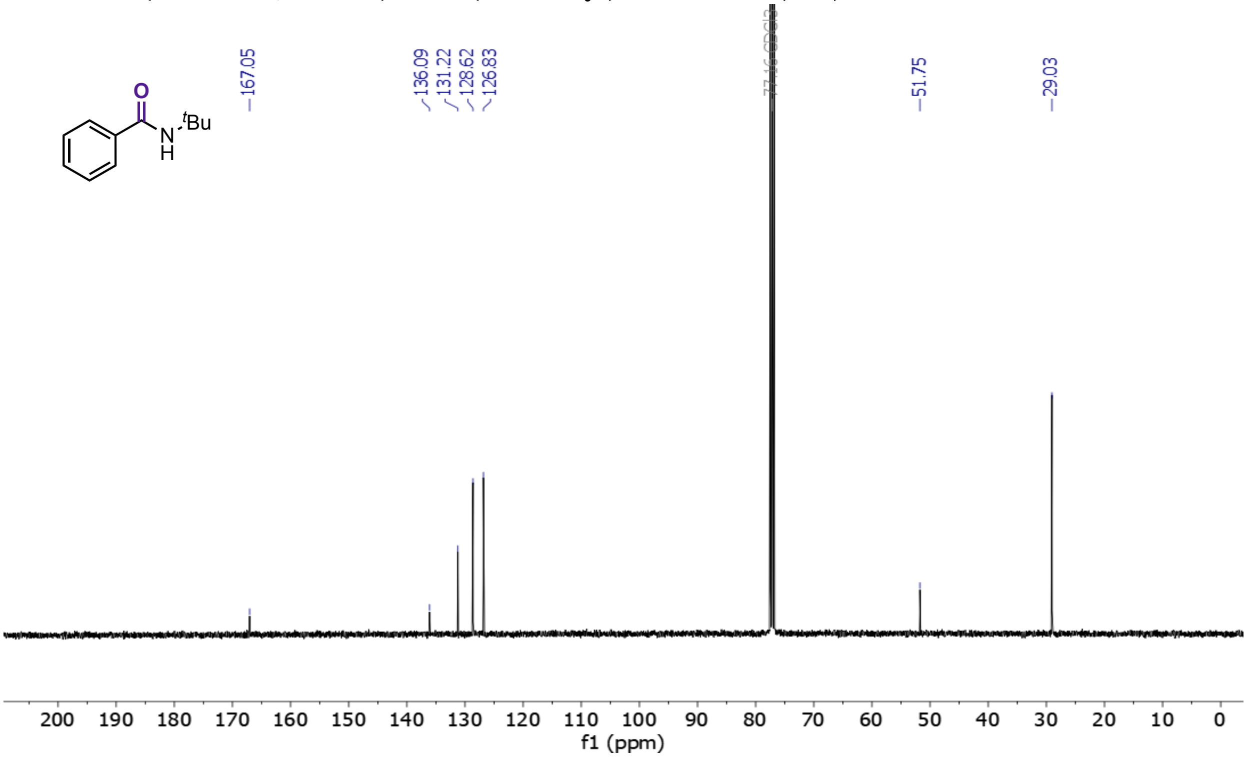

^{1}H NMR (500 MHz, $CDCl_3$) of *N*-(4-Fluorophenyl)benzamide (11c)

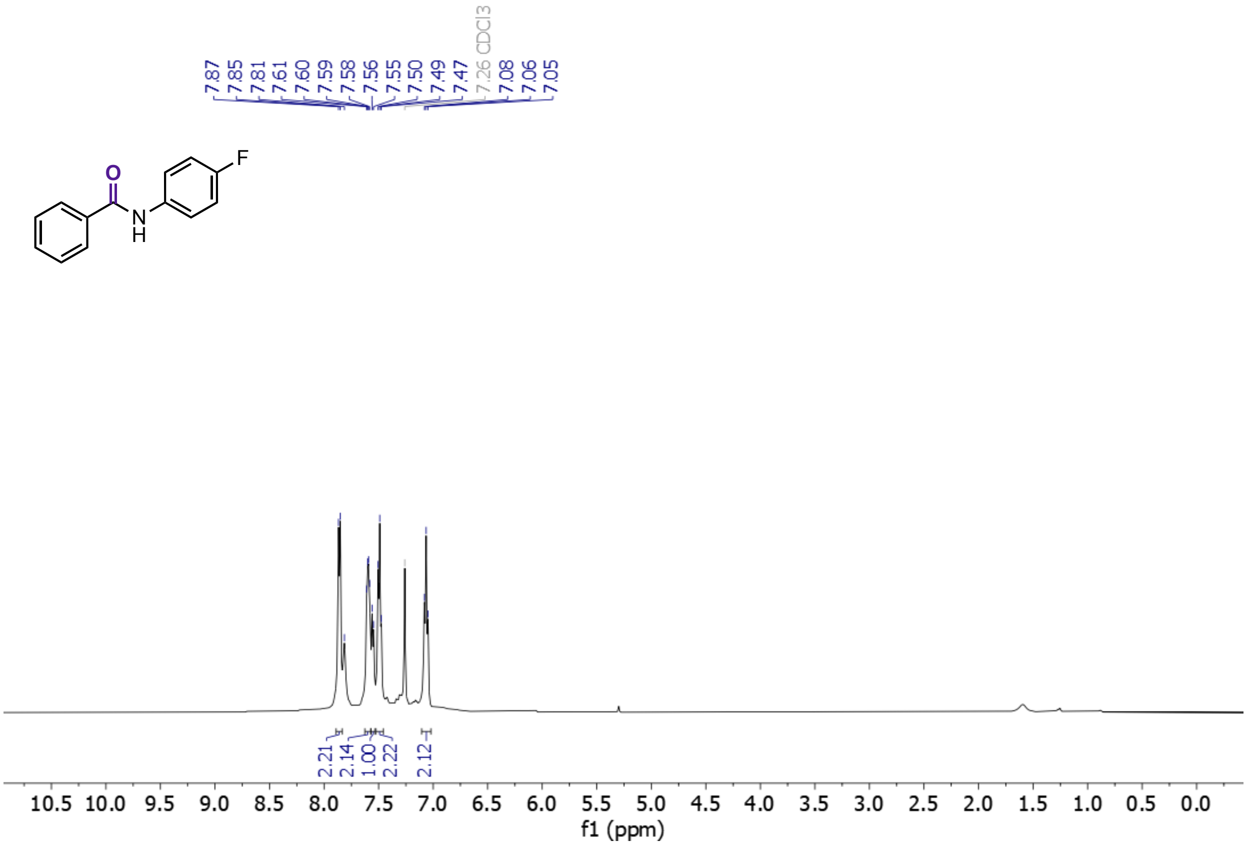

^{13}C NMR (126 MHz, $CDCl_3$) of *N*-(4-Fluorophenyl)benzamide (11c)

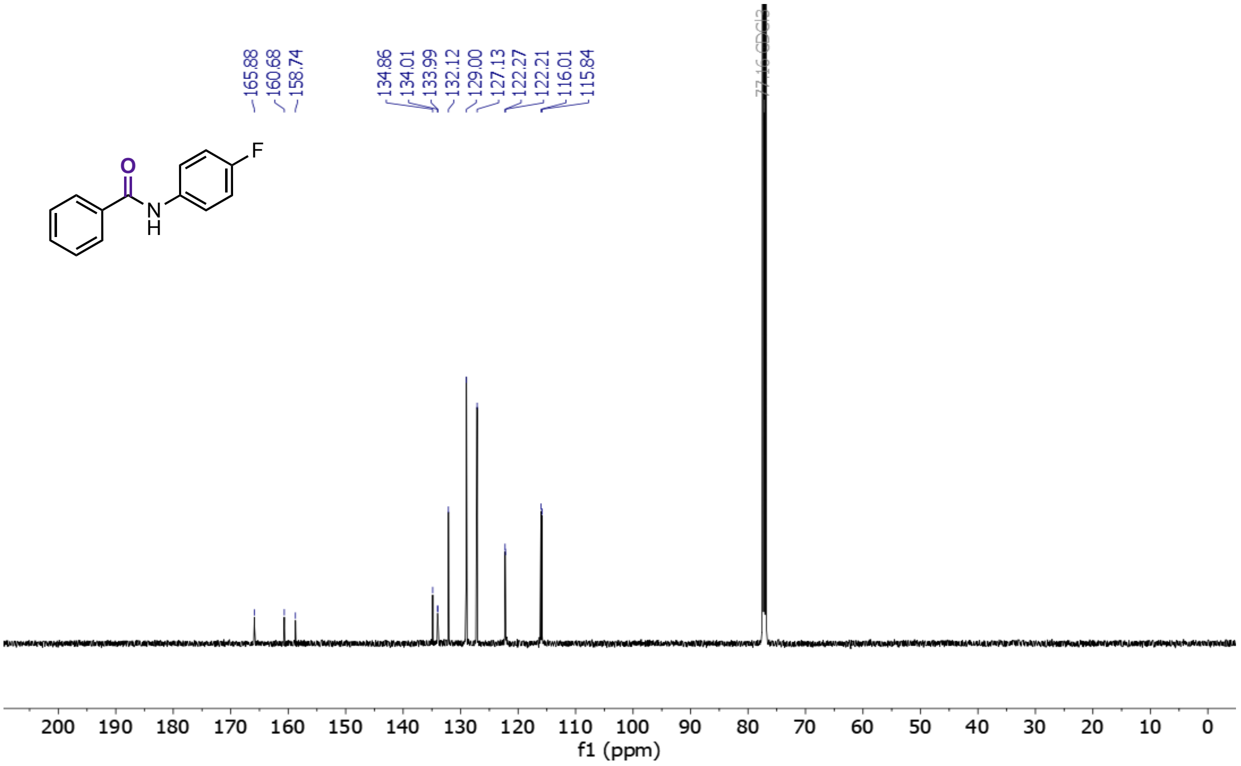

^{1}H NMR (400 MHz, $CDCl_3$) of *N*-Phenylcyclohexanamide (11d)

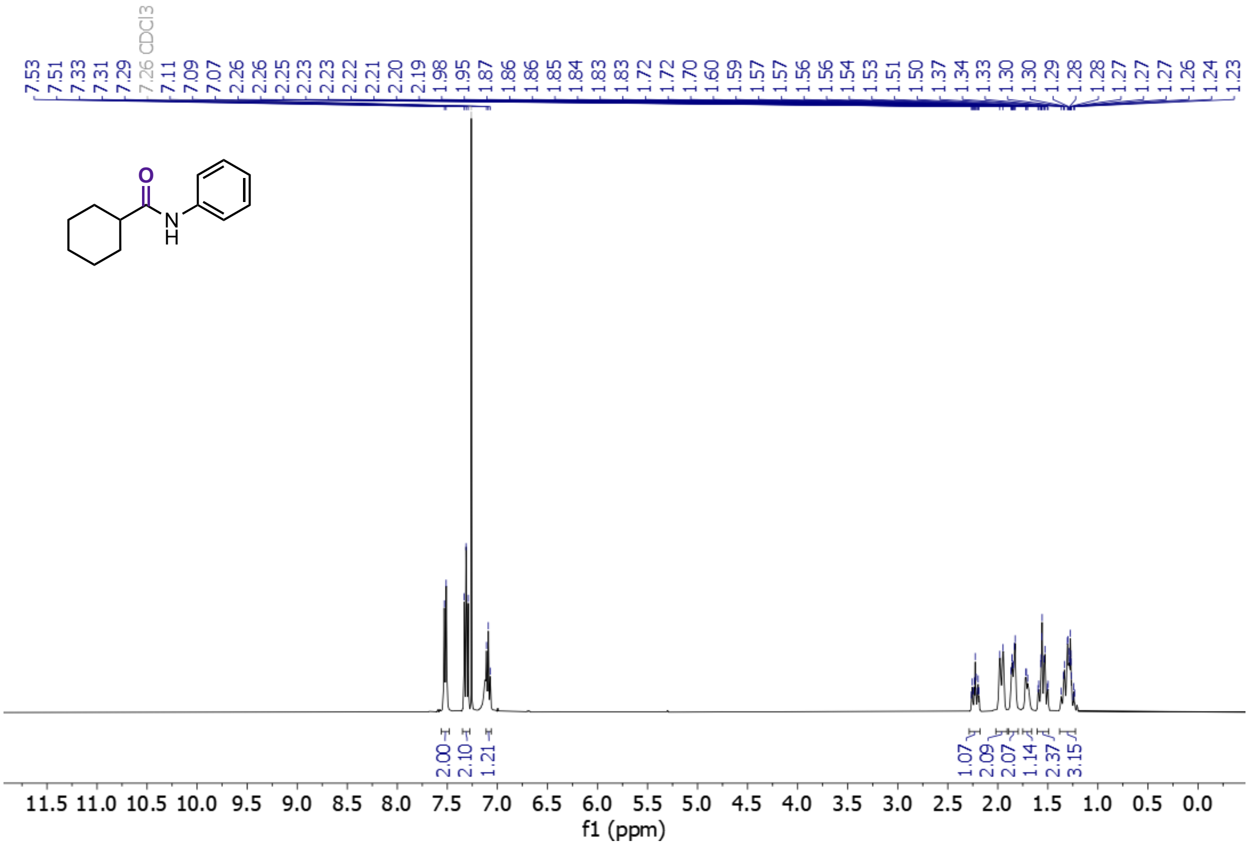

^{13}C NMR (101 MHz, $CDCl_3$) of *N*-Phenylcyclohexanamide (11d)

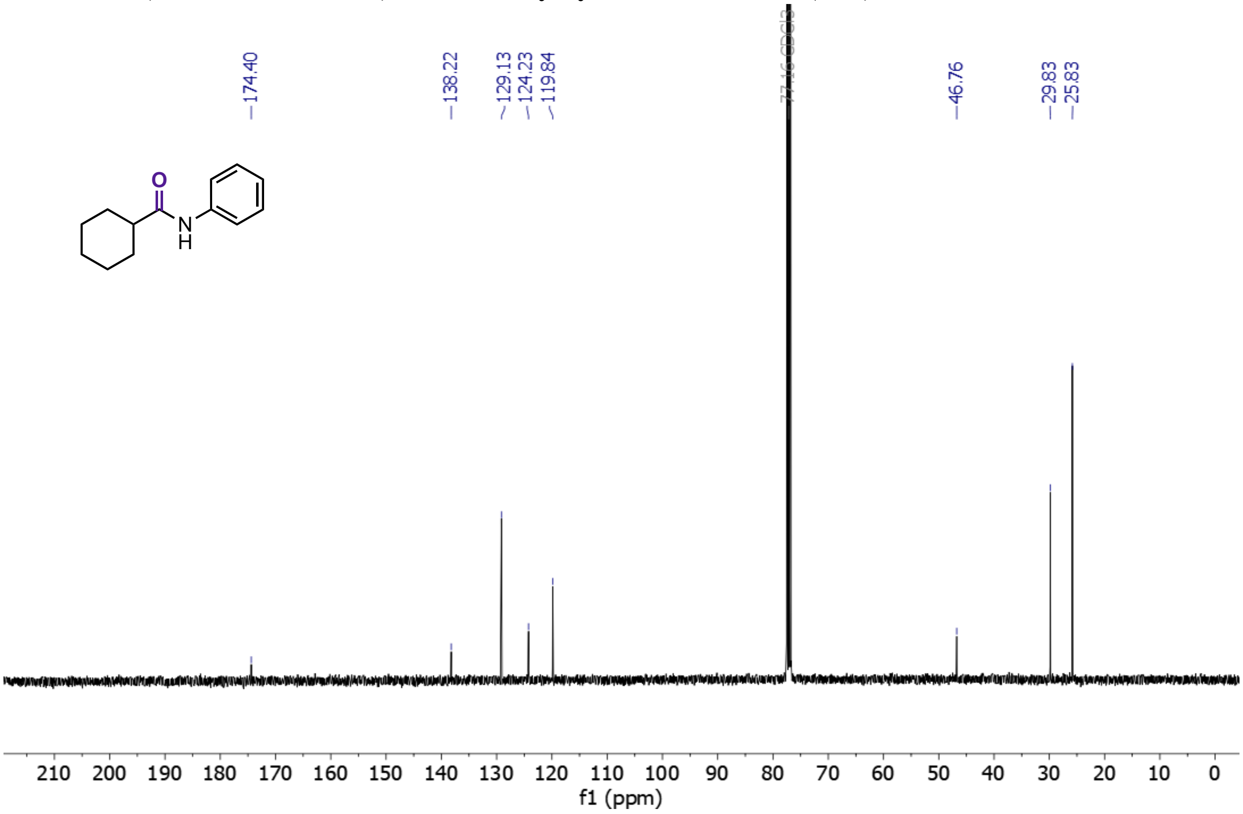

^{1}H NMR (500 MHz, $CDCl_3$) of *N*-Hexylcyclohexanecarboxamide (11e)

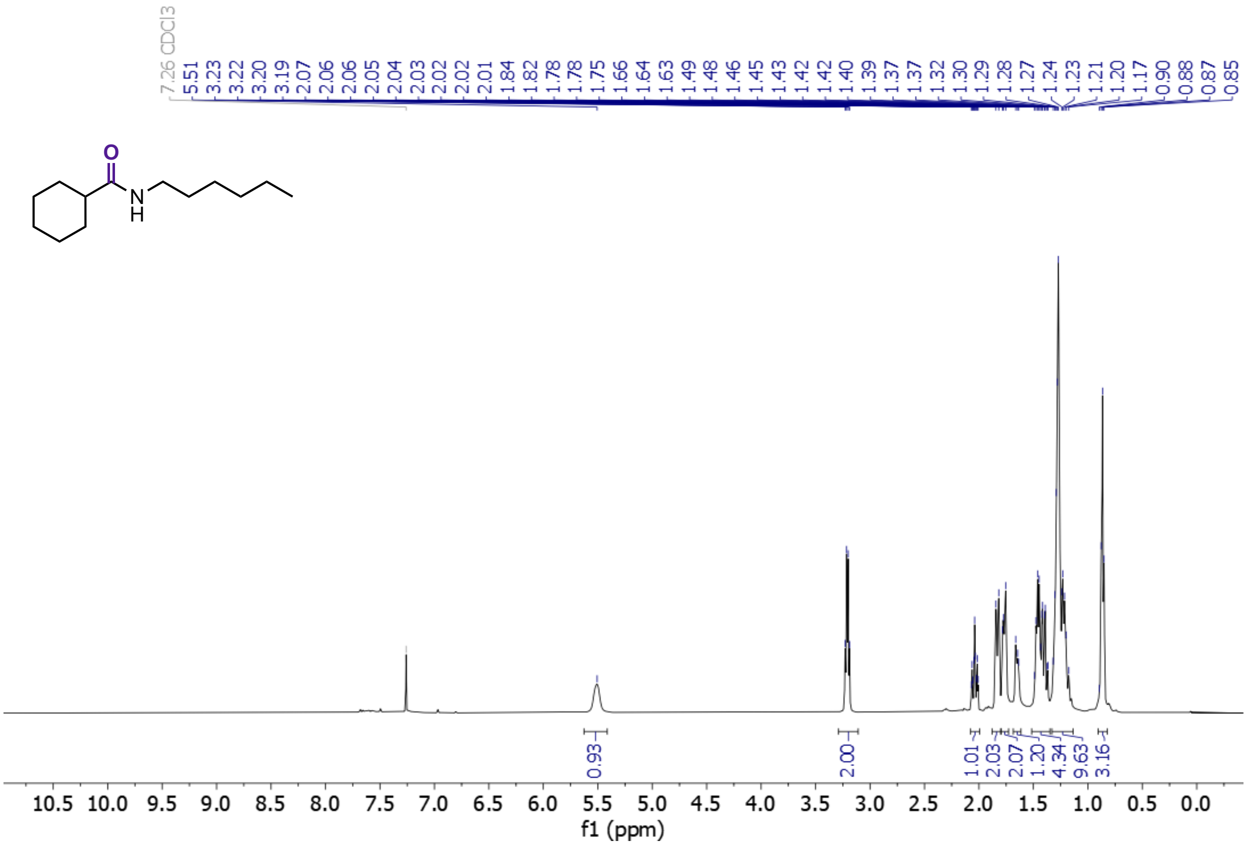

^{13}C NMR (126 MHz, $CDCl_3$) of *N*-Hexylcyclohexanecarboxamide (11e)

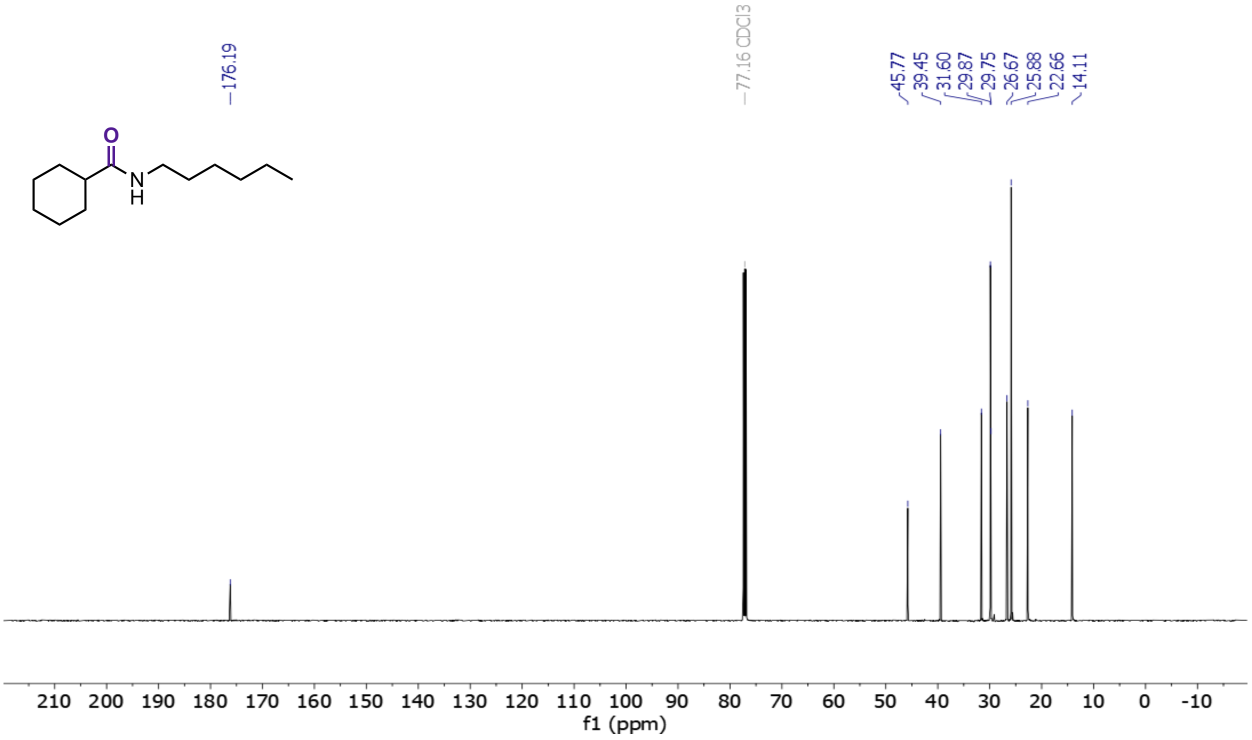

^{1}H NMR (500 MHz, $CDCl_3$) of 2-phenylbutane-2,3-diol

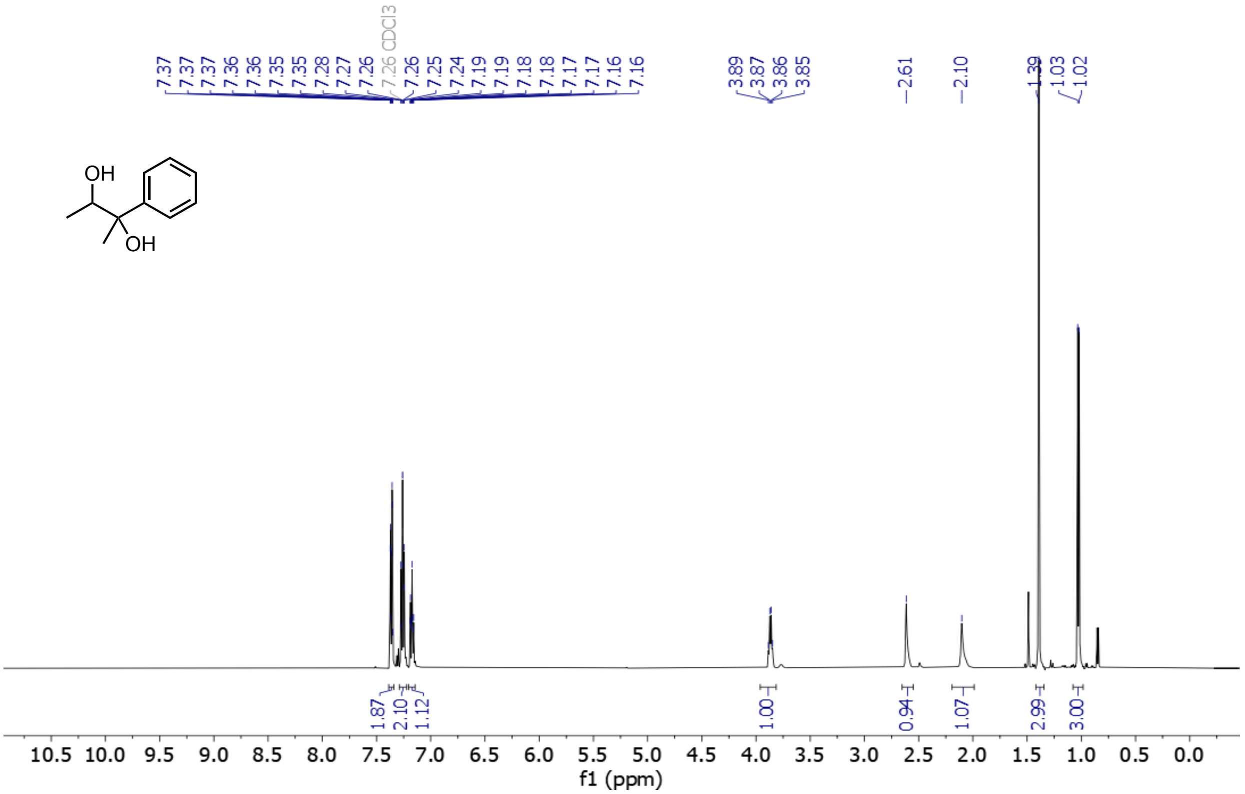

^{13}C NMR (126 MHz, $CDCl_3$) of 2-phenylbutane-2,3-diol

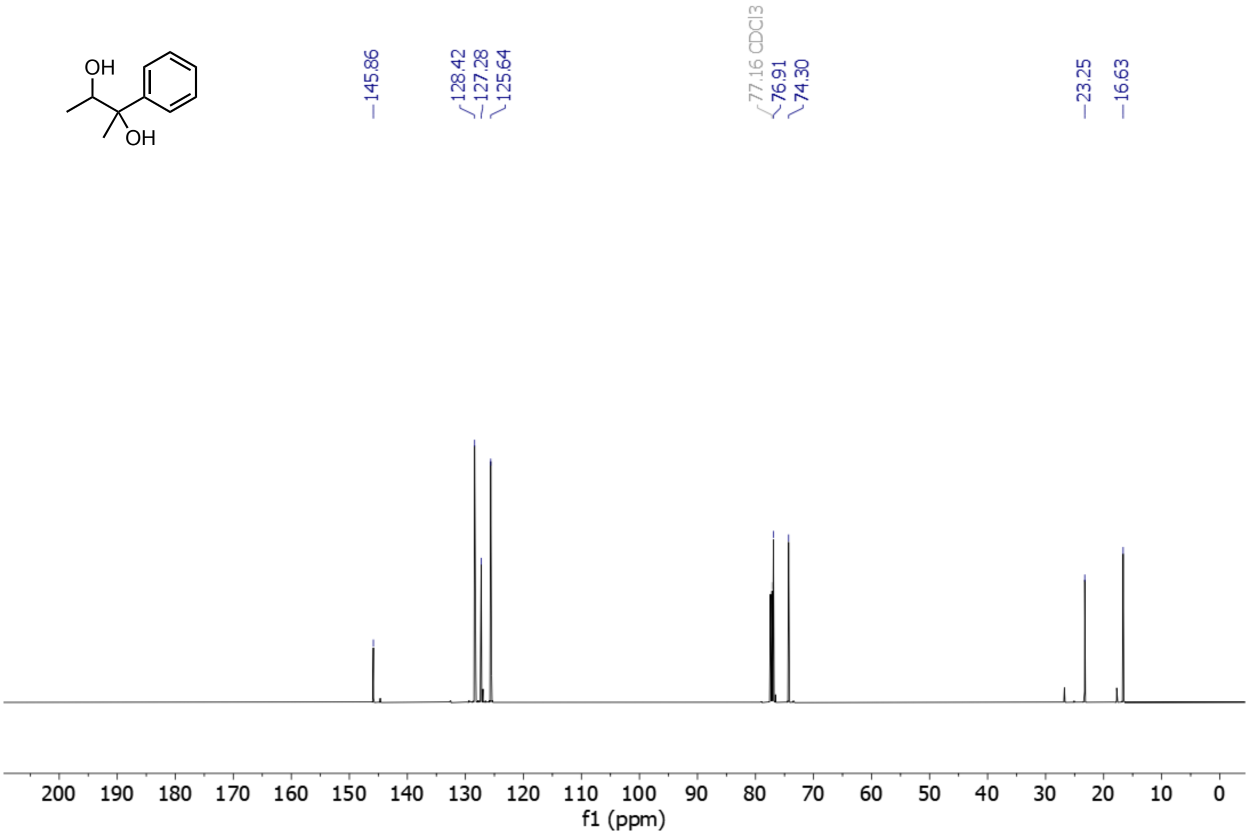

^{1}H NMR (500 MHz, $CDCl_3$) of 3-Hydroxy-3-phenylbutan-2-one

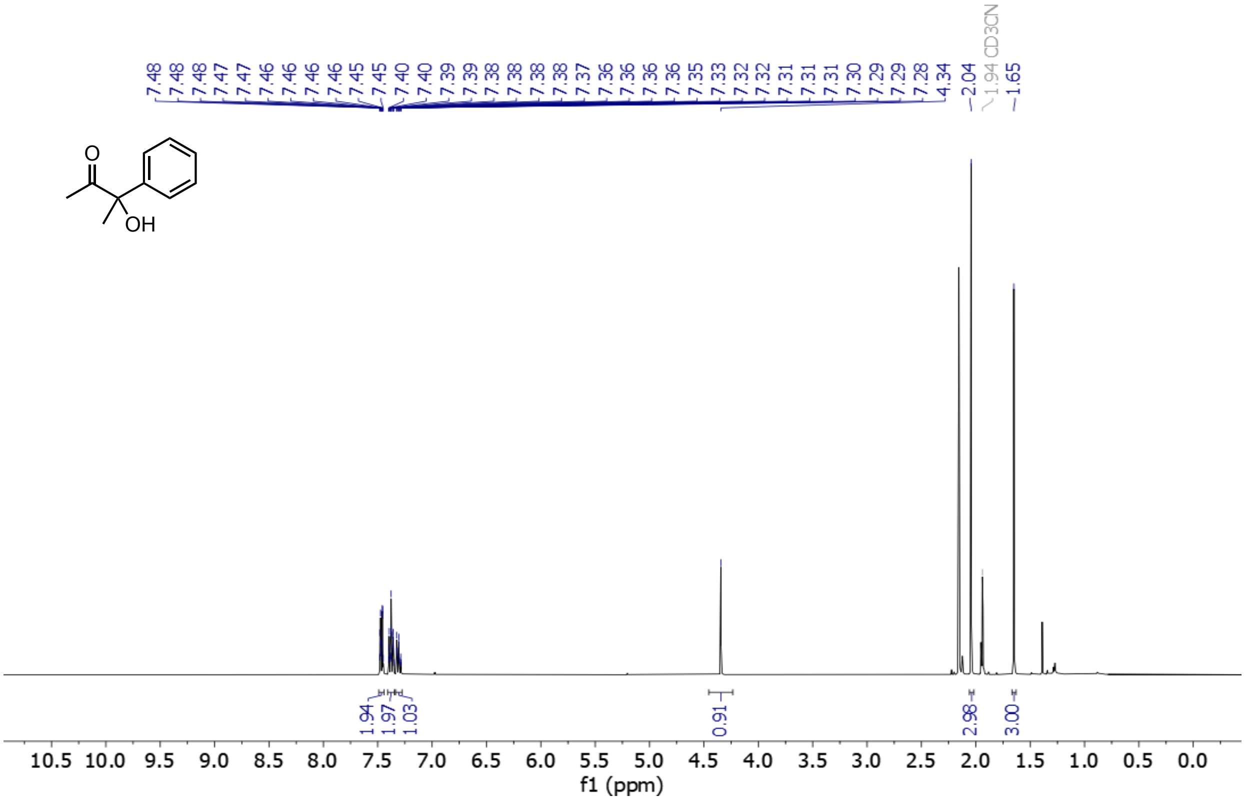

^{13}C NMR (126 MHz, $CDCl_3$) of 3-Hydroxy-3-phenylbutan-2-one

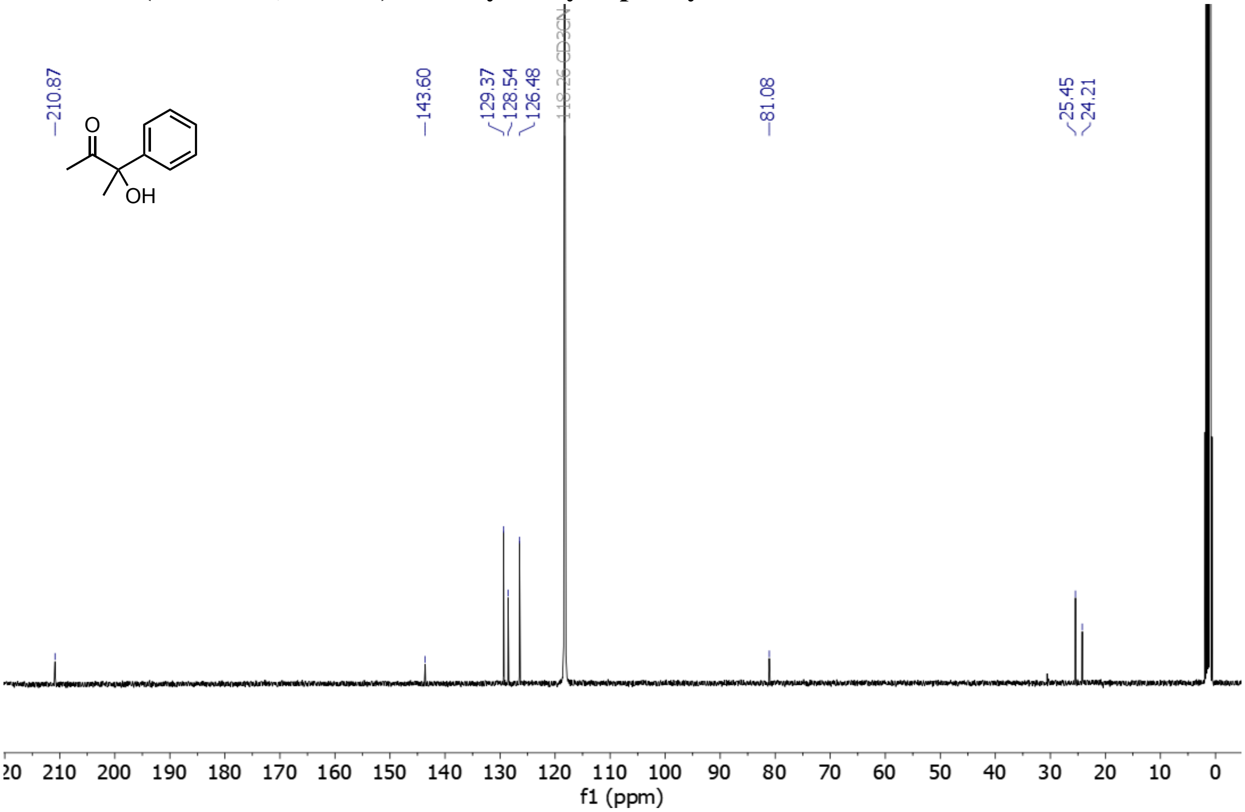

NMR yield spectra

^{1}H NMR (500 MHz, CD_3CN) of Cyclobutanone (3ac)

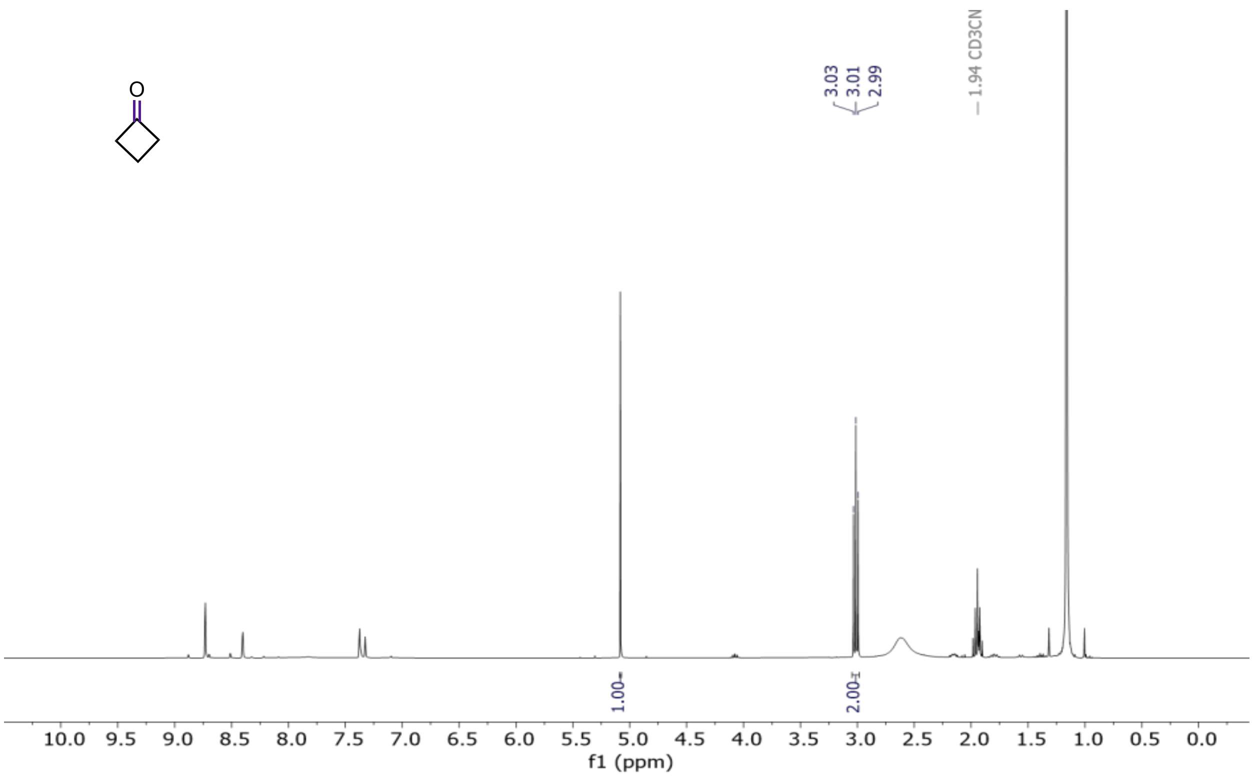

^{1}H NMR (500 MHz, CD_3CN) of Cyclopentanone (3ad)

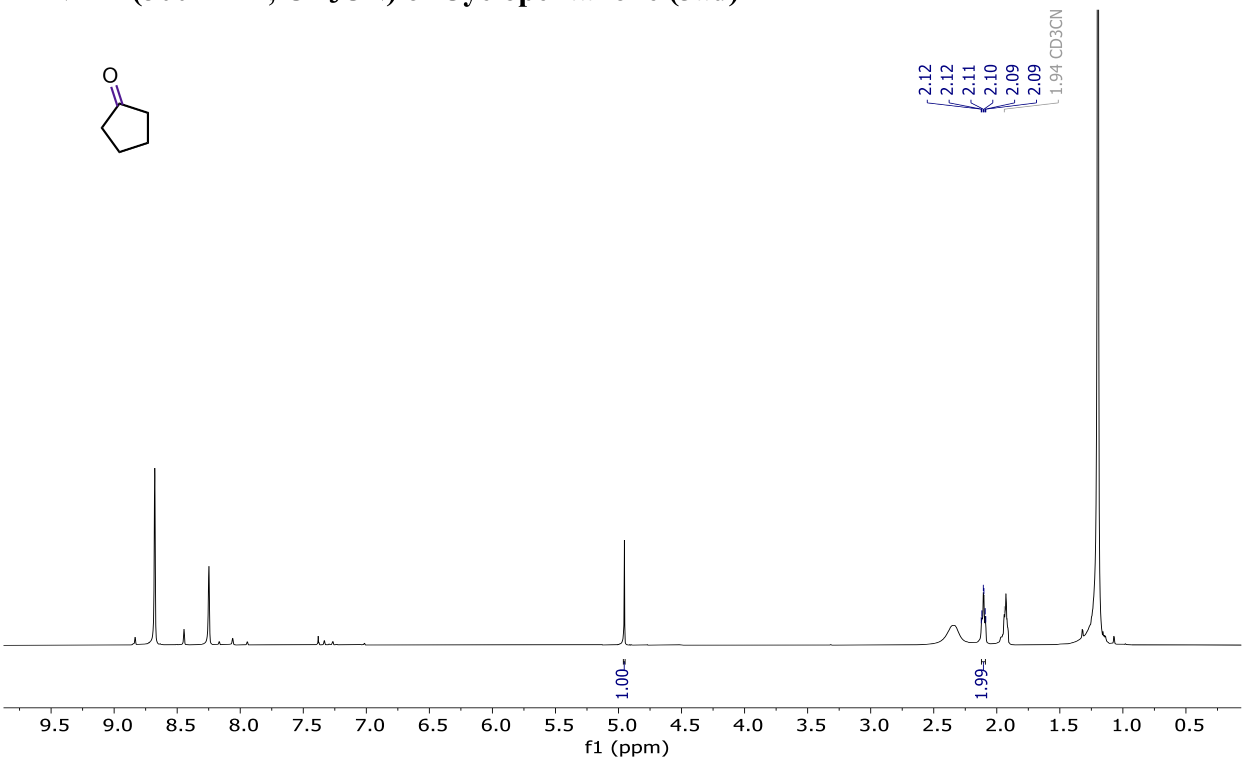

^1H NMR (500 MHz, CD_3CN) of Cyclohexanone (3ae)

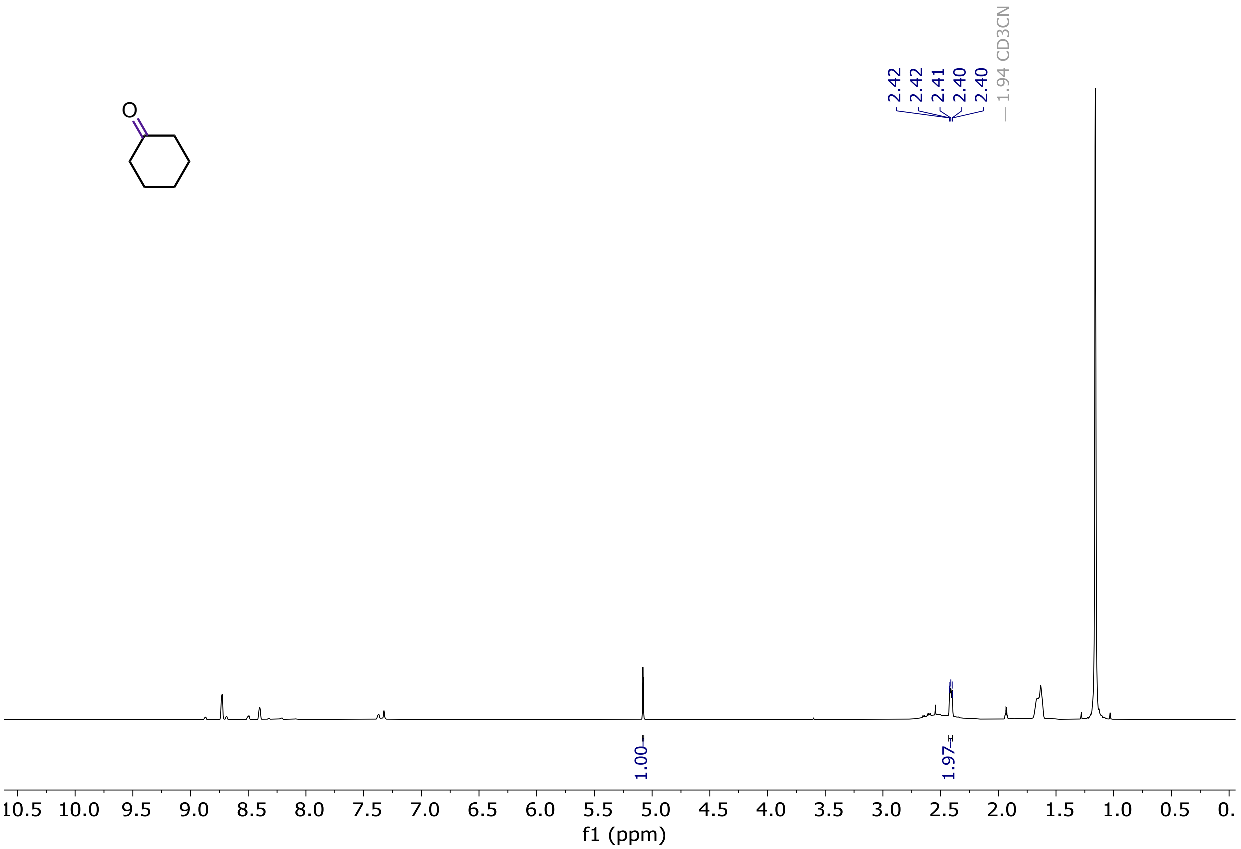

^1H NMR (500 MHz, CD_3CN) of Cycloheptanone (3af)

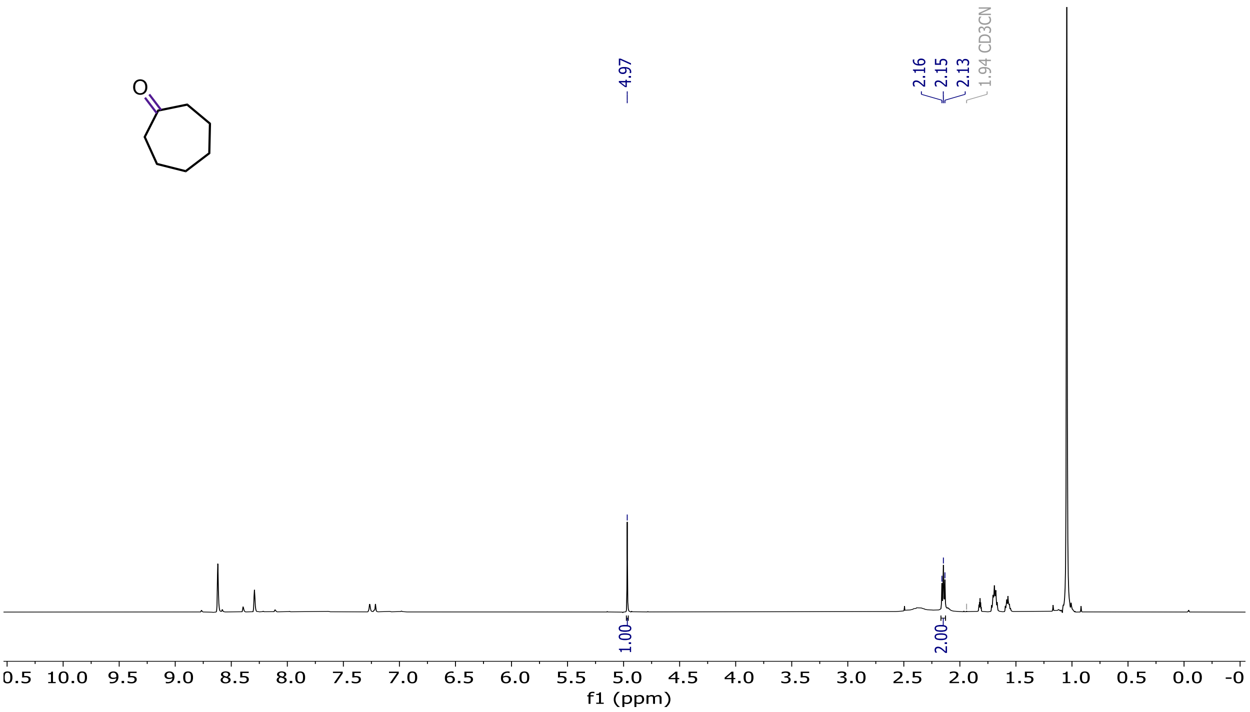

^{1}H NMR (500 MHz, $CDCl_3$) of (*E*)-*N*-*tert*-butyl-1-phenylmethanimine (4c)

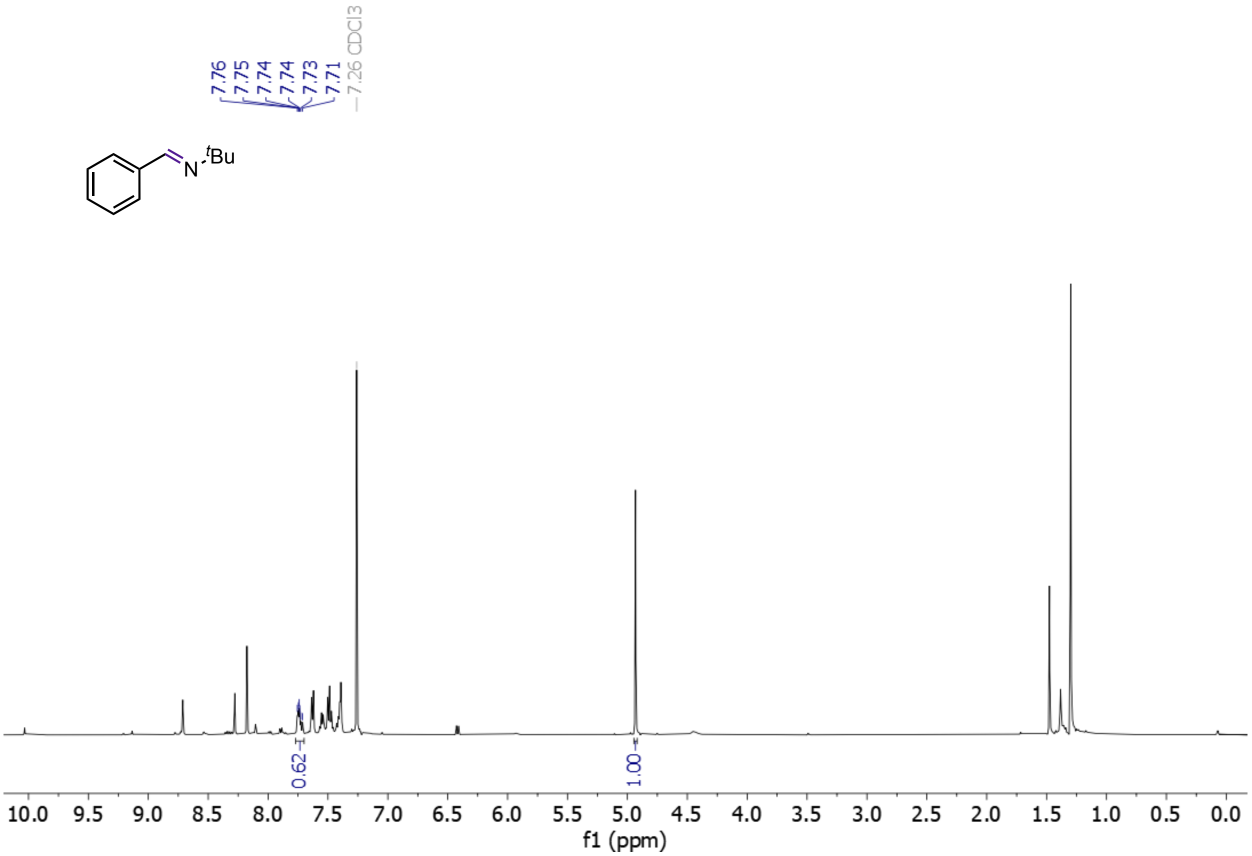

^{1}H NMR (500 MHz, $CDCl_3$) of (*E*)-*N*-(4-methoxybenzyl)-1-(4-methoxyphenyl)methanimine (4d)

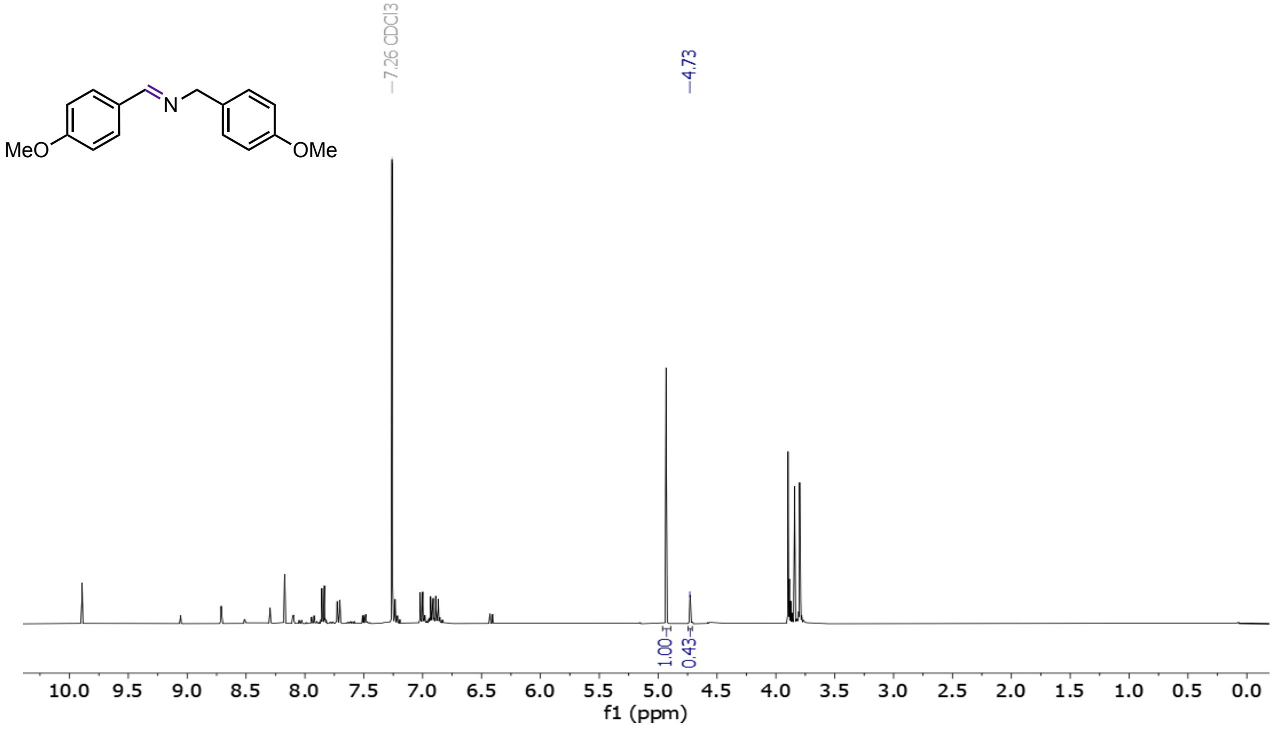

^{1}H NMR (500 MHz, CDCl₃) of 3,4-dihydroisoquinoline (4e)

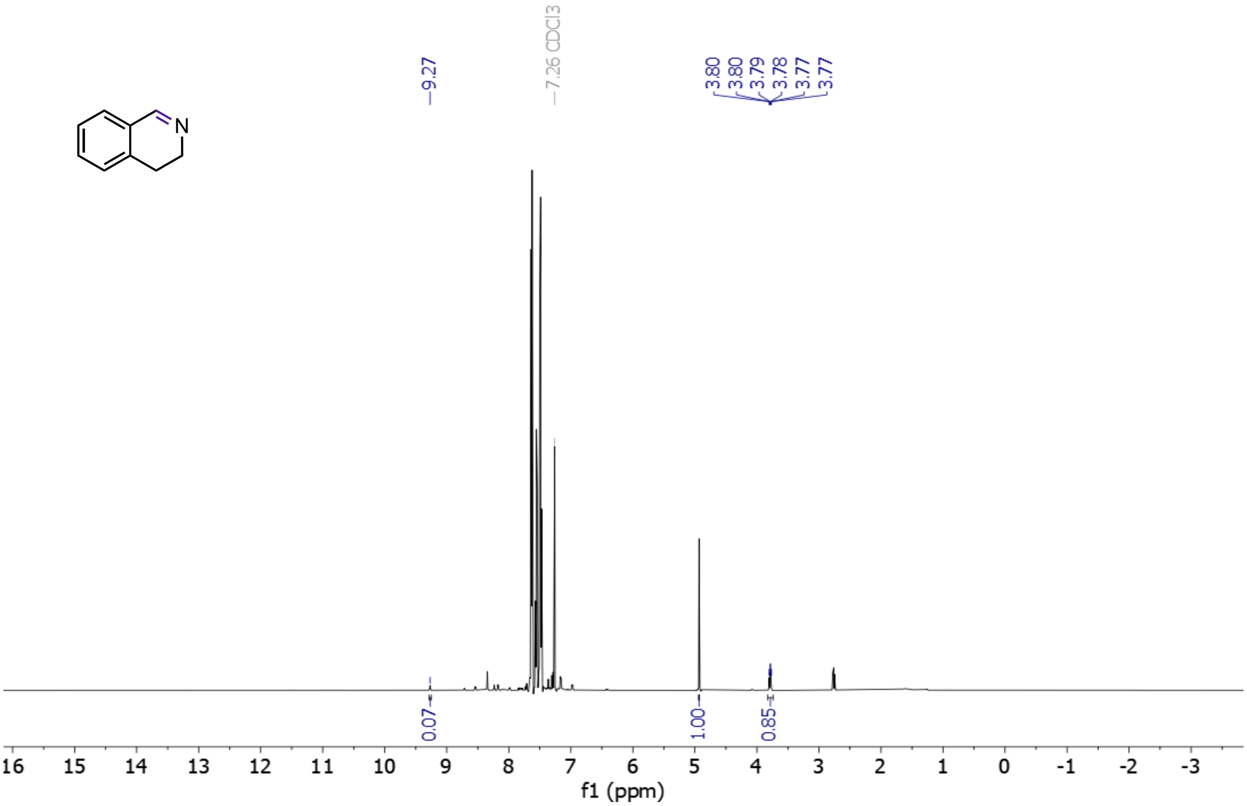

^{1}H NMR (500 MHz, CD₃CN) of Diphenylmethanimine (4g)

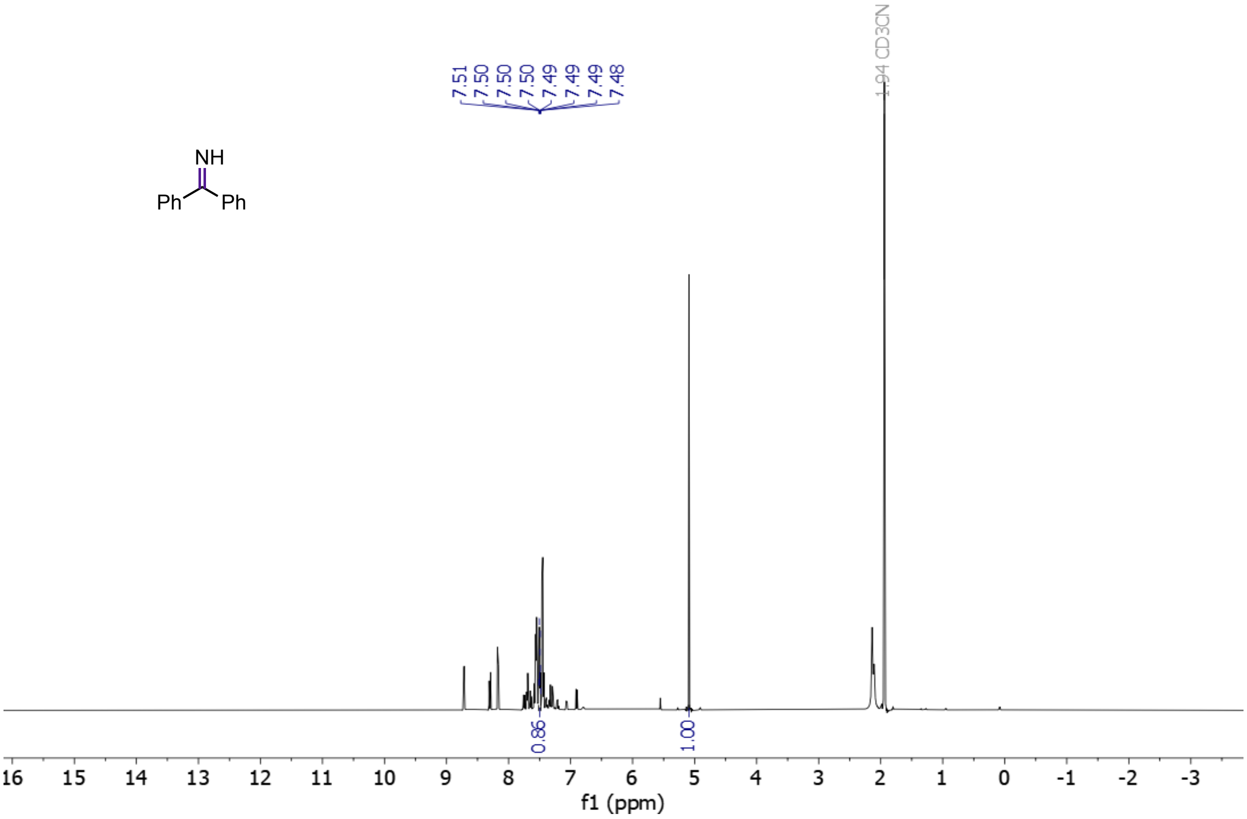

^{1}H NMR (500 MHz, $CDCl_3$) of (*E*)-*N*-Octyloctan-1-imine (4h)

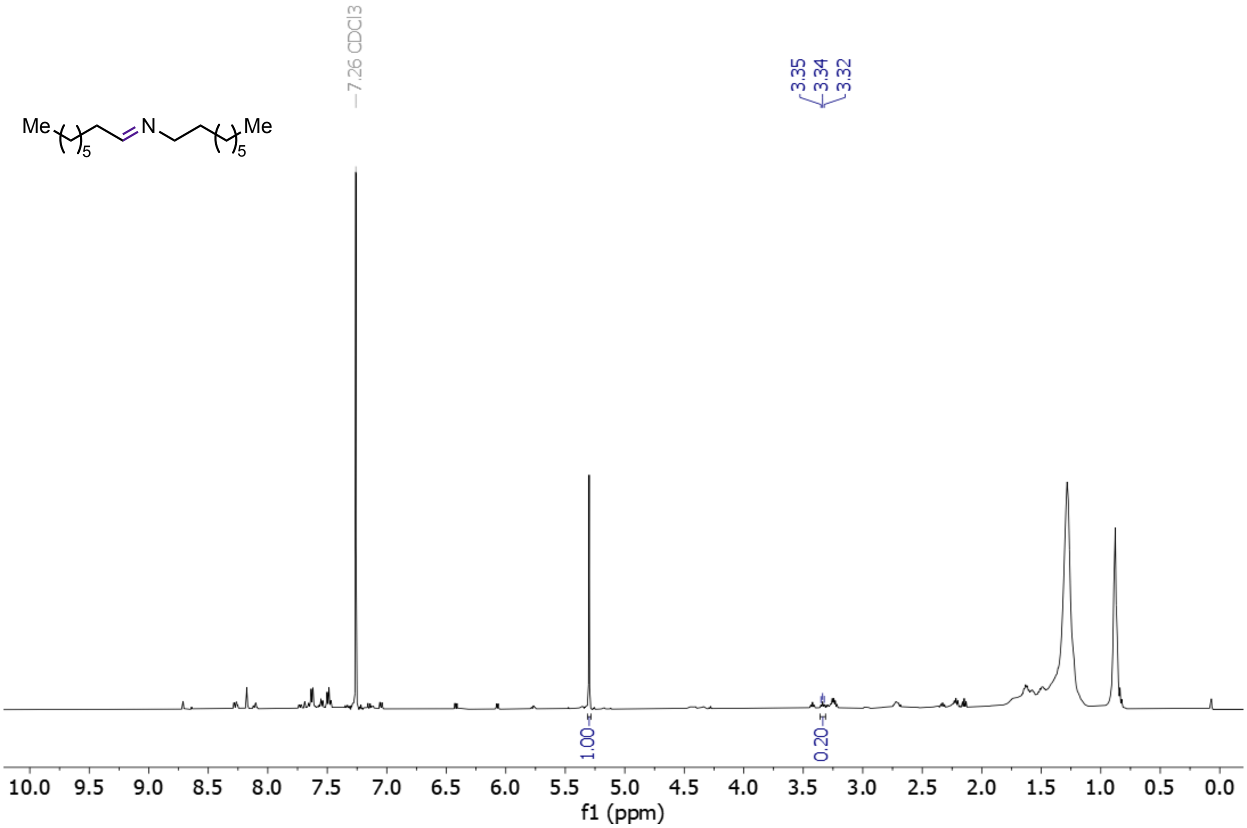

^{1}H NMR (500 MHz, $CDCl_3$) of (*E*)-*N*-(*tert*-butyl)octan-1-imine (4i)

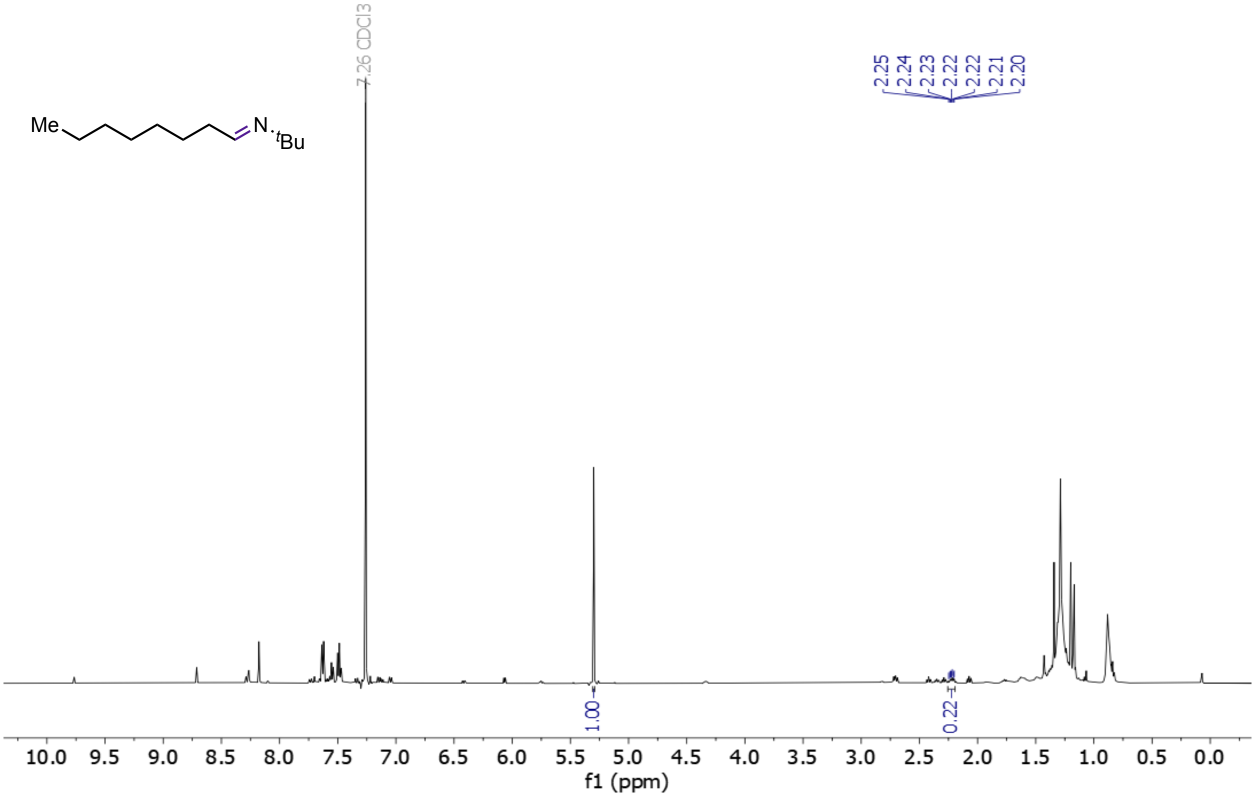

Continuous-Flow NMR yield spectra

^{1}H NMR (400 MHz, $CDCl_3$) of (*E*)-*N*-benzyl-1-phenylmethanimine (4a)

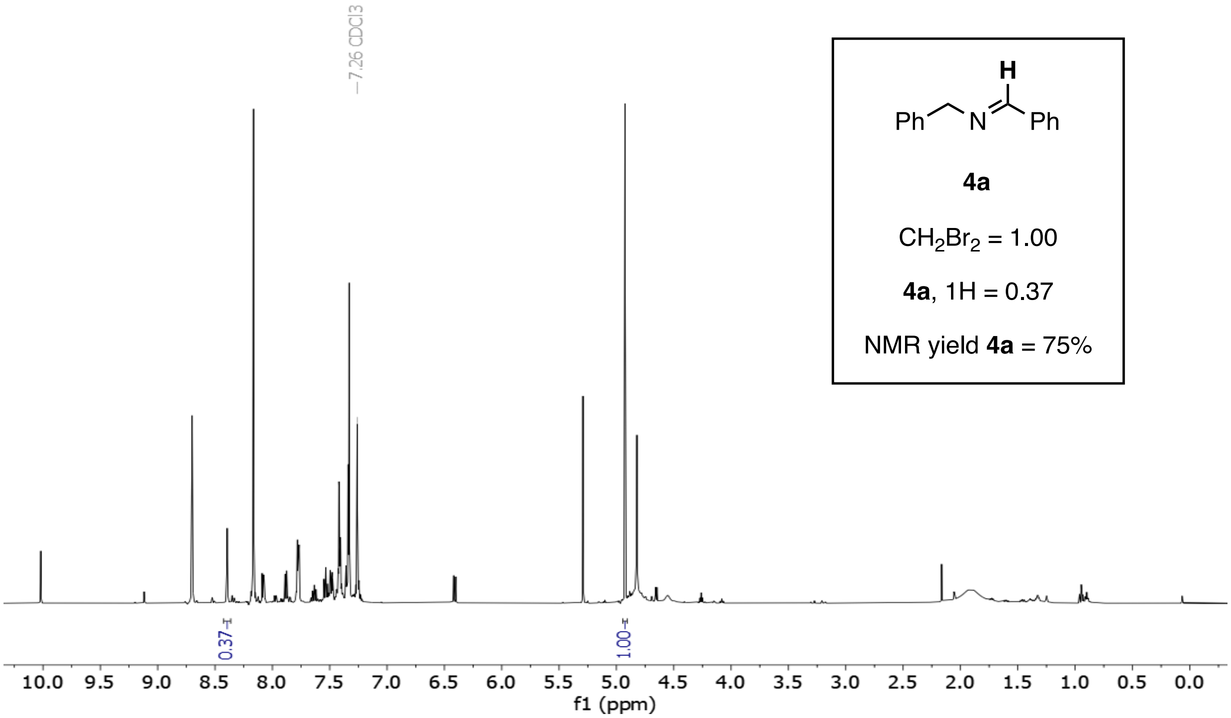

^{1}H NMR (400 MHz, $CDCl_3$) of 2-chloro-1-phenylethan-1-one (3q)

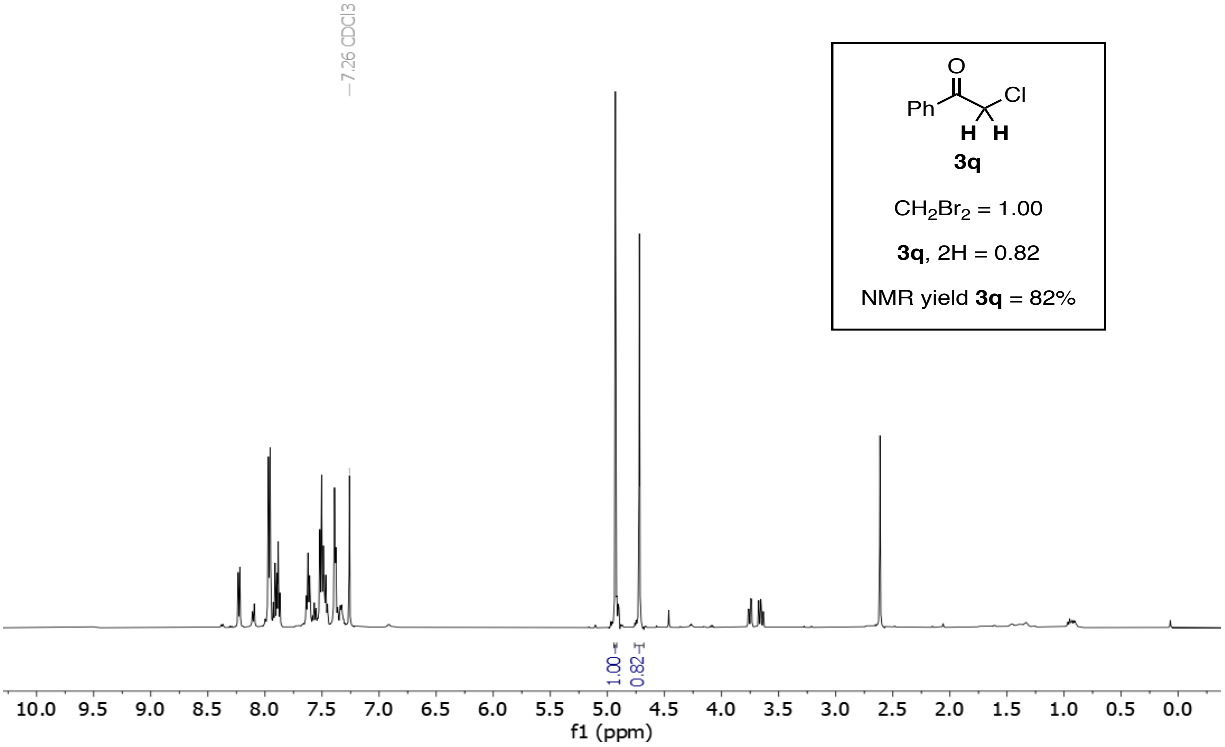

^{1}H NMR (400 MHz, $CDCl_3$) of 5-nonanone (3z)

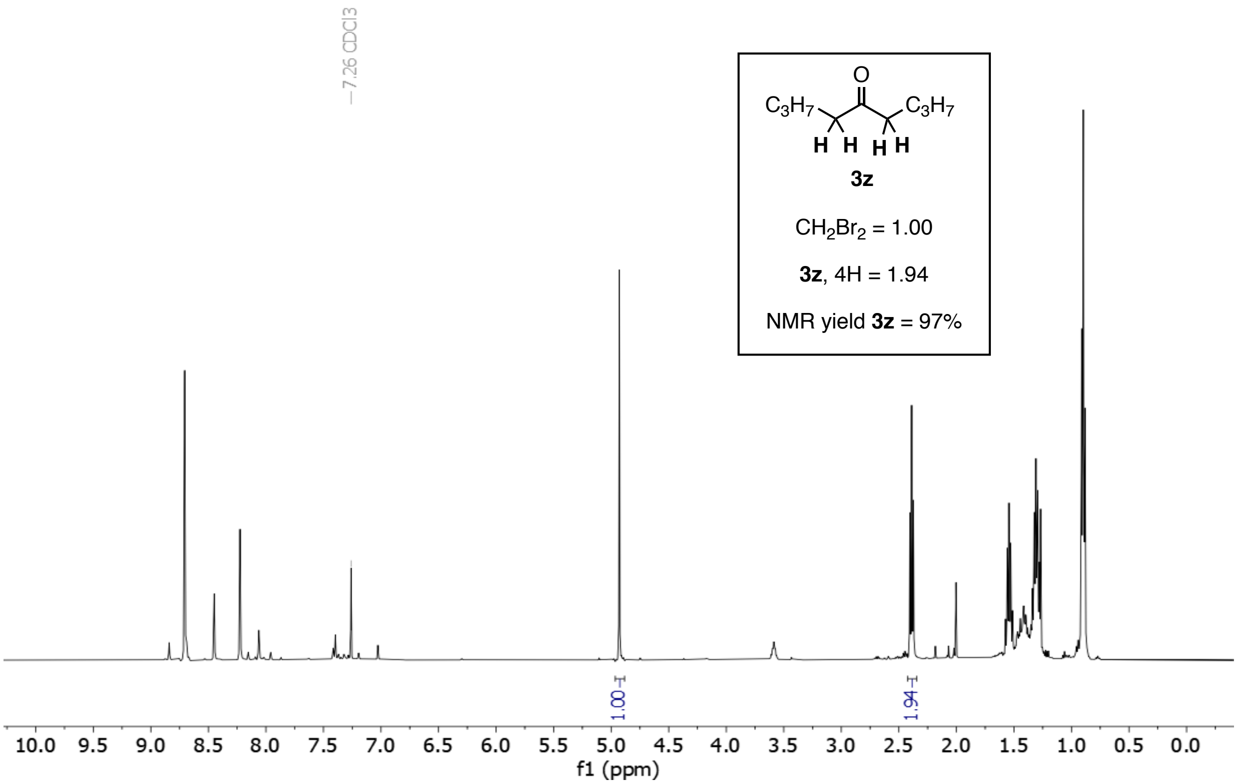

^{1}H NMR (400 MHz, $CDCl_3$) of Octanoic acid (10g)

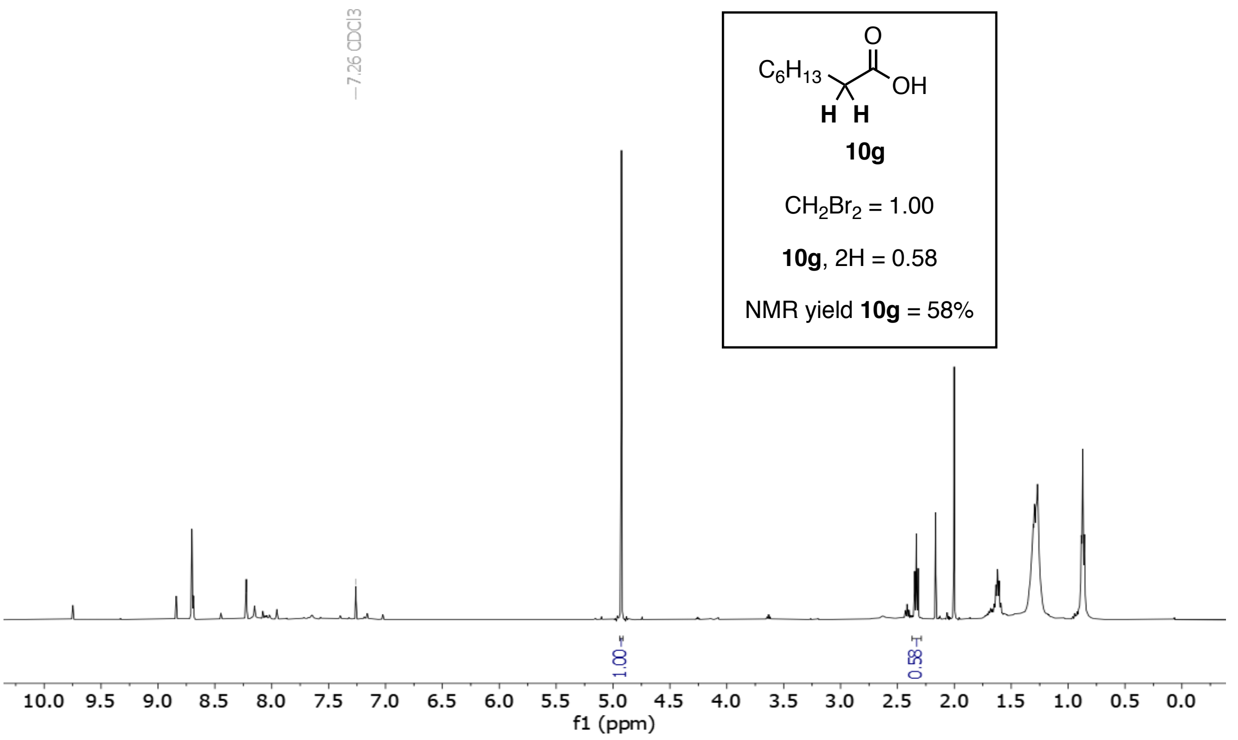

^{1}H NMR (400 MHz, CDCl3) of *N*-cyclohexylbenzamide (11a)

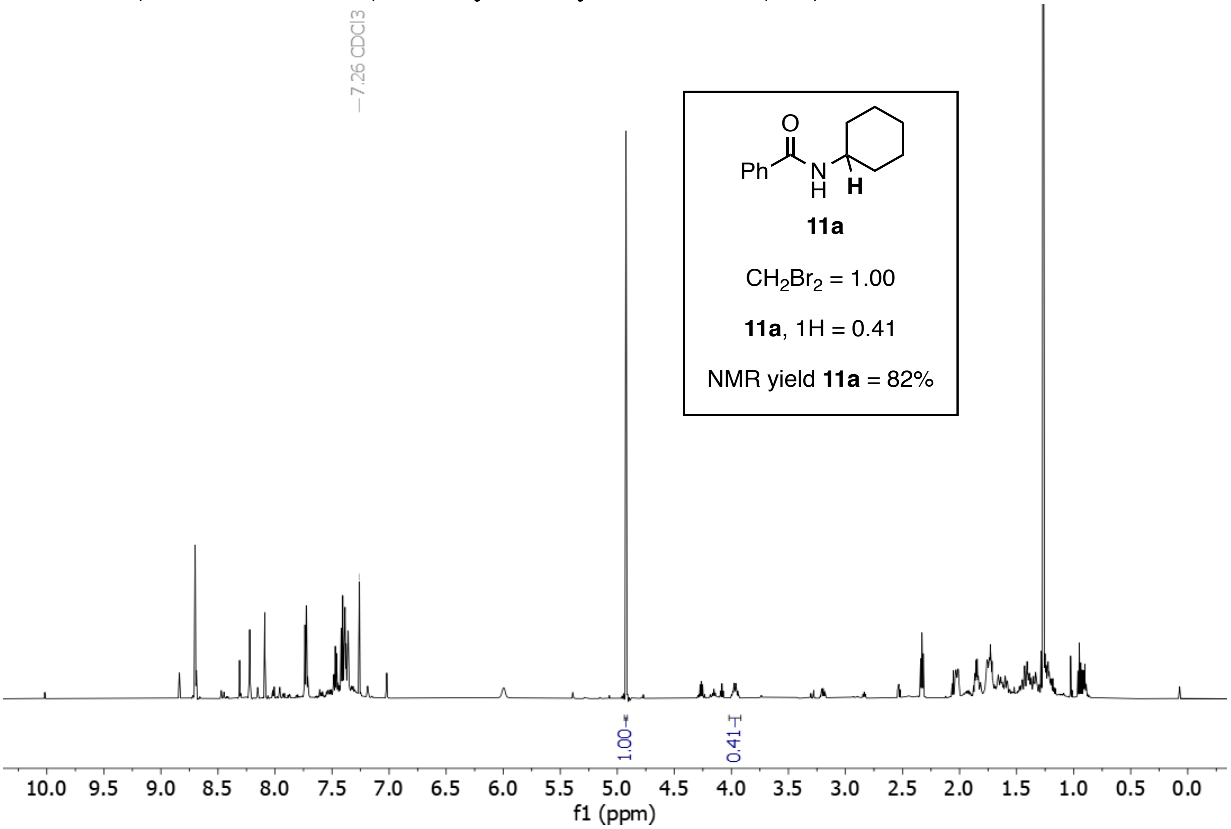

References

1. Lee, K. N.; Lei, Z.; Morales-Rivera, C. A.; Liu, P.; Ngai, M. Y. Mechanistic Studies on Intramolecular C-H Trifluoromethoxylation of (Hetero)Arenes *via* OCF_3-Migration. *Org. Biomol. Chem.* **2016**, *14*, 5599-5605.
2. Min, M.; Bang, G. S.; Lee, H.; Yu, B.-C. A Photoswitchable Methylene-Spaced Fluorinated Aryl Azobenzene Monolayer Grafter on Silicon. *Chem. Commun.* **2010**, *46*, 5232-5234.
3. Ying, Y.; Ye, Z.; Wang, A.; Chen, X.; Meng, S.; Xu, P.; Gao, Y.; Zhao, Y. Nickel-Catalyzed Radical Ring-Opening Phosphorylation of Cycloalkyl Hydroperoxides Leading to Distal Acylphosphine Oxides. *Org. Lett.* **2023**, *25*, 928–932.
4. Chen, Y.-X.; He, J.-T.; Wu, M.-C.; Liu, Z.-L.; Tang, K.; Xia, P.-J.; Chen, K.; Xiang, H.-Y.; Chen, X.-Q.; Yang, H. Photochemical Organocatalytic Aerobic Cleavage of C═C Bonds Enabled by Charge-Transfer Complex Formation. *Org. Lett.* **2022**, *24,* 3920–3925.
5. Obertík, R.; Chudoba, J.; Šturala, J.; Tarábek, J.; Ludvíková, L.; Slanina, T.; König, B.; Cibulka, R. Highly Chemoselective Catalytic Photooxidations by Using Solvent as a Sacrificial Electron Acceptor. *Chem. Eur. J.* **2022**, *28*, e202202487.
6. León Sandoval, A.; Doherty, K. E.; Wadey, G. P.; Schroeder, C. M.; Leadbeater, N. E. Fast, Easy Oxidation of Alcohols Using an Oxoammonium Salt Bearing the Nitrate Anion. *Tetrahedron Lett.* **2023**, *116*, 154332.
7. a) Sandford, C.; Fries, L. R.; Ball, T. E.; Minteer, S. D.; Sigman, M. S. Mechanistic Studies into the Oxidative Addition of Co(I) Complexes: Combining Electroanalytical Techniques with Parameterization. *J. Am. Chem. Soc.* **2019**, *141*, 18877-18889. b) Duran-Camacho, G.; Caleb Hethcox, J. Nickel-Catalyzed Cyanation of (Hetero)Aryl Bromides Using DABAL-Me_3 as a Soluble Reductant. *Org. Lett.* **2022**, *24*, 8397–8400.
8. Politano, F.; Brydon, W. P.; Leadbeater, N. E. Oxidation of α-Trifluoromethyl and Nonfluorinated Secondary Alcohols to Ketones Using a Nitroxide Catalyst. *Synthesis*, **2023**, 1517-1524.
9. Liu, B.; Jin, F.; Wang, T.; Yuan, X.; Han, W. Wacker-Type Oxidation Using an Iron Catalyst and Ambient Air: Application to Late-Stage Oxidation of Complex Molecules. *Angew. Chem., Int. Ed.* **2017**, *56*, 12712-12717.
10. Shih, Y.-L.; Wu, Y.-K.; Hyodo, M.; Ryu, I. Photocatalytic Oxidative Cleavage of Alkenes by Molecular Oxygen: Reaction Scope, Mechanistic Insights, and Flow Application. *J. Org. Chem.* **2022**, *88*, 6548-6552.
11. Zhao, J.; Luo, Z.; Liu, Y.; Xu, J.; Huang, Z.; Xiong, W. Photochemical Oxidation of Alcohols to Ketones or Aldehydes Using DMSO as an Oxidant Without Activated Agent. *Tetrahedron*, **2023**, *131*, 133208.
12. Kamijo, S.; Tao, K.; Takao, G.; Tonoda, H.; Murafuji, T. Photoinduced Oxidation of Secondary Alcohols Using 4-Benzoylpyridine as an Oxidant. *Org. Lett.* **2015**, *17*, 3326–3329.
13. Shee, M.; Singh, N. D. P. Photogenerated Azido Radical Mediated Oxidation: Access to Carbonyl Functionality from Alcohols, Alkylarenes, and Olefins via Organophotoredox. *Adv. Synth. Catal.* **2022**, *364*, 2032-2039.
14. Lal, G. S. Site-Selective Fluorination of Organic Compounds Using 1-alkyl-4-fluoro-1,4-diazabicyclo[2.2.2]octane Salts (Selectfluor Reagents). *J. Org. Chem.* **1993**, *58*, 2791-2796.
15. Lv, W. X.; Zeng, Y. F.; Li, Q.; Chen, Y.; Tan, D. H.; Yang, L.; Wang, H. Oxidative Difunctionalization of Alkenyl MIDA Boronates: A Versatile Platform for Halogenated and Trifluoromethylated α-Boryl Ketones. *Angew. Chem., Int. Ed.* **2016**, *55*, 10069-10073.

16. Dai, W.; Lv, Y.; Wang, L.; Shang, S.; Chen, B.; Li, G.; Gao, S. Highly Efficient Oxidation of Alcohols Catalyzed by a Prophyrin-Inspired Manganese Complex. *Chem. Commun.* **2015**, *51*, 11268-11271.
17. Bergamaschi, E.; Lunic, D.; McLean, L. A.; Hohenadel, M.; Chen, Y. K.; Teskey, C. J. Controlling Chemoselectivity of Catalytic Hydroboration with Light. *Angew. Chem., Int. Ed.* **2022**, *61*, e202114482.
18. a) Fyfe, T. J.; Kellam, B.; Sykes, D. A.; Capuano, B.; Scammells, P. J.; Lane, R.; Charlton, S. J.; Mistry, S. N. Structure-Kinetic Profiling of Haloperidol Analogues at the Human Dopamine D_2 Receptor. *J. Med. Chem.* **2019**, *62*, 9488-9520. b) Wang, J.; Pang, Y.-B.; Tao, N.; Zeng, R.-S.; Zhao, Y. Mn-Enabled Radical-Based Alkyl-Alkyl Cross-Coupling Reaction from 4-Alkyl-1,4-dihydropyridines. *J. Org. Chem.* **2019**, *84*, 15315-15322.
19. a) Li, J.; Jiang, D.-N.; Chen, J.-X.; Liu, M.-C.; Ding, J.-C.; Wu, H.-Y. Eco-Friendly Synthesis of Quinoxaline Derivatives by Grinding Under Solvent-Free Conditions. *J. Heterocycl. Chem.* **2011**, *48*, 403-406. b) Meng, X.; Bi, X.; Yu, C.; Chen, G.; Chen, B.; Jing, Z.; Zhao, P. Ball-Milling Synthesized Hydrotalcite Supported Cu-Mn Mixed Oxide under Solvent-Free Conditions: An Active Catalyst for Aerobic Oxidative Synthesis of 2-Acylbenzothiazoles and Quinoxalines. *Green Chem.* **2018**, *20*, 4638-4644.
20. Dua, S. S.; Gilman, H. Polyhalo-Organometallic and Organometalloidal Compounds: XIX. Some Reactions of Pentachloropyridine with Organometallic Compounds. *J. Organomet. Chem.* **1968**, *12*, 299-303.
21. Moriyama, K.; Nakamura, Y.; Togo, H. Oxidative Debenzylation of N-benzyl Amides and O-benzyl Ethers Using Alkali Metal Bromide. *Org. Lett.* **2014**, *16*, 3812-3815.
22. a) Tanaka, K.; Matsui, S.; Kaji, A. Lithiation of Alkoxyalkyl Phenyl Sulfones. New Approach to Acyl Anion Synthesis. *Bull. Chem. Soc. Jpn.* **1980**, *53*, 3619-3622. b) Tanaka, K.; Uneme, H.; Yamagishi, N.; Tanikaga, R.; Kaji, A. New Methods for Stereoselective Synthesis of α-Alkylidene-γ-butyrolactones Using Monoanion of O-Ethyl S-(Tetrahydro-2-oxo-3-furanyl) Thiocarbonate and Dianion of α-Mercapto-γ-butyrolactone. *Bull. Chem. Soc. Jpn.* **1980**, *53*, 2910-2916.
23. Fuse, H.; Irie, Y.; Fuki, M.; Kobori, Y.; Kato, K.; Yamakata, A.; Higashi, M.; Mitsunuma, H.; Kanai, M. Identification of a Self-Photosensitizing Hydrogen Atom Transfer Organocatalyst System. *J. Am. Chem. Soc.* **2022**, *144*, 6566-6574.
24. Ryland, B. L.; McCann, S. D.; Brunold, T. C.; Stahl, S. S. Mechanism of Alcohol Oxidation Mediated by Copper (II) and Nitroxyl Radicals. *J. Am. Chem. Soc.* **2014**, *136*, 12166-12173.
25. Posner, G. H.; Chapdelaine, M. J. Organic Reactions at Alumina Surfaces. Mild, Selective, and High-Yield Oxidation of Cyclobutanol to Cyclobutanone. *Synthesis* **1977**, *1977*, 555.
26. Zhang, G.; Wen, X.; Wang, Y.; Mo, W.; Ding, C. Sodium Nitrite Catalyzed Aerobic Oxidative Deoximation Under Mild Conditions. *J. Org. Chem.* **2011**, *76*, 4665-4668.
27. Chai, Z.; Zeng, T.-T.; Li, Q.; Lu, L.-Q.; Xiao, W.-J.; Xu, D. Efficient Visible Light-Driven Splitting of Alcohols into Hydrogen and Corresponding Carbonyl Compounds over a Ni-Modified CdS Photocatalyst. *J. Am. Chem. Soc.* **2016**, *138*, 10128-10131.
28. Steves, J. E.; Stahl, S. S. Copper (I)/ ABNO-Catalyzed Aerobic Alcohol Oxidation: Alleviating Steric and Electronic Constraints of Cu/TEMPO Catalyst Systems. *J. Am. Chem. Soc.* **2013**, *135*, 15642-15745.
29. Liu, C.; Li, T.; Dai, X.; Zhao, J.; He, D.; Li, G.; Wang, B.; Cui, X. Catalytic Activity Enhancement on Alcohol Dehydrogenation via Directing Reaction Pathways from Single-to Double-Atom Catalysis. *J. Am. Chem. Soc.* **2022**, *144*, 4913-4924.

30. Zhang, R.; Zhang, R.; Jian, R.; Zhang, L.; Zhang, M.-T.; Xia, Y.; Luo, S.; Bio-Inspired Lanthanum-Ortho-Quinone Catalysis for Aerobic Alchol Oxidation: Semi-Quinone Anionic Radical as Redox Ligand. *Nat. Commun.* **2022**, *13*, 428.
31. Huang, Z.; Guan, R.; Shanmugam, M.; Bennett, E. L.; Robertson, C. M.; Brookfield, A.; McInnes, E. J.; Xiao, J. Oxidative Cleavage of Alkenes by O_2 with a Non-Heme Manganese Catalyst. *J. Am. Chem. Soc.* **2021**, *143*, 10005-10013.
32. Li, Z.; Sun, W.; Wang, X.; Li, L.; Zhang, Y.; Li, C. Electrochemically Enabled, Nickel-Catalyzed Dehydroxylative Cross-Coupling of Alcohols with Aryl Halides. *J. Am. Chem. Soc.* **2021**, *143*, 3536-3543.
33. Schulz, G.; George, V.; Taser, D.; Kirschning, A. Taming Bromine Azide for Use in Organic Solvents─ Radical Bromoazidations and Alcohol Oxidations. *J. Org. Chem.* **2023**, *88*, 3781-3786.
34. Castner, E.; Dickson, M.; Mykytyn, A.; Seeram, N. P.; Henry, G. E.; Vivekanand, P. Synthesis and Evaluation of Apoptotic Induction of Human Cancer Cells by Ester Derivatives of Thujone. *Med. Chem. Res.* **2020**, *29*, 268-280.
35. Thamm, I.; Richers, J.; Rychlik, M.; Tiefenbacher, K. A Six-Step Total Synthesis of α-Thujone and d_6-α-Thujone, Enabling Facile Access to Isotopically Labelled Metabolites. *Chem. Commun.* **2016**, *52*, 11701-11703.
36. Wang, L.; Shang, S.; Li, G.; Ren, L.; Lv, Y.; Gao, S. Iron/ABNO-catalyzed Aerobic Oxidation of Alcohols to Aldehydes and Ketones Under Ambient Atmosphere. *J. Org. Chem.* **2016**, *81*, 2189-2193.
37. Wang, Y.; Huang, Z.; Leng, X.; Zhu, H.; Liu, G.; Huang, Z. Transfer Hydrogenation of Alkenes Using Ethanol Catalyzed by a NCP Pincer Iridium Complex: Scope and Mechanism. *J. Am. Chem. Soc.* **2018**, *140*, 4417-4429.
38. Lewis, S. G.; Dadum, A, G.; McLean, D.; Buenavista, J.; Myers, J.; Lambert, K. M.; Fair, J. D. Chemoselective Oxidation of Alcohols in the Presence of Amines Using an Oxoammonium Salt. *Tetrahedron*, **2023**, *131*, 133226.
39. Junor, G. P.; Romeo, E. A.; Chen, X.; Jazzar, R.; Bertrand, G. Readily Available Primary Aminoboranes as Powerful Reagents for Aldimine Synthesis. *Angew. Chem., Int. Ed.*, **2019**, *58*, 2875-2878.
40. Cui, X.; Li, W.; Junge, K.; Fei, Z.; Beller, M. Selective Acceptorless Dehydrogenation of Primary Amines to Imines by Core-Shell Cobalt Nanoparticles. *Angew. Chem., Int. Ed.*, **2020**, *59*, 7501-7507.
41. Chen, D.; Xu, G.; Zhou, Q.; Chung, L. W.; Tang, W. Practical and Asymmetric Reductive Coupling of Isoquinolines Templated by Chiral Diborons. *J. Am. Chem. Soc.* **2017**, *139*, 9767-9770.
42. Zhao, C.; Glazier, D. A.; Yang, D.; Yin, D.; Guzei, I. A.; Aristov, M.M.; Liu, P.; Tang, W. Intermolecular Regio- and Stereoselective Hetero-[5+2] Cycloaddition of Oxidopyrylium Ylides and Cyclic Imines. *Angew. Chem., Int. Ed.* **2019**, *58*, 887-891.
43. Kondo, Y.; Hirazawa, Y.; Kadota, T.; Yamada, K.; Morisaki, K.; Morimoto, H.; Ohshima, T. One-Pot Catalytic Synthesis of a-Tetrasubstituted Amino Acid Derivative via *in situ* Generation of N-Unsubstituted Ketimines. *Org. Lett.* **2022**, *24*, 6594-8598.
44. a) Tzani, M. A.; Fountoulaki, S.; Lykakis, I. N. Polyoxometalate-Driven Ease Conversion of Valuable Furfural to *trans-N,N*-4,5-Diaminocyclopenten-2-ones. *J. Org. Chem.* **2022**, *87*, 2601-2615. b) Tillack, A.; Jiao, H.; Castro, I.G.; Hartung, C. G.; Beller, M. A General Study of [(η^5-Cp′)$_2$Ti(η^2-$Me_3SiC_2SiMe_3$)]-Catalyzed Hydroamination of Terminal Alkynes: Regioselective

formation of Markovnikov and Anti-Markovnikov Products and Mechanistic Explanation (Cp′=C_5H_5, C_5H_4Et, C_5Me_5). *Chem. Eur. J.* **2004**, *10*, 2409-2420.
45. Horvat, M.; Iskra, J. Oxidative Cleavage of C-C Double Bond in Cinnamic Acids with Hydrogen Peroxide Catalyzed by Vanadium (V) Oxide. *Green Chem.* **2022**, *24*, 2073-2081.
46. Ma, C.; Zhao, C.-Q.; Xu, X.-T.; Li, Z.-M.; Wang, X.-Y.; Zhang, K.; Mei, T.-S. Nickel-Catalyzed Carboxylation of Aryl and Heteroaryl Fluorosulfates Using Carbon Dioxide. *Org. Lett.* **2019**, *21*, 2464-2467.
47. Shee, M.; Shah, S. S.; Singh, N. D. P. Organophotoredox Assisted Cyanation of Bromoarenes *via* Silyl-Radical-Mediated Bromine Abstraction. *Chem. Commun.* **2020**, *56*, 4240-4243.
48. Wang, Z.-Q.; Tang, X.-S.; Yang, Z.-Q.; Yu, B.-Y.; Wang, H.-J.; Sang, W.; Yuan, Y.; Chen, C. Highly Active Bidentate N-Heterocyclic Carbene/Ruthenium Complexes Performing Dehydrogenative Coupling of Alcohols and Hydroxides in Open Air. *Chem. Commun.* **2019**, *55*, 8591-8594.
49. Xiao, G.; Xie, C.; Guo, Q.; Zi, G.; Hou, G.; Huang, Y. Nickel-Catalyzed Asymmetric Hydrogenation of γ-Keto Acids, Esters, and Amides to Chiral γ-Lactones and γ-Hydroxy Acid Derivatives. *Org. Lett.* **2022**, *24*, 2722-2727.
50. Kaicharla, T.; Thangaraj, M.; Biju, A. T. Practical Synthesis of Phthalimides and Benzamides by a Multicomponent Reaction Involving Arynes, Isocyanides, and CO_2/H_2O. *Org. Lett.* **2014**, *16*, 1728-1731.
51. Forni, J. A.; Micic, N.; Connell, T. U.; Weragoda, G.; Polyzos, A. Tandem Photoredox Catalysis: Enabling Carbonylative Amidation of Aryl and Alkylhalides. *Angew. Chem., Int. Ed.* **2020**, *59*, 18646-18654.
52. Zhang, W.; Smillovich, J.; Albert, V. Palladium Catalyzed Amidation of Phenyl Carboxylates and Anilines Using Aqueous Micellar Catalysis. *Tetrahedron Lett.* **2023**, *114*, 154242.
53. Chatterjee, B.; Gunanathan, C. Ruthenium Catalyzed Selective α- and α,β-Deuteration of Alcohols Using D_2O. *Org. Lett.* **2015**, *17*, 4794–4797.
54. Ranu, B. C.; Banerjee, S. Indium Triflate Catalyzed Rearrangement of Aryl-Substituted Cyclopropyl Carbinols to 1,4-Disubstituted 1,3-Butadienes. *Eur. J. Org. Chem.* **2006**, *2006*, 3012-3015.
55. a) Motiwala, H. F.; Gülgeze, B.; Aubé, J. Copper-Catalyzed Oxaziridine-Mediated Oxidation of C-H Bonds. *J. Org. Chem.* **2012**, *77*, 7005-7022. b) Mizar, P.; Wirth, T. Flexible Stereoselective Functionalizations of Ketones through Umpolung with Hypervalent Iodine Reagents. *Angew. Chem., Int. Ed.* **2014**, *53*, 5993-5997.
56. Beddoe, R. H.; Edwards, D. C.; Goodman, L.; Sneddon, H. F.; Denton, R. M. Synthesis of ^{18}O-Labelled alcohols from Unlabelled Alcohols. *Chem. Commun.* **2020**, *56*, 6480-6483.
57. Wang, J.-L.; Li, H.-J.; Wang, H.-S.; Wu, Y.-C. Regioselective 1,2-Diol Rearrangement by Controlling the Loading of $BF_3 \cdot Et_2O$ and its Application to the Synthesis of Related Nor-Sesquiterene- and Sesquiterene-Type Marine Natural Products. *Org. Lett.* **2017**, *19*, 3811-3814
58. Song, Q.-W.; Zhao, Q.-N.; Li, J.-Y.; Zhang, K.; Liu, P. Selective Conversion of CO2 and Switchable Alcohols into Linear or Cyclic Carbonates via Versatile Zinc Catalysis. *Synthesis* **2019**, *51*, 739-746.
